# Quantifying the Denticle Multiverse: A Standardized Coding System to Capture Three Dimensional Morphological Variations for Quantitative Evolutionary and Ecological Studies of Elasmobranch Denticles

**DOI:** 10.1093/iob/obaf021

**Published:** 2025-05-13

**Authors:** L D Rubin, G J Fraser, M K Gabler-Smith, G V Lauder, W V Ribeiro, D F B Vaz, N Wallis-Mauro, E C Sibert

**Affiliations:** Department of Environmental Biology, The State University of New York College of Environmental Science and Forestry, Syracuse, NY 13210, USA; Department of Geology and Geophysics, Woods Hole Oceanographic Institution, Woods Hole, MA 02543, USA; Department of Biology, University of Florida, Gainesville, FL 32611, USA; Museum of Comparative Zoology, Harvard University, Cambridge, MA 02138, USA; Museum of Comparative Zoology, Harvard University, Cambridge, MA 02138, USA; Brown University Department of Earth, Environment and Planetary Sciences, Providence, RI 02912, USA; Natural History Museum, London SW7 5BD, UK; Department of Geology and Geophysics, Woods Hole Oceanographic Institution, Woods Hole, MA 02543, USA; Department of Biology, University of Florida, Gainesville, FL 32611, USA; Department of Geology and Geophysics, Woods Hole Oceanographic Institution, Woods Hole, MA 02543, USA

## Abstract

Dermal denticles—microscopic tooth-like scales—are a major defining feature of elasmobranch skin, and are of interest to a wide array of fields, including paleontology, evolutionary biology, developmental biology, functional morphology, and bio-inspired design. While dermal denticle research is a growing field, there is currently no standardized vocabulary or framework to compare denticle morphology across research fields, siloing, and limiting denticle research efforts. Here, we present a morphological framework, which includes a character code that comprehensively captures denticle morphology from a wide diversity of denticle sampling types and imaging methods, and is backed by an easy-to-use google sheets-based coding tool and R package for replicating disparity analyses. The code is based on a wide-spread literature review of published denticle images, scanning electron microscope (SEMs), and computed tomography (CT) scans of extant shark denticles, and a review of tens of thousands of fossil denticles from pelagic ocean sediments dating back over 100 million years. The code's flexibility and replicability facilitate comparison across studies and independent research teams, and the addition of novel character categories. Denticle morphotypes are defined as denticles with unique combinations of character traits. This coding system facilitates morphologically backed disparity analyses of denticle morphological diversity, whether through deep time, across the body of a shark, or across a time-series of development, providing a more detailed, quantitative, and universal tool for analyzing denticle morphology across studies.

## Abstract (Fijian)

Na yago ni matavuvale vaka ika me vaka na qio kei na vai e dau dikevi vakawasoma e na veiqaravi nei ira e ra dau vakadidike e na veimataqali vuli e so. Me vaka na vuli veika vaka-ivakarau makawa, na vuli ni veisau ni tuvaki vakayago, na vuli e dau dikeva na duidui ni kena vakayagataki na veitikini yago ni manumanu kei na ituvatuva e dusia na veika e dau vakayacori ena bula kei na nodra itovo na manumanu (bio-inspired design).

Na kena toso na vakadidike ni vuli me baleta na yago ni vuvale vaka ika oqo, e kunekune dredre tiko na kena vakatauvatani na veika e dikevi baleta ni sega ni tiko e dua na yavu dei me cokona vata na veimataqali vuli e sa cavuti oti. Ka mai vakavuna na qiqo ni vakadidike kei na kena vakayagataki na kena ivosa matau e so. E na vuku ni vakadidike oqo, keitou sa vakarautaka e dua na sala vou e rawa ni semata kina na i tukutuku lailai me vaka na yaca ni ika, na kena ibulibuli kei na kena yaga me kilai kina na kedra duidui. Na sala keitou vakayacora e kumuna vata na veiporokaramu e so e na mona livaliva me vaka na google sheet kei na R.

Na yaga ni porokaramu e vukea na kena soqoni ka dikevi na duidui ni tukutuku e kumuni rawa e na vakadidike oqo. Na mona ni porokaramu oqo e vukea na kena waki vata na itukutuku, na itaba, na SEMs kei na yaloyalo vakamatailalai (CT scans) ni yagodra na qio kei na kena vakadikevi lesu na batidra e kune e na boto ni sauloa ka tu mai na dua na drau na milioni na yabaki sa oti.

Na mona ni porokaramu o ya e vakarawarawataka na vakatauvatani ni vakadidike kei na tukutuku e ra kumuna na vei matailawalawa ni vakadidike tudei e liu, ka vakacurumi kina e dua na iwasewase vou ni ivakatakilakila e so.

Na mona ni porokaramu oqo e vakararamataka na kena dikevi na kena tuvaki ni kuli ni yago ni veimataqali ika mai na matavuvale ni qio kei na vai. E rawa ni vakayagataki talega e na kena vakatauvatani ni kuli ni yagodra e na balavu ni gauna e ra bula

tu kina se kena bula voli e na veitaba gauna. Na mona ni porokaramu oqo e na vukea na kena kilai vakatitobu kei na kena vakatauvatani na i tuvaki ni kuli ni matavuvale ni ika oqo e na vei vuli eso.

## Abstract (Portuguese)

Dentículos dérmicos - escamas microscópicas semelhantes a dentes - são uma característica diagnostica importante da pele dos elasmobrânquios e são de interesse para uma ampla variedade de áreas, incluindo paleontologia, biologia evolutiva, biologia do desenvolvimento, morfologia funcional e design bioinspirado. Embora a pesquisa sobre dentículos dérmicos seja uma área em crescimento, atualmente não existe um vocabulário ou estrutura padronizada para comparar a morfologia dos dentículos entre diferentes áreas de pesquisa, o que acaba isolando e limitando os esforços de pesquisa sobre dentículos. Aqui, apresentamos uma estrutura morfológica que inclui um código de caracteres que captura de maneira abrangente a morfologia dos dentículos a partir de uma grande diversidade de tipos de amostras de dentículos e métodos de imagem, e que é respaldada por uma ferramenta de codificação fácil de usar baseada no Google Sheets e por um pacote R para replicar análises de disparidade. O código é baseado em uma ampla revisão da literatura de imagens publicadas de dentículos, MEVs e tomografias computadorizadas (CT) de dentículos de tubarões viventes, além de uma revisão de dezenas de milhares de dentículos fósseis de sedimentos oceânicos pelágicos com mais de 100 milhões de anos. A flexibilidade e replicabilidade do código facilitam a comparação entre estudos e equipes de pesquisa independentes, além da adição de novas categorias de caracteres. Os morfotipos de dentículos são definidos como dentículos com combinações únicas de características. Este sistema de codificação facilita análises de disparidade com base morfológica sobre a diversidade morfológica dos dentículos, seja ao longo do tempo geológico, ao longo do corpo de um tubarão, ou ao longo de uma série temporal de desenvolvimento, oferecendo uma ferramenta mais detalhada, quantitativa e universal para analisar a morfologia dos dentículos em diferentes estudos.

## Abstract (Spanish)

Cuantificación del multiverso de dentículos: Un sistema de codificación estandarizado para capturar la variación morfológica tridimensional para estudios cuantitativos evolutivos y ecológicos de dentículos dérmicos de elasmobranquios.

Los dentículos dérmicos (escamas microscópicas similares a dientes) son una característica definitoria importante de la piel de los elasmobranquios y son de interés para una amplia gama de campos, incluyendo la paleontología, la biología evolutiva, la biología del desarrollo, la morfología funcional y la ingeniería biomimética. Mientras la investigación de los dentículos dérmicos es un campo en expansión, actualmente no existe un vocabulario ni un marco estandarizado para comparar la morfología de los dentículos en diferentes campos de estudio, lo que aísla y limita los esfuerzos de investigación. Aquí se presenta un marco morfológico que incluye un código de caracteres que captura exhaustivamente la morfología de los dentículos dérmicos a partir de una amplia diversidad de tipos de muestreo y métodos de imagen. Este marco está respaldado por una herramienta de codificación fácil de usar basada en hojas de cálculo de Google y un paquete R para replicar análisis de disparidad. El código se basa en una extensa revisión bibliográfica de imágenes publicadas, microscopía electrónica de barrido (MEB) y tomografías computarizadas de dentículos de tiburón existentes, y en una revisión de decenas de miles de dentículos fósiles de sedimentos oceánicos pelágicos que datan de más de 100 millones de años. La flexibilidad y replicabilidad del código facilitan la comparación entre estudios y equipos de investigación independientes, así como la incorporación de nuevas categorías de caracteres. Los morfotipos de dentículos se definen como dentículos que presentan combinaciones únicas de rasgos de carácter. Este sistema de codificación facilita el análisis de disparidad morfológica de la diversidad de los dentículos, ya sea a lo largo de grandes períodos de tiempo, a lo largo del cuerpo de un tiburón o a lo largo de una serie temporal de desarrollo, proporcionando una herramienta más detallada, cuantitativa y universal para el análisis de la morfología de los dentículos dérmicos en diferentes estudios.

## Introduction

Sharks are important marine predators facing global population declines due to anthropogenic pressures, including exploitation, habitat degradation, and climate change (Dulvy et al. 2014; Dulvy et al. 2021; Pacoureau et al. 2021; Jorgensen et al. 2022). There are more than 500 extant shark species that inhabit a diverse range of habitats, from coastal coral reefs to the deep sea. Found in all modern ocean basins, sharks fill a range of vital ecological niches (Dedman et al. 2024). They display diverse feeding strategies, which range from the large planktivorous basking sharks and whale sharks, to the apex predators people are most familiar with, to deep-sea scavenging sleeper sharks. As apex predators, sharks structure community dynamics via direct and indirect food web interactions and influence the behavior of other animals through predator avoidance strategies (Heithaus et al. 2008). Furthermore, sharks hold significant cultural and economic importance. In their 2015 report, *State of the global market for shark products*, the FAO reported that the value of shark related products (including but not limited to meat, prepared jaws, and liver oil) was approaching 1 billion USD annually. This number does not include the rapidly growing sector of shark-related tourism, which has an estimated annual value of 314 million USD (Healy et al. 2020).

While the ecological and cultural importance of sharks is well demonstrated, they present unique challenges to scientific study; their behavior and diverse range of habitats, as well as large home ranges, can make them difficult to survey in modern ecosystems (Dillon et al. 2017). Furthermore, sharks have cartilaginous skeletons, making full body fossils relatively rare, limiting evolutionary studies (Dillon, Norris, and O’ Dea 2017; Whitenack et al. 2022; Cooper et al. 2023). In addition, there is currently limited understanding of the developmental and genetic basis of morphological variation in sharks, and chondrichthyans more generally. However, genomic and transcriptomic analyses have begun to circulate, offering descriptions of phenotypic distinction with the clade of cartilaginous fishes and the variety among chondrichthyes (Venkatesh et al. 2014; Hara et al. 2018; Marra et al. 2019; Kuraku 2021; Mayeur et al. 2024). Importantly, our understanding of the developmental genetic aspects of tooth emergence and regeneration, skin denticle morphogenesis, and sensory appendage patterning in sharks have received more attention (Martin et al. 2016; Cooper et al. 2018; Thiery et al. 2022; Zimm et al. 2023; Nicklin et al. 2024). However, the link between genetics, development, and morphology remains poorly understood in the clade, limiting our ability to reconstruct ancestral states or understand the true breadth of morphological variation (e.g., Gayford 2023).

Conversely, shark teeth do fossilize and researchers have studied the paleo and historical ecology of sharks by analyzing the morphology of their teeth to identify evolutionary relationships and taxonomy (e.g., Ginter et al. 2010; Cappetta and Schultze 2012; Grogan et al. 2012; Bazzi 2021). Studies using fossil shark teeth have focused primarily on taxonomy and biology, while investigating questions of ecology or population estimates is more challenging, though possible (eg. Kim et al. 2022). Sharks are covered in dermal denticles, small placoid scales encased in a hard enamel—the same general composition as their teeth (Cooper et al. 2023). Since enamel is generally well-preserved in the sedimentary record, dermal denticles are one of the best preserved and most complete fossil records for sharks, and offer insight into both the modern and paleoecology of sharks (Dillon et al. 2017; Sibert and Rubin 2021). Denticles are also significantly smaller than oral teeth and additionally cover the entire shark body: Thus, denticles are many orders of magnitude more abundant in the fossil record than oral teeth, albeit preserved within a much smaller size fraction (e.g., 38–500 µm). Their exceptional preservation potential means they can be found in deep ocean sediment cores, terrestrial fossil sites, and coral reef rubble (Doyle and Riedel 1985; Ginter et al. 2010; Sibert et al. 2016; Dillon, Norris, and O'Dea 2017; Dillon et al. 2020). Recent work has utilized morphometric methods to compare isolated denticles to denticles from known modern taxa as a way to understand shark ecology through time scales ranging from millions of years ago to present day (Sibert and Rubin 2021; Dillon 2022); however, these methods have focused only on small subsets of denticle morphology and are not generalizable beyond the specific study system in which they were applied, making cross-study comparisons impossible.

By 1900, researchers had identified fossil dermal denticles from Carboniferous deposits in Ohio and had begun theorizing the potential for denticles to illuminate ancient elasmobranch biology and evolution (Dean 1894, 1895). Indeed, dermal denticles are thought to be some of the oldest known vertebrate fossils (Turner 2004; Sansom et al. 2012). In more recent years, dermal denticles have gained attention for their hydrodynamic properties (Wen et al. 2015; Ankhelyi et al. 2018). Analysis of denticles from museum specimens have been used to clarify delimitation among closely related species and assess their taxonomic validity (Vaz and Carvalho 2013, 2018; White et al. 2013, 2019, 2022; Vaz 2021; Vaz et al. 2023). Denticles are also increasingly being used to study the historical and prehistoric ecology of sharks with geographic focuses in the coastal Caribbean and Pacific open ocean using fossil reefs and deep-sea sediment cores. There are a few researchers working on microfossil denticles at present, ranging in time from Holocene to Paleozoic and from coral reefs to the deep sea. These complementary efforts have contributed to this evolving field and include denticle abundance calibration work as well as fossil time series reconstructions of shark diversity and ecology through time. For example, an aquarium-based observational study found that denticle shedding rates differed by taxa (Dillon et al. 2022) and an *in-situ* coral reef study found that modern shark presence corresponds to denticles found in surface sediments (Dillon et al. 2020). Deep-sea sediment-based studies found a large and significant extinction of denticle morphotypes in the early Miocene (Sibert and Rubin 2021) and coral reef-based studies have revealed significant shifts in shark abundance and ecological groups in the Caribbean corresponding with human impacts (Dillon et al. 2021).

Denticle morphology has been a focus of researchers across a variety of disciplines over the last century. For example, one of the most influential denticle morphology researchers of the 20th century, Wolf-Ernst Reif, imaged and described denticles representing a wide range of both taxonomic and ontogenetic diversity (1985) in his in-depth monograph “The Squamation and Ecology of Sharks,” which documented denticle diversity of a subset of extant sharks. Further, denticles have long been studied in early Paleozoic deposits as evidence of some of the earliest vertebrates (Turner 2004; Sansom et al. 2012). While research examining denticle morphology has grown in prominence both in the paleontological and modern morphological and developmental spheres, cross-study and cross-disciplinary comparisons have proven difficult due to inconsistencies in the language and methods employed to image and describe denticle morphology. Furthermore, while morphological character coding of denticles is not new (see Doyle and Riedel 1979; Dillon, Norris, and O'Dea 2017), prior studies have been restricted to a low diversity of denticles from localized studies or just a few limited taxa, and only include images, sample types, and preservation that are typical in their distinct field of study. For example, researchers studying elasmobranch skin patches from fully articulated specimens have the benefit of knowing the denticle's directionality relative to the organism's body and generally have access to the entire denticle's morphology (including base, peduncle, and crown). In contrast, paleontologists work primarily with disarticulated denticles where the subcutaneous base is rarely preserved, and therefore must focus exclusively on denticle crowns. Further, as denticles in the fossil record are nearly always disarticulated, denticle orientation, ontological stage, and taxonomic identity are unknown quantities, limiting fossil studies to observable morphological information independent of the organismal context. To integrate across these disciplinary and preservational boundaries, a common language, which applies across all sampling and observational strategies, is essential. For these reasons, this paper establishes a standardized but flexible scheme for the identification and description of denticles, which facilitates capturing variation in both currently known morphologies, while allowing for integration of as yet undiscovered morphotypes into the system. Further, such a morphological code allows for quantitative exploration of denticle morphology across ontogenetic series (Vaz et al. 2023), body position (Ankhelyi et al. 2018), between taxa (Reif 1985; Deynat and Séret 1996), and provides a basis for linking isolated fossils to known taxonomic, ecological, and functional characteristics of the sharks that may have produced them (Dillon et al. 2017; Sibert and Rubin 2021).

Here, we present a comprehensive morphological character coding system that can be used to describe the currently known range of dermal denticle morphological variation, drawing on datasets from deep-sea sediment cores, museum collections, coral reef sediments, and the scientific literature. This coding system integrates prior coding schemes and expands to include an extensive array of currently known denticle morphologies, both modern and fossil, drawing on the published scientific literature as well as microfossil collections, to place them in a common comparative morphological framework. This framework is flexible, and allows for expansion as new facets of denticle morphology are observed, while accurately capturing all of the currently observed variation in denticle morphological diversity. Further, while the majority of character states are observed in the current dataset, the code does include some currently hypothetical states that are intermediates or clear extensions of currently observed denticle character states. To facilitate use and cross-study comparisons, we also have developed an R package, *ichthyoliths*, which can be used to calculate denticle disparity using the morphometric code, and compare denticle morphology across time, space, taxonomy, and ontogeny.

## The morphological character code

Denticles have a wide range of morphological variability; however, most discussions of denticle morphological variation have been restricted to within-individual or intraspecific variation (Reif 1985; Gravendeel et al. 2002; Castro 2011; Ankhelyi et al. 2018; Popp et al. 2020). A few studies have addressed denticle morphology across multiple shark species that inhabit the same local environment (Dillon et al. 2020; Appleby et al. 2024). However, beyond simply documenting skin samples (Reif 1985; Castro 2011), there has been no comprehensive effort to place all known dermal denticles within a single morphological framework, to allow for broad-scale comparisons, a significant need within the growing community of denticle researchers.

To address this, we developed a coding scheme by describing the characteristics of both fossil and modern dermal denticles from both coastal and open ocean sharks, drawing on a wide-spread literature review (Reif 1985; Gravendeel et al. 2002; Serra-Pereira et al. 2008; Marshall et al. 2009; Castro 2011; Vaz and Carvalho 2013, 2018; White et al. 2015; Weigmann et al. 2016; Dillon, Norris, and O'Dea 2017; Ankhelyi et al. 2018; Feichtinger et al. 2021; Gabler-Smith et al. 2021; Vaz 2021; Dillon 2022; Lourtie et al. 2022) as well as denticles collected during deep-sea sediment processing for other ichthyoliths (Sibert and Norris 2015; Sibert et al. 2016, 2018, 2020; Sibert and Rubin 2021). In addition to the >1500 fossil denticles reviewed spanning 80 million years, the literature-based denticle dataset contains dermal denticles from 181 extant species, representing 31 families from all 8 orders of sharks and 2 orders of rays. These species range from small, coastal sharks to large and migratory species, inhabiting the open ocean and deep sea. For 69 of these species, we included multiple images of denticles from across their body, and in 9 species, we coded their embryonic as well as adult denticles (Reif 1985; Gravendeel et al. 2002; Serra-Pereira et al. 2008; Marshall et al. 2009; Castro 2011; Motta et al. 2012; Vaz and Carvalho 2013; Weigmann et al. 2016; Ankhelyi et al. 2018; Feichtinger et al. 2021; Gabler-Smith et al. 2021; Vaz 2021; Lourtie et al. 2022). This modern literature review included 16 publications, which included a variety of imaging techniques, including Scanning Electron Microscopy, MicroCT, GelSight Imaging, and both transmitted and reflected microscopy. Papers also differed in the number and locations across the body they imaged, leading to a non-standardized dataset, which provided examples of denticle diversity, while making cross-species or even within-species comparisons nearly impossible.

**Fig. 1:**
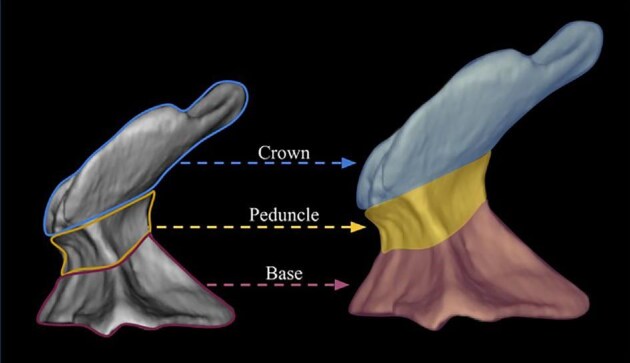
Major parts of a denticle include the crown, the peduncle, and the base; shown here on a spiracle denticle from a Portuguese dogfish *Centroscymnus coelolepis* (MCZ 38297, juvenile male 650 mm TL) and orientated with the anterior to the left and the posterior to the right (After Vaz et al. 2023).

We define 46 traits for denticle morphology (A.1: Overall shape–O.10: Mounding), which can be further generalized as falling under crown general shape, ridge characteristics, texture, and base characteristics (see Fig. 1 for a diagram of the crown, base, and peduncle, the three major portions of a denticle). The majority of traits quantify variability in the ridges observed on most denticle crowns, which vary in number, shape, length, width, and orientation. We outline these characters, and their identification and significance, below. Additionally, we have designed this code and documentation to make it generally flexible and easily replicable with other samples, as well as facilitate the addition of novel character states or even additional characters, as additional diversity is observed. To facilitate utilization of the toolkit, we have developed a google spreadsheet, which can be used to code denticles, with dropdown menus for character states, and have developed an R package, *ichthyoliths*, which integrates with the google sheet output and can be used for analysis across studies within the common coding scheme. The character coding scheme described in this manuscript has the version number *denticles_v0.5*, and prior published and internal use versions of the character coding system included in the R package as historical versions v0.1, v0.2, and v0.4. Subsequent publications, which update the character coding scheme with additional characters and states will be assigned updated version numbers; however, all version documentation, coding schemes, and distance matrices are included in updated versions all of the package, to facilitate replication of prior studies. The *ichthyoliths* R package also contains a similar morphometric coding system and disparity calculation for quantifying morphological variation in fish teeth, which have a similar but even more numerically abundant microfossil record compared to denticles. A forthcoming manuscript developed in parallel will detail the tooth morpholgy system.

The characters and states in this code are meant to be informative and verbally descriptive while capturing as much morphological diversity as possible. Although a “perfect” morphometric code would have all characters and states completely independent from one another, the characters in this code are occasionally nested. For example, one has to acknowledge whether or not a denticle has ridges before describing the many and varying possible shapes, sizes, structures, and directions of those ridges. Independence within characters (e.g., between states) has been carefully curated to ensure no overlapping states exist within a single character. To deal with this pseudo-independence, specific nested characters are down-weighted depending on their relative independence, with the first character describing a feature (e.g., E1 = cusps) carrying a weight of 1, and all subsequent nested characters (e.g., E2, E3, and E4 describe the cusps) weighted at 0.5. This allows the disparity calculation to not overweight the absence of a character (e.g., absence of ridges being coded in dozens of characters) while also accounting for variability within that trait when present. Further, while the first character in a character-group codes a “lack” of the character as a state value of 1, the nested characters record that lack of the character as a state value of 0, which is then discounted in the disparity analyses, reducing the overall weight of the character group in the disparity calculations. Equivalently, a value of 0 is given when a denticle is too broken or obscured to code a particular character, which allows for these denticles to be included in a reduced fashion in the disparity analyses. This handling of 0’s in the disparity calculation allows partial or broken denticles to be compared on their observable character states without counting the non-observable character states as additional disparity. This analytical pathway does have the potential to lump broken denticles together, as each denticle is compared within the disparity calculation based only on the Venn diagram overlap of codable traits; however, this generally only happens when denticles are extremely broken and end up under the broad catch-all morphotypes of “generic linear” and “generic geometric,” and sensitivity tests systematically removing characters from the analysis demonstrate that it is able to robustly group broken denticles with better preserved representatives of their morphotype.

Although the vast majority of denticle character states described here are based on observed character states, we do include some currently hypothetical character states because it is our experience that as more denticles are observed, we see additional character states that are clearly independent from those that exist already along specific axes of morphological variation (e.g., if there are denticles with 1 cusp, denticles with 3 cusps, and denticle with 8 cusps, we assume there will also be denticles with 2 and 6 cusps)—having these options already in the code facilitates future code updates and allows for additional flexibility in the analyses.

## Section 2: Denticle morphological characters

The denticle morphological code includes a total of 46 characters (see Appendix 2  *for illustrations of these characters and their states*). We have grouped different traits together into general categories, or letter groups, so related traits may fall under the same letter, e.g., A1 and A2 are both related to denticle shape. These broad categories are: Overall Shape (A), Anterior/Posterior characteristics (B and C), Symmetry (D), Cusps (E), Overall Ridge Characteristics (F–H), Central Ridge characteristics (I and J), Central Ridge System (K), Ridge Size (L), Depressions and Dimples (M), Surface texture (N), and Base characteristics (O) (Figs 3–48). Together these characters are used to quantify the morphological diversity of the majority of known denticles. The alphanumeric naming of character traits allows for additional character traits to be added to the scheme in a flexible manner, to accommodate novel denticle morphologies as they appear. For example, a new character trait for overall denticle shape could become Character Trait A3 and be grouped effectively within that category without significant disruption to the remainder of the system. Throughout, **character traits** and **character states are in bold**. Discussion of how the character states are handled by the disparity calculation are *italicized*, as are any notes about individual character states. Disparity matrices and example photographs for each character and state are included in the supplemental “Appendix 1: Denticle Character State Examples” document, as well as in the R package.

**Fig. 2:**
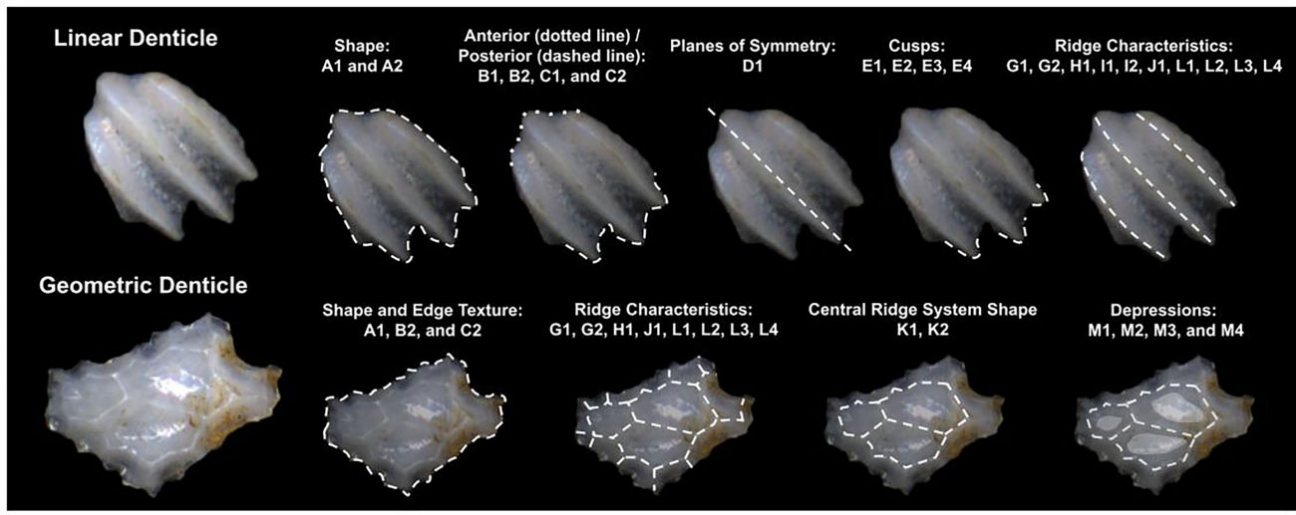
Differences in traits for geometric and linear denticles illustrated on fossil denticles of unknown taxa from Deep Sea Drilling Project (DSDP) site 596, approximately Early Eocene in age.

The most defining character that denticles have is the organization of ridges (or lack thereof) on the crown. We define six broad categories based on the ridge system character trait which all denticle morphotypes in the present code fall in; smooth (no ridges), linear (can tell directionality-anterior/posterior, ridges only curve in a maximum of one way and the ridges themselves are straight/not wavy), geometric (with a central branching pattern), meandering (with ridges that curve or intersect in more than one direction and may appear shaky), spines (crown and ridges are oriented vertically from the skin), and branching (with ridges, which have clear directionality but which curve or intersect in more than one way). While we have aimed to have all characters be relevant to as many denticles as possible, some character traits are applicable only to denticles with specific ridge systems. For example, linear denticles do not have dimples or central ridge system shapes, as those features are characteristic to geometric denticles. Further, geometric denticles rarely have an identifiable directionality (anterior/posterior) (Fig. 2). Resources for coding denticles, including an extended table with example images (Appendix 1) and a two-page compilation of all line-drawings below (Fig. 49 and Appendix 2) are provided as supplemental materials to facilitate coding. We have found that while the extended table provides additional detail and specific examples of possible character states, these specific examples may not be effectively generalized based on individual denticle images, and we encourage use of the written descriptions presented here alongside the generalized line drawings and image-based examples when applying the code.

### General crown shape (A)

#### **Trait A1: Overall shape of crown** (Fig. 3)

Shape is defined as the overall outline shape of the crown, ignoring any topographical features (e.g., ridges, troughs, etc.). There are many additional potential generic denticle shapes; however, we have limited this trait to shapes present in our currently comprehensive dataset. Please see the section about adding to the code for incorporation of additional shapes.

A1.1:
Spine—*Taller than it is wide and stands perpendicular to the skin when attached to the body.*
A1.2:
Cruciform—*“Kite-shaped,” or cross-like (✝) with two transverse ridges originating from the lateral opposing vertices.*
A1.3:
Circular or Oval—*No vertices, curved outline.*
A1.4:
Spade—*Any denticle with a symmetric anterior made by two diverging edges of the same relative length which then converge at a perpendicular straight, curved, or pointed posterior edge.*
A1.5:
Diamond/Rectangle—*A parallelogram, rhombus or equal cross [+], can be asymmetrical.*
A1.6:
Elongated Asymmetrical—*Boot shaped, asymmetric.*
A1.7:
Irregular/other—*Does not fit into any other shape category.*
A1.8:
Fan-like—*Wider than long with only one vertex.*
A1.9:
Triangular or Arrow-like—*Outline diverges from the anterior vertex and converges at the apex, concave on either side of at least one of these vertices, and the length of the denticle is equal to or larger than its width.*

**Fig. 3:**
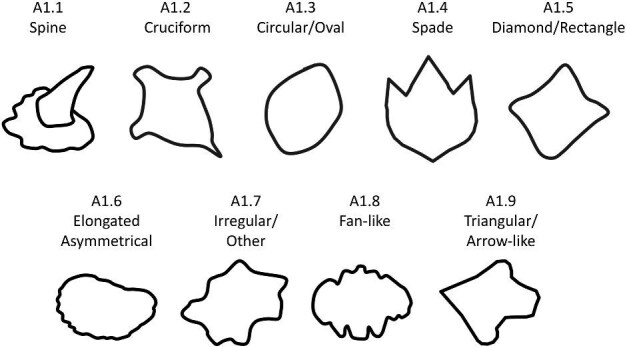
Generalized line drawing examples of character states for Trait A1.

Trait A1 Character Disparity Description: Spines are coded as very different from other denticle shapes because they erupt from the skin semi-vertically and their base is attached with the rest of the crown further from the skin rather than laying horizontally over the skin. The cruciform shape has an anterior and posterior which are not necessarily identifiable but are distinct from the side edges of the denticle. Cruciforms are angular and generally associated with depressions/mounds; they are coded as more different from denticles with rounder shapes and denticles which have different shapes of their anterior and posterior. Circular shaped denticles are also coded as more different from other more angular shapes as they do not have identifiable or distinct anterior/posterior. Weight in disparity calculation: 2.

#### **Trait A2: Spade subtype** (Fig. 4)

The “spade” category allows for further description and classification of denticles, which have an identifiable anterior and posterior (i.e., directionality) and an anterior which is generally symmetrical. If a denticle falls into the generic “spade” category, there are a number of sub-shapes that it can be, based on the shape and structure of the posterior part of the denticle.

A2.0:
Not a Spade.
A2.1:
Rounded Spade—*Lateral edges are curved and the angle between these edges and the anterior vertex is obtuse. Any edges and cusps are also rounded*.
A2.2:
Squared Spade—*The posterior edge is parallel to the anterior vertex and is flat and/or the lateral edges which connect the anterior and posterior are parallel to each other and relatively flat/straight.*
A2.3:
Pointed Spade—*The posterior edge is composed of cusp(s) or point(s)*.
A2.4:
Stretched Spade—*The crown has both an anterior and posterior vertex and the posterior has no more than one cusp or has any number of cusps which extend no further than ¼ the length of the crown.*
A2.5:
Lobed Spade—*Large rounded ridges define the entire crown including rounded lateral edges and a scalloped posterior edge.*
A2.6:
Irregular—*Meet the stipulation of at least one single anterior or posterior vertex but do not fall into any of the aforementioned and commonly seen groupings.*

**Fig. 4:**
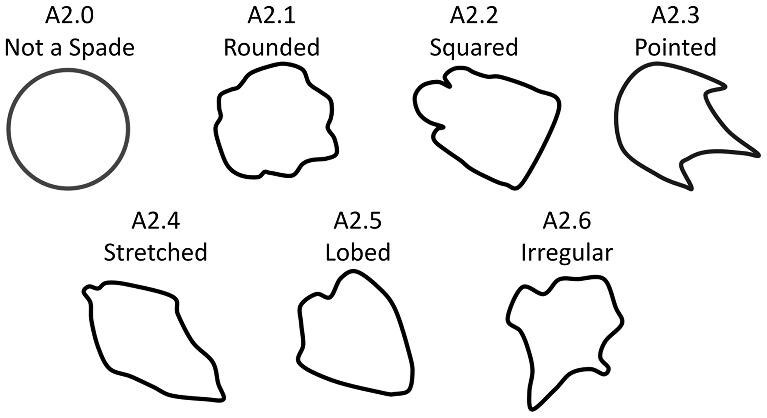
Generalized line drawing examples of character states for Trait A2.

Trait A2 Character Disparity Description: Non-spade shaped denticles are coded as very different from spades. Rounded spades are coded as different from most other spade shapes as they have a greater width than length and round posterior shape. The lobed spade also has round edges and is thus marked as more similar to the rounded spade. The pointed spade shape has both a vertex shaped anterior and a pointed posterior. This makes it different compared to the rounded and squared spades but more similar to other denticles with a pointed edge texture like the stretched spade. The heart-shaped spade has 3 rounded portions with a vertex anterior; its rounded edges make it similar to the rounded and lobed spade but different from the more pointed/jagged spades. Weight in disparity calculation: 0.5.

### Anterior/base (B) and posterior/tip (C) characteristics

#### **Trait B1: Anterior/base shape** (Fig. 5)

The anterior of the denticle (can also be described as the base of the crown) is defined as the area closer to the anterior (head) of the shark. The anterior cannot always be identified, either due to the denticle being partially broken, obscured from vision, or lacking clear directionality. When a disarticulated denticle is coded, it is often possible to assume an anterior/posterior shape based on patterns common amongst linear denticles (linear denticles often have triangular ridges which thin toward the posterior, the troughs, or areas between denticles, generally get wider towards the posterior, and anterior edges are often rounded, while posterior edges are generally pointed). For denticles, which do not have an obvious discernable anterior (base) or posterior (tip) (e.g., many geometric denticles), character state 4 should be used. Denticles that have distinct directionality but are not fully preserved along the anterior edge should be coded as a 0.

B1.0:
Anterior of denticle is too broken to code.
B1.1:
Straight—*Is a relatively flat edge.*
B1.2:
Rounded—*A rounded edge with no vertices.*
B1.3:
Pointed (vertex)—*A vertex (or vertices) compose(s) the anterior of the crown.*
B1.4:
Denticle Lacks Directionality/Cannot discern anterior.

**Fig. 5:**
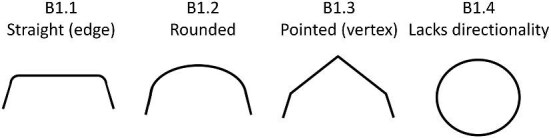
Generalized line drawing examples of character states for Trait B1.

Trait B1 Character Disparity Description: Denticles with an anterior shape that is not identifiable due to a lack of directionality are coded as very different from other anterior shapes. All other anterior shape trait states differ equally from each other with each trait differing one step from each other trait. Weight in disparity calculation: 1.

#### **Trait B2: Anterior marginal macro texture description** (Fig. 6)

*B2 can also describe overall marginal texture if unable to discern distinct anterior/posterior areas.*

The anterior marginal macro texture describes the appearance of the border of a denticle along the anterior of the crown. For denticles with a surrounding mound, code only the upper portion of the crown, and code the mound in Trait O10. For denticles, which do not have an obvious anterior (base)/posterior (tip), but which are not broken (e.g., most geometric denticles), they still have an edge texture. In this case, the edge texture for half of the denticle should be coded in trait B2 and the other half should be coded in Trait C2. If there are distinct regions of differing edge textures along the denticle, code Trait B2 with the lower character score and Trait C2 with the higher character score (Note that the character states for B2 and C2 are identical).

B2.0:
Anterior of denticle is too broken to code.
B2.1:
Smooth—*Is composed of flat margins with no or only shallow curves or vertices and with no cusps or projections.*
B2.2:
Scalloped edge texture—*The edge has a round and wavy pattern usually defined by terminating ridges.*
B2.3:
Pointed edge texture—*The edge is defined by cusps or large points which may or may not be associated with ridges.*
B2.4:
Anterior is a vertex—*Can be pointed or somewhat rounded.*

**Fig. 6:**
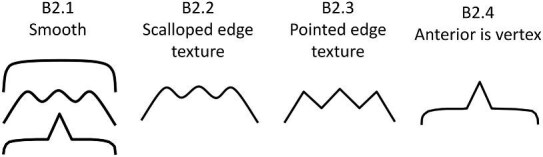
Generalized line drawing examples of character states for Trait B2.

Trait B2 Character Disparity Description: Denticles with a smooth anterior macro marginal texture are coded as very different from all other textures and are coded based on their level of difference from this smooth state. Denticles with scalloped and pointed textures are labeled as significantly different from smooth textures while denticles with an anterior vertex are labeled as equally similar/dissimilar from denticles with smooth or vertex textures. Weight in disparity calculation: 1.

#### **Trait B3: Anterior marginal micro texture description** (Fig. 7)

The marginal micro texture describes the fine-scale texture of the crown's edges independent of marginal shape and the macro texture. Denticles generally have smooth micro texture but some geometric and meandering types may have many jagged points giving the crown a distinctly serrated texture.

B3.1:
Smooth—*The anterior may have cusps or points but the micro texture of the anterior shape is smooth.*
B3.2:
Distinct serrated—*Independent of overall shape or general texture, the anterior micro texture is composed of many jagged points.*

**Fig. 7:**
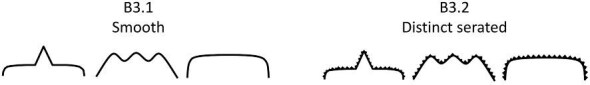
Generalized line drawing examples of character states for Trait B3.

Trait B3 Character Disparity Description: As there are only two trait states identified for this character, they are coded as equally different from each other. Weight in disparity calculation: 1.

#### **Trait C1: Posterior shape** (Fig. 8)

The posterior of the denticle (can also be described as the tip of the crown) is defined as the area closer to the posterior (or caudal fin) of the shark. The posterior cannot always be identified, either due to the denticle being partially broken, obscured from vision, or lacking clear directionality but when a disarticulated denticle is coded, it is often possible to assume an anterior/posterior shape based on patterns common amongst linear denticles (linear denticles often have triangular ridges, which thin toward the posterior or tip of the crown, the troughs, or areas between denticles, generally get wider towards the posterior, and the anterior is often rounded while posterior is generally pointed). For denticles which do not have an obvious discernable anterior (base) or posterior (tip) (e.g., many geometric denticles), character state 4 should be used. Denticles that have distinct directionality but are not fully preserved along the posterior edge should be coded as a 0.

C1.0:
Posterior of denticle is too broken to code.
C1.1:
Straight—*Is a relatively flat edge.*
C1.2:
Rounded—*A rounded edge with no vertices.*
C1.3:
Pointed (vertex)—A *vertex (or vertices) compose(s) the posterior of the crown.*
C1.4:
Denticle Lacks Directionality/Cannot discern posterior.

**Fig. 8:**
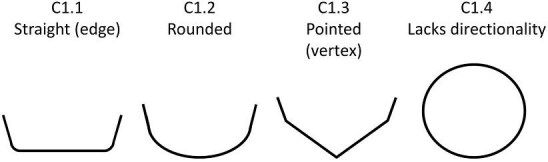
Generalized line drawing examples of character states for Trait C1.

Trait C1 Character Disparity Description: Denticles with a posterior shape that is not identifiable due to a lack of directionality are coded as very different from other posterior shapes. All other posterior shape trait states differ equally from each other with each trait differing one step from each other trait. Weight in disparity calculation: 1.

#### **Trait C2: Posterior marginal macro texture description** (Fig. 9)

*C2 can also describe overall marginal texture if unable to discern distinct anterior/posterior areas.*

Posterior marginal macro texture describes the appearance of the border of a denticle along the posterior of the crown. For denticles with a surrounding mound, code only the upper portion of the crown, and code the mound in Trait O10. For denticles, which do not have an obvious anterior/posterior, but which are not broken (e.g., most geometric denticles), they still have an edge texture. In this case, the edge texture for half of the denticle should be coded in trait B2 and the other half should be coded in Trait C2. If there are distinct regions of differing edge textures along the denticle, code Trait B2 with the lower character score and Trait C2 with the higher character score (Note that the character states for B2 and C2 are identical).

C2.0:
Anterior of denticle is too broken to code.
C2.1:
Smooth—*Is composed of flat margins with no or only shallow curves or vertices and with no cusps or projections.*
C2.2:
Scalloped edge texture—*The edge has a round and wavy pattern usually defined by terminating ridges.*
C2.3:
Pointed edge texture—*The edge is defined by cusps or large points which may or may not be associated with ridges.*
C2.4:
Posterior is a vertex—*Can be pointed or somewhat rounded.*

**Fig. 9:**
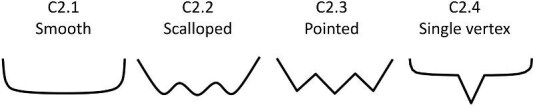
Generalized line drawing examples of character states for Trait C2.

Trait C2 Character Disparity Description: Denticles with a smooth posterior macro marginal texture are coded as very different from all other textures and are coded based on their level of difference from this smooth state. Denticles with scalloped and pointed textures are labeled as significantly different from smooth textures, while denticles with a posterior vertex are labeled as equally similar/dissimilar from denticles with smooth and other edge textures. Weight in disparity calculation: 1.

#### **Trait C3: Posterior marginal micro texture description** (Fig. 10)

The marginal micro texture describes the fine-scale texture of the crowns edges independent of marginal shape and the macro texture. Denticles generally have smooth micro texture but some geometric and meandering types may have many jagged points giving the crown a distinctly serrated texture.

C3.1:
Smooth—*The posterior may have cusps or points but the micro texture of the anterior shape is smooth.*
C3.2:
Distinct serrated—*Independent of overall shape or general texture, the posterior micro texture is composed of many jagged points.*

**Fig. 10:**
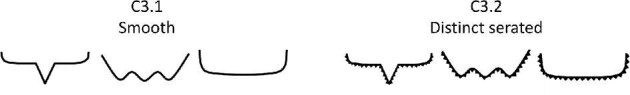
Generalized line drawing examples of character states for Trait C3.

Trait C3 Character Disparity Description: As there are only two trait states identified for this character, they are coded as equally different from each other. Weight in disparity calculation: 1.

### Symmetry (D)

#### **Trait D1: Planes of symmetry** (Fig. 11)

Defined as the number of axes, which can bifurcate the denticle. While small deviations from perfect symmetry (e.g., very slight asymmetries) are common, with such denticles considered “symmetrical,” denticles with one-sided additional ridges, ridge-outgrowths, cusps, etc., should be coded as asymmetrical.

D1.1:
None/Asymmetrical—*No planes of symmetry.*
D1.2:
One—*One plane of symmetry.*
D1.3:
Two—*Two planes of symmetry*.
D1.4:
Three+/Radial—*Three or more planes of symmetry or radial symmetry*.

**Fig. 11:**
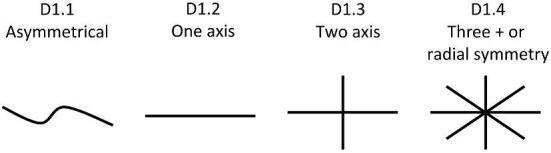
Generalized line drawing examples of character states for Trait D1.

Trait D1 Character Disparity Description: The planes of symmetry trait labels denticles with no planes of symmetry as significantly different from those with planes of symmetry and even more different from denticles with radial symmetry. The trait defines denticles with one and two planes of symmetry as being equally different but denticles with radial symmetry as significantly different from denticles with only one or two planes of symmetry and more different from denticles with no planes of symmetry. Weight in disparity calculation: 1.

### Cusps (E)

#### **Trait E1: Number of cusps** (Fig. 12)

Cusps are defined as extensions from the posterior or tip of the denticle where two vertical edges meet in a vertex between two horizontal posterior edges. Cusps by definition are only present on denticles with an obvious directionality. If the directionality of the denticle cannot be discerned (such as for most geometric denticles), code the denticle with state E1.1 “No Cusps.” Note that the number of cusps does not match the numeric coded character state value.

E1.1:
No cusps.
E1.2:
One cusp.
E1.3:
Two cusps.
E1.4:
Three cusps.
E1.5:
Four cusps.
E1.6:
Five cusps.
E1.7:
Six cusps.
E1.8:
Seven cusps.
E1.9:
Eight cusps.
E1.10:
Nine cusps.
E1.11:
Ten or more cusps.

**Fig. 12:**
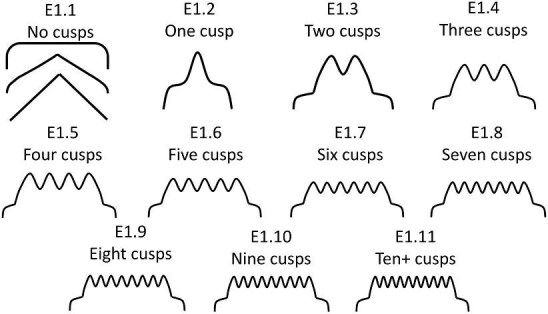
Generalized line drawing examples of character states for Trait E1.

Trait E1 Character Disparity Description: The number of cusps trait has states, which generally differ in equal amounts when cusps are present with increasing numbers of cusps differing in one step increments from each other. Denticles with no cusps are coded as significantly different from those with cusps and denticles with only one cusp are labeled as more different from multi-cusped denticles. Weight in disparity calculation: 1.

#### **Trait E2: Cusp-ridge association** (Fig. 13)

Cusp association describes whether cusps are associated with or independent from ridges. Most denticles with cusps have ridges that run the length of the crown terminating at the tips of the cusps.

E2.0:
No cusps—*The posterior edge of the crown does not have any cusps.*
E2.1:
Cusps are not associated with ridges and/or ridges are not associated with cusps—*Cusps are present but there are either no ridges or ridges which do not run down the center of the cusp.*
E2.2:
Cusps are associated with ridges—*Each cusp has a ridge which runs along its length, usually down the center of the cusp.*
E2.3:
Some cusps are associated with ridges—*There are both cusps and ridges present but only some cusps have ridges which run their length.*

**Fig. 13:**
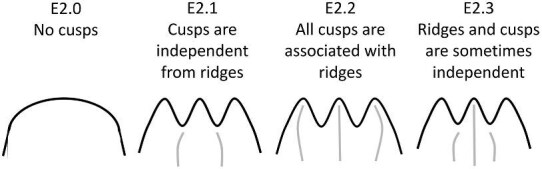
Generalized line drawing examples of character states for Trait E2.

Trait E2 Character Disparity Description: Denticles with cusps are coded as very different from denticles that have no cusps. Denticles with ridges associated with cusps and denticles with cusps not associated with ridges are coded as somewhat different and denticles with some cusps associated with ridges are equally similar/dissimilar to other denticles with cusps. Weight in disparity calculation: 0.5.

#### **Trait E3: Cusps similarity** (Fig. 14)

The cusp similarity trait is a comparison between cusps length, width, and shape. It is only applicable for denticles with more than one cusp to compare between.

E3.0:
No cusps/only one cusp.
E3.1:
Similar cusps—*Cusps are the same length, width, and shape*.
E3.2:
Only the central cusp is distinct—*Meaning it is either a different size, or a different shape than side cusps. Central cusps are often longer and wider and more triangular while side cusps are often shorter and slightly curved.*
E3.3:
Cusps opposite each other are similar, cusps next to each other are distinct—*If a symmetrical crown has more than three cusps, and all cusps on a side are distinct from each other, rather than the denticle having only a distinct central cusp. Note that often the central cusp is longer and wider and the cusps on either side of the central cusp are the same size and slightly shorter, decreasing in size symmetrically closer to the edges.*
E3.4:
Irregular—*All cusps are distinct from each other.*

**Fig. 14:**
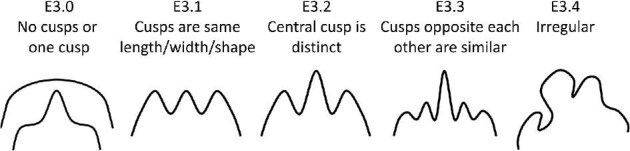
Generalized line drawing examples of character states for Trait E3.

Trait E3 Character Disparity Description: Denticles with no cusps or only one cusp are coded as very different from denticles with multiple cusps. Denticles with cusps that are all similar, that have a distinct central cusp and that have some distinct and some similar cusps are all coded as somewhat different and equally different from each other. Denticles with irregular cusp similarities are coded as very different. Weight in disparity calculation: 0.5.

#### **Trait E4: Relative maximum cusp length** (Fig. 15)

The relative maximum cusp length is a ratio of the longest cusp length to total crown length; crown length is measured from anterior of the crown to the posterior point of the longest cusp.

E4.0:
No cusps.
E4.1:
Cusp length < ¼ crown—*Cusp length is less than ¼ of the total crown length.*
E4.2:
¼ Crown < cusp length < ½ crown—*Cusp length is more than ¼ of the total crown length but less than ½ of the total crown length.*
E4.3:
Cusp length > ½ crown—*Cusp length is more than ½ of the total crown length.*

**Fig. 15:**
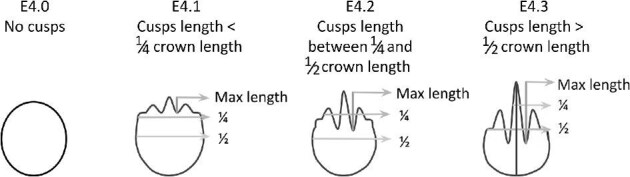
Generalized line drawing examples of character states for Trait E4.

Trait E4 Character Disparity Description: Denticles with no cusps are coded as very different from denticles with cusps. Denticles with cusps are coded so that denticles with increased relative cusp length differ in one step increments from the previous length bin. Weight in disparity calculation: 0.5.

### Overall ridge characteristics (F, G, and H)

#### **Trait F1: Ridge system** (Fig. 16)

The ridge system is a broad categorization for ridge character traits and which primarily accounts for crown directionality (or lack thereof) by taking into account ridge orientation and ridge shape. Linear denticles have clear directionality, meaning all of their ridges run in the same direction. Crowns, which have a geometric ridge system, have ridges with a central branching pattern without a clear directionality. Note that in the case that a crown has an overall shape of spine (trait A1, state 1) but is smooth, it should be coded as having a smooth ridge system.

F1.1:
Smooth—*Smooth denticles have no ridges and thus no ridge system.*
F1.2:
Linear—*Ridges give the crown a clear directionality and anterior/posterior and the ridges can only curve slightly in a maximum of 1 direction.*
F1.3:
Geometric—*Ridges have a central branching pattern usually around a central ridge system shape and a dimple. The crown frequently does not have clear directionality.*
F1.4:
Meandering—*Not all denticles with meandering ridges are categorized as having a meandering ridge system. Crowns with a meandering ridge system are categorized as such due to a somewhat random display of meandering ridges with no other discernible ridge system pattern or directionality.*
F1.5:
Spine—*Spines may or may not have ridges but when they do, these ridges converge at a higher vertical point.*
F1.6:
Branching—*Ridges intersect and branch with a clear directionality meaning each subsequent branching ridge has a predictable direction often on either side of a central ridge.*

**Fig. 16:**
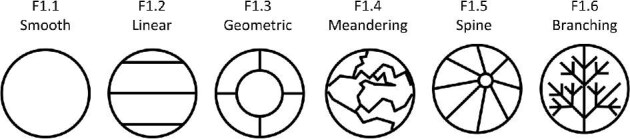
Generalized line drawing examples of character states for Trait F1.

Trait F1 Character Disparity Description: Denticles with branching, geometric, and meandering ridge systems are coded as more similar to each other due to their irregular/unidirectional ridges and more different to denticles that have a spine, smooth, or linear ridge system. Spines and linear denticles are equally dissimilar to each other but more similar to each other than to branching, geometric, meandering, and smooth denticles. Smooth denticles are dissimilar to all other ridge systems as they are the only denticles with no ridges. Weight in disparity calculation: 2.

#### **Trait G1: Number of ridge segments** (Fig. 17)

Ridge segments are enumerated by counting the number of ridges on the crown of a denticle. In the case of linear denticles, these are usually parallel or pseudo-parallel. Where linear ridges branch, the segments are counted as individual ridge segments. For geometric denticles, whenever there is a vertex or pointed change in direction of a ridge, that is considered a separate “segment.” In the case that a denticle has troughs, or long narrow valleys between ridges and other vertically higher crown traits, in addition to ridges, the trough is not counted in ridge number. In the case that a denticle has many troughs but no ridges the troughs are counted and described as ridges. Note that “ridge outgrowths” (see Trait G3) are not counted in the number of ridges here. Note that the number of ridge segments does not match the numeric coded character state value.

G1.1:
None—*The denticle is smooth.*
G1.2:
One.
G1.3:
Two.
G1.4:
Three.
G1.5:
Four.
G1.6:
Five.
G1.7:
Six-ten.
G1.8:
Eleven or more.

**Fig. 17:**
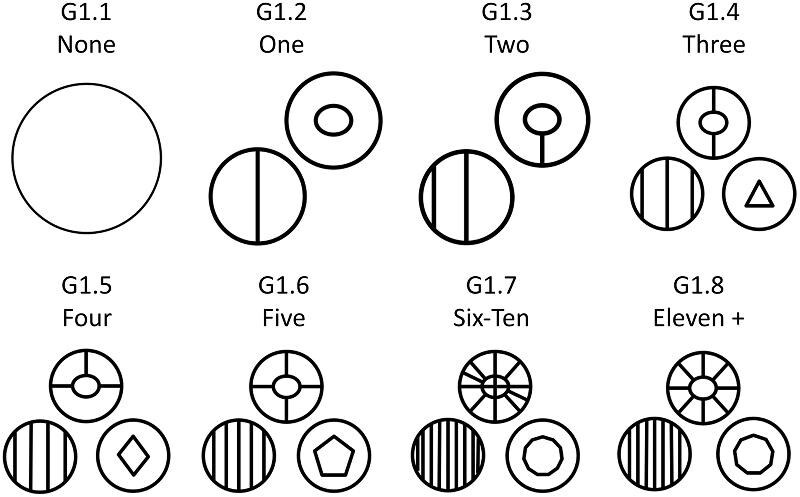
Generalized line drawing examples of character states for Trait G1.

Trait G1 Character Disparity Description: Denticles with no ridge segments are very dissimilar to denticles with any ridge segments. With denticles with one through five segments are different linearly with denticles with more ridge segments being more different in proportionate steps to denticles with less segments. Denticles with six or more denticles are coded as more similar in their difference to other denticles with less segments as many types with this many ridges occasionally differ in number of segments by a few. Weight in disparity calculation: 1.

#### **Trait G2: Number of independent ridges** (Fig. 18)

Independent ridges are defined as ridges that do not intersect with any other ridges or curve in more than one direction. Where ridge segments diverge, branch, or change direction but are still connected, they are counted as one independent ridge. For geometric denticles, whenever there is a vertex or pointed change in direction of a ridge, it is only considered one independent ridge. In the case that a denticle has troughs, or long narrow channel segments in addition to ridges, the trough(s) is not counted in independent ridge number. In the case that a denticle has many troughs but no ridges the troughs are counted and described as ridges with each separate branch of troughs counted as an independent “ridge.” Note that the number of independent ridges does not match the numeric coded character state value.

G2.1:
None—*The denticle is smooth.*
G2.2:
One.
G2.3:
Two.
G2.4:
Three.
G2.5:
Four.
G2.6:
Five.
G2.7:
Six or more.

**Fig. 18:**
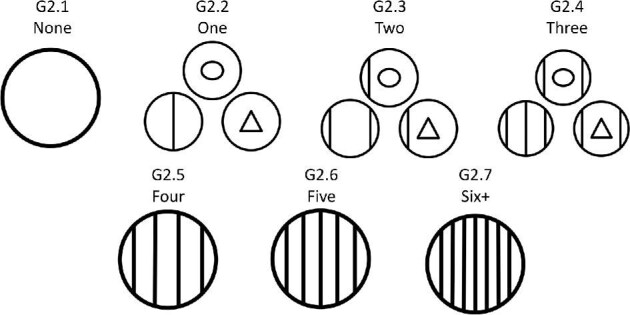
Generalized line drawing examples of character states for Trait G2.

Trait G2 Character Disparity Description: Denticles with no ridges are coded as very different from denticles with any independent ridge segments. Denticles with only one independent ridge segment are coded as different from denticles with more than one independent ridge, this is because denticles with only one independent ridge include denticles with many intersecting/branching ridge segments. Denticles with two, three, four, and five ridges are coded as equally dissimilar while denticles with six or more independent ridges are coded as more different. Weight in disparity calculation: 1.

#### **Trait G3: Ridge outgrowths** (Fig. 19)

Ridge outgrowths are defined as small ridge-like extensions on the anterior edges of some linear denticles. These outgrowths appear distinct from the central main crown in shape and length. Ridge outgrowths are not counted in the total number of ridges or number of independent ridges on a denticle (trait G1 and G2). Note that the number of ridge outgrowths does not match the numeric coded character state value.

G3.0:
No ridges (smooth) or broken—*No ridges or too broken to code.*
G3.1:
No ridge outgrowths.
G3.2:
One ridge outgrowth—*One ridge outgrowth on one lateral edge of the main section of the crown.*
G3.3:
Two ridge outgrowths—*Two ridge outgrowths on either lateral edge of the main section of the crown.*

**Fig. 19:**
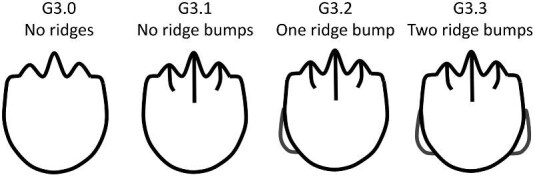
Generalized line drawing examples of character states for Trait G3.

Trait G3 Character Disparity Description: Denticles with no ridges are coded as more different from denticles with ridges and ridge outgrowths. Denticles with no ridge outgrowths are coded as more different than denticles with one or two ridge outgrowths and denticles with one or two ridge outgrowths are coded as less dissimilar to each other. Weight in disparity calculation: 1.

#### **Trait H1: Ridge orientation** (Fig. 20)

Ridges with a linear system can be further characterized by their ridges’ orientation to one another, as diverging or converging from the anterior, branching, or truly parallel, while geometric denticles always have a branching pattern and spine denticles always have radial ridges when ridges are present. For geometric denticles, use character state 5, ridges intersect/branch unless there is an obvious better fit.

H1.0:
Only one Ridge/No Ridges.
H1.1:
Parallel—*All ridges run parallel to each other. Many linear denticles may appear to have slight divergence from the anterior but are parallel along the majority of the crown. These denticles should be coded with a parallel ridge orientation.*
H1.2:
Converges from anterior—*Ridges converge from the anterior towards the posterior.*
H1.3:
Diverges from anterior—*Ridges diverge from the anterior towards the posterior.*
H1.4:
Diverges then converges—*Ridges diverge and then converge from the anterior towards the posterior.*
H1.5:
Ridges intesect/branch—*This includes denticles with geometric, branching, and some meandering ridge systems.*
H1.6:
Apex Radial—*Often spine*s.
H1.7:
Irregular/no discernable anterior/posterior or visible pattern.
H1.8:
Multiple ridge orientations.

**Fig. 20:**
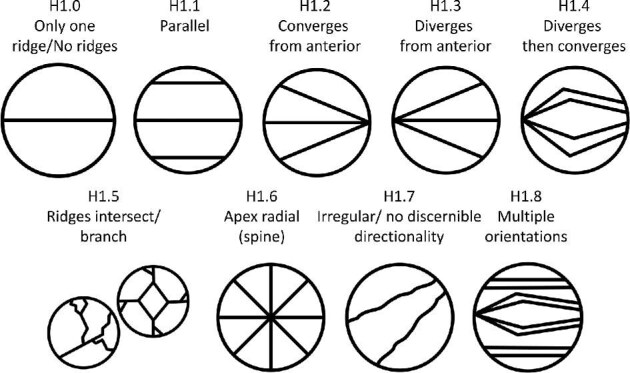
Generalized line drawing examples of character states for Trait H1.

Trait H1 Character Disparity Description: Denticles with only one or no ridges are coded as very dissimilar to all other denticles. Denticles with parallel, or converging/diverging from base ridge orientations are coded as more similar to each other than other ridge orientations including denticles, which have ridges that diverge and then converge. Denticles with apex/radial or intersecting or branching ridge orientations are coded as distinct but more similar than other orientations and very dissimilar to other ridge orientations. Weight in disparity calculation: 0.5.

### Central (I) and non-central (J) ridges

#### **Trait I1: Central ridges or trough disparity** (Fig. 21)

The central ridge is defined as the ridge (or ridges for crowns with a central trough) in the center of all other ridges on the crown, or in the case that the crown has only one ridge, may be the ridge that is at the center of the crown. The central ridge usually runs from the anterior to the posterior bifurcating the crown. It is sometimes, though not always, distinct from the other ridges in shape and size.

I1.0:
None—*No ridges.*
I1.1:
No central ridge—*Ridges present on the crown but there is either an even number of ridges with no central ridge or a ridge orientation without a central ridge (i.e., many geometric denticles have a central ridge system shape (trait K1) but may not have a singular central ridge).*
I1.2:
Only one ridge—*When a denticle has only one ridge, it often runs down the center of the crown from anterior to posterior.*
I1.3:
Same as other ridges—*The central ridge is the same shape and size as the other ridges.*
I1.4:
Distinct from other ridges—*The central ridge is a different shape and/or size than the other ridges.*

**Fig. 21:**
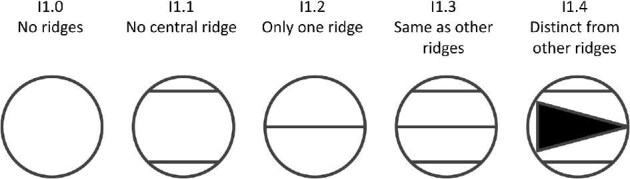
Generalized line drawing examples of character states for Trait I1.

Trait I1 Character Disparity Description: Denticles with no ridges are coded as very different from other denticles. Denticles with no central ridge are coded as more similar to denticles with only one ridge and very different from denticles with a central ridge that is the same as other ridges or distinct. Denticles with only one ridge are coded as more similar to denticles with central ridges (both that are similar ridges and distinct central ridges) because often denticles with only one ridge are in the center of the crown (though not in the center of other ridges). Denticles with central ridges that are distinct are coded as different from denticles with similar central ridges but less distinct than these central ridged denticles from other orientations with no central ridges. Weight in disparity calculation: 0.5.

#### **Trait I2: Central ridge directionality** (Fig. 22)

Central Ridge directionality is defined by the edges of the central ridge (or ridges for crowns with a central trough), independent of ridge orientation or the central ridge relative to other ridges.

I2.0:
None—*There are no ridges or there is no central ridge.*
I2.1:
Straight—*The central ridge runs in a single direction, usually anterior to posterior and has parallel sides.*
I2.2:
Curved—*The central ridge curves, changing direction at a single inflection point.*
I2.3:
Meandering—*An irregularly shaped ridge with uneven and/or inconsistent edges which may have a general direction but curves or deviates direction slightly along its length.*

**Fig. 22:**
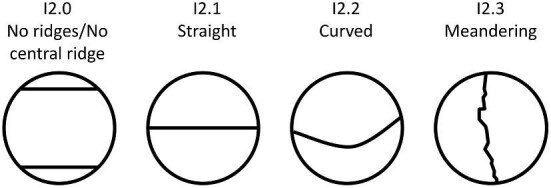
Generalized line drawing examples of character states for Trait I2.

Trait I2 Character Disparity Description: Denticles with straight and curved are coded as being more similar to each other than denticles with meandering central ridge directionalities. Weight in disparity calculation: 0.5.

#### Trait I3: Central ridge width (Fig. 23)

Width is defined by the edges of the independent ridge(s) relative to the anterior of the crown.

I3.0:
None—*No central ridge.*
I3.1:
Parallel—*Consistent width along length.*
I3.2:
Triangular widening from anterior—*The central ridge is triangular with an anterior vertex which widens to a flat edge at the posterior.*
I3.3:
Triangular thinning from anterior—*The central ridge is triangular with an anterior flat edge which thins to a posterior vertex.*
I3.4:
Diamond-like—*Widening and then thinning from anterior.*
I3.5:
Creates a Central Trough—*Two central ridges converge at the anterior and/or posterior of the crown creating a long narrow valley in between.*
I3.6:
Irregular.

**Fig. 23:**
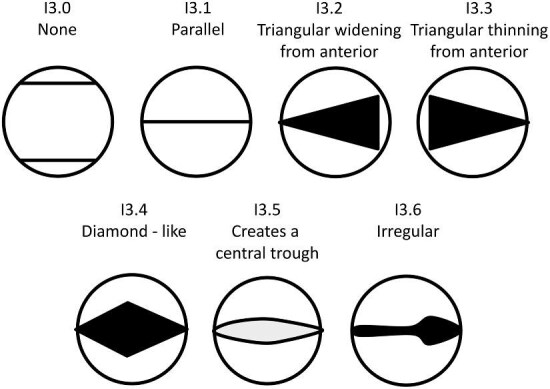
Generalized line drawing examples of character states for Trait I3.

Trait I3 Character Disparity Description: Denticles with a straight central ridge are coded as different from all other central ridge widths based on their level of difference from this straight state. Triangular and diamond shaped central ridges are coded as equally similar/dissimilar from straight ridges, while troughs and irregular widths are coded as the most dissimilar from straight ridges. Weight in disparity calculation: 0.5.

#### **Trait J1: Non-central ridge(s) directionality** (Fig. 24)

Non-central ridge directionality is defined by the edges of the non-central ridge, independent of ridge orientation or the non-central ridge relative to any central ridge. Many denticles with a geometric ridge system have a combination of curved ridges and straight ridges.

J1.0:
None/only central ridge present—*There are no ridges or there is only a central ridge.*
J1.1:
Straight—*Each ridge segment runs in a single direction.*
J1.2:
Concave Curved—*Ridges are curved with a concave angle relative to the center of the crown.*
J1.3:
Convex Curved—*Ridges are curved with a convex angle relative to the center of the crown.*
J1.4:
Meandering—*Irregularly shaped ridges with uneven and/or inconsistent edges which have a general direction but which curve or deviate in direction slightly along their length.*
J1.5:
Combination—*Multiple or irregular shapes.*

**Fig. 24:**
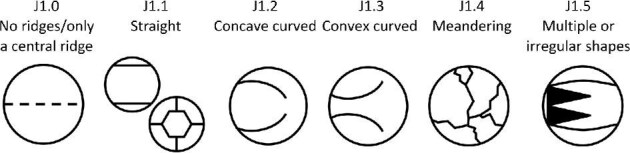
Generalized line drawing examples of character states for Trait J1.

Trait J1 Character Disparity Description: Denticles with straight non central ridges are coded as equally different from both curved directionalities, which are more similar to each other. Denticles with meandering or combinations of non-central ridge directionalities are coded as the most distinct from other ridge directionalities but more similar to each other. Weight in disparity calculation: 0.5.

#### **Trait J2: Non-central ridge(s) width** (Fig. 25)

Width is defined by the edges of the independent ridge(s) relative to the anterior of the crown.

J2.0:
None/only central ridge present—*No ridges or only a central ridge.*
J2.1:
Parallel—*Consistent width along length.*
J2.2:
Triangular widening from anterior—*The non-central ridges are triangular with an anterior vertex which widens to a flat edge at the posterior.*
J2.3:
Triangular thinning from anterior—*The central ridge is triangular with an anterior flat edge which thins to a posterior vertex.*
J2.4:
Diamond-like—*Widening and then thinning from the anterior.*
J2.5:
Create Troughs—*In pairs, non-central ridges converge at the anterior and/or posterior of the crown creating a long narrow valley in between.*
J2.6:
Irregular/Combination.

**Fig. 25:**
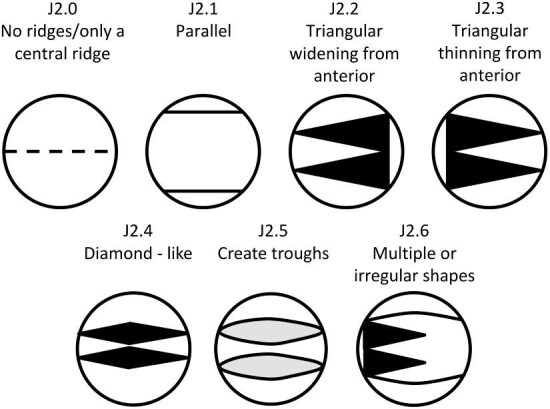
Generalized line drawing examples of character states for Trait J2.

Trait J2 Character Disparity Description: Denticles with straight non central ridges are coded as different from all other ridge widths based on their level of difference from this straight state. Triangular and diamond shaped non central ridges are coded as equally similar/dissimilar from straight ridges while troughs and irregular or combination widths are coded as the most dissimilar from straight ridges and equally similar/dissimilar to each other. Weight in disparity calculation: 0.5.

### Central ridge system (K)

#### **Trait K1: Central ridge system shape** (Fig. 26)

Denticles with a geometric ridge system often have a central branching ridge orientation with ridges at the center, which define the edges of this enclosed polygon or rounded shape, called the central ridge system shape. Other ridges generally branch and radiate from the central ridge system shape's edges.

K1.0:
No Ridges.
K1.1:
Ridges present but no central shape observed—*for example, linear denticles fit this description.*
K1.2:
Circular/oval—*Central shape is round and curved with no vertices or straight edges.*
K1.3:
Triangular—*The central shape has three edges which converge at three vertices.*
K1.4:
Quadrilateral—*The central shape has four edges which converge at four vertices.*
K1.5:
Pentagonal—*The central shape has five edges which converge at five vertices*.
K1.6:
Hexagon—*The central shape has six edges which converge at six vertices.*
K1.7:
Heptagon—*The central shape has seven edges which converge at seven vertices.*
K1.8:
Mound—*The central shape has any number of edges which converge at a central mound, or plateau, or a raised nub.*
K1.9:
Octagon—*The central shape has eight edges which converge at eight vertices.*
K1.10:
Multiple—*Multiple central ridge system shapes.*
K1.11:
Irregular—*Shape is considerably irregular and does not fall into other categories.*

**Fig. 26:**
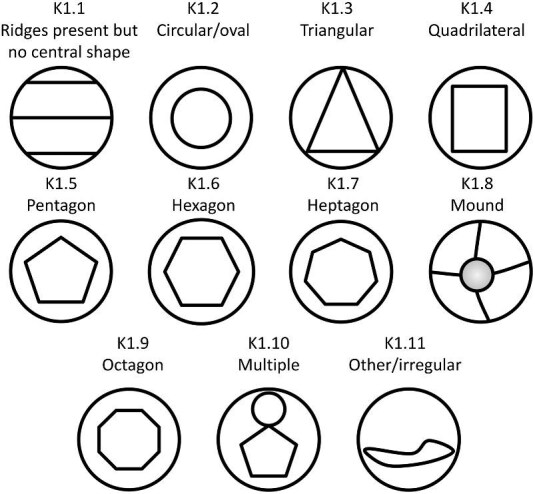
Generalized line drawing examples of character states for Trait K1.

Trait K1 Character Disparity Description: Denticles with a circular/oval, triangular, quadrilateral, pentagonal, hexagonal, heptagonal, and octagonal central ridge system shape are all composed of enclosed geometric depressions with a dimple in the center and are thus coded as fairly similar in comparison to a mound which has a significantly different composition which is not necessarily associated with a geometric shape or dimple. Additionally, denticles with ridges with an observed central ridge system shape are coded as very different from denticles with ridges that do not have any central shape. Denticles with no ridges are likewise coded as equally different from all other ridged denticles and denticles with multiple central shapes observed are also coded as significantly different from other denticles with a single central ridge system shape. Weight in disparity calculation: 1.

#### **Trait K2: Central ridge system shape planes of symmetry** (Fig. 27)

The central ridge system shape planes of symmetry is defined as the number of axes, which can bifurcate the central ridge system shape.

K2.0:
No central ridge system shape.
K2.1:
No symmetry—*Central shape present but has no planes symmetry.*
K2.2:
One—*Central shape has one plane of symmetry.*
K2.3:
Two—*Central shape has two planes of symmetry.*
K2.4:
Radial—*Central shape has radial symmetry.*

**Fig. 27:**
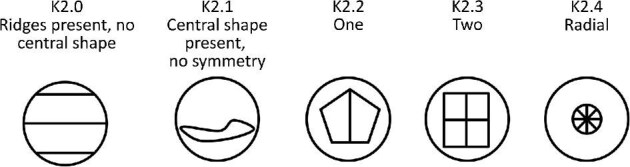
Generalized line drawing examples of character states for Trait K2.

Trait K2 Character Disparity Description: Denticles with no central shape are coded as very different from denticles with a central ridge system shape. Denticles with a central ridge system shape with no planes of symmetry are coded as significantly different from those with planes of symmetry and even more different from denticles with central ridge system shapes with radial symmetry. Denticles with central ridge system shapes with one and two planes of symmetry are coded as being less different from each other and less different to those with radial symmetry than those with no central shape or those with no planes of symmetry. Weight in disparity calculation: 0.5.

### Ridge size (L)

#### **Trait L1: Ridge length** (Fig. 28)

The lengths of ridges vary in relation to the crown length and compared to other ridges. Linear denticles typically have ridges, which stretch from the anterior to the posterior edges of the crown, while geometric denticles with exclusively branching ridges tend to include ridges, which do not reach the edges of the crown and rather begin and terminate at different areas on the crown.

L1.1:
No ridges.
L1.2:
Ridge length = lateral length—*The ridge(s) run from the anterior edge to the posterior edge of the crown. This does not mean that all ridges extend the longest axis of the crown, rather that the ridge runs from edge to edge (even if the side ridges and edges are shorter than the center of the crown and center ridges).*
L1.3:
Ridge ends mid crown/Ridge begins mid crown—*The ridge(s) begin at the anterior edge but terminate before reaching the posterior edge or begin mid-crown and terminate at the posterior edge.*
L1.4:
Ridge begins and ends mid crown—*The ridge(s) do not reach the posterior or anterior edges.*
L1.5:
Includes ridges described by a combination of 1,2, 3,—*Different ridge(s) have differing lengths relative to the size of the crown and anterior and posterior edges.*

**Fig. 28:**
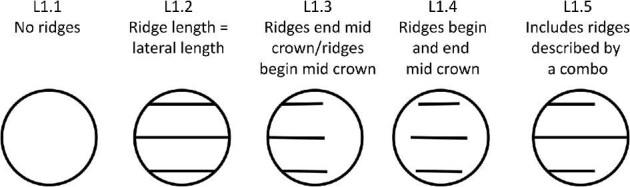
Generalized line drawing examples of character states for Trait L1.

Trait L1 Character Disparity Description: Denticles with no ridges are coded as very distinct from denticles with ridges. Denticles with equal crown length and ridge length, ridges that end or begin mid crown, and denticles with ridges that both end and begin mid crown are all coded as less distinct from each other. Denticles with ridges that have a combination of the previously described ridge length character states are coded as the most dissimilar to all other ridge lengths. Weight in disparity calculation: 0.5.

#### **Trait L2: Ridge definition** (Fig. 29)

Ridges differ in their level of definition or visibility. They can be visible with deep troughs or shallow with nearly invisible ridge patterns. Some denticles display ridges of varying definition. This can also be thought of as smoothness or rugosity of the denticle's crown.

L2.0:
No Ridges.
L2.1:
Not Clearly Defined—*Ridge(s) are shallow and difficult to see.*
L2.2:
Clearly defined on part of the crown but gets shallower and does not extend to full length of denticle—*Usually this occurs when ridge(s) are taller and more defined at the anterior but become shallower and less clearly visible at the posterior.*
L2.3:
Clearly Defined—*Ridges are tall and clearly visible on the entire crown.*
L2.4:
Only the central ridge is clearly defined—*Side ridges are shallow and difficult to see and the central ridge is taller and clearly visible.*

**Fig. 29:**
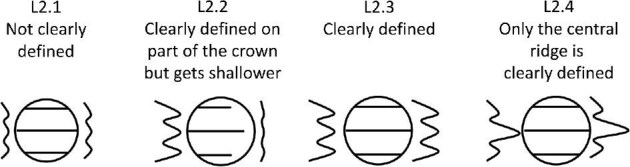
Generalized line drawing examples of character states for Trait L2.

Trait L2 Character Disparity Description: Denticles with ridges are coded as very different from denticles without ridges. Denticles with ridges that are not clearly defined at all are coded as very different from denticles with some clear ridges and even more different to denticles with completely clearly defined denticles. Denticles with only a defined central ridge and denticles with ridges that are clear on part of the crown but become less defined are coded as more similar to each other than the other trait states. Weight in disparity calculation: 0.5.

#### **Trait L3: Relative ridge heights** (Fig. 30)

Heights can differ among the ridges on a denticle, if there is height disparity the tallest ridge is usually towards the center of a denticle with a linear system.

L3.0:
None—*No ridges.*
L3.1:
Only one ridge—*Only one ridge and thus has no relative height compared to other ridges.*
L3.2:
Equal—*All ridges appear to have equal ridge heights.*
L3.3:
Variable—*Ridges have variable heights with some ridges which are taller than others.*

**Fig. 30:**
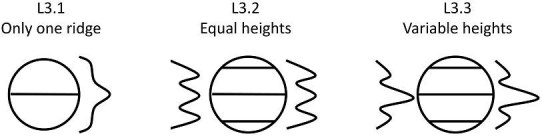
Generalized line drawing examples of character states for Trait L3.

Trait L3 Character Disparity Description: Denticles with no ridges and only one ridge are coded as very different from denticles with multiple ridges. Denticles with equal ridge heights and variable ridge heights are coded as equally different from each other. Weight in disparity calculation: 0.5.

#### Trait L4: Ridge/trough angularity (Fig. 31)

Ridge vertical profiles (or cross sections) can have different shapes, some are rounded, while others are triangular. Some denticles display a range of ridge angularity.

L4.0:
No ridges.
L4.1:
Ridge profiles or troughs are rounded.
L4.2:
Ridge profiles or troughs are triangular.
L4.3:
Ridge profiles or troughs are funnel shaped—*Ridge profiles or troughs are funnel shapes (rounded at the anterior and thin to a skinnier point at the posterior).*
L4.4:
Ridge profiles or troughs are variable or described by another shape.
L4.5:
Ridge profiles or troughs are rectangular.

**Fig. 31:**
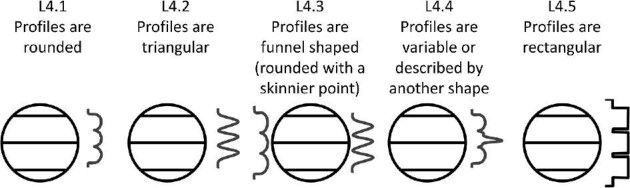
Generalized line drawing examples of character states for Trait L4.

Trait L4 Character Disparity Description: Denticles with no ridges and denticles with ridges or troughs, which have variable ridge profiles are both coded as very different from all other ridge/trough profiles, which are all coded as equally different from each other. Weight in disparity calculation: 0.5.

### Depressions and dimples (M)

Depressions are defined as low areas or impressions on the crown. Dimples are a type of depression, which are clearly outlined by surrounding ridges.

#### **Trait M1: Number of depressions** (Fig. 32)

Number of depressions: Depressions are defined as low areas or impressions on the crown. These occur most often in geometric denticles with ridges that have a central branching pattern.

M1.1:
None.
M1.2:
One.
M1.3:
Two.
M1.4:
Three.
M1.5:
Four.
M1.6:
Five or more.
M1.7:
Multiple depressions (>2) but denticle is broken so unable to discern precise number.

**Fig. 32:**
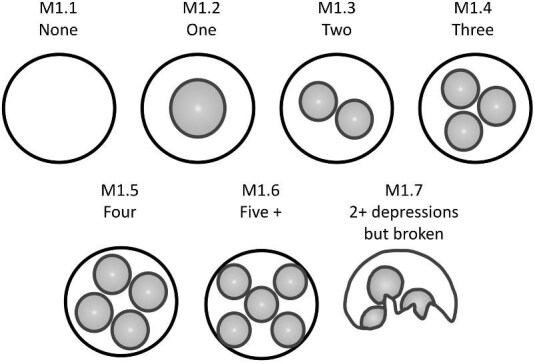
Generalized line drawing examples of character states for Trait M1.

Trait M1 Character Disparity Description: Denticles with no depressions are coded as very different from denticles with depressions. Denticles with depressions are then coded as having linear increases in differences from each increasing number of depressions with denticles with multiple depressions but no exact number being coded as the same level of difference to other character states as denticles with two depressions. Weight in disparity calculation: 1.

#### **Trait M2: Depression(s) type** (Fig. 33)

Depressions can be further divided by their surroundings on the crown. The most common type of depression are dimples, or depressions surrounded by ridges.

M2.0:
No depression—*Ridges may or may not be present but there are no depressions on the crown.*
M2.1:
Thumbprint—*Surface layer impression on the crown without surrounding ridges.*
M2.2:
Open tunnel—*Partially covered impression where the lateral edges of the crown fold over and cover the lateral edges of the depression.*
M2.3:
Dimples—*Depressions in the center of a central ridge system shape (clearly surrounded by ridges).*

**Fig. 33:**
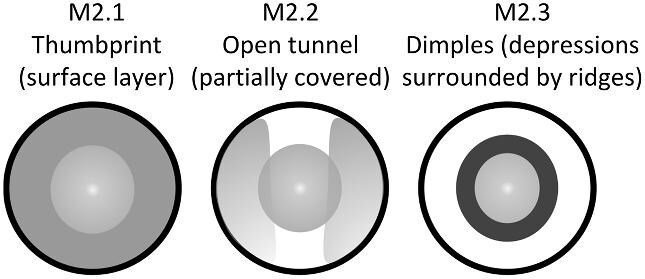
Generalized line drawing examples of character states for Trait M2.

Trait M2 Character Disparity Description: Denticles with no depressions are coded as very different from denticles with depressions. Denticles with thumbprint, open tunnel, or dimple depressions are coded as equally different from each other. Weight in disparity calculation: 0.5.

#### **Trait M3: Location of depression** (Fig. 34)

Depressions can be located in the center, along the edges, or both in the center and along the edges of the crown.

M3.0:
No depression.
M3.1:
Central depression—*Depression does not touch the edges of the crown and is generally centered between the posterior and anterior of the crown.*
M3.2:
Close to edge depression—*Depression may touch an edge of the crown and is closer to the posterior or anterior rather than being in the middle.*
M3.3:
Both central and edge depressions—*Multiple depressions present with some being in the center and others closer to the edges of the crown.*

**Fig. 34:**
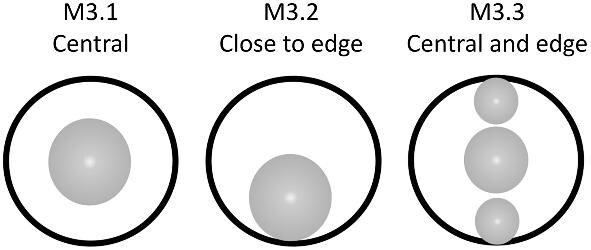
Generalized line drawing examples of character states for Trait M3.

Trait M3 Character Disparity Description: Denticles with no depressions are coded as very different to denticles with depressions. Denticles with depressions that are both close to the edge of the crown and in the center are also coded as very different from denticles with depressions that are only found along the edge of the crown or the center of the crown. Denticles with depressions that are only found along the edge of the crown or the center of the crown are coded as more similar to each other than to other character states in this trait. Weight in disparity calculation: 0.5.

#### **Trait M4: Shape of dimple** (Fig. 35)

The dimple shape describes the outline of the lowest area in a central ridge system shape, as clearly outlined by surrounding ridges. Dimple shapes are often difficult to identify and often mimic the shape of the central ridge system shape. However, there is occasionally a clear dimple shape, which differs from the central shape or mimics the overall crown shape. When a crown has multiple dimples of the same shape, they can be coded by that shape, but if they differ in shapes, they should be coded as “M4.7 multiple with different shapes.”

M4.0:
None—*There are no dimples on the crown.*
M4.1:
Circle—*The dimple(s) are circular.*
M4.2:
Elongated—*The dimple is round and elongated.*
M4.3:
Teardrop (smooth curve)—*The dimple is teardrop shaped with a wide round portion which thins to a vertex.*
M4.4:
Square/quadrilateral—*The dimple is quadrilateral like with four straight edges and four vertices.*
M4.5:
Pentagon—*The dimple is pentagon like with five straight edges and five vertices.*
M4.6:
Irregular—*The dimple does not have a recognizable or definable shape.*
M4.7:
Multiple dimples w/different shapes.

**Fig. 35:**
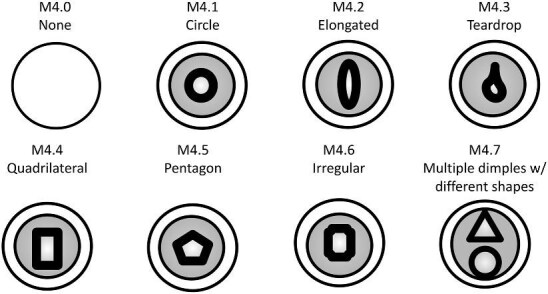
Generalized line drawing examples of character states for Trait M4.

Trait M4 Character Disparity Description: Denticles with dimples are coded as very different from denticles without dimples and different dimple shapes are all coded as equally different from each other. Weight in disparity calculation: 0.5.

### Secondary features and surface textures (N)

#### **Trait N1: Secondary ridge features** (Fig. 36)

Secondary ridge features include micro reliefs (smaller ridges that are present as features on top of larger ridges), surface textures (or shallow thin patterns on top of larger ridges or the crown surface), and vertical protrusions on ridges (in which a small spine-like feature protrudes from a ridge). If these are present, all prior ridge character classifications are done on the larger ridges.

N1.1:
No Ridges.
N1.2:
No secondary ridge features—*Ridges are present but do not have any additional textures or additional ridges on them.*
N1.3:
Micro-reliefs on ridges—*Ridges present and have additional ridge-like features on their surface.*
N1.4:
Honeycomb surface texture—*A thin raised honeycomb or web-like texture covers a portion of the crown and/or ridges.*
N1.5:
Wavy surface texture—*Thin wavy or scalloped texture covers a portion of the crown and/or ridges.*
N1.6:
Honeycomb/wavy combination surface texture—*Both honeycomb and wavy texture are both present on the crown and or ridges.*
N1.7:
Vertical protrusion(s) from ridge(s)—*A vertical nub or bump is present on ridge(s).*

**Fig. 36:**
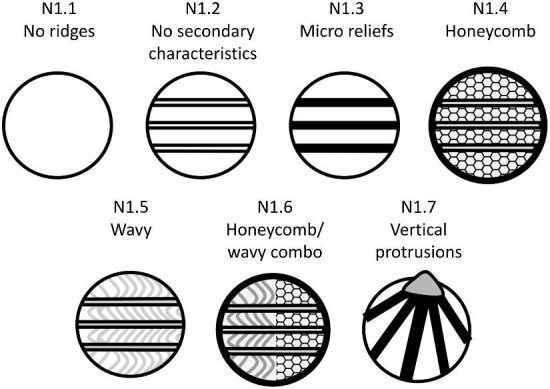
Generalized line drawing examples of character states for Trait N1.

Trait N1 Character Disparity Description: Denticles with no ridges are coded as very distinct from denticles that have ridges. Denticles with no secondary ridge features are then coded as being slightly less different than denticles with no ridges compared to other denticles that do have any type of secondary ridge features. Denticles, which have vertical protrusions from its ridges are coded as the most different from all other secondary ridge feature categories. Denticles with micro-reliefs on ridges are coded as less distinct from other denticles with other secondary ridge feature categories. Denticles coded as having a specified “surface texture” are coded as more similar to each other than to other categories. Weight in disparity calculation: 1.

#### **Trait N2: Surface texture location** (Fig. 37)

Surface texture location describes the part of the crown where there is a non-smooth surface, as described in trait *N1: Secondary Ridge Features* in state 4 (honeycomb), 5 (wavy) or 6 (combination of wavy and honeycomb).

N2.0:
None—*No secondary ridge features.*
N2.1:
Ridges only—*The ridges have a rough surface texture.*
N2.2:
Crown and ridges—*Both the crown and ridges have a rough surface texture.*
N2.3:
Crown except ridges—*The crown (but not the ridges) has a rough surface texture.*
N2.4:
Anterior of crown only—*The surface texture is rough at the anterior of the crown.*
N2.5:
Middle of crown only—*The surface texture is rough at the middle of the crown*
N2.6:
Posterior of crown only—*The surface texture is rough at the posterior of the crown.*
N2.7:
Edges of crown only—*The surface texture is rough at the edges of the crown.*

**Fig. 37:**
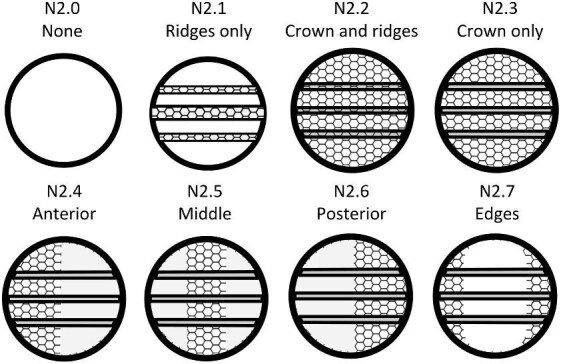
Generalized line drawing examples of character states for Trait N2.

Trait N2 Character Disparity Description: Denticles with a crown that has surface texture only on its ridges are coded as equally different to other surface textured crown except for the crown with surface texture on the entire crown except for the ridges. Denticles with surface texture on the entire crown including on the ridges are coded as more different from the crown with surface texture on only the anterior, middle, posterior, or edges. All other categories of surface texture differ equally. Weight in disparity calculation: 0.5.

#### **Trait N3: Surface texture coverage** (Fig. 38)

Surface texture coverage describes the % of the crown covered by a non-smooth surface texture as described in *N1: Secondary ridge features* in state 4 (honeycomb), 5 (wavy), or 6 (combination of wavy and honeycomb) and in *N2: Surface texture location.*

N3.0:
None—*The surface does not have a specified rough surface texture.*
N3.1:
Less than 30%—*Less than 30% of the crown has a specified rough surface texture.*
N3.2:
Between 30% and 70%—*Between 30% and 70% of the crown has a specified rough surface texture*.
N3.3:
Greater than 70%—*Greater than 70% of the crown has a specified rough surface texture.*

**Fig. 38:**
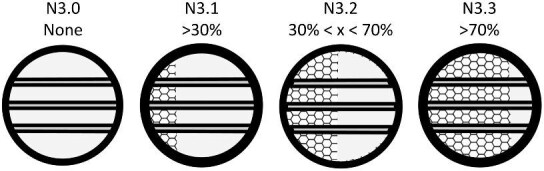
Generalized line drawing examples of character states for Trait N3.

Trait N3 Character Disparity Description: Denticles with surface texture are coded so that crowns with increased coverage differ in one step increments from the previous coverage bin. Weight in disparity calculation: 0.5.

### Base characteristics (O)

The base of the denticle is defined as the portion below the skin. The base is not always present and is not viewable with traditional 2D photographs of the skin surface—they can be imaged using micro-CT scanning and through the careful dissolving of a skin patch via dilute bleach, which preserves the denticle but removes the skin. Bases are rarely preserved in the fossil record.

While the majority of fossil denticles only preserve the crown of the denticle, the base, or subcutaneous portion of the denticle has considerable morphological diversity as well both within and between species, and can provide valuable information in capturing morphological diversity of extant denticles (Vaz et al. 2023). Thus, for the majority of fossil denticles, all base characteristics will be coded as 0 (“too broken to code/base not preserved”); this section is included to fully capture the breadth of known denticle morphological diversity.

#### **Trait O1: Overall base shape** (Fig. 39)

Overall base shape is defined as the overall outline shape of the base, ignoring any topographical features (e.g., grooves, foramen, etc.)

O1.0:
Base not preserved or base not observable.
O1.1:
Kite shaped—*Base is kite-like in shape with straight edges and two shorter lateral vertices, one short posterior or anterior vertex and a much longer anterior or posterior vertex.*
O1.2:
Kite/cruciform shaped with extended vertices—*Base is cruciform in shape with rounded edges and two shorter lateral corners, one shorter posterior or anterior rounded corner and a much longer anterior or posterior corner.*
O1.3:
Kite/cruciform shaped missing 1/4 of the cross ridges/extensions—*Base is cruciform-like in shape with two longer lateral vertices and one shorter anterior or posterior vertices and a short rounded or straight anterior or posterior edge.*
O1.4:
Rhombus shaped—*Base is a quadrilateral with opposite acute angles and straight edges. It is only up to two times as long laterally than it is from anterior to the posterior. May or may not be symmetrical along the lateral axis.*
O1.5:
Rounded rhombus—*Base is quadrilateral-like with internal acute angles and rounded edges which may be concave.*
O1.6:
Stretched rhombus—*Base is a quadrilateral with opposite acute angles and straight edges and is more than two times longer laterally than from the anterior to the posterior.*
O1.7:
Trapezoid/rhombus hybrid—*Rhombus like but with a flat anterior edge.*
O1.8:
Oval/oval-like—*Rounded and elongated.*
O1.9:
Circular—*Rounded and symmetrical around the center*.
O1.10:
Tree roots—*Irregular with many radiating protrusions, common for spines*.
O1.11:
Mirrors crown shape.
O1.12:
Base extends from crown without distinct separation between crown and base—*Commonly observed on geometric denticles.*

**Fig. 39:**
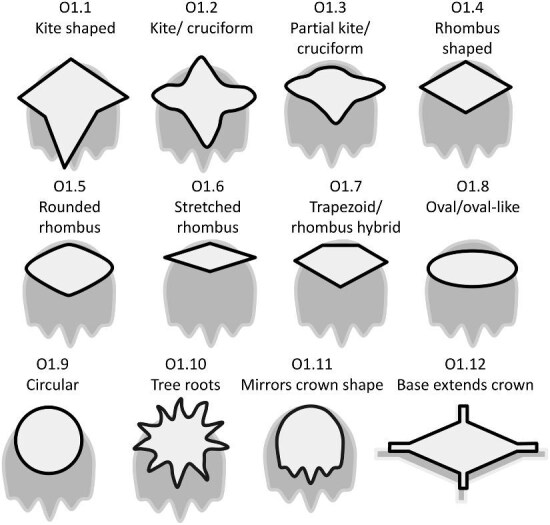
Generalized line drawing examples of character states for Trait O1.

Trait O1 Character Disparity Description: Denticles with an overall base shape described as “tree roots” or radiating protrusions from the root foremen are coded as very different from all other base shapes. Denticles with bases that mirror the crown shape are also coded as very different from other base shapes (though less different than those with “tree root” shapes). All other base shapes are coded as equally different from each other. Denticles with no discernable difference between the base and crown are coded as the most significantly different from other base shape trait states. Weight in disparity calculation: 1.

#### **Trait O2: Base width/length** (Fig. 40)

The base length is defined by the anterior/posterior axis and the width is defined by the width of the lateral edges.

O2.0:
Base not preserved or base not observable.
O2.1:
Equal width and length.
O2.2:
Wider than long—*Wider laterally than long (anterior to posterior).*
O2.3:
Longer than wide—S*horter laterally than long (anterior to posterior).*
O2.4:
Unequal width and length (no directionality).

**Fig. 40:**
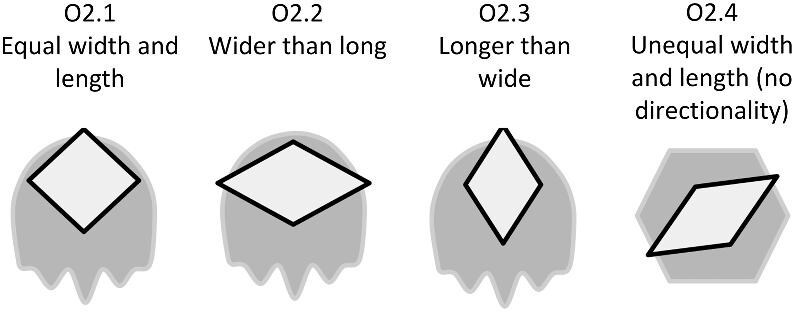
Generalized line drawing examples of character states for Trait O2.

Trait O2 Character Disparity Description: Denticles with no identifiable directionality and thus no identifiable width or length are coded as very different from any denticle with a base that does have an identifiable width and length. Denticles with bases that have equal width and length, a width larger than its length, and larger lengths than widths are all coded as equally different from each other. Weight in disparity calculation: 1.

#### **Trait O3: Crown to base ratio** (Fig. 41)

The crown to base ratio describes the size of the crown compared to the size of the base.

O3.0:
Base not preserved or base not observable.
O3.1:
Crown and root have the same area.
O3.2:
Crown has a larger area than base.
O3.3:
Base has a larger area than crown.

**Fig. 41:**
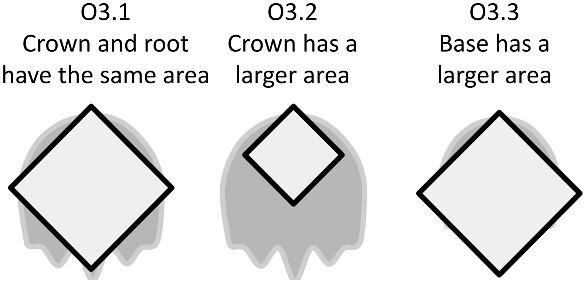
Generalized line drawing examples of character states for Trait O3.

Trait O3 Character Disparity Description: Denticle's crown to base ratio trait states are all coded as equally different from other trait states of this character. Weight in disparity calculation: 1.

#### **Trait O4: Number of grooves** (Fig. 42)

Grooves are lines in the concave underside of the base. They generally point inwards toward the base foramen growing less defined as they reach the center.

O4.0:
Base not preserved or base not observable.
O4.1:
No grooves, completely rounded/smooth.
O4.2:
One.
O4.3:
Two.
O4.4:
Three.
O4.5:
Four.
O4.6:
Five.
O4.7:
Six (plus).

**Fig. 42:**
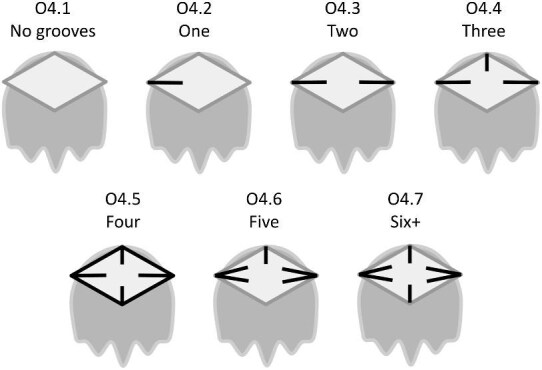
Generalized line drawing examples of character states for Trait O4.

Trait O4 Character Disparity Description: Denticles with bases that have no grooves are coded as very different from bases with grooves. All denticles with any number of grooves are coded as equally different from each other. Weight in disparity calculation: 1.

#### **Trait O5: Root foramen opening shape** (Fig. 43)

The root foramen is defined as the canal, which connects the bottom of the base of the denticle up into the crown and it can differ in shape between denticles.

O5.0:
Base not preserved or base not observable.
O5.1:
No root opening.
O5.2:
Rhombus—*Quadrilateral with opposite acute angles and straight edges.*
O5.3:
Elipse—*Round and elongated.*
O5.4:
Arc—*A slice of a circle with a rounded anterior edge and a posterior vertex.*
O5.5:
Mirrors base shape with more rounded edges.

**Fig. 43:**
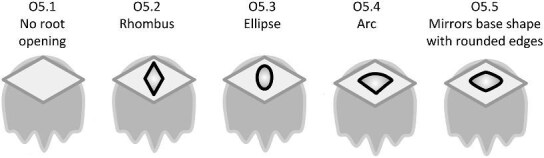
Generalized line drawing examples of character states for Trait O5.

Trait O5 Character Disparity Description: Denticles with root foramens of any shape are all coded as equally different from each other. Denticles with no root foramen are coded as more different than denticles with a root foramen. Weight in disparity calculation: 1.

#### **Trait O6: Root foramen location** (Fig. 44)

The root foramen is always located somewhere within the outer edges of the base, but can be closer to the anterior or posterior.

O6.0:
Base not preserved or base not observable or no root opening.
O6.1:
Center of base.
O6.2:
Anterior of base.
O6.3:
Posterior of base.

**Fig. 44:**
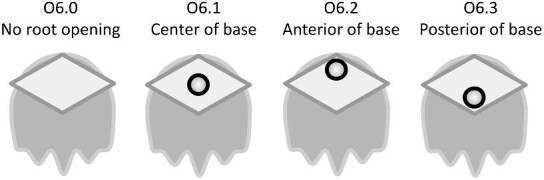
Generalized line drawing examples of character states for Trait O6.

Trait O6 Character Disparity Description: Denticles with a root foramen are all coded as equally different from each other. Denticles with no root foramen are coded as more different from other denticles. Weight in disparity calculation: 1.

#### **Trait O7: Peduncle height: width** (Fig. 45)

The peduncle is the vertical structure which connects the base and crown on some denticles.

O7.0:
Base not preserved or base not observable.
O7.1:
Equal width and height.
O7.2:
Wider than height.
O7.3:
Higher than width.
O7.4:
No Peduncle—*Peduncle is continuous (not distinguishable) from the top of the crown which appears to sit flat on the skin surface, common for geometric denticles.*

**Fig. 45:**
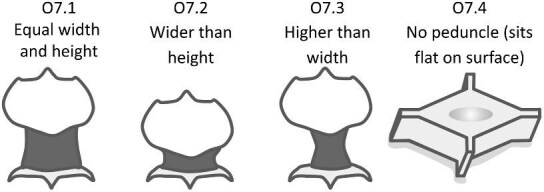
Generalized line drawing examples of character states for Trait O7.

Trait O7 Character Disparity Description: Denticles with peduncles that have equal width and height, width larger than height and height larger than width are all coded as equally different from each other. Denticles with no peduncle are coded as more different than denticles with peduncles. Weight in disparity calculation: 1.

#### **Trait O8: Crown to root angle** (Fig. 46)

The crown to root angle is described by the angle between the skin and crown. Spines often display a perpendicular angle, while most other denticles display an obtuse or parallel angle. Acute angles are relatively rare.

O8.0:
Base not preserved or base not observable.
O8.1:
Perpendicular—*Crown is completely vertical.*
O8.2:
Obtuse—*Crown's posterior is pointed up vertically and higher than the anterior.*
O8.3:
Parallel—*The crown is parallel to the skin with both the posterior and anterior at equal vertical heights.*
O8.4:
Acute—*Crown's posterior is pointed down vertically and is lower than the anterior.*

**Fig. 46:**
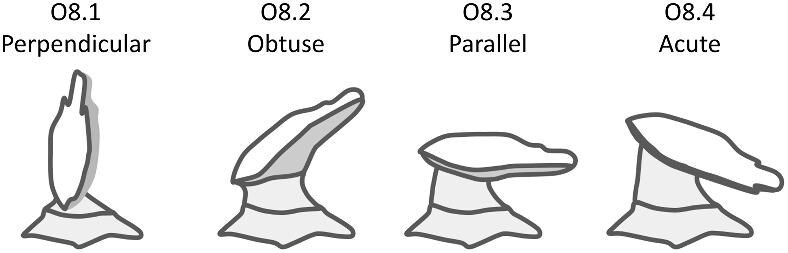
Generalized line drawing examples of character states for Trait O8.

Trait O8 Character Disparity Description: The crown-to-base angle is a semi-continuous trait with perpendicular being coded as closest to obtuse, then parallel, then acute. Weight in disparity calculation: 1.

#### **Trait O9: Base to crown connection location** (Fig. 47)

The Base to crown connection location describes the part of the crown where the base, either directly or via peduncle, is located.

O9.0:
Base not preserved or base not observable.
O9.1:
At anterior of crown.
O9.2:
At center of crown—*Note this includes denticles which have a base which is connected to the entire crown.*
O9.3:
At posterior of crown.

**Fig. 47:**
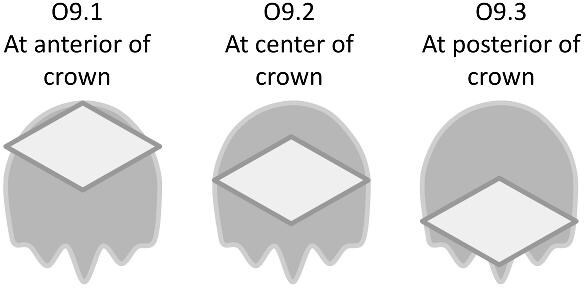
Generalized line drawing examples of character states for Trait O9.

Trait O9 Character Disparity Description: Denticles' base to crown connection location is coded as being equally different from each other. Weight in disparity calculation: 1.

#### **Trait O10: Mound** (Fig. 48)

Mounds are defined by a raised portion below the crown but above the skin surface, which is larger in area than the crown.

O10.0:
Base not preserved or base not observable.
O10.1:
Not mounded—*the base to crown connection is surrounded by a smooth flat area around where the crown emerges.*
O10.2:
Mounded—*The base to crown connection is surrounded by a raised area where the crown emerges.*

**Fig. 48:**
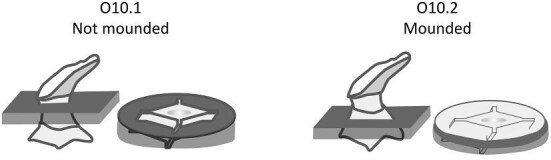
Generalized line drawing examples of character states for Trait O10.

Trait O10 Character Disparity Description: Denticles with a mound on the skin surface are coded as different from denticles with only the crown erupting through the skin (non-mounded). Weight in disparity calculation: 1.

**Fig. 49:**
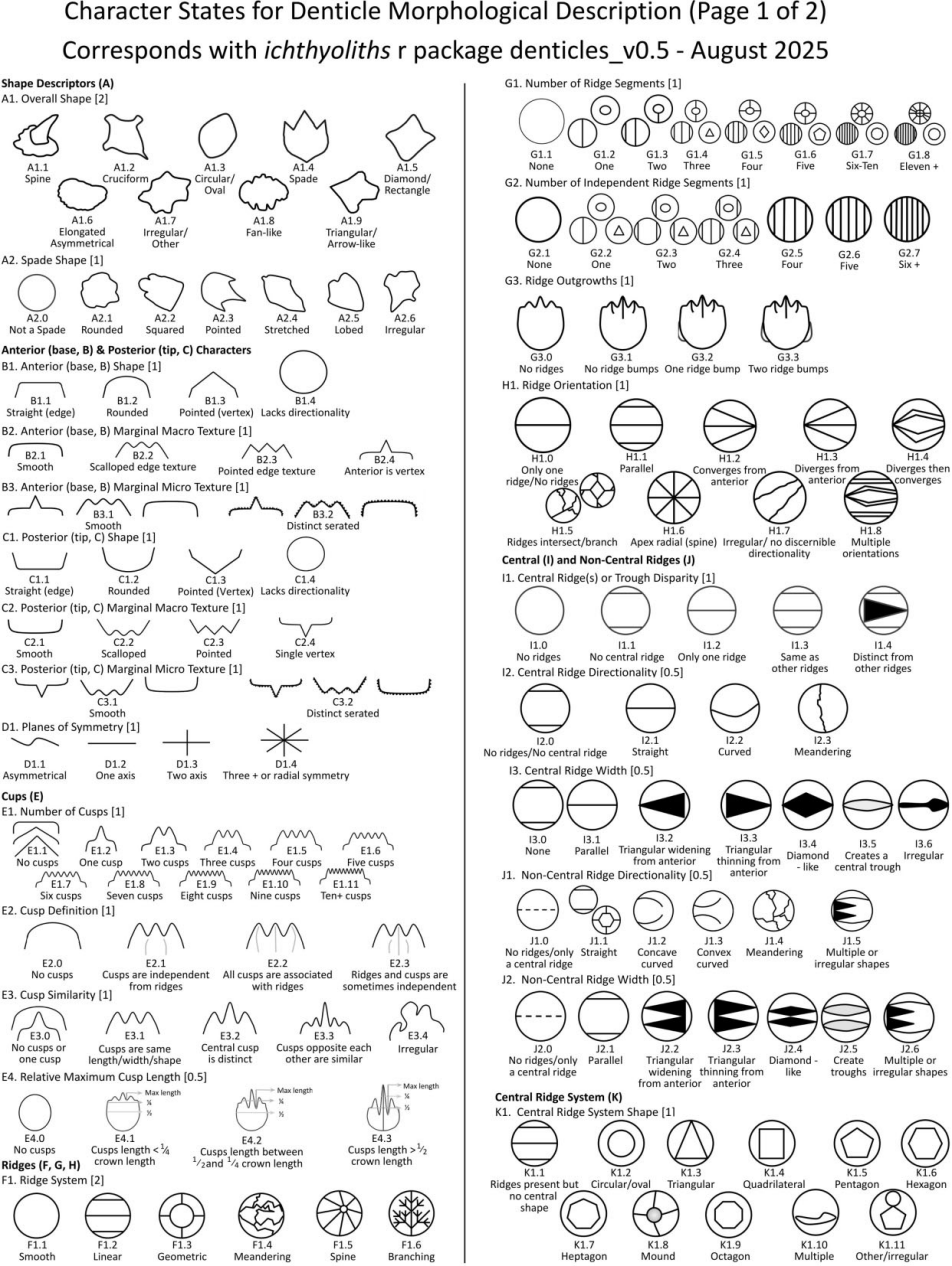

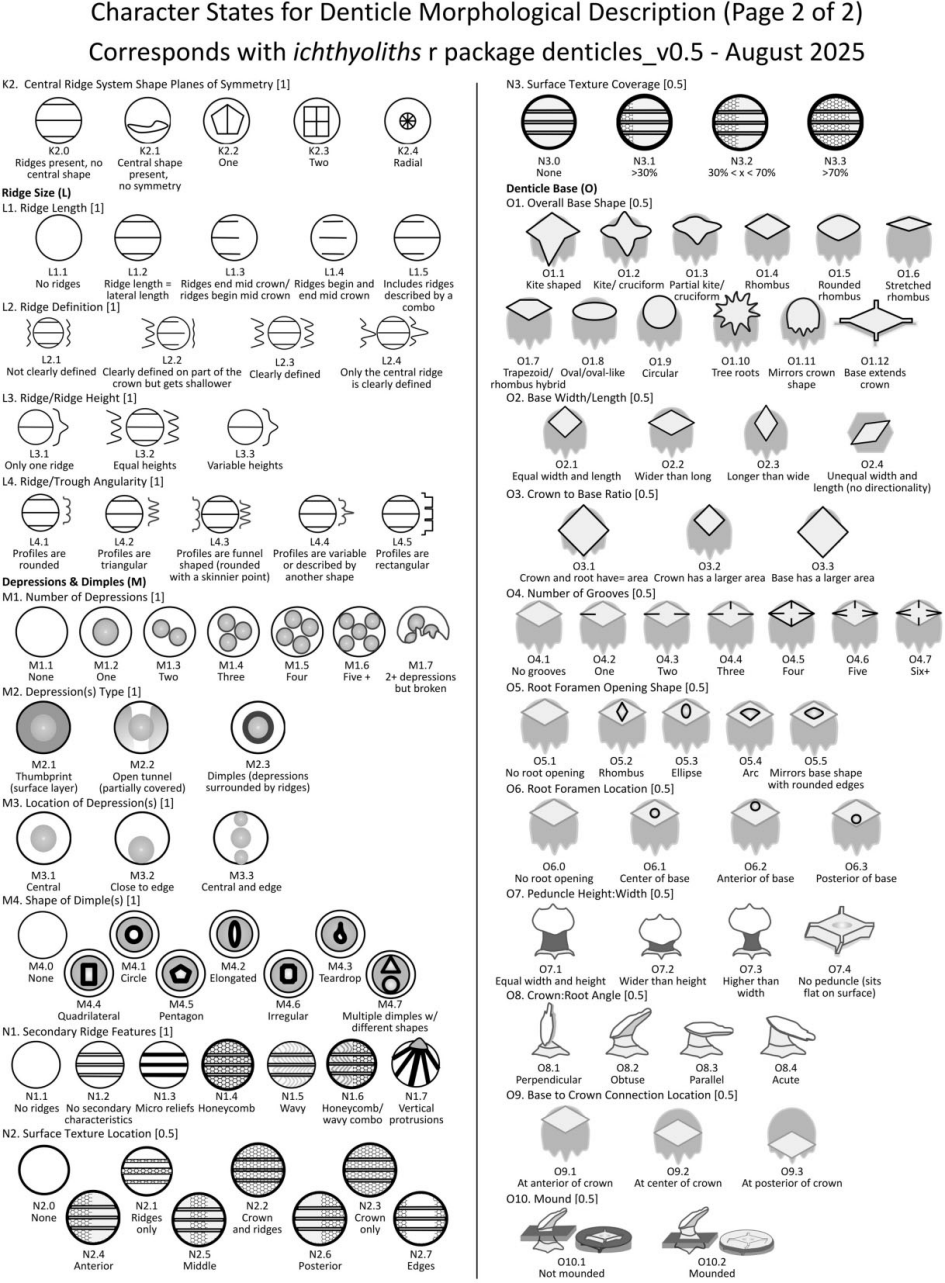
Line drawings of all traits and character states, also available as Appendix 2.

**Fig. 50:**
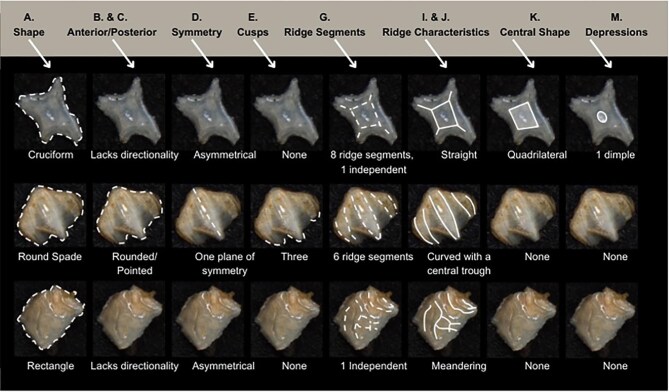
Example denticles with character traits highlighted.

## Adding to the morphological character code

The present morphometric framework fully captures all of the morphological disparity that we have observed in both extant and fossil denticles, and is based on a dataset of well over 5000 denticles, compiled from our own fossil datasets spanning the past 85 million years, as well as a widespread literature review of modern and fossil shark denticle diversity. However, as more denticles are added to this dataset, particularly those from pelagic open ocean datasets going back to the Cretaceous and beyond, novel morphologies are inevitable. We have designed this character coding scheme as well as the associated R package to be inherently flexible and straightforward to update as novel characteristics are found that cannot be quantified in the present version.

To facilitate including previously coded denticles in future analyses, the simplest way to add to the code is to simply add a new character state to an existing character, and to then update the disparity matrix for that trait accordingly. No previously coded denticles will require updates to their code in this situation.

Should adding a new state be insufficient to effectively quantify the newly observed morphology, the addition of a novel character is appropriate. It is essential, if possible, to define the new character trait in such a way that all previously coded denticles have the same state as each other, with the new denticle(s) that inspired the additional character trait as the only denticles which vary. This facilitates placement of novel denticles within the context of all prior coded denticles without having to manually re-code everything in the database. In this case, an additional trait matrix would be constructed and appended to the R package-based disparity calculations.

All additions to the character coding scheme should be fully documented in the relevant publication. If integrated into the R package, they will be given unique coded version numbers, which will be stored and able to be used within the R package for future analyses. The current code version is denticles_v0.5. If an update is made to individual character states, the version will go up by 0.1 increments (e.g., v1.1 may contain an update to Trait N1). Full novel character traits will increase the version by increments of 1 (e.g., v2.0 may include an additional Trait B3). All prior versions of the denticle code are maintained within the package to facilitate analyses of historical denticle datasets. We encourage all users of the code to document and submit their updated code versions to the R package, to centralize communication, and facilitate repeatable, open science.

## Defining denticle morphotypes and groups

While it is not presently possible to identify specific denticles to individual species, denticles have unique morphotypes, which can be incredibly useful as units for discussion and analysis. Here, we define a “morphotype” as a unique combination of character states. Using this code, we identified 160 denticle types and 2 catch all types in our modern and fossil datasets from both published literature and microfossil data; these types represent denticles, which appeared visually similar and had either identical morphometric codes, or differed by only one or two specific traits. In addition to individual morphotypes, morphotype groups (“gross types”) were established, which group individual denticle morphotypes with similar characteristics (Table 1). These groupings facilitate conversation, and are visually determined, based on overall denticle appearance. They are defined independent of the disparity analysis calculation. The overall code is outlined below, with specific annotated examples given for each character and character state in Appendix 1. We envision that this grouping will evolve as more denticle morphotypes are observed.

**Table 1:** Gross Type Descriptions

Gross Type	Description
**Arrowheads, Airplanes, Diamonds, and Triangles**	**Description:** Morphotypes in the arrowheads, airplanes, diamonds, and triangles gross type category usually have a linear ridge orientation with clear directionality and have one or more edges which converge in a terminal vertex. ***Morphotypes****: Airplane, Angular Edged Diamond, Angular Straight Edge, Arrowhead, Bilateral Single Ridged Arrowhead, Central Ridged Diamond, Central Ridged Triangle, Complex Central Ridged Triangle, Flying Squirrel, Funky Central Ridged Diamond, Geometric Arrowhead, Extended Forked Arrow, High Ridged Diamond, Short Troughed Diamond, Stretched Airplane, Triangular Troughed Arrow, Troughed Diamond*
**Bilateral Crowns**	**Description:** Morphotypes in the bilateral crown gross type category have a single ridge which bifurcates the crown and no other discernible traits. ***Morphotypes****: Bilateral Fan, Bilateral Single Ridged, Stretched Bilateral Central Ridged*
**Criss Cross**	**Description:** Morphotypes in the criss-cross gross type category are defined by four ridges which radiate from a central point or shorter ridge. ***Morphotypes****: Asymmetrical Criss Cross, Five Ridged Criss Cross,* *and* *Symmetrical Criss Cross*
**Kites**	**Description:** Morphotypes in the kite gross type category can also be described within the single dimpled complex polygon gross type. They have a rectangular or cruciform shape with a four, five, or six sided central shape which outlines a dimple or a mound. ***Morphotypes****: Asymmetrical Kite with Oval Center, Asymmetrical Kite with Ridged Mound, Diamond Kite, Generic Kite, Kite with Bubble, Kite-Like, Sharp Kite,* *and* *Wrinkly Kite*
**Many Ridged Fans and Spades**	**Description:** Morphotypes in the many ridged fans and spades gross type category have a fan or spade shape and have five or more linear running ridges. Note that “fan” is used for denticles that are wider than they are long. ***Morphotypes****: Five Thin Ridged Fan, Many Ridged Linear Fan, Many Thin Ridged Spade, Rake, Shallow Ridged Wide Crown,* *and* *Three Ridged Linear Fan with Ridge Outgrowths*
**Multi Dimpled Complex Polygons**	**Description:** Morphotypes in the multi dimpled complex polygon gross type category have a geometric central branching ridge orientation outlining two or more dimples. ***Morphotypes****: Bunny Ears, Double Dimpled Hexagon, Generic Double Dimpled Complex Polygon, Many Celled, Many Celled Central Complex Polygon, Stretched Two Dimpled Spade, Triple Dimpled Crown, Two Dimpled Fan, Two Dimpled Fan with Tail,* *and* *Two Dimpled Rectangle*
**Petals**	**Description:** Morphotypes in the petal gross type category have one or no cusps and are defined by rounded side edges and at least one terminal vertex. ***Morphotypes****: Bottom Bulbed Three Ridged Petal, Central Ridged Petal, Central Troughed Petal, Extended Central Long Troughed Petal, Extended Central Troughed Petal, Circular Petal, Four Thin Ridged Petal, Madeleine, Many Ridged Petal, Minnie Indented Shell, Single Ridged Petal, Stretched Minnie Indented Shell,* *and* *Three Ridged Petal*
**Ridged Circles**	**Description:** Morphotypes in the ridged circle gross type category have a circular shape and linear ridge orientation. ***Morphotypes****: Central Ridged Textured Circle, Minnie Ridged Circle, Ridged Circle,* *and* *Shallow Ridged Circle*
**Scoops**	**Description:** Morphotypes in the scoop gross type category have a central depression which composes the entirety of the crown surface and which may or may not be surrounded by ridges. ***Morphotypes****: Caldera Diamond, Cracked Kite, Spear, Tunnel,* *and* *Volcano*
**Single Dimpled Complex Polygons**	**Description:** Morphotypes in the single dimpled complex polygon gross type category have a geometric central branching ridge orientation outlining a single central dimple. ***Morphotypes****: Bilateral Polygon, Eyeball, Fusiform Dimpled Diamond, Generic Single Dimpled Complex Polygon, Helter Skelter, Raised Dimple, Rounded Cone, Single Dimpled Octagon, Single Dimpled Heptagon, Single Dimpled Hexagon, Single Dimpled Hexagon with Serrated Edges, Single Dimpled Pentagon,* *and* *Single Dimpled Quadrilateral*
**Smooth**	**Description:** Morphotypes in the smooth gross type category are defined as having no ridges. These denticles can differ in edge textures, numbers of cusps, and overall shape. ***Morphotypes****: Cheeky, Smooth, Smooth Arrow, Smooth Extended Trident, Smooth Nubbed, Smooth Petal,* *and* *Smooth Scalloped*
**Spines**	**Description:** Morphotypes in the spine gross type category have a spine shape with a crown that emerges from the skin at a vertical angle and then continues vertically or angles down to be parallel over the skin. ***Morphotypes****: Circular Tipping Spine, Fusiform spines, Nub Spine, Radial Angled Spines,* *and* *Triangular Spines*
**Tridents**	**Description:** Morphotypes in the trident gross type category have three cusps and/or three ridges. They usually have a linear ridge orientation and have a clear directionality. ***Morphotypes****: Big Three Ridge, Central Five Ridged Extended Trident, Central Ridged Extended Trident, Chubby Trident, Dagger, Eared Trident, Extended Central Ridged and Textured Trident, Lobular Trident, Lopsided Trident, Madeleine Trident, Short Many Ridged Trident, Many Ridged Trident, Round Bottomed Textured Tulip, Round Ridged Trident, Round Sided Trident, Textured Trident, Thin Ridged Trident, Trident, Trident with One Side Outgrowth, Trident with Pointed Base, Trident with Rounded Base, Troughed Trident,* *and* *Two Ridged Troughed Trident, Wavey Trident, Wide Thin Ridged Trident, Wrapped Trident*
**Wedges**	**Description:** Morphotypes in the wedge gross type category have at least one straight or only slightly curved edge and at least two edges which converge in a vertex. ***Morphotypes****: Elongated Asymmetrical,* *Pear-Shaped* *Wedge, Skinny Pear Shaped Wedge, Two Big Lobes, Two Ridged Wedge, Two Sectioned Wedge, Wedged Branching Crown,* *and* *Whale Blow*
**Other Branching**	**Description:** Morphotypes in the general branching gross type category have a branching ridge orientation but no other distinguishable traits. ***Morphotype****: Branching Fragment*
**Other Geometric**	**Description:** Morphotypes in the general geometric gross type category have a geometric ridge system but no other distinguishable traits. ***Morphotype****: GenGeo*
**Other Linear**	**Description:** Morphotypes in the general linear gross type category have a linear ridge orientation but no other distinguishable traits. ***Morphotypes****: Crinkle Tops, Elephant, Four Ridged Linear Spade, Funky Central Ridged Spade, Llama, Right Angled Five Ridge, Single Ridged Spade,* *and* *Wilted Tulip, Zebra Hoof*
**Other Meandering**	**Description:** Morphotypes in the meandering gross morphotype have meandering ridges. ***Morphotypes****: Branching and Flared Chunk, Wrinkly*

## Quantifying denticle morphospace

**Fig. 51:**
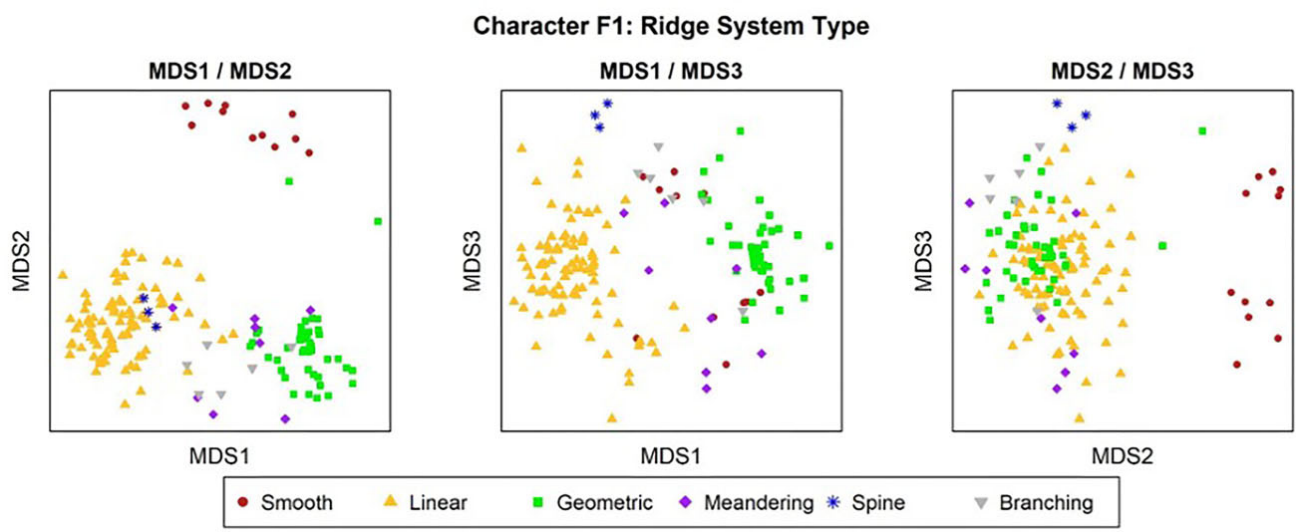
A three-dimensional NMDS-based morphospace ordination highlighting the 6 denticle ridge system trait states (F1) for all denticle morphotypes defined in this publication, showing clear groupings between the different denticle types in the three-dimensional space. This figure and others exploring other character states, can be reproduced using the example code published in the ichthyoliths R package and included in the supplemental materials here.

The morphometric code produces a morphospace, which allows for comparative observations and groupings of denticles. As the morphometric code is composed of categorical, rather than continuous variables, NMDS (non-metric multidimensional scaling) is used to reduce dimensionality, and is implemented using the R package *vegan* (Oksanen et al. 2024). Here, we include a representative morphospace with the currently described denticle morphotypes included in the appendices to this manuscript (Fig. 51). We find that denticle ridge system type (character F1) is a strong indicator of morphological similarity, while the overall shape of the denticle (Trait A1), which varies considerably within otherwise similar denticles, is less diagnostic. The full morphospace can be recreated and explored using the supplemental example R code vignette (Appendix 5, also available at https://github.com/esibert/ichthyoliths/blob/master/example_code/dentmorph_v0.5_Example_code.R). The morphospace tool holds considerable potential for exploring within- and between-taxa denticle morphological diversity, as well as providing a framework for taxonomic identifications of fossil denticle types.

## Conclusions: The importance of morphological code and morphotype framework for supporting future denticle research

Research on dermal denticles is a growing field and this morphological code provides a common language for scientists to collaborate and compare their findings. Denticle research spans disciplinary boundaries, from advancing the understanding of denticle hydrodynamics (Wen et al. 2015) to studying the ontogenetic and developmental origins of denticles (Reif 1985; Cooper et al. 2023; Vaz et al. 2023), to paleontological studies going back over 450 million years across marine ecosystems (Turner 2004; Feichtinger et al. 2020; Sibert and Rubin 2021). Denticles have even been applied to conservation paleobiology on coral reefs (Dillon et al. 2022). This novel coding scheme has been developed based on the currently known morphological diversity of dermal denticles, from both modern and fossil specimens as well as a range of imaging techniques, from light microscopy to scanning electron microscopy to micro-CT scans. We have also created a user-friendly set of tools including a spreadsheet with dropdown menus, simplified two-page “cheat sheet” that combines all of the character states (Fig. 49, Appendix 2, we have also highlighted some of the characters on photographs of denticles above Fig. 50), and a publicly accessible R package to facilitate disparity analyses. Quantifying the breadth and variability of denticle morphology across time, ontogeny, and taxonomy has the potential to transform our understanding of shark evolutionary history, ecology, and developmental biology, and will provide a framework for interdisciplinary work on this important part of vertebrate morphology.

## Supplementary Material

obaf021_Supplemental_Files

## Data Availability

All denticles used in this manuscript are available in the supplementary documentation: the spreadsheet of all coded morphotypes, the powerpoint of all morphotypes, and the denticles tables; The R package, vignette, and morphotype coded dataset is available at https://www.github.com/esibert/ichthyoliths.
