# Supplementary material for: Quantifying the Denticle Multiverse: A Standardized Coding System to Capture Three Dimensional Morphological Variations for Quantitative Evolutionary and Ecological Studies of Elasmobranch Denticles
Source: Integr Org Biol. 2025 May 13;7(1):obaf021. doi: 10.1093/iob/obaf021 (PMC12576789; doi:10.1093/iob/obaf021)
Supplement: obaf021_Supplemental_Files [file obaf021_supplemental_files.zip › Appendix_01-Denticle Character States_Cleaned Copy of Revised Version of Denticle Table.pdf]

Denticle Character State Examples

This document is meant to act as a guide for denticle character coding. It includes example denticles that are representative of nearly all possible character states. The majority of images are from our own modern and fossil datasets, however previously published images have been included as necessary.

Table of Contents:

Trait A1: General denticle Shape.....3
Trait A2: Spade Subtype.....6
Trait B1: Anterior/Base Shape.....9
Trait B2: Anterior Marginal Macro Texture Description.....11
Trait B3: Anterior Marginal Micro Texture Description.....13
Trait C1: Posterior Shape.....14
Trait C2: Posterior Marginal Macro Texture Description.....16
Trait C3: Posterior Marginal Micro Texture Description.....18
Trait D1: Planes of Symmetry.....19
Trait E1: Number of cusps.....21
Trait E2: Cusps definition.....23
Trait E3: Cusp similarity.....25
Trait E4: Maximum Cusp length.....27
Trait F1: Ridge System.....29
Trait G1: Number of ridge segments.....31
Trait G2: Number of independent ridges.....33
Trait G3: Ridge Outgrowths.....35
Trait H1: Ridge Orientation.....37
Trait I1: Central Ridge(s) or trough Disparity.....40
Trait I2: Central Ridge Directionality.....42
Trait I3: Central Ridge Width.....44
Trait J1: Non-Central Ridge Directionality.....46
Trait J2: Non-Central Ridge Width.....48
Trait K1: Central ridge system shape.....50
Trait K2: Central Ridge System Shape Planes of Symmetry.....54
Trait L1: Ridge length.....56
Trait L2: Ridge Definition.....58
Trait L3: Relative Ridge Heights.....60
Trait L4: Ridge/Trough Angularity.....62
Trait M1: Number of depressions.....64
Trait M2: Depression(s) type.....66

Trait M3: Location of depression........68
Trait M4: Shape of dimple(s)........70
Trait N1: Secondary ridge features........73
Trait N2: Surface texture location........75
Trait N3: Surface texture coverage........77
Trait O1: Overall base shape........78
Trait O2: Base width/length........81
Trait O3: Crown to base ratio........83
Trait O4: Number of Grooves........85
Trait O5: Root foramen opening shape........87
Trait O6: Root foramen location........89
Trait O7: Peduncle height:width........91
Trait O8: Crown:root angle........93
Trait O9: Base to crown connection location........95
Trait O10: Mound........96
References........96

Trait A1: General denticle Shape

Overall shape of denticle: Shape is defined as the overall outline shape of the crown, ignoring any topographical features (e.g. ridges, troughs, etc.) There are many additional potential generic denticle shapes, however we have limited this trait to shapes present in our currently comprehensive dataset. Please see the section about adding to the code for incorporation of additional shapes.

#	Name	Description	Illustration	Image
1	Spine	Taller than it is wide and stands perpendicular to the skin when attached to the body.	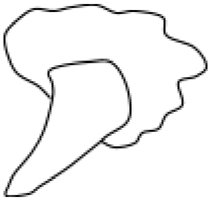	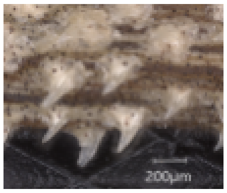 MCZ S1279, *Mitsukurina owstoni,* loc. H
2	Cruciform	"Kite-shaped," or cross-like (†) with two transverse ridges originating from the lateral opposing vertices.	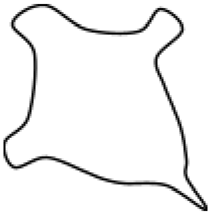	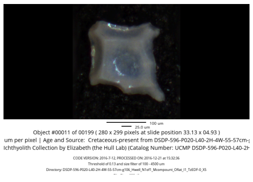 DSDP-596-P020-L40-2H-4W-55-57cm-g106_Hwell_N1of1_obj00011
3	Circular or oval	No vertices, curved outline.	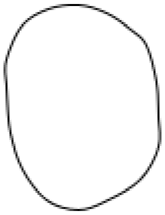	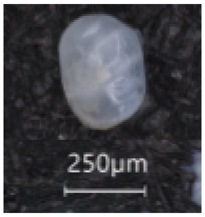 MCZ 32340, *Mustelus manzo*
4	Spade	Any denticle with a symmetric anterior made by two diverging edges of the same relative length which then converge at a perpendicular straight, curved, or pointed posterior edge.	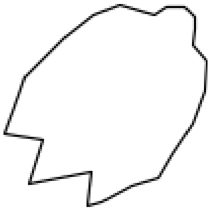	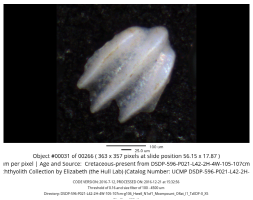 DSDP-596-P021-L42-2H-4W-105-107cm-g106_Hwell_N1of1_obj00031

5	Diamond/ Rectangle	A parallelogram, rhombus or equal cross [+], can be asymmetrical.	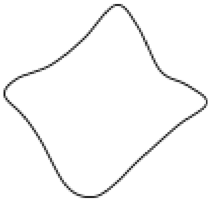	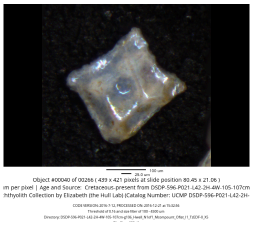 DSDP-596-P021-L42-2H-4W-105-107cm-g106_Hwell_N1of1_obj00040
6	Elongated Asymmetrical	Boot shaped, asymmetric.	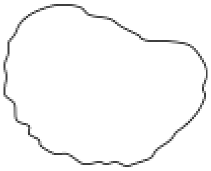	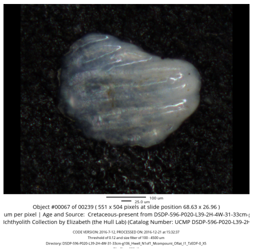 DSDP-596-P020-L39-2H-4W-31-33cm-g106_Hwell_N1of1_obj00067
7	Irregular/ other	Does not fit into any other shape category.	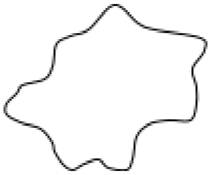	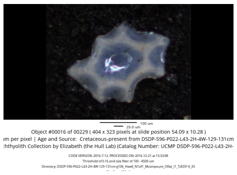 DSDP-596-P022-L43-2H-4W-129-131cm-g106_Hwell_N1of39_obj00016
8	“Fan-like”	Wider than long with only one vertex.	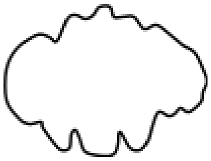	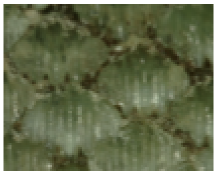 MCZ S-394, *Sphyrna tiburo,* loc. J
9	Triangular or Arrow-like	Outline diverges from the anterior vertex and converges at the apex, concave on either side of at least one of these vertices, and the length of the denticle is equal to or larger than its width.	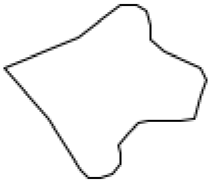	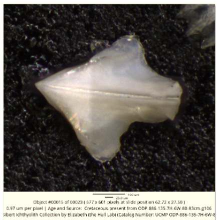 ODP-886-135-7H-6W-80-83cm-g106_obj00015_edf

Trait A1 Character Disparity Description:
Spines are coded as very different from other denticle shapes because they erupt from the skin semi-vertically and their base is attached with the rest of the crown further from the skin rather than laying horizontally over the skin. The cruciform shape has an anterior and posterior which are not necessarily identifiable but are distinct from the side edges of the denticle. Cruciforms are angular and generally associated with depressions/mounds; they are coded as more different from denticles with rounder shapes and denticles which have different shapes of their anterior and posterior. Circular shaped denticles are also coded as more different from other more angular shapes as they do not have identifiable or distinct anterior/posterior.
Weight in disparity calculation: 2.

A1	1	2	3	4	5	6	7	8	9
**1**	0	3	3	3	3	3	3	3	3
**2**	3	0	3	2	1	2	2	2	2
**3**	3	3	0	3	3	3	3	3	3
**4**	3	2	3	0	2	1	2	2	1
**5**	3	1	3	2	0	2	2	2	2
**6**	3	2	3	1	2	0	2	2	2
**7**	3	2	3	2	2	2	0	2	2
**8**	3	2	3	2	2	2	2	0	2
**9**	3	2	3	1	2	2	2	2	0

**Trait A2: Spade Subtype**<br>The "spade" category allows for further description and classification of denticles which have an identifiable anterior and posterior (i.e. directionality) and an anterior which is generally symmetrical. If a denticle falls into the generic "spade" category, there are a number of sub-shapes that it can be, based on the shape and structure of the posterior part of the denticle.				
#	Name	Description	Illustration	Image
0	Not a Spade	Not a Spade	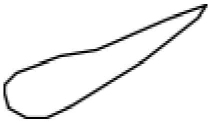	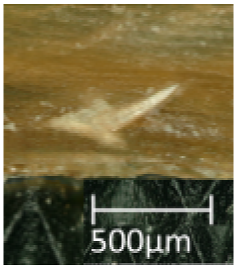<br>MCZ153780, *Etmopterus princeps,* loc. T
1	Rounded Spade	Lateral edges are curved and the angle between these edges and the anterior vertex is obtuse. Any edges and cusps are also rounded.	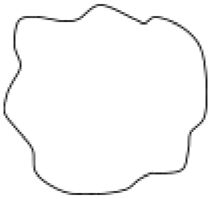	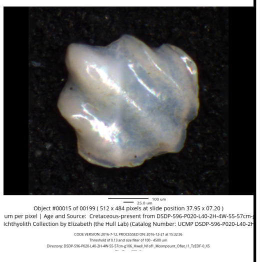<br>DSDP-596-P020-L40-2H-4W-55-57cm-g106_Hwell_N1of1_obj00015
2	Squared Spade	The posterior edge is parallel to the anterior vertex and is flat and/or the lateral edges which connect the anterior and posterior are parallel to each other and relatively flat/straight.	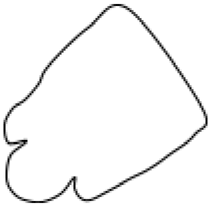	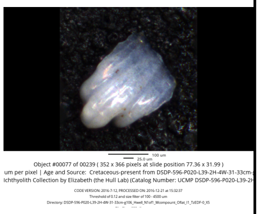<br>DSDP-596-P020-L39-2H-4W-31-33cm-g106_Hwell_N1of1_obj00077

3	Pointed Spade	The posterior edge is composed of cusp(s) or point(s).	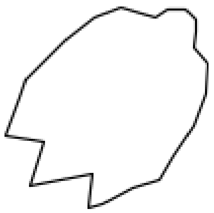	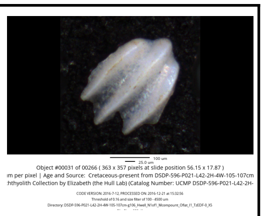 DSDP-596-P021-L42-2H-4W-105-107cm-g106_Hwell_N1of1_obj00031
4	Stretched Spade	The crown has both an anterior and posterior vertex and the posterior has no more than one cusp or has any number of cusps which extend no further than ¼ the length of the crown.	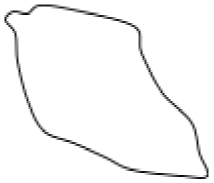	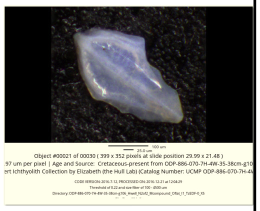 ODP-886-070-7H-4W-35-38cm-g106_obj00021_edf
5	Lobed Spade	Large rounded ridges define the entire crown including rounded lateral edges and a scalloped posterior edge.	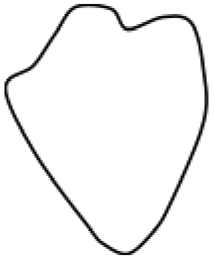	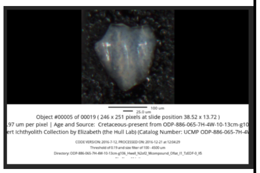 ODP-886-065-7H-4W-10-13cm-g106_obj00005_edf
6	Irregular	Meet the stipulation of at least one single anterior or posterior vertex but do not fall into any of the aforementioned and commonly seen groupings.	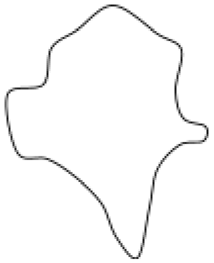	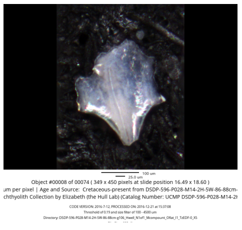 DSDP-596-P028-M14-2H-5W-86-88cm-g106_Hwell_N1of1_obj00008

Trait A2 Character Disparity Description:
Non-spade shaped denticles are coded as very different from spades. Rounded spades are coded as different from most other spade shapes as they have a greater width than length and round posterior shape. The lobed spade also has round edges and is thus marked as more similar to the rounded spade. The pointed spade shape has both a vertex shaped anterior and a pointed posterior. This makes it different compared to the rounded and squared spades but more similar to other denticles with a pointed edge texture like the stretched spade. The heart-shaped spade has 3 rounded portions with a vertex anterior; its rounded edges make it similar to the rounded and lobed spade but different from the more pointed/jagged spades.
Weight in disparity calculation: 0.5.

A2	1	2	3	4	5	6
1	0	2	2	2	1	1
2	2	0	2	2	2	2
3	2	2	0	1	2	2
4	2	2	1	0	2	2
5	1	2	2	2	0	1
6	1	2	2	2	1	0

Trait B1: Anterior/Base Shape
The anterior of the denticle (can also be described as the base of the crown) is defined as the area closer to the anterior (head) of the shark. The anterior cannot always be identified, either due to the denticle being partially broken, obscured from vision, or lacking clear directionality. When a disarticulated denticle is coded it is often possible to assume an anterior/posterior shape based on patterns common amongst linear denticles (linear denticles often have triangular ridges which thin towards the posterior, the troughs, or areas between denticles, generally get wider towards the posterior, and anterior edges are often rounded while posterior edges are generally pointed). For denticles which do not have an obvious discernable anterior (base) or posterior (tip) (e.g. many geometric denticles), character state 4 should be used. Denticles that have distinct directionality but are not fully preserved along the anterior edge should be coded as a 0.

#	Name	Description	Illustration	Image
1	Straight	Is a relatively flat edge.	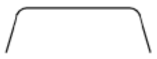	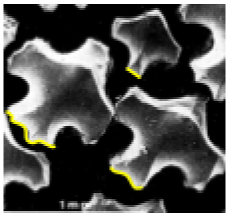<br>*Heterodontus japonicus,* Reif, Pg. 25, Fig. 1D
2	Rounded	A rounded edge with no vertices.	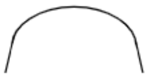	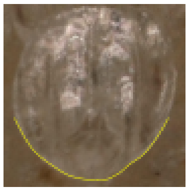<br>MCZ 36995, *Scyliorhinus retifer,* loc. F
3	Pointed	A vertex (or vertices) compose the anterior of the crown.	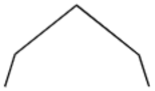	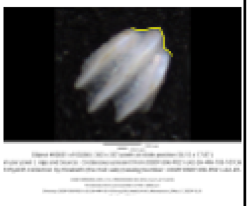<br>DSDP-596-P021-L42-2H-4W-105-107cm-g106_Hwell_N1of1_obj00031

4	Denticle Lacks Directionality /Cannot discern Anterior		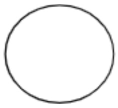	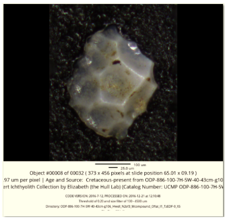<br>ODP-886-100-7H-5W-40-43cm-g106_obj00008_edf

Trait B1 Character Disparity Description:
Denticles with an anterior shape that is not identifiable due to a lack of directionality are coded as very different from other anterior shapes. All other anterior shape trait states differ equally from each other with each trait differing one step from each other trait.
Weight in disparity calculation: 1.

B1	1	2	3	4
1	0	1	1	2
2	1	0	1	2
3	1	1	0	2
4	2	2	2	0

Trait B2: Anterior Marginal Macro Texture Description

B2 can also describe overall marginal texture if unable to discern distinct anterior/posterior areas

The anterior marginal macro texture describes the appearance of the border of a denticle along the anterior of the crown. For denticles with a surrounding mound, code only the upper portion of the crown, and code the mound in Trait O10. For denticles which do not have an obvious anterior (base)/ posterior (tip), but which are not broken (e.g. most geometric denticles), they still have an edge texture. In this case, the edge texture for half of the denticle should be coded in trait B2 and the other half should be coded in Trait C2. If there are distinct regions of differing edge textures along the denticle, code Trait B2 with the lower character score and Trait C2 with the higher character score (Note that the character states for B2 and C2 are identical).

#	Name	Description	Illustration	Image
1	Approximately Smooth	is composed of flat margins with no or only shallow curves or vertices and with no cusps or projections.	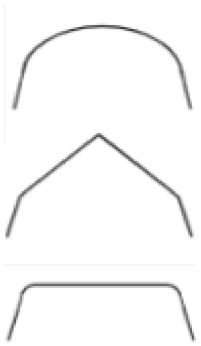	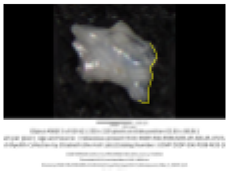 DSDP-596-P038-M33-2H-6W-45-47cm-g106_obj00013
2	Scalloped	The edge has a round and wavy pattern usually defined by terminating ridges.	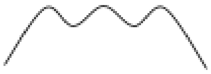	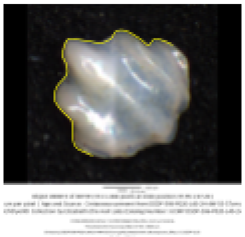 DSDP-596-P020-L40-2H-4W-55-57cm-g106_Hwell_N1of1_obj00015
3	Pointed	The edge is defined by cusps or large points which may or may not be associated with ridges.	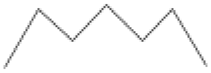	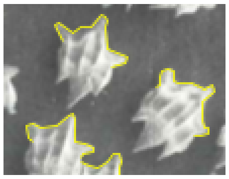 *Hexanchus griseus,* Reif, Pg. 105, Fig. M3

4	Vertex	Anterior is a vertex (can be pointed or somewhat rounded).	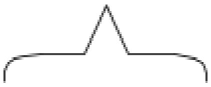	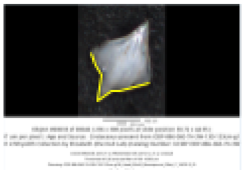 ODP-886-060-7H-3W-130-133cm-g106_obj00033_edf

Trait B2 Character Disparity Description:
Denticles with a smooth anterior macro marginal texture are coded as very different from all other textures and are coded based on their level of difference from this smooth state. Denticles with scalloped and pointed textures are labeled as significantly different from smooth textures while denticles with an anterior vertex are labeled as equally similar/dissimilar from denticles with smooth or vertex textures.
Weight in disparity calculation: 1.

B2	1	2	3	4
**1**	0	2	2	1
**2**	2	0	1	1
**3**	2	1	0	1
**4**	1	1	1	0

Trait B3: Anterior Marginal Micro Texture Description

The marginal micro texture describes the fine-scale texture of the crown's edges independent of marginal shape and the macro texture. Denticles generally have smooth micro texture but some geometric and meandering types may have many jagged points giving the crown a distinctly serrated texture.

#	Name	Description	Illustration	Image
1	Smooth	The anterior may have cusps or points but the micro texture of the anterior shape is smooth.	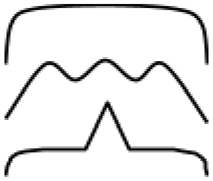	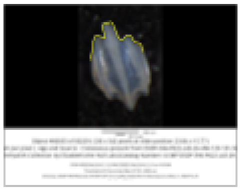 DSDP-596-P022-L43-2H-4W-129-131cm-g106_Hwell_N1of43_obj00033
2	Distinct serrated	Independent of overall shape or general texture, the anterior micro texture is composed of many jagged points.	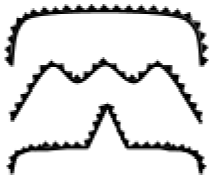	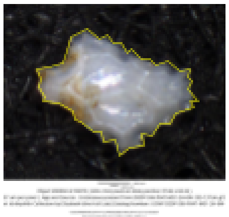 DSDP-596-P047-M51-2H-6W-135-137cm-g106_obj00002

Trait B3 Character Disparity Description:

As there are only two trait states identified for this character, they are coded as equally different from each other.

Weight in disparity calculation: 1.

B3	1	2
1	0	1
2	1	0

Trait C1: Posterior Shape

The posterior of the denticle (can also be described as the tip of the crown) is defined as the area closer to the posterior (or caudal fin) of the shark. The posterior cannot always be identified, either due to the denticle being partially broken, obscured from vision, or lacking clear directionality but when a disarticulated denticle is coded it is often possible to assume an anterior/posterior shape based on patterns common amongst linear denticles (linear denticles often have triangular ridges which thin towards the posterior or tip of the crown, the troughs, or areas between denticles, generally get wider towards the posterior, and the anteriors are often rounded while posteriors are generally pointed). For denticles which do not have an obvious discernable anterior (base) or posterior (tip) (e.g. many geometric denticles), character state 4 should be used. Denticles that have distinct directionality but are not fully preserved along the posterior edge should be coded as a 0.

#	Name	Description	Illustration	Image
1	Straight	Is a relatively flat edge.	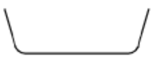	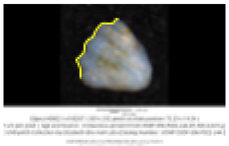 DSDP-596-P022-L44-2H-5W-4-6cm-g106_Hwell_N1of1_obj00021f
2	Rounded	A rounded edge with no vertices.	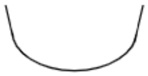	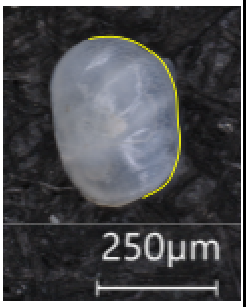 MCZ32340, *Mustelus manzo*, loc. A
3	Pointed	A vertex (or vertices) compose the posterior of the crown.	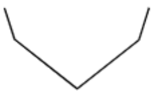	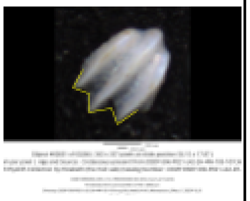 DSDP-596-P021-L42-2H-4W-105-107cm-g106_Hwell_N1of1_obj00031

5	Denticle lacks directionality/ Cannot discern posterior		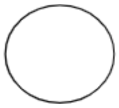	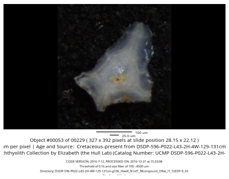 DSDP-596-P022-L43-2H-4W-129-131cm-g106_Hwell_N1of20_obj00053

Trait C1 Character Disparity Description:
Denticles with a posterior shape that is not identifiable due to a lack of directionality are coded as very different from other posterior shapes. All other posterior shape trait states differ equally from each other with each trait differing one step from each other trait.
Weight in disparity calculation: 1.

C1	1	2	3	4
1	0	1	1	2
2	1	0	1	2
3	1	1	0	2
4	2	2	2	0

Trait C2: Posterior Marginal Macro Texture Description

C2 can also describe overall marginal texture if unable to discern distinct anterior/posterior areas

Posterior marginal macro texture describes the appearance of the border of a denticle along the posterior of the crown. For denticles with a surrounding mound, code only the upper portion of the crown, and code the mound in Trait O10. For denticles which do not have an obvious anterior/posterior, but which are not broken (e.g. most geometric denticles), they still have an edge texture. In this case, the edge texture for half of the denticle should be coded in trait B2 and the other half should be coded in Trait C2. If there are distinct regions of differing edge textures along the denticle, code Trait B2 with the lower character score and Trait C2 with the higher character score (Note that the character states for B2 and C2 are identical).

#	Name	Description	Illustration	Image
1	Approximately Smooth	Is composed of flat margins with no or only shallow curves or vertices and with no cusps or projections.	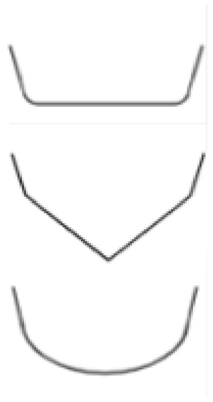	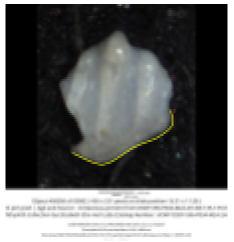 DSDP-596-P034-M24-2H-5W-135-137cm-g106_obj00006
2	Scalloped edge texture	The edge has a round and wavy pattern usually defined by terminating ridges.	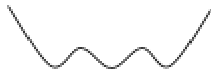	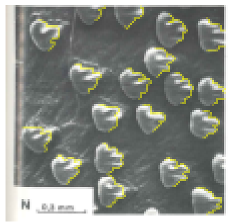 Galeocerdo cuvier, Reif, Pg. 227, Image N
3	Pointed edge texture (more common as posterior texture)	The edge is defined by cusps or large points which may or may not be associated with ridges.	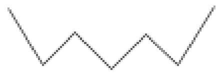	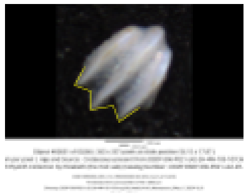 DSDP-596-P021-L42-2H-4W-105-107cm-g106_Hwell_N1of1_obj00031

4	Posterior is a vertex	Posterior is a vertex (can be pointed or somewhat rounded).	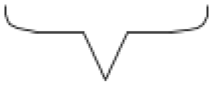	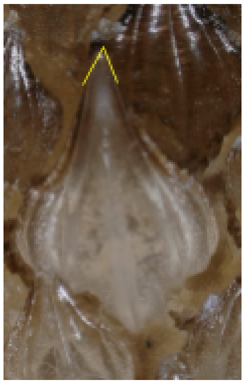 MCZ 36995, *Scyliorhinus retifer,* loc. C

Trait C2 Character Disparity Description:

Denticles with a smooth posterior macro marginal texture are coded as very different from all other textures and are coded based on their level of difference from this smooth state. Denticles with scalloped and pointed textures are labeled as significantly different from smooth textures while denticles with an posterior vertex are labeled as equally similar/dissimilar from denticles with smooth and other edge textures.

Weight in disparity calculation: 1.

C2	1	2	3	4
1	0	2	2	1
2	2	0	1	1
3	2	1	0	1
4	1	1	1	0

Trait C3: Posterior Marginal Micro Texture Description
The marginal micro texture describes the fine-scale texture of the crowns edges independent of marginal shape and the macro texture. Denticles generally have smooth micro texture but some geometric and meandering types may have many jagged points giving the crown a distinctly serrated texture.

#	Name	Description	Illustration	Image
1	Smooth	The posterior may have cusps or points but the micro texture of the anterior shape is smooth.	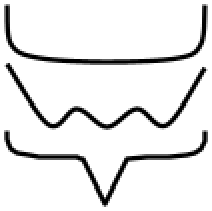	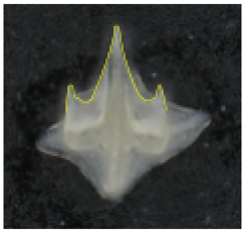 Squalus acanthias - MCZ 32341 (loc. P)
2	Distinct serrated	Independent of overall shape or general texture, the posterior micro texture is composed of many jagged points.	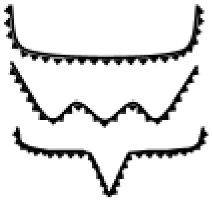	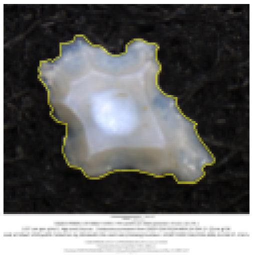 DSDP-596-P024-M04-2H-5W-21-23cm-g106_Hwell_N1of1_obj00012

Trait C3 Character Disparity Description:
As there are only two trait states identified for this character, they are coded as equally different from each other.
Weight in disparity calculation: 1.

C3	1	2
1	0	1
2	1	0

Trait D1: Planes of Symmetry
Defined as the number of axes which can bifurcate the denticle. While small deviations from perfect symmetry (e.g. very slight asymmetries) are common, with such denticles considered ‘symmetrical’, denticles with one-sided additional ridges, ridge-outgrowths, cusps, etc., should be coded as asymmetrical.

#	Name	Description	Illustration	Image
1	None	No planes of symmetry.	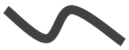	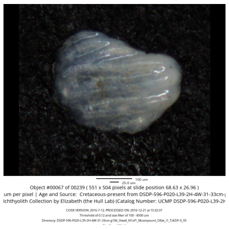 DSDP-596-P020-L39-2H-4W-31-33cm-g106_Hwell_N1of1_obj00067
2	One	One plane of symmetry.		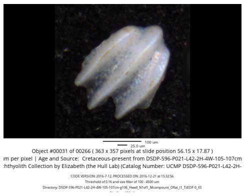 DSDP-596-P021-L42-2H-4W-105-107cm-g106_Hwell_N1of1_obj00031
3	Two	Two planes of symmetry.	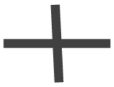	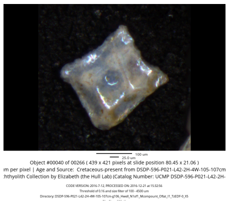 DSDP-596-P021-L42-2H-4W-105-107cm-g106_Hwell_N1of1_obj00040
4	Three+/ Radial	>3 planes of symmetry or Radial symmetry.	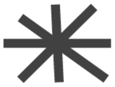	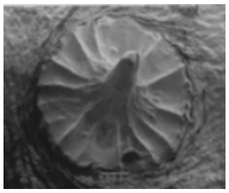 Echinorhinus brucus, Castro, Pg. 44

Trait D1 Character Disparity Description:

The planes of symmetry trait labels denticles with no planes of symmetry as significantly different from those with planes of symmetry and even more different from denticles with radial symmetry. The trait defines denticles with one and two planes of symmetry as being equally different but denticles with radial symmetry as significantly different from denticles with only one or two planes of symmetry and more different from denticles with no planes of symmetry.

Weight in disparity calculation: 1.

D1	1	2	3	4
1	0	2	2	3
2	2	0	1	2
3	2	1	0	2
4	3	2	2	0

**Trait E1: Number of cusps**<br>Cusps are defined as extensions from the posterior or tip of the denticle where two vertical edges meet in a vertex between two horizontal posterior edges. Cusps by definition are only present on denticles with an obvious directionality. If the directionality of the denticle cannot be discerned (such as for most geometric denticles), code the denticle with state E1.1 “No Cusps”. Note that the number of cusps does not match the numeric coded character state value.			
#	Name	Illustration	Image
1	No cusps	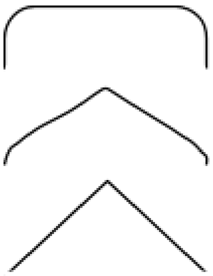	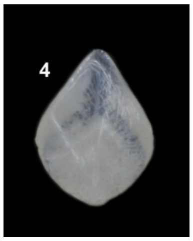 Dillon et al. 2017, Figure 6 (4)
2	One cusps	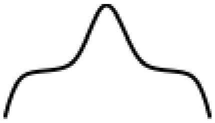	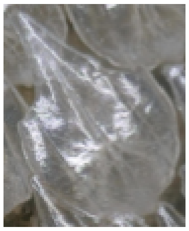 MCZ 36995, *Scyliorhinus retifer,* loc. Y
3	Two cusps	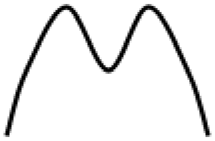	N/A
4	Three cusps	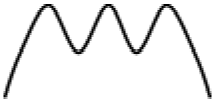	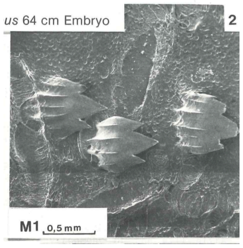 Hexanchus griseus, Reif, Pg. 105, Fig. M1

5	Four cusps	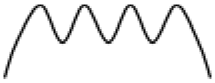	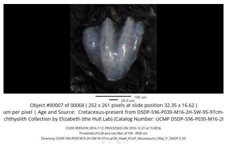 DSDP-596-P030-M16-2H-5W-95-97cm-g106_obj00007
6	Five cusps	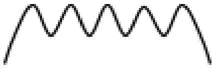	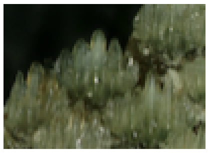 MCZS-394, *Sphyrna tiburo*, loc. J
7	Six cusps	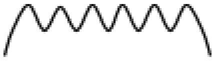	N/A
8	Seven cusps	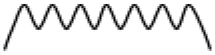	N/A
9	Eight cusps	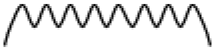	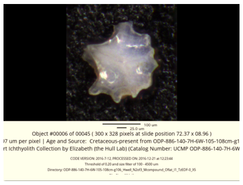 ODP-886-140-7H-6W-105-108cm-g106_obj00006_edf
10	Nine cusps	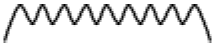	N/A
11	Ten or more cusps	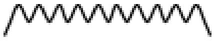	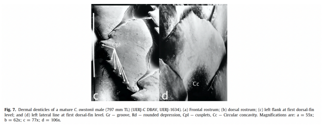 Fig. 7 of Rincon et al (2021).

Trait E1 Character Disparity Description:

The number of cusps trait has states which generally differ in equal amounts when cusps are present with increasing numbers of cusps differing in one step increments from each other. Denticles with no cusps are coded as significantly different from those with cusps and denticles with only one cusp are labeled as more different from multi-cusped denticles.

Weight in disparity calculation: 0.5

E1	1	2	3	4	5	6	7	8	9	10	11
1	0	3	3	3	3	3	3	3	3	3	3
2	3	0	2	2	2	2	2	2	2	2	2
3	3	2	0	1	1	1	1	1	1	1	1
4	3	2	1	0	1	1	1	1	1	1	1
5	3	2	1	1	0	1	1	1	1	1	1
6	3	2	1	1	1	0	1	1	1	1	1
7	3	2	1	1	1	1	0	1	1	1	1
8	3	2	1	1	1	1	1	0	1	1	1
9	3	2	1	1	1	1	1	1	0	1	1
10	3	2	1	1	1	1	1	1	1	0	1
11	3	2	1	1	1	1	1	1	1	1	0

**Trait E2: Cusp-idge association**<br>Describes whether cusps are associated with or independent from ridges. Most denticles with cusps have ridges that run the length of the crown terminating at the tips of the cusps.				
#	Name	Description	Illustration	Image
0	No cusps	The posterior edge of the crown does not have any cusps.	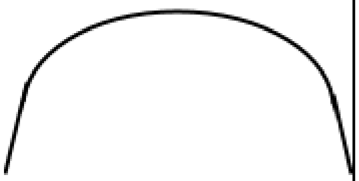	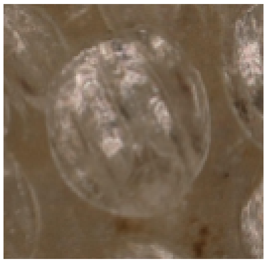<br>*Scyliorhinus retifer,* MCZ 36995 (loc. F)
1	Ridges not associated with cusps	Cusps are present but there are either no ridges or ridges which do not run down the center of the cusp.	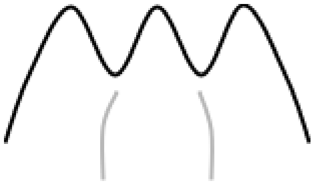	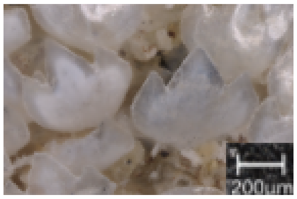<br>*Asymbolus analis*, MCZ793 (loc. F)
2	Cusps associated with ridges	Each cusp has a ridge which runs along its length, usually down the center of the cusp.	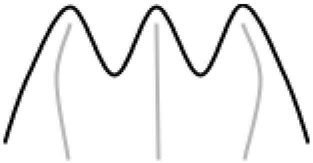	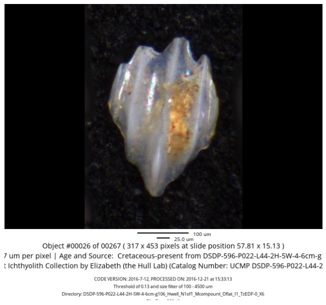<br>DSDP-596-P022-L44-2H-5W-4-6cm-g106_Hwell_N1of1_obj00026

3	Some cusps associated with ridges/some ridges define cusps	There are both cusps and ridges present but only some cusps have ridges which run their length.	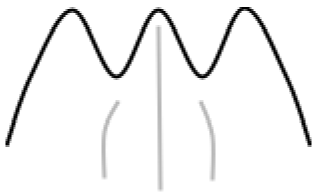	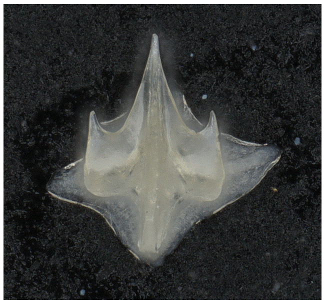 *Squalus acanthias* - MCZ 32341 (loc. P)

Trait E2 Character Disparity Description:
Denticles with cusps are coded as very different from denticles that have no cusps. Denticles with ridges associated with cusps and denticles with cusps not associated with ridges are coded as somewhat different and denticles with some cusps associated with ridges are equally similar/dissimilar to other denticles with cusps.
Weight in disparity calculation: 0.5

E2	1	2	3
1	0	2	1
2	2	0	1
3	1	1	0

Trait E3: Cusp similarity
Comparison between cusps length, width, and shape. It is only applicable for denticles with more than one cusp to compare between.

#	Name	Description	Illustration	Image
0	None/one	No cusps or only one cusp.	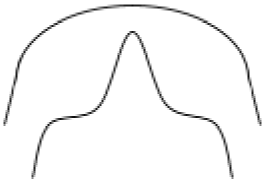	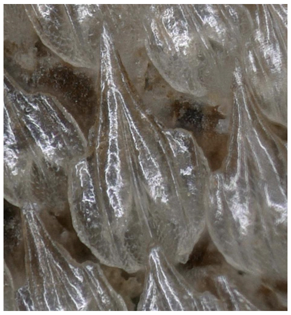 MCZ 36995, *Scyliorhinus retifer,* loc. H
1	Cusps are same length / width / shape	Cusps are the same length, width, and shape.	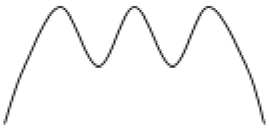	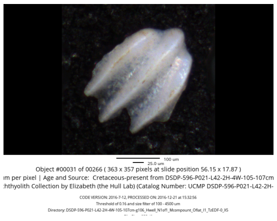 DSDP-596-P021-L42-2H-4W-105-107cm-g106_Hwell_N1of1_obj00031
2	Central cusp is distinct	Only the central cusp is distinct - meaning it is either a different size, or a different shape than side cusps. Central cusps are often longer and wider and more triangular while side cusps are often shorter and slightly curved.	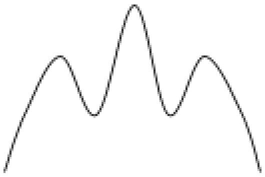	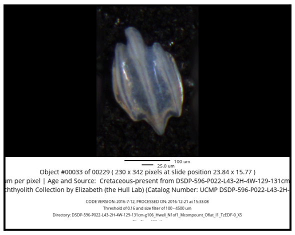 DSDP-596-P022-L43-2H-4W-129-131cm-g106_Hwell_N1of43_obj00033

<table>
<tr>
<td>3</td>
<td>Cusps opposite each other are similar, cusps perpendicular to each other are distinct</td>
<td>If a symmetrical crown has more than three cusps, and all cusps on a side are distinct from each other, rather than the denticle having only a distinct central cusp. Note that often the central cusp is longer and wider and the cusps on either side of the central cusp are the same size and slightly shorter, decreasing in size symmetrically closer to the edges.</td>
<td>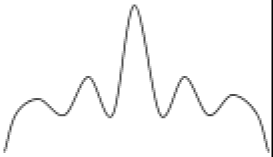</td>
<td>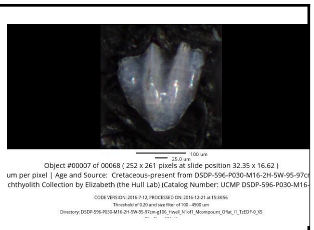

DSDP-596-P030-M16-2H-5W-95-97cm-g106_obj00007</td>
</tr>
<tr>
<td>4</td>
<td>Irregular</td>
<td>All cusps are distinct from each other.</td>
<td>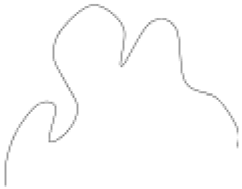</td>
<td>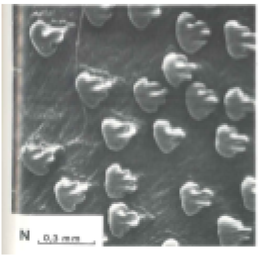

Galeocerdo cuvier, Reif, Pg. 227, Image N</td>
</tr>
</table>

Trait E3 Character Disparity Description:
Denticles with no cusps or only one cusp are coded as very different from denticles with multiple cusps. Denticles with cusps that are all similar, that have a distinct central cusp and that have some distinct and some similar cusps are all coded as somewhat different and equally different from each other. Denticles with irregular cusp similarities are coded as very different.
Weight in disparity calculation: 0.5

E3	1	2	3	4
1	0	1	1	3
2	1	0	1	3
3	1	1	0	3
4	3	3	3	0

**Trait E4: Maximum Cusp length**<br>The relative maximum cusp length is a ratio of the longest cusp length to total crown length; crown length is measured from anterior of the crown to the posterior point of the longest cusp.				
#	Name	Description	Illustration	Image
0	No cusps		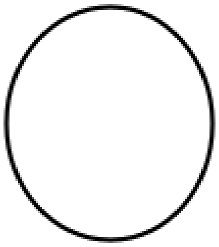	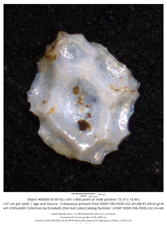<br>DSDP-596-P039-L52-2H-6W-81-83cm-g106_Hwell_N1of1_obj00030
1	Cusp < ¼ crown	Cusp length is less than ¼ of the total crown length.	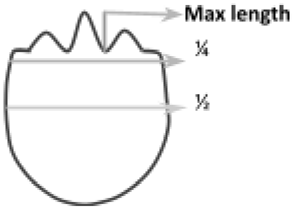	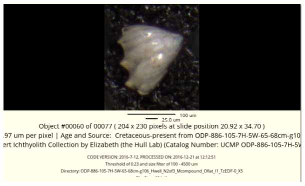<br>ODP-886-105-7H-5W-65-68cm-g106_obj00060_edf
2	¼ Crown < cusp <½ crown	Cusp length is more than ¼ of the total crown length but less than ½ of the total crown length.	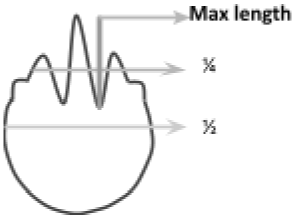	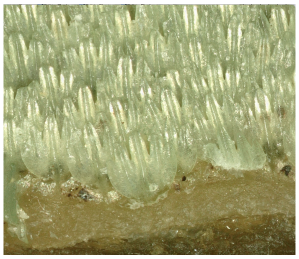<br>MCZS-394, *Sphyrna tiburo*, loc. F
3	Cusp > ½ crown	Cusp length is more than ½ of the total crown length.	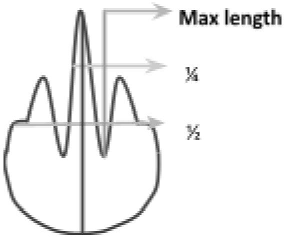	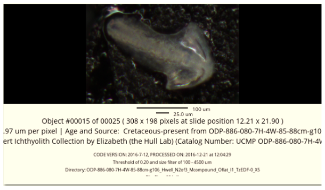<br>ODP-886-080-7H-4W-85-88cm-g106_obj00015_edf

Trait E4 Character Disparity Description:
Denticles with no cusps are coded as very different from denticles with cusps. Denticles with cusps are coded so that denticles with increased relative cusp length differ in one step increments from the previous length bin.
Weight in disparity calculation: 0.5.

E4	1	2	3
1	0	1	2
2	1	0	1
3	2	1	0

Trait F1: Ridge System
The ridge system is a broad categorization for ridge character traits and which primarily accounts for crown directionality (or lack thereof) by taking into account ridge orientation and ridge shape. Linear denticles have clear directionality, meaning all of their ridges run in the same direction. Crowns which have a geometric ridge system have ridges with a central branching pattern without a clear directionality. Note that in the case that a crown has an overall shape of spine (trait A1, state 1) but is smooth, it should be coded as having a smooth ridge system.

#	Name	Description	Illustration	Image
1	Smooth	Smooth denticles have no ridges and thus no ridge system	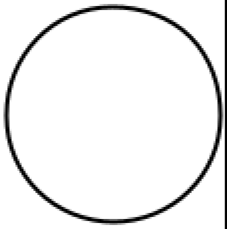	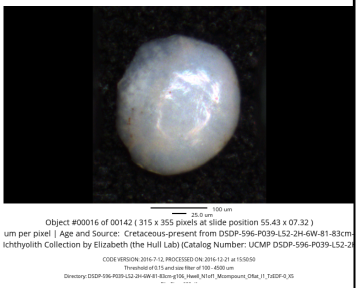 DSDP-596-P039-L52-2H-6W-81-83cm-g106_Hwell_N1of1_obj00016
2	Linear	Ridges give the crown a clear directionality and anterior/posterior and the ridges can only curve slightly in a maximum of 1 direction.	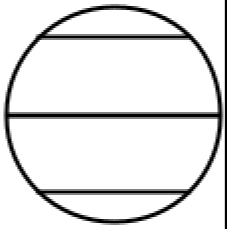	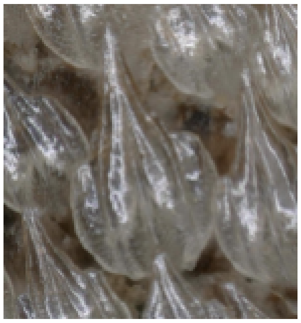 MCZ 36995, *Scyliorhinus retifer,* loc. H
3	Geometric	Ridges have a central branching pattern usually around a central ridge system shape and a dimple. The crown frequently does not have clear directionality	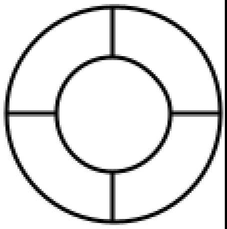	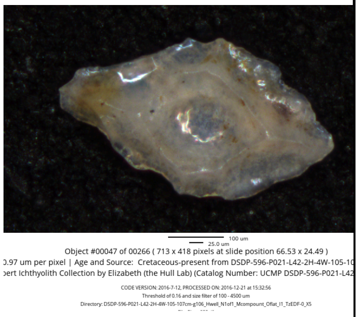 DSDP-596-P021-L42-2H-4W-105-107cm-g106_Hwell_N1of1_obj00047

4	Meandering	Not all denticles with meandering ridges are categorized as having a meandering ridge system. Crowns with a meandering ridge system are categorized as such due to a somewhat random display of meandering ridges with no other discernible ridge system pattern or directionality.	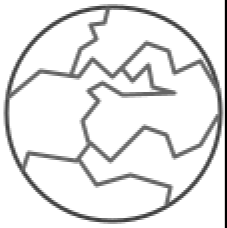	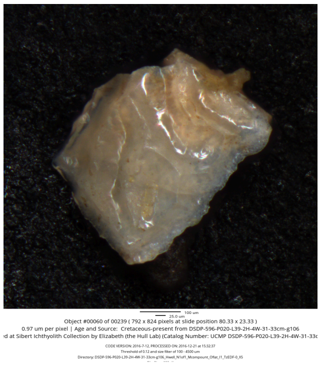 DSDP-596-P020-L39-2H-4W-31-33cm-g106_Hwell_N1of1_obj00060
5	Spine	Spines may or may not have ridges but when they do, these ridges usually converge at a higher vertical point.	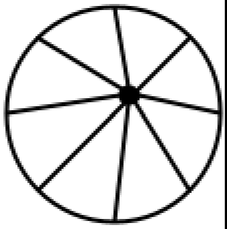	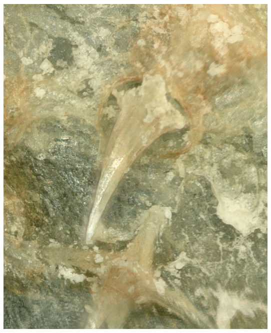 MCZ153780, *Etmopterus princeps*, loc. P
6	Branching	Ridges intersect and branch with a clear directionality meaning each subsequent branching ridge has a predictable direction often on either side of a central ridge.	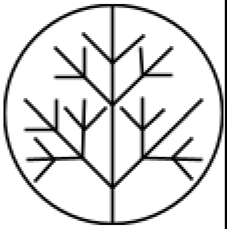	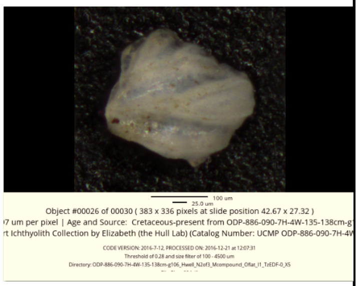 ODP-886-090-7H-4W-135-138cm-g106_obj00026_edf

Trait F1 Character Disparity Description:
Denticles with branching, geometric, and meandering ridge systems are coded as more similar to each other due to their irregular/unidirectional ridges and more different to denticles that have a spine, smooth, or linear ridge system. Spines and linear denticles are equally dissimilar to each other but more similar to each other than to branching, geometric, meandering, and smooth denticles. Smooth denticles are dissimilar to all other ridge systems as they are the only denticles with no ridges.
Weight in disparity calculation: 2.

F1	1	2	3	4	5	6
1	0	3	3	3	3	3
2	3	0	3	3	2	3
3	3	3	0	1	3	1
4	3	3	1	0	3	1
5	3	2	3	3	0	3
6	3	3	1	1	3	0

**Trait G1: Number of ridge segments**<br>Ridge segments are enumerated by counting the number of ridges on the crown of a denticle. In the case of linear denticles, these are usually parallel or pseudo-parallel. Where linear ridges branch, the segments are counted as individual ridge segments. For geometric denticles, whenever there is a vertex or pointed change in direction of a ridge, that is considered a separate 'segment'. In the case that a denticle has troughs, or long narrow valleys between ridges and other vertically higher crown traits, in addition to ridges, the trough is not counted in ridge number. In the case that a denticle has many troughs but no ridges the troughs are counted and described as ridges. Note that "ridge outgrowths" (see Trait G3) are not counted in the number of ridges here. Note that the number of ridge segments does not match the numeric coded character state value.			
#	Name	Illustration	Image
1	None	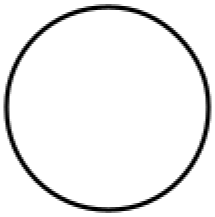	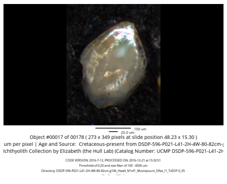<br>DSDP-596-P021-L41-2H-4W-80-82cm-g106_Hwell_N1of1_obj00017
2	One	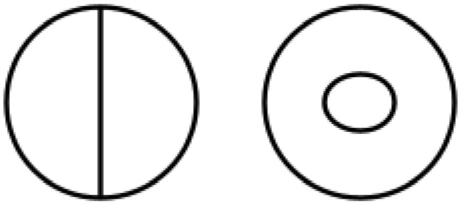	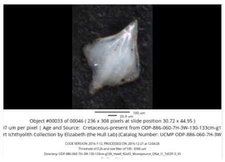<br>ODP-886-060-7H-3W-130-133cm-g106_obj00033_edf
3	Two	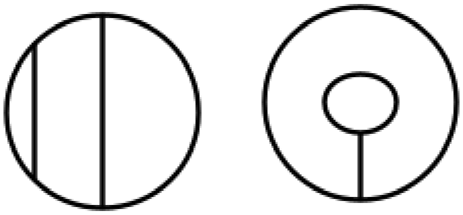	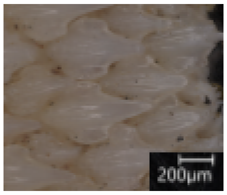<br>MCZ49, *Atelomycterus marmoratus*, loc. P
4	Three	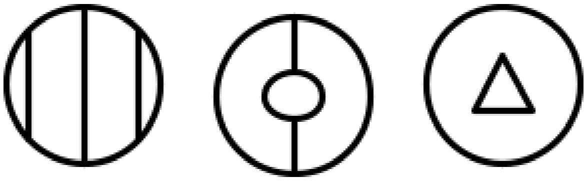	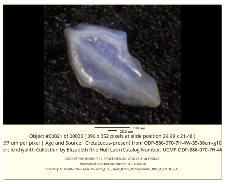<br>ODP-886-070-7H-4W-35-38cm-g106_obj00021_edf

5	Four	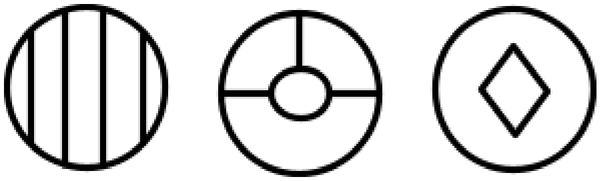	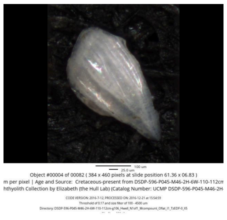 DSDP-596-P045-M46-2H-6W-110-112cm-g106_obj00004
6	Five	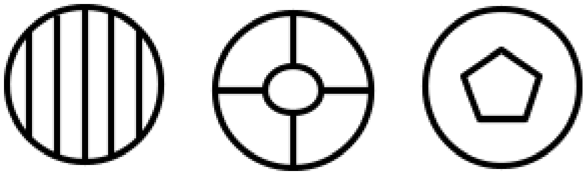	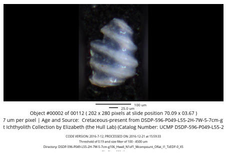 DSDP-596-P049-L55-2H-7W-5-7cm-g106_Hwell_N1of1_obj00002
7	Six-ten	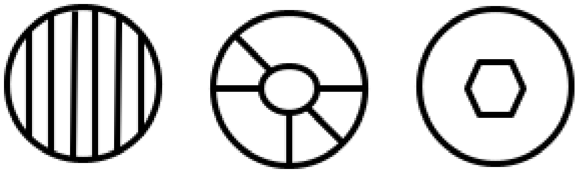	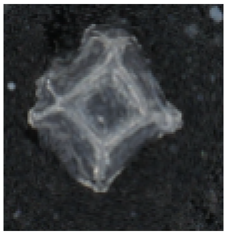 Isistius brasiliensis - MCZ 55447 - (loc. N)
8	Eleven or more	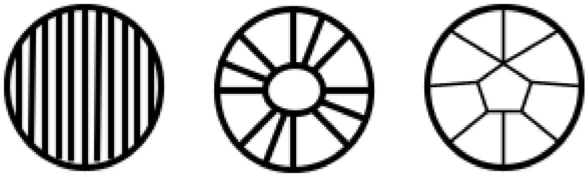	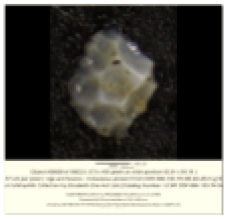 ODP-886-100-7H-5W-40-43cm-g106_obj00008_edf

Trait G1 Character Disparity Description:
Denticles with no ridge segments are very dissimilar to denticles with any ridge segments. With denticles with one through five segments are different linearly with denticles with more ridge segments being more different in proportionate steps to denticles with less segments. Denticles with six or more denticles are coded as more similar in their difference to other denticles with less segments as many types with this many ridges occasionally differ in number of segments by a few.
Weight in disparity calculation: 1.

G1	1	2	3	4	5	6	7	8
1	0	6	6	6	6	6	6	6
2	6	0	1	2	3	4	5	5
3	6	1	0	1	2	3	4	4
4	6	2	1	0	1	2	3	3
5	6	3	2	1	0	1	2	2
6	6	4	3	2	1	0	1	1
7	6	5	4	3	2	1	0	1
8	6	5	4	3	2	1	1	0

Trait G2: Number of independent ridges

Independent ridges are defined as ridges that do not intersect with any other ridges or curve in more than one direction. Where ridge segments diverge, branch, or change direction but are still connected, they are counted as one independent ridge. For geometric denticles, whenever there is a vertex or pointed change in direction of a ridge, it is only considered one independent ridge. In the case that a denticle has troughs, or long narrow channel segments in addition to ridges, the trough(s) is not counted in independent ridge number. In the case that a denticle has many troughs but no ridges the troughs are counted and described as ridges with each separate branch of troughs counted as an independent "ridge." Note that the number of independent ridges does not match the numeric coded character state value.

#	Name	Illustration	Image
1	None	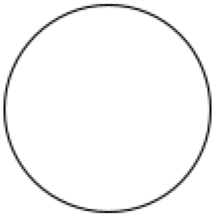	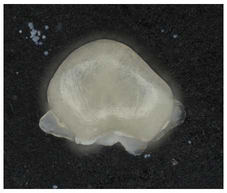 Squalus acanthias - MCZ 32341 (loc. D)
2	One independent ridge	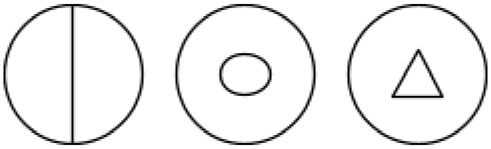	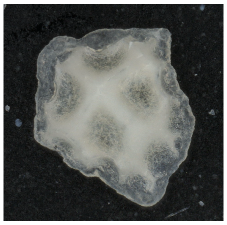 Chlamydoselachus anguineus - MCZ 34247 (loc. F)
3	Two independent ridges	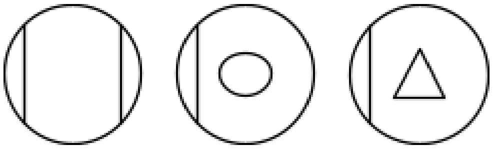	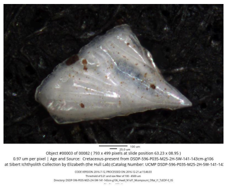 DSDP-596-P035-M25-2H-5W-141-143cm-g106_obj00003 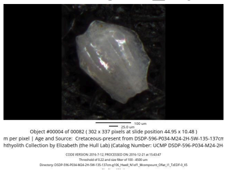 DSDP-596-P034-M24-2H-5W-135-137cm-g106_obj00004

4	Three independent ridges	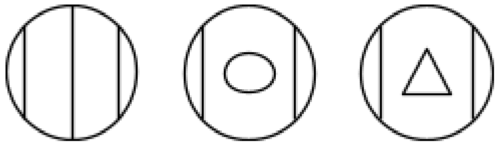	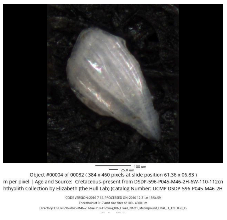
5	Four independent ridges	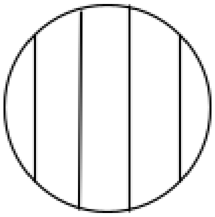	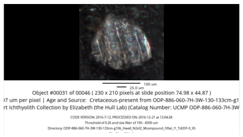 ODP-886-060-7H-3W-130-133cm-g106_obj00031_edf
6	Five independent ridges	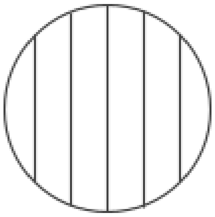	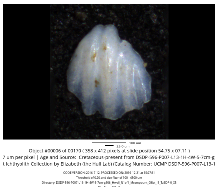 DSDP-596-P007-L13-1H-4W-5-7cm-g106_Hwell_N1of1_obj00006
7	Six or more independent ridges	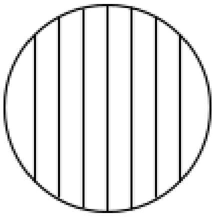	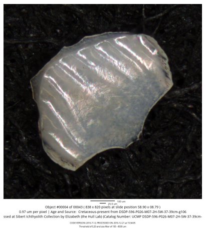 DSDP-596-P026-M07-2H-5W-37-39cm-g106_Hwell_N1of2_obj00004

Trait G2 Character Disparity Description:
Denticles with no ridges are coded as very different from denticles with any independent ridge segments. Denticles with only one independent ridge segment are coded as different from denticles with more than one independent ridge, this is because denticles with only one independent ridge include denticles with many intersecting/branching ridge segments. Denticles with two, three, four, and five ridges are coded as equally dissimilar while denticles with six or more independent ridges are coded as more different.
Weight in disparity calculation: 1.

G2	1	2	3	4	5	6	7
1	0	4	4	4	4	4	4
2	4	0	3	3	3	3	3
3	4	3	0	1	1	1	2
4	4	3	1	0	1	1	2
5	4	3	1	1	0	1	2
6	4	3	1	1	1	0	2
7	4	3	2	2	2	2	0

Trait G3: Ridge Outgrowths
Ridge outgrowths are defined as small ridge-like extensions on the anterior edges of some linear denticles. These outgrowths appear distinct from the central main crown in shape and length. Ridge outgrowths are not counted in the total number of ridges or number of independent ridges on a denticle (trait G1 and G2). Note that the number of ridge outgrowths does not match the numeric coded character state value.

#	Name	Description	Illustration	Image
0	No ridges or too broken to code		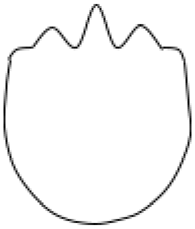	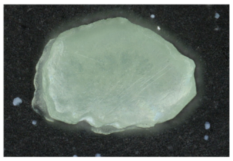 *Rhizoprionodon terraenovae* - MCZ S-432 (loc. A)
1	No ridge outgrowths		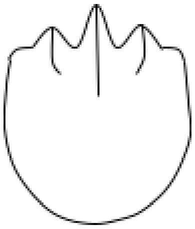	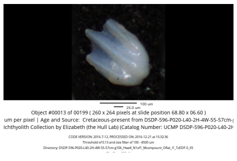 DSDP-596-P020-L40-2H-4W-55-57cm-g106_Hwell_N1of1_obj00013
2	One ridge outgrowth	One ridge outgrowth on one lateral edge of the main section of the crown.	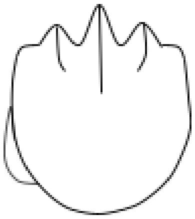	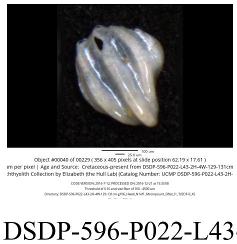 DSDP-596-P022-L43-2H-4W-129-131cm-g106_Hwell_N1of25_obj00040

3	Two ridge outgrowths	Two ridge outgrowths on either lateral edge of the main section of the crown.	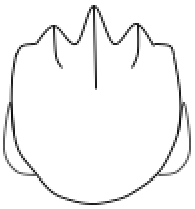	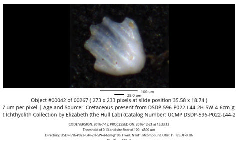<br>DSDP-596-P022-L44-2H-5W-4-6cm-g106_Hwell_N1of1_obj00042

Trait G3 Character Disparity Description:
Denticles with no ridges are coded as more different from denticles with ridges and ridge outgrowths. Denticles with no ridge outgrowths are coded as more different than denticles with one or two ridge outgrowths and denticles with one or two ridge outgrowths are coded as less dissimilar to each other.
Weight in disparity calculation: 1.

G3	1	2	3
1	0	2	2
2	2	0	1
3	2	1	0

**Trait H1: Ridge Orientation**<br>Ridges with a linear system can be further characterized by their ridges' orientation to one another, as diverging or converging from the anterior, branching, or truly parallel, while geometric denticles always have a branching pattern and spine denticles always have radial ridges when ridges are present. For geometric denticles, use character state 5, ridges intersect/branch unless there is an obvious better fit.				
#	Name	Description	Illustration	Image
0	Only one ridge / no ridges		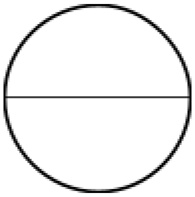	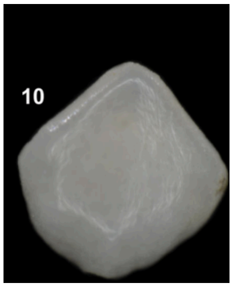<br>Dillon, E. M., Norris, R. D., & Dea, A. O. (2017).
1	Parallel	All ridges run parallel to each other. Many linear denticles may appear to have slight divergence from the anterior but are parallel along the majority of the crown. These denticles should be coded with a parallel ridge orientation.	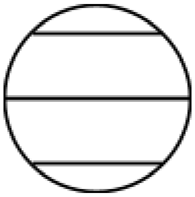	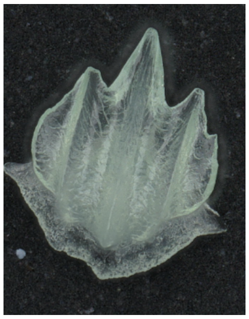<br>Rhizoprionodon terraenovae - MCZ S-432 (Loc. Z)
2	Converges from anterior (base)	Ridges converge from the anterior towards the posterior.	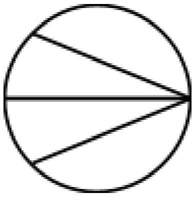	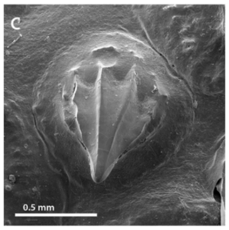<br>Squatina occulta Vaz, D. F., & De Carvalho, M. R. (2013)

3	Diverges from anterior (base)	Ridges diverge from the anterior towards the posterior.	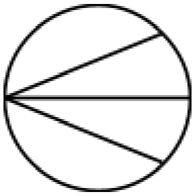	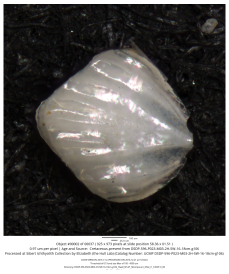 DSDP-596-P023-M03-2H-5W-16-18cm-g106_Hwell_N1of1_obj00002
4	Diverges then converges	Ridges converge from the anterior towards the posterior.	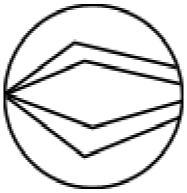	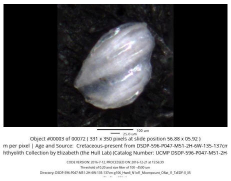 DSDP-596-P047-M51-2H-6W-135-137cm-g106_obj00003
5	Ridges intesect/branch	This includes denticles with geometric, branching, and some meandering ridge systems.	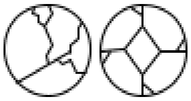	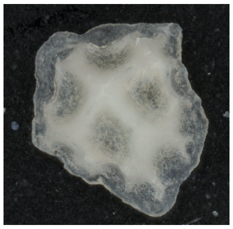 *Chlamydoselachus anguineus* - MCZ 34247 (loc. F)
6	Apex Radial	Often spines.	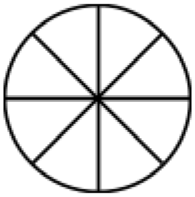	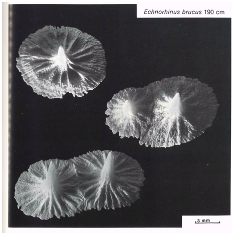 *Echinorhinus brucus,* Reif, Pg. 135
7	Irregular or No discernable anterior/posterior		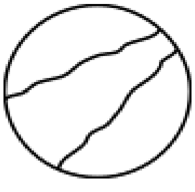	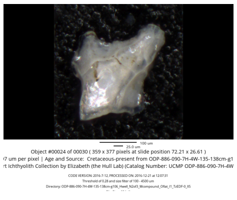 ODP-886-090-7H-4W-1

				35-138cm-g106_obj00024_edf
8	Multiple ridge orientations		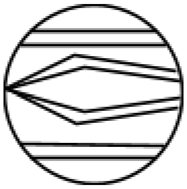	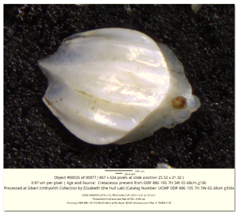 ODP-886-105-7H-5W-65-68cm-g106_obj00026_edf

Trait H1 Character Disparity Description: Denticles with only one or no ridges are coded as very dissimilar to all other denticles. Denticles with parallel, or converging/diverging from base ridge orientations are coded as more similar to each other than other ridge orientations including denticles which have ridges that diverge and then converge. Denticles with apex/radial or intersecting or branching ridge orientations are coded as distinct but more similar than other orientations and very dissimilar to other ridge orientations.
Weight in disparity calculation: 0.5

H1	1	2	3	4	5	6	7	8
**1**	0	1	1	2	3	3	3	1
**2**	1	0	1	2	3	3	3	1
**3**	1	1	0	2	3	3	3	1
**4**	2	2	2	0	3	3	3	1
**5**	3	3	3	3	0	2	3	1
**6**	3	3	3	3	2	0	3	1
**7**	3	3	3	3	3	3	0	1
**8**	1	1	1	1	1	1	1	0

Trait I1: Central Ridge(s) or trough Disparity
The central ridge is defined as the ridge (or ridges for crowns with a central trough) in the center of all other ridges on the crown, or in the case that the crown has only one ridge, may be the ridge which is at the center of the crown. The central ridge usually runs from the anterior to the posterior bifurcating the crown. It is sometimes, though not always, distinct from the other ridges in shape and size.

#	Name	Description	Illustration	Image
0	No ridges		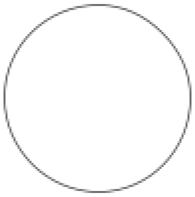	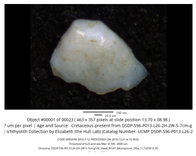 DSDP-596-P013-L26-2H-2W-5-7cm-g106_Hwell_N1of1_obj00001
1	No central ridge	Ridges present on the crown but there is either an even number of ridges with no central ridge or a ridge orientation without a central ridge (i.e. many geometric denticles have a central ridge system shape (trait K1) but may not have a singular central ridge).	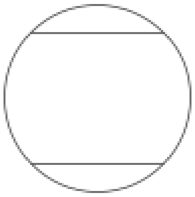	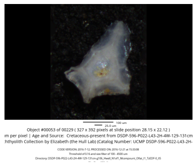 DSDP-596-P022-L43-2H-4W-129-131cm-g106_Hwell_N1of20_obj00053
2	Only one ridge	When a denticle has only one ridge, it often runs down the center of the crown from anterior to posterior.	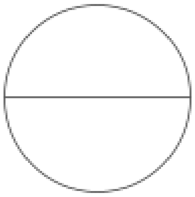	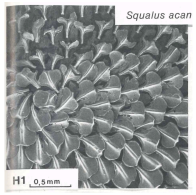 *Squalus acanthias,* Reif, Pg. 114, Fig. H1

3	Same as other ridges	The central ridge is the same shape and size as the other ridges.	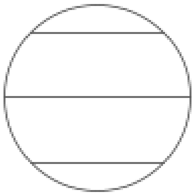	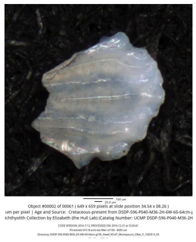<br>DSDP-596-P040-M36-2H-6W-60-64cm-g106_obj00002
4	Distinct shape/size from other ridges	The central ridge is a different shape and/or size than the other ridges.	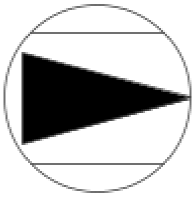	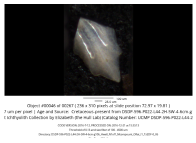<br>DSDP-596-P022-L44-2H-5W-4-6cm-g106_Hwell_N1of1_obj00046<br>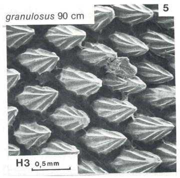<br><br>*Centrophorus granulosus,* Reif, Pg. 112, Fig. H3

Trait I1 Character Disparity Description:
Denticles with no ridges are coded as very different from other denticles. Denticles with no central ridge are coded as more similar to denticles with only one ridge and very different from denticles with a central ridge that is the same as other ridges or distinct. Denticles with only one ridge are coded as more similar to denticles with central ridges (both that are similar ridges and distinct central ridges) because often denticles with only one ridge are in the center of the crown (though not in the center of other ridges). Denticles with central ridges that are distinct are coded as different from denticles with similar central ridges but less distinct than these central ridged denticles from other orientations with no central ridges.

I1	1	2	3	4
1	0	2	3	3
2	2	0	1	1
3	3	1	0	2
4	3	1	2	0

Weight in disparity calculation: 0.5.

Trait I2: Central Ridge Directionality
Central Ridge directionality is defined by the edges of the central ridge (or ridges for crowns with a central trough), independent of ridge orientation or the central ridge relative to other ridges.

#	Name	Description	Illustration	Image
0	None	No central ridge.	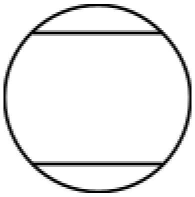	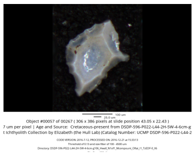 DSDP-596-P022-L44-2H-5W-4-6cm-g106_Hwell_N1of1_obj00057
1	Straight	The central ridge runs in a single direction, usually anterior to posterior and has parallel sides.	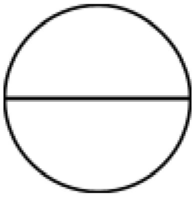	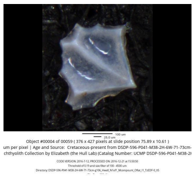 DSDP-596-P041-M38-2H-6W-71-73cm-g106_obj00004
2	Curved	The central ridge curves, changing direction at a single inflection point.	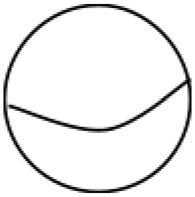	N/A
3	Meandering	An irregularly shaped ridge with uneven and/or inconsistent edges which may have a general direction but curves or deviates direction slightly along its length.	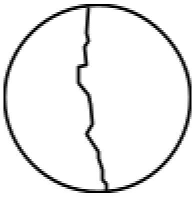	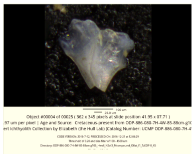 ODP-886-080-7H-4W-85-88cm-g106_Hwell_N2of3_Mcompound_Oflat_I1_TzEDF-0_X5

Trait I2 Character Disparity Description:
Denticles with straight and curved are coded as being more similar to each other than denticles with meandering central ridge directionalities
Weight in disparity calculation: 0.5.

I2	1	2	3
1	0	1	2
2	1	0	2
3	2	2	0

Trait I3: Central Ridge Width
Width is defined by the edges of the independent ridge(s) relative to the anterior of the crown.

#	Name	Description	Illustration	Image
0	None	No central ridge.	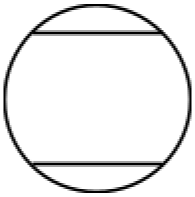	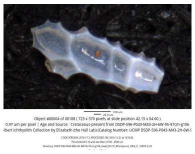 DSDP-596-P043-M43-2H-6W-95-97cm-g106_obj00004
1	Parallel	Consistent width along length.	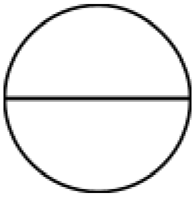	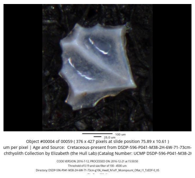 DSDP-596-P041-M38-2H-6W-71-73cm-g106_obj00004
2	Triangular widening from anterior	The central ridge is triangular with an anterior vertex which widens to a flat edge at the posterior.	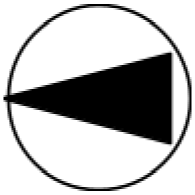	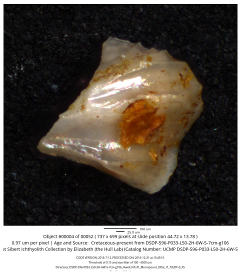 DSDP-596-P033-L50-2H-6W-5-7cm-g106_Hwell_N1of1_obj00004
3	Triangular thinning from anterior	The central ridge is triangular with an anterior flat edge which thins to a posterior vertex.	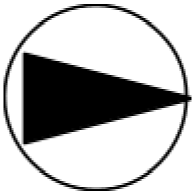	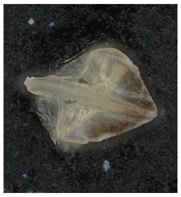 Squalus acanthias, MCZ 32341

4	Diamond-like	Widening and then thinning from the anterior.	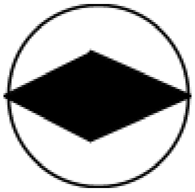	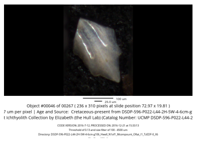 DSDP-596-P022-L44-2H-5W-4-6cm-g106_Hwell_N1of1_obj00046
5	Creates a Central Trough	Two central ridges converge at the anterior and/or posterior of the crown creating a long narrow valley in between.	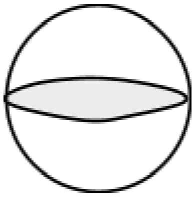	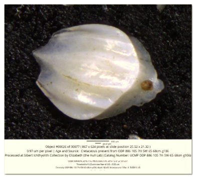 ODP-886-105-7H-5W-65-68cm-g106_obj00026_edf
6	Irregular		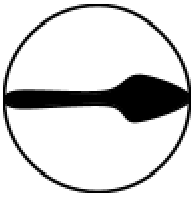	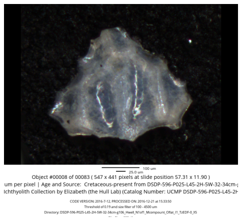 DSDP-596-P025-L45-2H-5W-32-34cm-g106_Hwell_N1of1_obj00008

Trait I3 Character Disparity Description:
Denticles with a straight central ridge are coded as different from all other central ridge widths based on their level of difference from this straight state. Triangular and diamond shaped central ridges are coded as equally similar/dissimilar from straight ridges while troughs and irregular widths are coded as the most dissimilar from straight ridges.
Weight in disparity calculation: 0.5

I3	1	2	3	4	5	6
1	0	1	1	1	2	2
2	1	0	1	1	2	2
3	1	1	0	1	2	2
4	1	1	1	0	2	2
5	2	2	2	2	0	1
6	2	2	2	2	1	0

**Trait J1: Non-Central Ridge Directionality**<br>Non-central ridge directionality is defined by the edges of the non central ridge, independent of ridge orientation or the non-central ridge relative to any central ridge. Many denticles with a geometric ridge system have a combination of curved ridges and straight ridges.				
#	Name	Description	Illustration	Image
0	None/only central ridge	There are no ridges or there is only a central ridge.	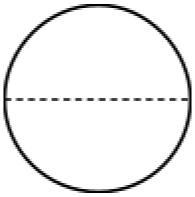	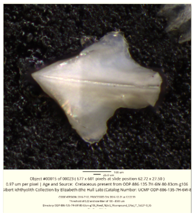<br>ODP-886-135-7H-6W-80-83cm-g106_Hwell_N2of2_Mcompound_Oflat_I1_TzEDF-0_X5
1	Straight	Each ridge segment runs in a single direction.	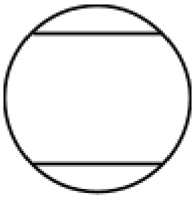<br>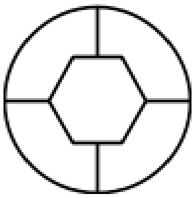	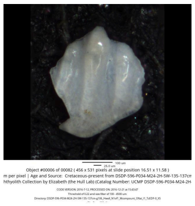<br>DSDP-596-P034-M24-2H-5W-135-137cm-g106_obj00006<br>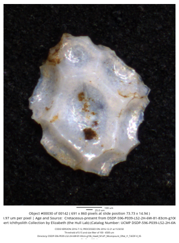<br>DSDP-596-P039-L52-2H-6W-81-83cm-g106_Hwell_N1of1_obj00030
2	Concave Curved	Ridges are curved with a concave angle relative to the center of the crown.	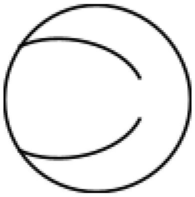	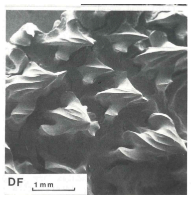<br>*Oxynotus centrina*,

				Reif, Pg. 120, Fig. DF
3	Convex Curved	Ridges are curved with a convex angle relative to the center of the crown.	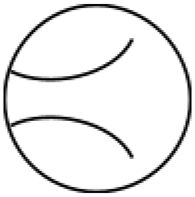	N/A
4	Meandering	Irregularly shaped ridges with uneven and/or inconsistent edges which have a general direction but which curve or deviate in direction slightly along their length.	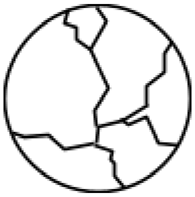	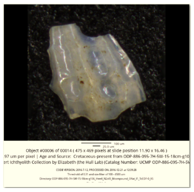 ODP-886-095-7H-5W-15-18cm-g106_obj00006_edf
5	Combination	Multiple or Irregular shapes.	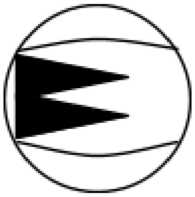	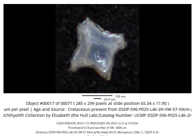 DSDP-596-P025-L46-2H-5W-57-59cm-g106_Hwell_N1of1_obj00017

Trait J1 Character Disparity Description:
Denticles with straight non central ridges are coded as equally different from both curved directionalities which are more similar to each other. Denticles with meandering or combinations of non-central ridge directionalities are coded as the most distinct from other ridge directionalities but more similar to each other.
Weight in disparity calculation: 0.5

J1	1	2	3	4	5
1	0	2	2	3	3
2	2	0	1	3	3
3	2	1	0	3	3
4	3	3	3	0	1
5	3	3	3	1	0

**Trait J2: Non-Central Ridge Width**<br>Width is defined by the edges of the independent ridge(s) relative to the anterior of the crown.				
#	Name	Description	Illustration	Image
0	None/only central ridge	No ridges or only a central ridge.	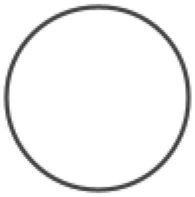	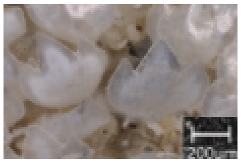<br>MCZ793, *Asymbolus analis*, loc. F
1	Parallel	Consistent width along length.	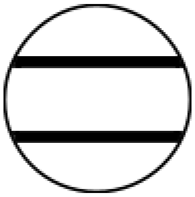	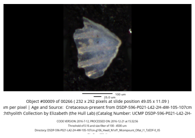<br>DSDP-596-P021-L42-2H-4W-105-107cm-g106_Hwell_N1of1_obj00009
2	Triangular widening from anterior	The non-central ridges are triangular with an anterior vertex which widens to a flat edge at the posterior.	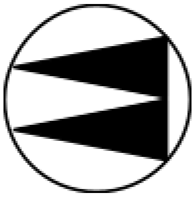	N/A
3	Triangular thinning from anterior	The central ridge is triangular with an anterior flat edge which thins to a posterior vertex.	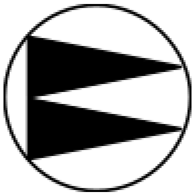	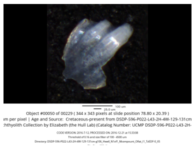<br>DSDP-596-P022-L43-2H-4W-129-131cm-g106_Hwell_N1of30_obj00050
4	Diamond-like	Widening and then thinning from anterior.	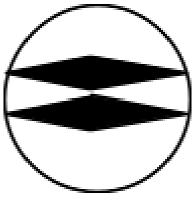	N/A

5	Create Troughs	In pairs, non-central ridges converge at the anterior and/or posterior of the crown creating a long narrow valley in between.	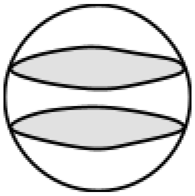	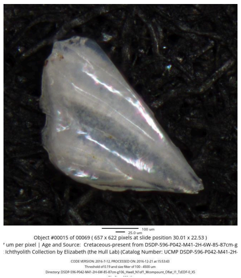 DSDP-596-P042-M41-2H-6W-85-87cm-g106_obj00015
6	Irregular/ Combination		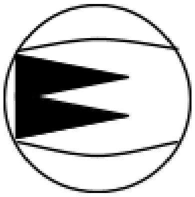	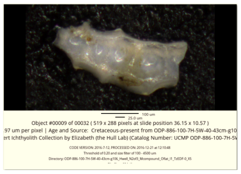 ODP-886-100-7H-5W-40-43cm-g106_obj00009_edf

Trait J2 Character Disparity Description:
Denticles with straight non central ridges are coded as different from all other ridge widths based on their level of difference from this straight state. Triangular and diamond shaped non central ridges are coded as equally similar/dissimilar from straight ridges while troughs and irregular or combination widths are coded as the most dissimilar from straight ridges and equally similar/dissimilar to each other.
Weight in disparity calculation: .5

J2	1	2	3	4	5	6
1	0	1	1	1	2	2
2	1	0	1	1	2	2
3	1	1	0	1	2	2
4	1	1	1	0	2	2
5	2	2	2	2	0	1
6	2	2	2	2	1	0

**Trait K1: Central ridge system shape**<br>Denticles with a geometric ridge system often have a central branching ridge orientation with ridges at the center which define the edges of this enclosed polygon or rounded shape, called the central ridge system shape. Other ridges generally branch and radiate from the central ridge system shape's edges.				
#	Name	Description	Illustration	Image
0	No Ridges		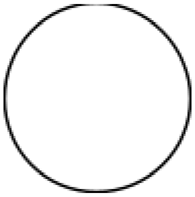	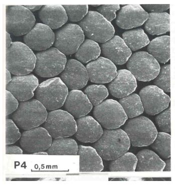<br>Squatina japonica, Reif, Pg. 128, fig. P4
1	Ridges present but no central shape observed	For example, linear denticles fit this description.	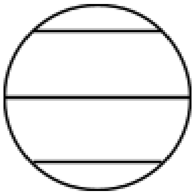	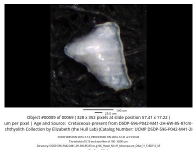<br>DSDP-596-P042-M41-2H-6W-85-87 cm-g106_obj00009
2	Circular/oval	Central shape is round and curved with no vertices or straight edges.	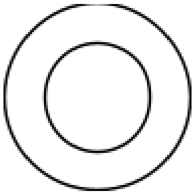	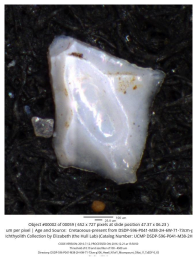<br>DSDP-596-P041-M38-2H-6W-71-73 cm-g106_obj00002
3	Triangular	The central shape has three edges which converge at three vertices.	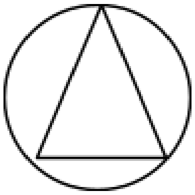	N/A

4	Quadrilateral	The central shape has four edges which converge at four vertices.	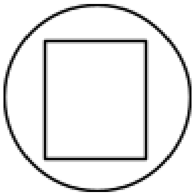	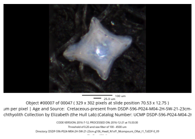 DSDP-596-P024-M04-2H-5W-21-23cm-g106_Hwell_N1of1_obj00007
5	Pentagonal	The central shape has five edges which converge at five vertices.	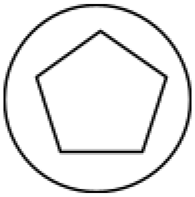	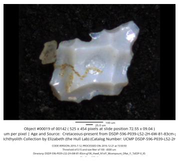 DSDP-596-P039-L52-2H-6W-81-83cm-g106_Hwell_N1of1_obj00019
6	Hexagon	The central shape has six edges which converge at six vertices.	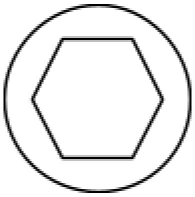	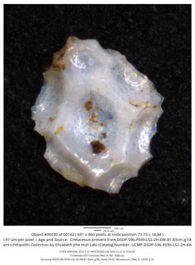 DSDP-596-P039-L52-2H-6W-81-83cm-g106_Hwell_N1of1_obj00030
7	Heptagon	The central shape has seven edges which converge at seven vertices.	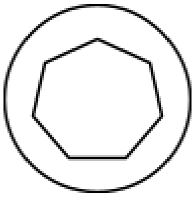	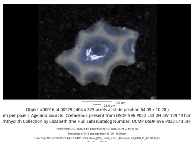 DSDP-596-P022-L43-2H-4W-129-131cm-g106_Hwell_N1of39_obj00016

8	Mound	The central shape has any number of edges and converges at a central mound, or plateau, or a raised nub.	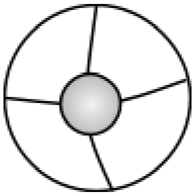	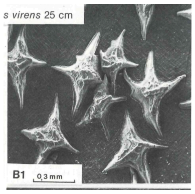<br>*Etmopterus virens*, Reif, Pg. 110, Fig. B1
9	Octagon	The central shape has eight edges which converge at eight vertices.	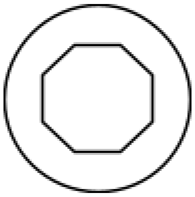	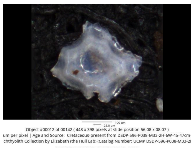<br>DSDP-596-P038-M33-2H-6W-45-47cm-g106_obj00012
10	Multiple	Multiple central ridge system shapes.	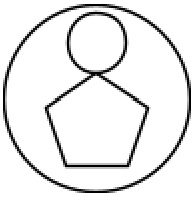	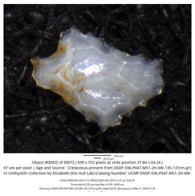<br>DSDP-596-P047-M51-2H-6W-135-137cm-g106_obj00002
11	Irregular/other	Shape is considerably irregular and does not fall into other categories.	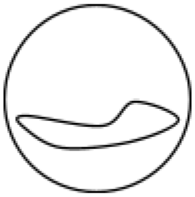	N/A

Trait K1 Character Disparity Description:
Denticles with a circular/oval, triangular, quadrilateral, pentagonal, hexagonal, heptagonal, and octagonal central ridge system shape are all composed of enclosed geometric depressions with a dimple in the center and are thus coded as fairly similar in comparison to a mound which has a significantly different composition which is not necessarily associated with a geometric shape or dimple. Additionally, denticles with ridges with an observed central ridge system shape are coded as very different from denticles with ridges that do not have any central shape. Denticles with no ridges are likewise coded as equally different from all other ridged denticles and denticles with multiple central shapes observed are also coded as significantly different from other denticles with a single central ridge system shape.
Weight in disparity calculation: 1.

K1	1	2	3	4	5	6	7	8	9	10	11
**1**	0	3	3	3	3	3	3	3	3	3	4
**2**	3	0	1	1	1	1	1	5	2	4	4
**3**	3	1	0	1	1	1	1	5	2	4	4
**4**	3	1	1	0	1	1	1	5	2	4	4
**5**	3	1	1	1	0	1	1	5	2	4	4
**6**	3	1	1	1	1	0	1	5	2	4	4
**7**	3	1	1	1	1	1	0	5	2	4	4
**8**	3	5	5	5	5	5	5	0	2	4	4
**9**	3	2	2	2	2	2	2	2	0	4	4
**10**	3	4	4	4	4	4	4	4	4	0	4
**11**	4	4	4	4	4	4	4	4	4	4	0

**Trait K2: Central Ridge System Shape Planes of Symmetry**<br>The central ridge system shape planes of symmetry is defined as the number of axes which can bifurcate the central ridge system shape.				
#	Name	Description	Illustration	Image
0	No central ridge system shape		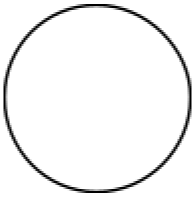	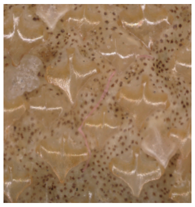<br>Heterodontus francisci - MCZ 392
1	No symmetry	Central shape present but has no planes symmetry.	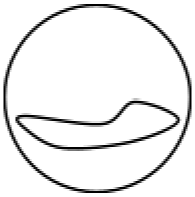	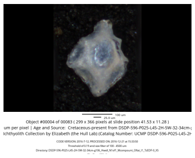<br>DSDP-596-P025-L45-2H-5W-32-34cm-g106_Hwell_N1of1_obj00004
2	One	Central shape has one plane of symmetry.	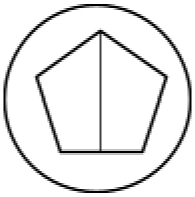	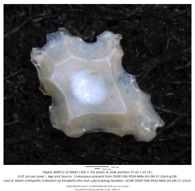<br>DSDP-596-P024-M04-2H-5W-21-23cm-g106_Hwell_N1of1_obj00012
3	Two	Central shape has two planes of symmetry.	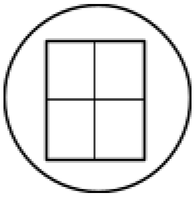	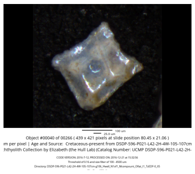<br>DSDP-596-P021-L42-2H-4W-105-107cm-g106_Hwell_N1of1_obj00040

4	Radial	Central shape has radial symmetry.	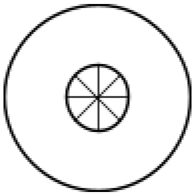	N/A

Trait K2 Character Disparity Description: Denticles with no central shape are coded as very different from denticles with a central ridge system shape. Denticles with a central ridge system shape with no planes of symmetry are coded as significantly different from those with planes of symmetry and even more different from denticles with central ridge system shapes with radial symmetry. Denticles with central ridge system shapes with one and two planes of symmetry are coded as being less different from each other and less different to those with radial symmetry than those with no central shape or those with no planes of symmetry.

K2	1	2	3	4
1	0	2	2	3
2	2	0	1	3
3	2	1	0	3
4	3	3	3	0

Weight in disparity calculation: 0.5.

Trait L1: Ridge length

The lengths of ridges vary in relation to the crown length and compared to other ridges. Linear denticles typically have ridges which stretch from the anterior to the posterior edges of the crown, while geometric denticles with exclusively branching ridges tend to include ridges which do not reach the edges of the crown and rather begin and terminate at different areas on the crown.

#	Name	Description	Illustration	Image
1	No ridges		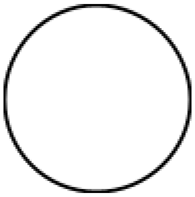	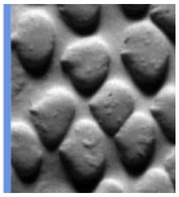 Alopias vulpinus, Gabler-Smith, M. K., Wainwright, D. K., Wong, G. A., & Lauder, G. V. (2021)
2	Ridge length=lateral length	The ridge(s) run from the anterior edge to the posterior edge of the crown. This does not mean that all ridges extend the longest axis of the crown, rather that the ridge runs from edge to edge (even if the side ridges and edges are shorter than the center of the crown and center ridges).	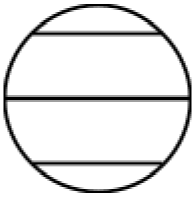	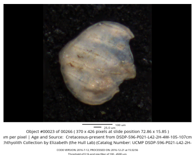 DSDP-596-P021-L42-2H-4W-105-107cm-g106_Hwell_N1of1_obj00023
3	Ridge ends mid crown/ Ridge begins mid crown	The ridge(s) begin at the anterior edge but terminate before reaching the posterior edge or begin mid-crown and terminate at the posterior edge.	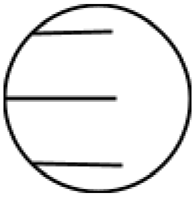	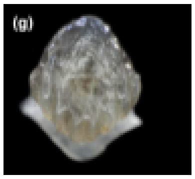 Dillon, E. M. (2022)

4	Ridge begins and ends mid crown	The ridge(s) do not reach the posterior or anterior edges.	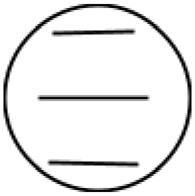	N/A
5	Includes ridges described by a combination of 1,2, 3,	Different ridge(s) have differing lengths relative to the size of the crown and anterior and posterior edges.	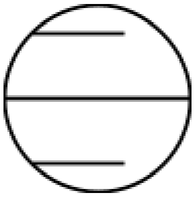	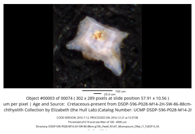 DSDP-596-P028-M14-2H-5W-86-88cm-g106_Hwell_N1of1_obj00003

Trait L1 Character Disparity Description:
Denticles with no ridges are coded as very distinct from denticles with ridges. Denticles with equal crown length and ridge length, ridges that end or begin mid crown, and denticles with ridges that both end and begin mid crown are all coded as less distinct from each other. Denticles with ridges that have a combination of the previously described ridge length character states are coded as the most dissimilar to all other ridge lengths.
Weight in disparity calculation: 0.5.

L1	1	2	3	4	5
1	0	2	2	2	2
2	2	0	1	1	3
3	2	1	0	1	3
4	2	1	1	0	3
5	3	3	3	3	0

Trait L2: Ridge Definition
Ridges differ in their level of definition or visibility. They can be visible with deep troughs or shallow with nearly invisible ridge patterns. Some denticles display ridges of varying definition. This can also be thought of as smoothness or rugosity of the denticle's crown.

#	Name	Description	Illustration	Image
0	No Ridges		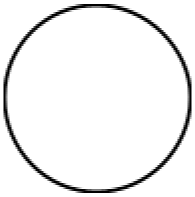	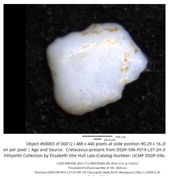 DSDP-596-P019-L37-2H-3W-130-132cm-g106_Hwell_N1of1_obj00003
1	Not Clearly Defined	Ridge(s) are shallow, barely raised from the crown, making them difficult to see.	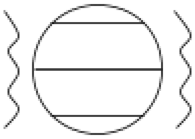	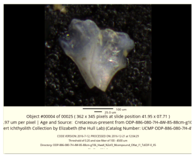 ODP-886-090-7H-4W-135-138cm-g106_obj00004_edf
2	Clearly defined on part of the crown but gets shallower and does not extend to full length of the crown	Usually this occurs when ridge(s) are taller and more defined at the anterior but become shallower and less clearly visible at the posterior.	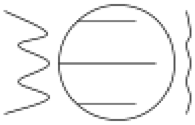	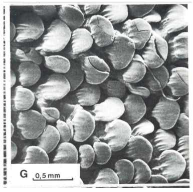 *Alopias superciliosus*, Reif, Pg. 143, Fig. G
3	Clearly Defined	Ridges are tall and clearly visible on the entire crown.	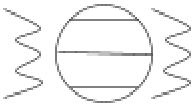	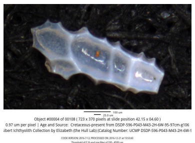 DSDP-596-P043-M43-2H-6W-95-97cm-g106_obj00004

4	Only the central ridge is clearly defined	Side ridges are shallow and difficult to see and the central ridge is taller and clearly visible.	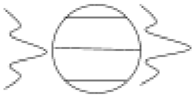	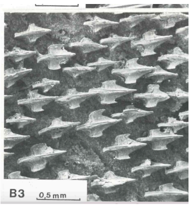<br>*Oxynotus centrina,* Reif, Pg. 120, Fig. B3

Trait L2 Character Disparity Description:
Denticles with ridges are coded as very different from denticles without ridges. Denticles with ridges that are not clearly defined at all are coded as very different from denticles with some clear ridges and even more different to denticles with completely clearly defined denticles. Denticles with only a defined central ridge and denticles with ridges that are clear on part of the crown but become less defined are coded as more similar to each other than the other trait states.
Weight in disparity calculation: 0.5.

L2	1	2	3	4
1	0	2	3	2
2	2	0	2	1
3	3	2	0	2
4	2	1	2	0

Trait L3: Relative Ridge Heights

Heights can differ among the ridges on a denticle, if there is height disparity the tallest ridge is usually towards the center of a denticle with a linear system

#	Name	Description	Illustration	Image
0	None		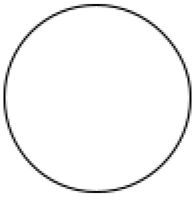	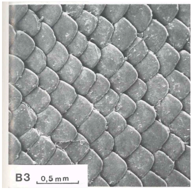 *Chiloscyllium plagiosum,* Reif, Pg. 130. Fig. B3
1	Only one ridge	Only one ridge and thus has no relative height compared to other ridges.	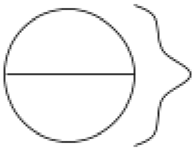	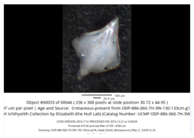 ODP-886-060-7H-3W-130-133cm-g106_obj00033_edf
2	Equal	All ridges appear to have equal ridge heights.	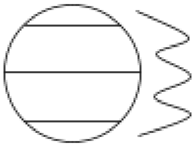	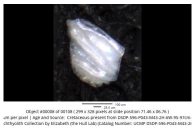 DSDP-596-P043-M44-2H-6W-101-103cm-g106_obj00008
3	Variable	Ridges have variable heights with some ridges which are taller than others.	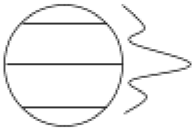	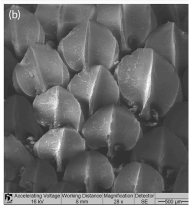 *Scymnodon macracanthus*, B. Vaz, D. F. (2021), Fig. 6.b

Trait L3 Character Disparity Description:
Denticles with no ridges and only one ridge are coded as very different from denticles with multiple ridges. Denticles with equal ridge heights and variable ridge heights are coded as equally different from each other.
Weight in disparity calculation: 0.5.

**L3**	**1**	**2**	**3**
**1**	0	2	2
**2**	2	0	1
**3**	2	1	0

**Trait L4: Ridge/Trough Angularity**<br>Ridge vertical profiles (or cross sections) can have different shapes, some are rounded while others are triangular. Some denticles display a range of ridge angularity.			
#	Name	Illustration	Image
0	No ridges	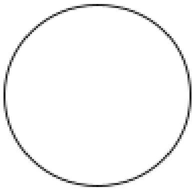	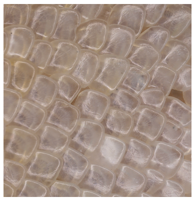<br>Hemiscyllium ocellatum - MCZ 155795 (loc. A)
1	Ridge profiles or trough are rounded	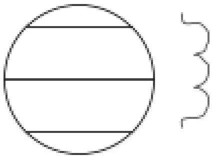	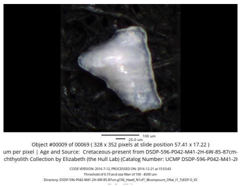<br>DSDP-596-P042-M41-2H-6W-85-87cm-g106_obj00009
2	Ridge profiles or troughs are triangular	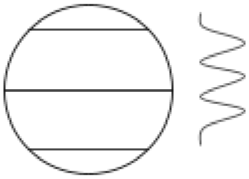	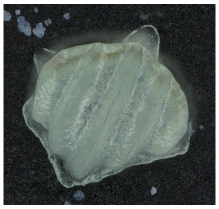<br>*Rhizoprionodon terraenovae* - MCZ S-432 (loc. O)
3	Ridge profiles or troughs are funnel shaped (rounded at the anterior and thin to a skinnier point at the posterior)	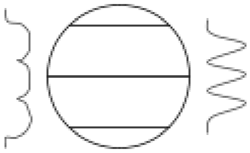	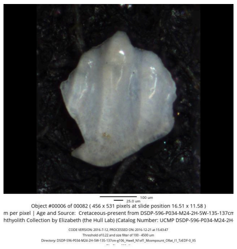<br>DSDP-596-P034-M24-2H-5W-135-137cm-g106_obj00006

4	Ridge profiles or troughs are variable or described by another shape	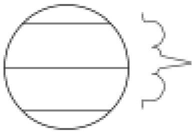	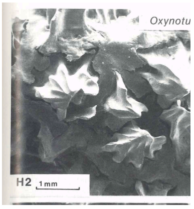<br>*Oxynotus centrina,* Reif, Pg. 120, Fig. H2
5	Ridge profiles or troughs are rectangular	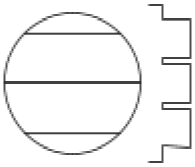	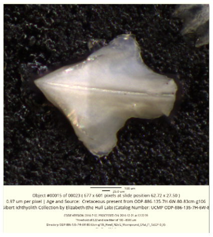<br>ODP-886-135-7H-6W-80-83 cm-g106_obj00015_edf

Trait L4 Character Disparity Description:
Denticles with no ridges and denticles with ridges or troughs which have variable ridge profiles are both coded as very different from all other ridge/trough profiles which are all coded as equally different from each other.
Weight in disparity calculation: 0.5.

L4	1	2	3	4	5
1	0	1	1	2	1
2	1	0	1	2	1
3	1	1	0	2	1
4	2	2	2	0	2
5	1	1	1	2	0

**Trait M1: Number of depressions**<br>Number of depressions: Depressions are defined as low areas or impressions on the crown. These occur most often in geometric denticles with ridges that have a central branching pattern.			
#	Name	Illustration	Image
1	None	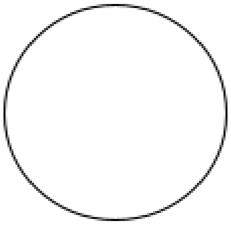	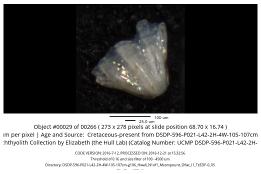<br>DSDP-596-P021-L42-2H-4W-105-107cm-g106_Hwell_N1of1_obj00029
2	One depression	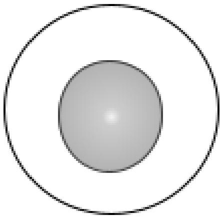	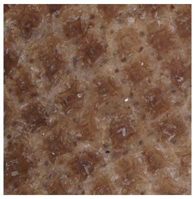<br>*Isistius brasiliensis* - MCZ 55447 (loc. V)
3	Two depressions	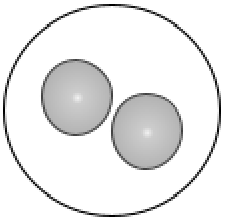	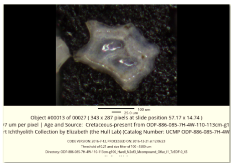<br>ODP-886-085-7H-4W-110-113cm-g106_obj00013_edf
4	Three depressions	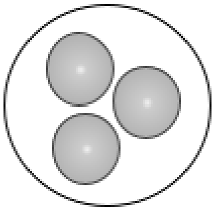	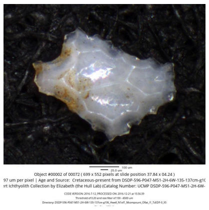<br>DSDP-596-P047-M51-2H-6W-135-137cm-g106_obj00002

5	Four depressions	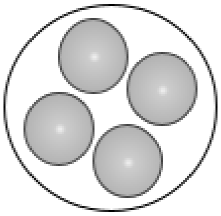	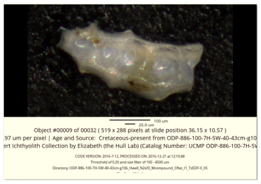 ODP-886-100-7H-5W-40-43cm-g106_obj00009_edf
6	five or more depressions	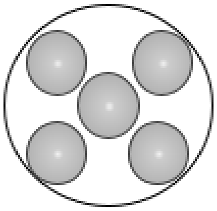	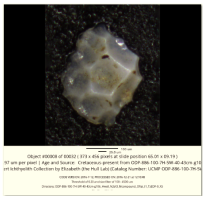 ODP-886-100-7H-5W-40-43cm-g106_obj00008_edf
7	Multiple depressions (>2) but denticle is broken so unable to discern precise number	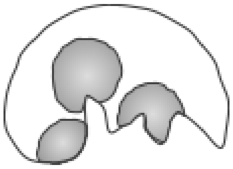	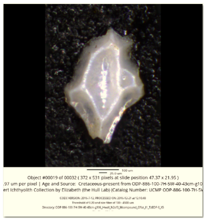 ODP-886-100-7H-5W-40-43cm-g106_obj00019_edf

Trait M1 Character Disparity Description:
Denticles with no depressions are coded as very different from denticles with depressions. Denticles with depressions are then coded as having linear increases in differences from each increasing number of depressions with denticles with multiple depressions but no exact number being coded as the same level of difference to other character states as denticles with two depressions.
Weight in disparity calculation: 1.

M1	1	2	3	4	5	6	7
1	0	1	2	3	4	5	3
2	1	0	1	2	3	4	2
3	2	1	0	1	2	3	1
4	3	2	1	0	1	3	2
5	4	3	2	1	0	1	3
6	5	4	3	3	1	0	4
7	3	2	1	2	3	4	0

**Trait M2: Depression(s) type**<br>Depressions can be further divided by their surroundings on the crown. The most common type of depression are dimples, or depressions surrounded by ridges.				
#	Name	Description	Illustration	Image
0	No depression	Ridges may or may not be present but there are no depressions on the crown.	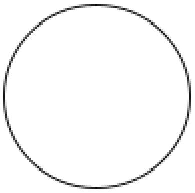	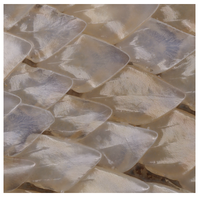<br>*Hemiscyllium ocellatum* - MCZ 155795 (loc. P)
1	Thumbprint (surface layer)	Surface layer impression on the crown without surrounding ridges.	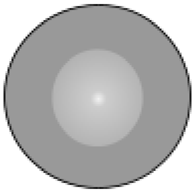	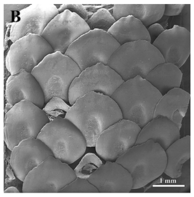<br>*Centroscymnus owstonii,* Fig. B, - Weigmann, S., Vaz, D. F., White, W. T., de Carvalho, M. R., & Thiel, R. (2016)
2	Open tunnel (partially covered)	Partially covered impression where the lateral edges of the crown fold over and cover the lateral edges of the depression.	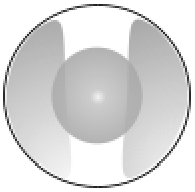	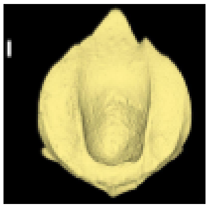<br>*Centroscymnus coelolepis,* Fig. 11 I Vaz, D. F., Avery, T. M., Gabler-Smith, M. K., & Lauder, G. V. (2023).

3	Dimples (depressions surrounded by ridges)	Depressions in the center of a central ridge system shape (clearly surrounded by ridges).	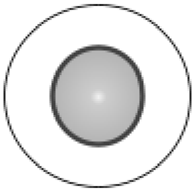	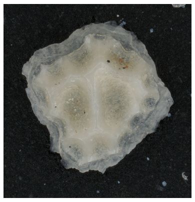 *Chlamydoselachus anguineus* - MCZ 34247 (loc. C)

Trait M2 Character Disparity Description:
Denticles with no depressions are coded as very different from denticles with depressions. Denticles with thumbprint, open tunnel, or dimple depressions are coded as equally different from each other.
Weight in disparity calculation: 0.5.

M2	1	2	3
1	0	1	1
2	1	0	1
3	1	1	0

Trait M3: Location of depression

Depressions can be located in the center, along the edges, or both in the center and along the edges of the crown.

#	Name	Description	Illustration	Image
0	No depression		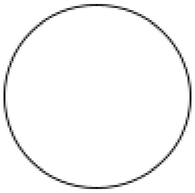	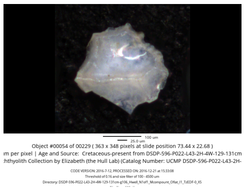<br>DSDP-596-P022-L43-2H-4W-129-131cm-g106_Hwell_N1of35_obj00054
1	Central depression	Depression does not touch the edges of the crown and is generally centered between the posterior and anterior of the crown.	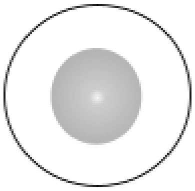	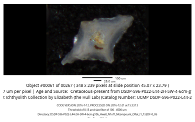<br>DSDP-596-P022-L44-2H-5W-4-6cm-g106_Hwell_N1of1_obj00061
2	Close to edge depression	Depression may touch an edge of the crown and is closer to the posterior or anterior rather than being in the middle.	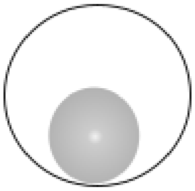	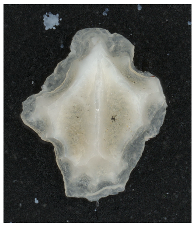<br>*Chlamydoselachus anguineus* - MCZ 34247 (loc. G)
3	Both central and edge depressions	Both central and edge depressions - multiple depressions present with some being in the center and others closer to the edges of the crown.	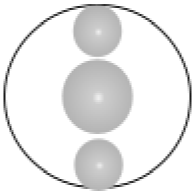	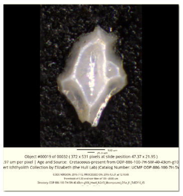<br>ODP-886-100-7H-5W-40-43cm-g106_obj00019_edf

Trait M3 Character Disparity Description:
Denticles with no depressions are coded as very different to denticles with depressions. Denticles with depressions that are both close to the edge of the crown and in the center are also coded as very different from denticles with depressions that are only found along the edge of the crown or the center of the crown. Denticles with depressions that are only found along the edge of the crown or the center of the crown are coded as more similar to each other than to other character states in this trait.
Weight in disparity calculation: 0.5.

M3	1	2	3
**1**	0	1	2
**2**	1	0	2
**3**	2	2	0

Trait M4: Shape of dimple(s)

The dimple shape describes the outline of the lowest area in a central ridge system shape, as clearly outlined by surrounding ridges. Dimple shapes are often difficult to identify and often mimic the shape of the central shape. However, there is occasionally a clear dimple shape which differs from the central shape or mimics the overall crown shape. When a crown has multiple dimples of the same shape they can be coded by that shape but if they differ in shapes they should be coded as “M4.7 multiple with different shapes”

#	Name	Description	Illustration	Image
0	None	There are no dimples on the crown.	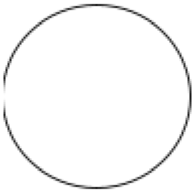	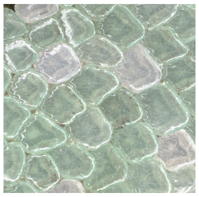 Rhizoprionodon terraenovae - MCZ S-432
1	Circle	The dimple(s) are circular.	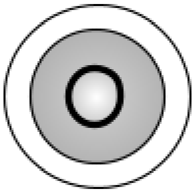	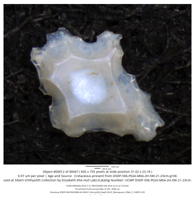 DSDP-596-P024-M04-2H-5W-21-23 cm-g106_Hwell_N 1of1_obj00012
2	Elongated	The dimple is round and elongated.	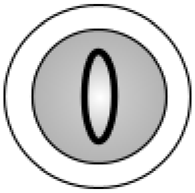	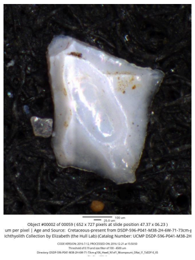 DSDP-596-P041-M38-2H-6W-71-73 cm-g106_obj00002

3	Teardrop (smooth curve)	The dimple is teardrop shaped with a wide round portion which thins to a vertex.	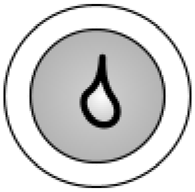	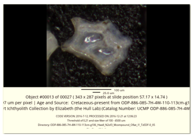 ODP-886-085-7H-4W-110-113cm-g106_obj00013_edf
4	Square/quadrilateral	The dimple is quadrilateral-like with four straight edges and four vertices.	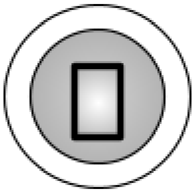	N/A
5	Pentagon	The dimple is pentagon-like with five straight edges and five vertices.	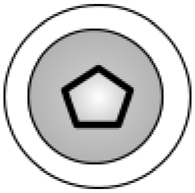	N/A
6	Irregular	The dimple does not have a recognizable or definable shape.	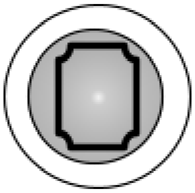	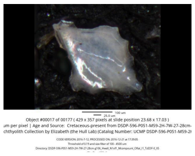 DSDP-596-P051-M59-2H-7W-27-28cm-g106_obj00017_edf.jpeg
7	Multiple different shapes		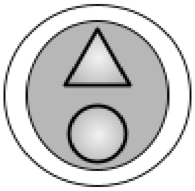	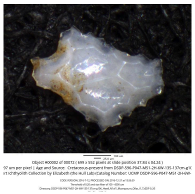 DSDP-596-P047-M51-2H-6W-135-137cm-g106_obj00002

Trait M4 Character Disparity Description:
Denticles with dimples are coded as very different from denticles without dimples and different dimple shapes are all coded as equally different from each other.
Weight in disparity calculation: 0.5.

M4	1	2	3	4	5	6	7
**1**	0	1	1	1	1	1	1
**2**	1	0	1	1	1	1	1
**3**	1	1	0	1	1	1	1
**4**	1	1	1	0	1	1	1
**5**	1	1	1	1	0	1	1
**6**	1	1	1	1	1	0	1
**7**	1	1	1	1	1	1	0

**Trait N1: Secondary ridge features**<br>Secondary ridge features include micro reliefs (smaller ridges that are present as features on top of larger ridges), surface textures (or shallow thin patterns on top of larger ridges or the crown surface), and vertical protrusions on ridges (in which a small spine-like feature protrudes from a ridge). If these are present, all prior ridge character classifications are done on the larger ridges.				
#	Name	Description	Illustration	Image
1	No Ridges		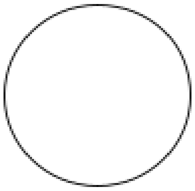	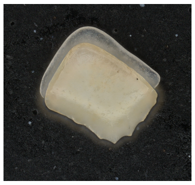<br>*Hemiscyllium ocellatum* - MCZ 155795 (loc. B)
2	No secondary ridge features	Ridges are present but do not have any additional textures or additional ridges on them.	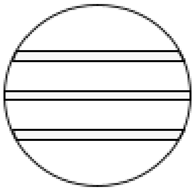	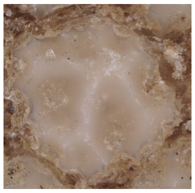<br>*Chlamydoselachus anguineus* - MCZ 34247 (loc. B)
3	Micro-reliefs on ridges	Ridges present and have additional ridge-like features on their surface.	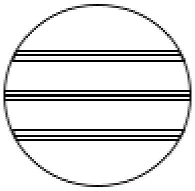	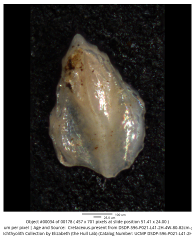<br>DSDP-596-P021-L41-2H-4W-80-82cm-g106_Hwell_N1of1_obj00034

4	Honeycomb surface texture	A thin raised honeycomb or web-like texture covers a portion of the crown and/or ridges.	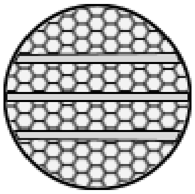	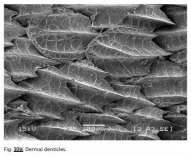 *Zameus squamulosus*, Castro, Pg. 122
5	Wavy surface texture	Thin wavy or scalloped texture covers a portion of the crown and/or ridges.	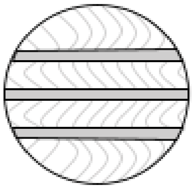	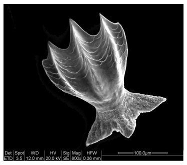 *Carcharhinus leucas, SIO# H48-271*
6	Honeycomb/wavey combination surface texture	Honeycomb and wavy texture are both present on the crown and or ridges.	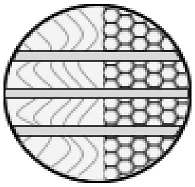	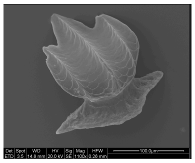 *Rhizoprionodon longurio, SIO# 62-591*
7	Vertical protrusion(s) from ridge(s)	A vertical nub or bump is present on ridge(s).	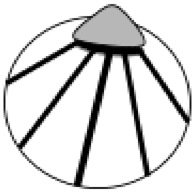	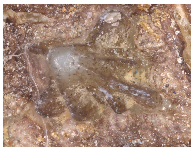 *Oxynotus centrina* MCZ39631 (loc. D)

Trait N1 Character Disparity Description:
Denticles with no ridges are coded as very distinct from denticles that have ridges. Denticles with no secondary ridge features are then coded as being slightly less different than denticles with no ridges compared to other denticles that do have any type of secondary ridge features. Denticles which have vertical protrusions from its ridges are coded as the most different from all other secondary ridge feature categories. Denticles with micro-reliefs on ridges are coded as less distinct from other denticles with other secondary ridge feature categories. Denticles coded as having a specified "surface texture" are coded as more similar to each other than to other categories.
Weight in disparity calculation: 1.

N1	1	2	3	4	5	6	7
1	0	4	4	4	4	4	5
2	4	0	3	3	3	3	5
3	4	3	0	2	2	2	5
4	4	3	2	0	1	1	5
5	4	3	2	1	0	1	5
6	4	3	2	1	1	0	5
7	5	5	5	5	5	5	0

Trait N2: Surface texture location
Surface texture location describes the part of the crown where there is a non-smooth surface, as described in trait N1 in state 4 (honeycomb), 5 (wavy) or 6 (combination of wavy and honeycomb).

#	Name	Description	Illustration	Image
0	None	No secondary ridge features.	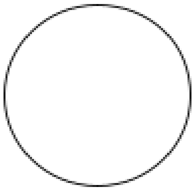	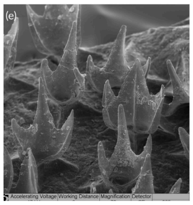 *Scymnodon macracanthus,* Vaz 2021, Fig. 14 (e)
1	Ridges only	The ridges have a rough surface texture.	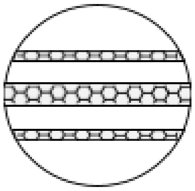	N/A
2	Crown and ridges	Both the crown and ridges have a rough surface texture.	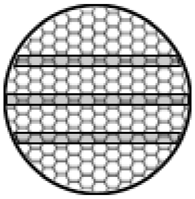	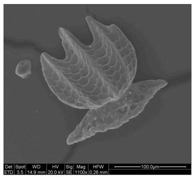 *Sphyrna media, 60-88*
3	Crown except ridges	The crown (but not the ridges) has a rough surface texture.	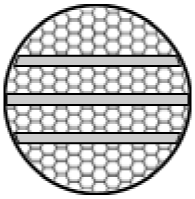	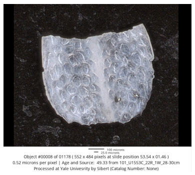 101_U1553C_22R_1W_28-30CM_obj00008
4	Anterior of crown only	The surface texture is rough at the anterior of the crown.	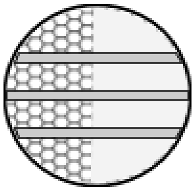	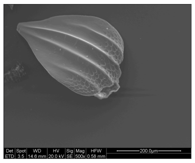 *Mustelus manazo,* SIO# 64-257

5	Middle of crown only	The surface texture is rough at the middle of the crown.	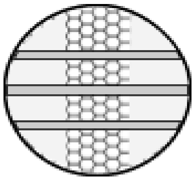	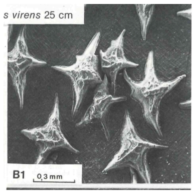<br>*Etmopterus virens*, Reif, Pg. 110, Fig. B1
6	Posterior of crown only	The surface texture is rough at the posterior of the crown.	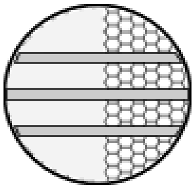	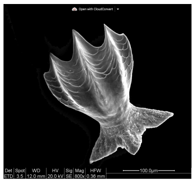<br>*Carcharhinus leucas, SIO# H48-271*
7	Edges of crown only	The surface texture is rough at the edges of the crown.	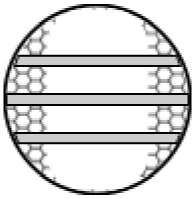	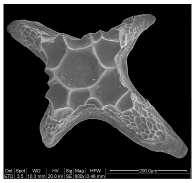<br>*Euprotomicrus bispinatus*, SIO# 98-173

Trait N2 Character Disparity Description:
Denticles with a crown that has surface texture only on its ridges are coded as equally different to other surface textured crown's except for crown's with surface texture on the entire crown except for the ridges. Denticles with surface texture on the entire crown including on the ridges are coded as more different from crown's with surface texture on only the anterior, middle, posterior or edges. All other categories of surface texture differ equally.
Weight in disparity calculation: .5.

N2	1	2	3	4	5	6	7
1	0	1	2	1	1	1	1
2	1	0	1	2	2	2	2
3	2	2	0	1	1	1	1
4	1	2	1	0	1	1	1
5	1	2	1	1	0	1	1
6	1	2	1	1	1	0	1
7	1	2	1	1	1	1	0

Trait N3: Surface texture coverage
Surface texture coverage describes the percent of the crown covered by a non-smooth surface texture as described in N1: Secondary ridge features in state 4 (honeycomb), 5 (wavy) or 6 (combination of wavy and honeycomb) and in N2: Surface texture location.

#	Name	Description	Illustration	Image
0	None	The surface does not have a specified rough surface texture.	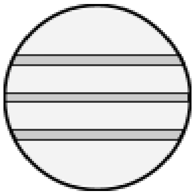	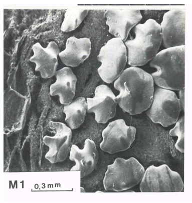 *Chiloscyllium plagiosum,* Reif, Pg. 130, Fig. M1
1	Less than 30%	Less than 30% of the crown has a specified rough surface texture.	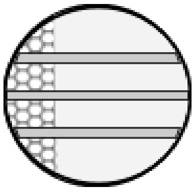	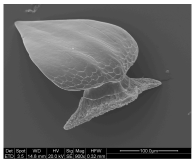 *Mustelus manazo*, SIO# 64-257
2	Between 30% and 70%	Between 30% and 70% of the crown has a specified rough surface texture.	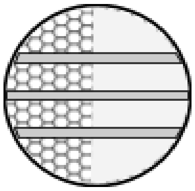	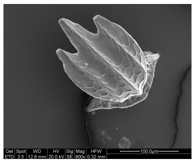 *Scoliodon laticaudus*, SIO# 65-47421`
3	Greater than 70%	Greater than 70% of the crown has a specified rough surface texture.	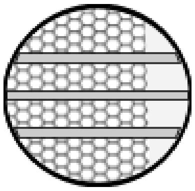	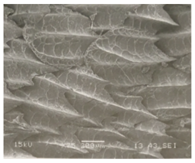 *Zameus squamulosus,* Castro, Pg. 122

Trait N3 Character Disparity Description:
Denticles with surface texture are coded so that crowns with increased coverage differ in one step increments from the previous coverage bin.
Weight in disparity calculation: .5.

N3	1	2	3
1	0	1	2
2	1	0	1
3	2	1	0

Trait O1: Overall base shape
Overall base shape is defined as the overall outline shape of the base, ignoring any topographical features (e.g. grooves, foramen, etc.)

#	Name	Description	Illustration	Image
1	Kite shaped	Base is kite-like in shape with straight edges and two shorter lateral vertices, one short posterior or anterior vertex and a much longer anterior or posterior vertex.	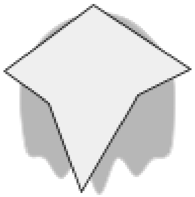	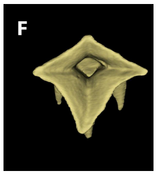 *Centroscymnus coelolepis*, Vaz et al., Figure 10 (F)
2	Kite/cruciform shaped with extended vertices	Base is cruciform in shape with rounded edges and two shorter lateral corners, one shorter posterior or anterior rounded corner and a much longer anterior or posterior corner.	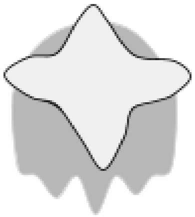	
3	Kite/cruciform shaped missing 1/4 of the cross ridges/extensions	Base is cruciform-like in shape with two longer lateral vertices and one shorter anterior or posterior vertices and a short rounded or straight anterior or posterior edge.	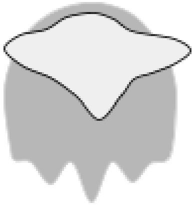	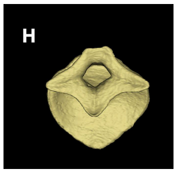 *Centroscymnus coelolepis*, Vaz et al., Figure 4 (H)
4	Rhombus shaped	Base is a quadrilateral with opposite acute angles and straight edges. It is only up to two times as long laterally than it is from anterior to the posterior. May or may not be symmetrical along the lateral axis.	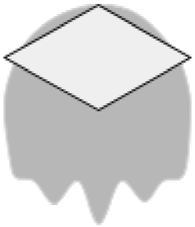	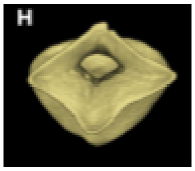 *Centroscymnus coelolepis*, Vaz et al., Figure 8 (H)

5	Rounded rhombus	Base is quadrilateral-like with internal acute angles and rounded edges which may be concave.	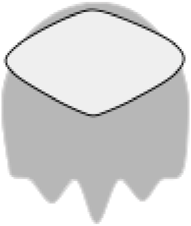	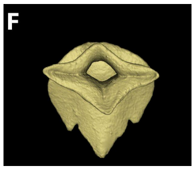 *Centroscymnus coelolepis*, Vaz et al., Figure 12 (F)
6	Stretched rhombus	Base is a quadrilateral with opposite acute angles and straight edges and is more than two times longer laterally than from the anterior to the posterior.	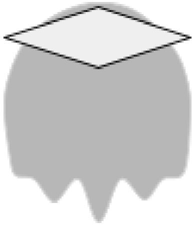	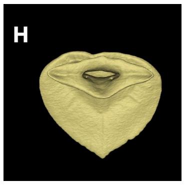 *Centroscymnus coelolepis*, Vaz et al., Figure 14 (H)
7	Trapezoid/ rhombus hybrid (rhombus like but with a flat anterior edge)	Base is rhombus-like but with a flat anterior edge.	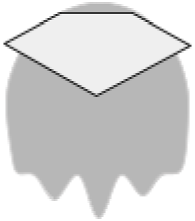	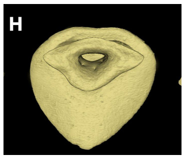 *Centroscymnus coelolepis*, Vaz et al., Figure 13 (H)
8	Oval/oval like	Base is rounded and elongated.	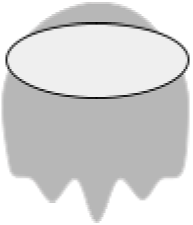	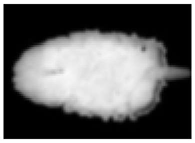 *Raja brachyura,* Gravendeel et al., Figure 6 (median)
9	Circular	Base is rounded and symmetrical around the center.	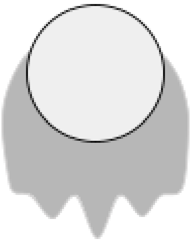	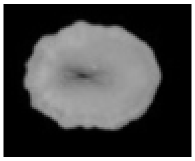 *Dipturus batis,* Gravendeel et al., Figure 18 (median)

10	Tree roots (irregular with many radiating ridges)	Base is irregularly shaped with many radiating protrusions, common for spines.	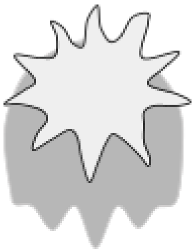	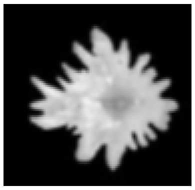 *Raja brachyura,* Gravendeel et al., Figure 6 (pectoral fin)
11	Mirrors crown shape		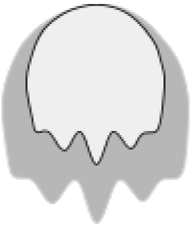	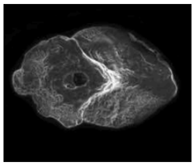 *Isurus oxyrinchus,* Motta et al. (2012) Fig. 5
12	Base extends from crown without distinct separation between crown and base		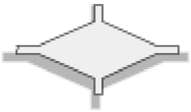	

Trait O1 Character Disparity Description:

Denticles with an overall base shape described as "tree roots" or radiating protrusions from the root foremen are coded as very different from all other base shapes. Denticles with bases that mirror the crown shape are also coded as very different from other base shapes (though less different than those with "tree root" shapes). All other base shapes are coded as equally different from each other. Denticles with no discernable difference between the base and crown are coded as the most significantly different from other base shape trait states.

Weight in disparity calculation: 1.

O1	1	2	3	4	5	6	7	8	9	10	11	12
1	0	1	1	1	1	1	1	1	1	3	2	3
2	1	0	1	1	1	1	1	1	1	3	2	3
3	1	1	0	1	1	1	1	1	1	3	2	3
4	1	1	1	0	1	1	1	1	1	3	2	3
5	1	1	1	1	0	1	1	1	1	3	2	3
6	1	1	1	1	1	0	1	1	1	3	2	3
7	1	1	1	1	1	1	0	1	1	3	2	3
8	1	1	1	1	1	1	1	0	1	3	2	3
9	1	1	1	1	1	1	1	1	0	3	2	3
10	3	3	3	3	3	3	3	3	3	0	3	3
11	2	2	2	2	2	2	2	2	2	3	0	3
12	3	3	3	3	3	3	3	3	3	3	3	0

Trait O2: Base width/length
The base length is defined by the anterior/posterior axis and the width is defined by the width of the lateral edges.

#	Name	Description	Illustration	Image
1	Equal width and length		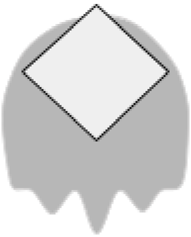	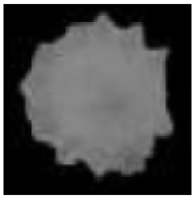 *Raja microocellata,* Gravendeel et al., Figure 12 (star type prickle)
2	Wider than long	Wider laterally than long (anterior to posterior).	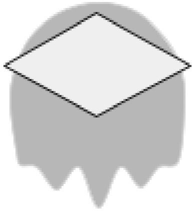	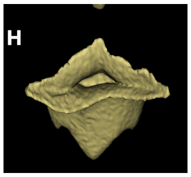 *Centroscymnus coelolepis*, Vaz et al., Figure 6 (H)
3	Longer than wide	Shorter laterally than long (anterior to posterior).	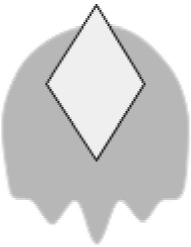	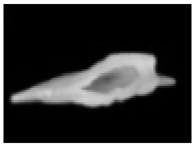 *Raja clavata,* Gravendeel et al., Figure 8 (alar thorn)
4	Unequal width and length (no directionality)		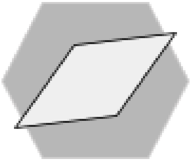	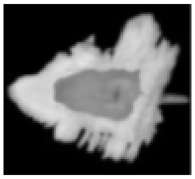 *Raja clavata,* Gravendeel et al., Figure 9 (fin)

Trait O2 Character Disparity Description:

Denticles with no identifiable directionality and thus no identifiable width or length are coded as very different from any denticle with a base that does have an identifiable width and length. Denticles with bases that have equal width and length, a width larger than its length, and larger lengths than widths are all coded as equally different from each other.

Weight in disparity calculation: 1.

O2	1	2	3	4
1	0	1	1	2
2	1	0	1	2
3	1	1	0	2
4	2	2	2	0

Trait O3: Crown to base ratio
The crown to base ratio describes the size of the crown compared to the size of the base.

#	Name	Illustration	Image
1	Crown and base have the same area	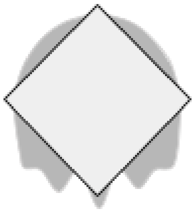	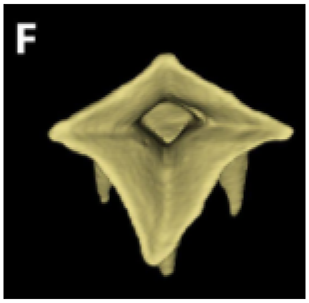<br>*Centroscymnus coelolepis*, Vaz et al., Figure 10 (F)
2	Crown has a larger area	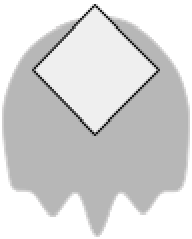	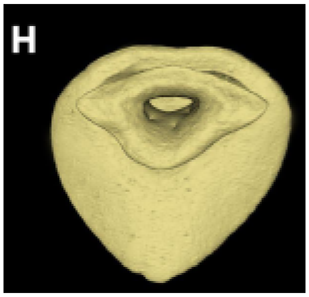<br>*Centroscymnus coelolepis*, Vaz et al., Figure 13 (H)
3	Base has a larger area	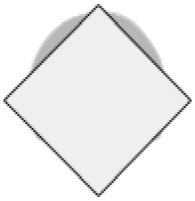	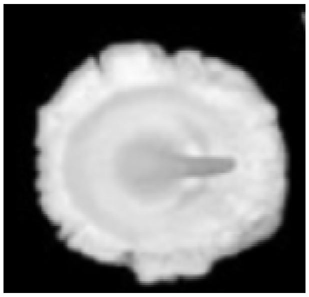<br>*Raja clavata*, Gravendeel et al., Figure 8 (ventral buckler)

Trait O3 Character Disparity Description:
Denticle's crown to base ratio trait states are all coded as equally different from other trait states of this character.
Weight in disparity calculation: 1.

O3	1	2	3
1	0	1	1
2	1	0	1
3	1	1	0

**Trait O4: Number of Grooves**<br>Grooves are lines in the concave underside of the base. They generally point inwards toward the base foramen growing less defined as they reach the center.			
#	Name	Illustration	Image
1	No grooves, completely rounded/smooth	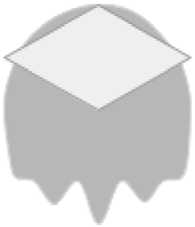	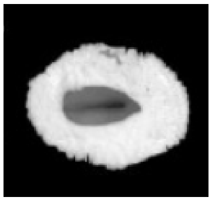<br>*Raja clavata,* Gravendeel et al., Figure 9 (parallel)
2	One	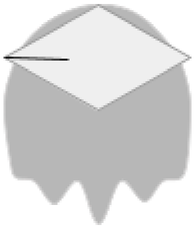	N/A
3	Two	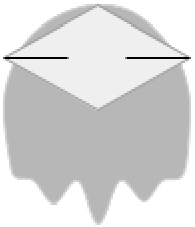	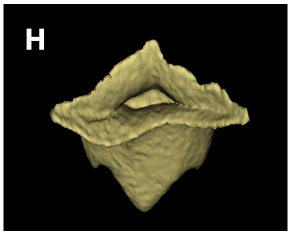<br>*Centroscymnus coelolepis*, Vaz et al., Figure 6 (H)
4	Three	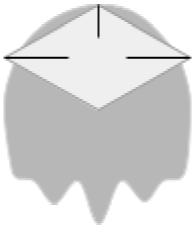	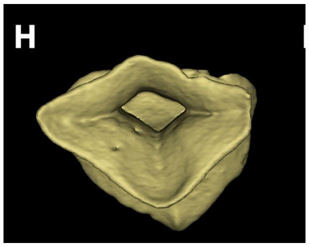<br>*Centroscymnus coelolepis*, Vaz et al., Figure 7 (H)

5	Four	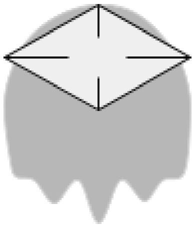	N/A
6	Five	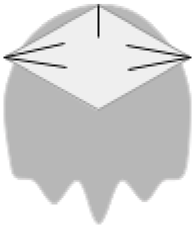	N/A
7	Six (plus)	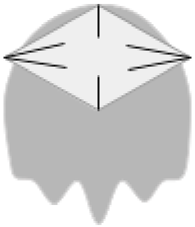	N/A

Trait O4 Character Disparity Description:
Denticles with bases that have no grooves are coded as very different from bases with grooves. All denticles with any number of grooves are coded as equally different from each other.
Weight in disparity calculation: 1.

O4	1	2	3	4	5	6	7
1	0	2	2	2	2	2	2
2	2	0	1	1	1	1	1
3	2	1	0	1	1	1	1
4	2	1	1	0	1	1	1
5	2	1	1	1	0	1	1
6	2	1	1	1	1	0	1
7	2	1	1	1	1	1	0

Trait O5: Root foramen opening shape

The root foramen is defined as the canal which connects the bottom of the base of the denticle up into the crown.

#	Name	Description	Illustration	Image
1	No root opening		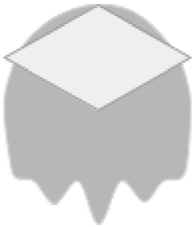	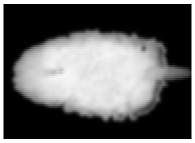 *Raja brachyura,* Gravendeel et al., Figure 6 (median)
2	Rhombus	Quadrilateral with opposite acute angles and straight edges.	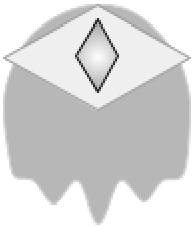	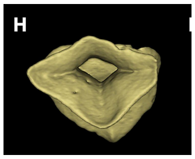 *Centroscymnus coelolepis*, Vaz et al., Figure 7 (H)
3	Elipse	Round and elongated.	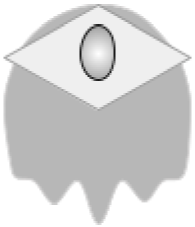	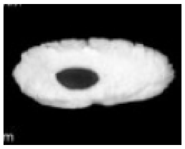 *Raja clavata,* Gravendeel et al., Figure 8 (lateral buckler)
4	Arc	A slice of a circle with a rounded anterior edge and a posterior vertex.	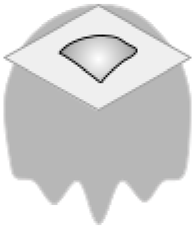	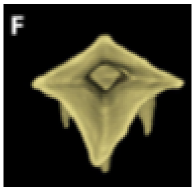 *Centroscymnus coelolepis*, Vaz et al., Figure 10 (F)

5	Mirrors base shape with more rounded edges		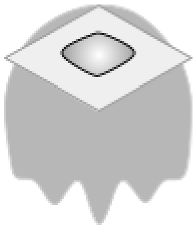	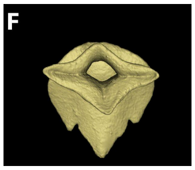<br><br>*Centroscymnus coelolepis*, Vaz et al., Figure 12 (F)

Trait O5 Character Disparity Description:

Denticles with root foramens of any shape are all coded as equally different from each other. Denticles with no root foramen are coded as more different than denticles with a root foramen.

Weight in disparity calculation: 1.

O5	1	2	3	4	5
1	0	2	2	2	2
2	2	0	1	1	1
3	2	1	0	1	1
4	2	1	1	0	1
5	2	1	1	1	0

**Trait O6: Root foramen location**<br>The root foramen is always located somewhere within the outer edges of the base but can be closer to the anterior or posterior.			
#	Name	Illustration	Image
0	No root opening or base not preserved/visible	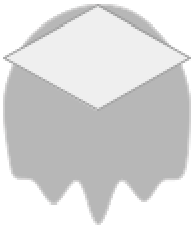	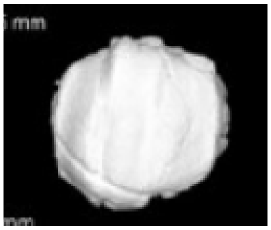<br>*Raja clavata*, Gravendeel et al., Figure 8 (ventral buckler)
1	Center of base	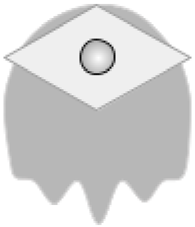	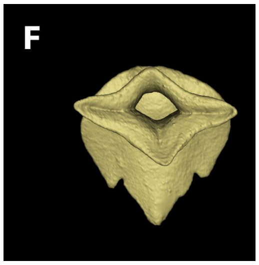<br>*Centroscymnus coelolepis*, Vaz et al., Figure 12 (F)
2	Anterior of base	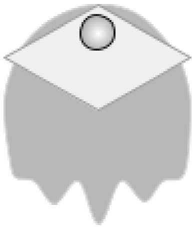	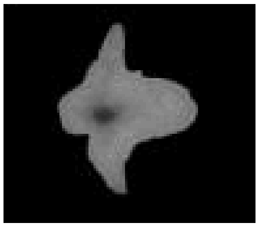<br>*Raja microocellata,* Gravendeel et al., Figure12 (claw type prickle)

3	Posterior of base	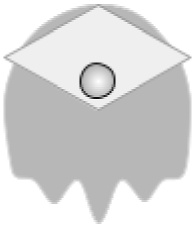	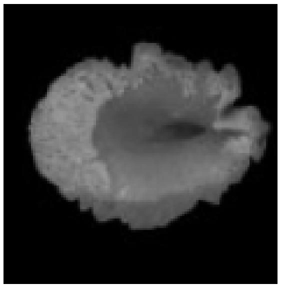<br>*Raja microocellata,*<br>Gravendeel et al., Figure 11 (rostral)

Trait O6 Character Disparity Description:
Denticles with a root foramen are all coded as equally different from each other. Denticles with no root foramen are coded as more different from other denticles.
Weight in disparity calculation: 1.

O6	1	2	3
1	0	1	1
2	1	0	1
3	1	1	0

Trait O7: Peduncle height:width
The peduncle is the vertical structure which connects the base and crown on some denticles.

#	Name	Description	Illustration	Image
1	Equal width and height		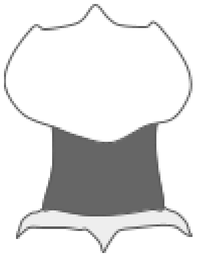	N/A
2	Wider than height		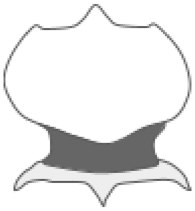	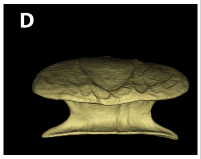 *Centroscymnus coelolepis*, Vaz et al., Figure 14 (D)
3	Higher than width		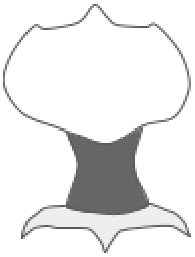	N/A
4	No visible Peduncle (sits flat on surface)	Peduncle is continuous (not distinguishable) from the top of the crown which appears to sit flat on the skin surface, common for geometric denticles.	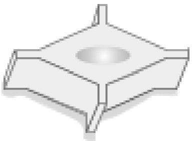	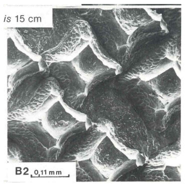 *Isistius brasiliensis,* Castro, Pg. 145

Trait O7 Character Disparity Description:
Denticles with peduncles that have equal width and height, width larger than height and height larger than width are all coded as equally different from each other. Denticles with no peduncle are coded as more different than denticles with peduncles.
Weight in disparity calculation: 1.

O7	1	2	3	4
1	0	1	1	2
2	1	0	1	2
3	1	1	0	2
4	2	2	2	0

Trait O8: Crown:root angle
The crown to root angle is described by the angle between the skin and crown. Spines often display a perpendicular angle while most other denticles display an obtuse or parallel angle. Acute angles are relatively rare.

#	Name	Description	Illustration	Image
1	Perpendicular (Crown pointing directly up from base, e.g. spine)	Crown is completely vertical.	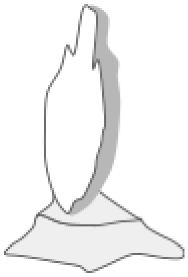	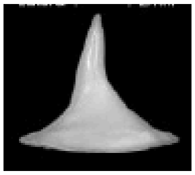 *Raja undulata,* Gravendeel et al. Fig. 17 (lateral)
2	Obtuse (Crown pointing away from base)	Crown's posterior is pointed up vertically and is positioned higher than the anterior.	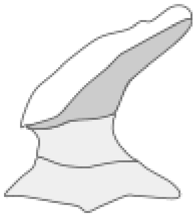	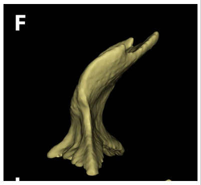 *Centroscymnus coelolepis*, Vaz et al., Figure 6 (F)
3	Parallel (Crown directly over Base)	Crown is parallel to the skin with both the posterior and anterior at equal vertical heights.	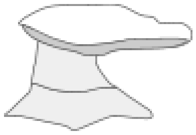	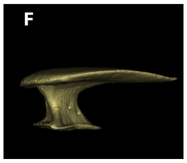 *Centroscymnus coelolepis*, Vaz et al., Figure 13 (F)
4	Acute (Crown pointing towards base)	Crown's posterior is pointed down vertically and is positioned lower than the anterior.	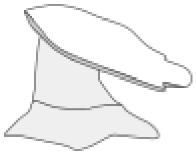	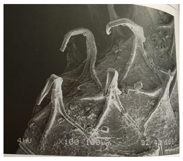 *Cephalurus cephalus*, Castro, Pg. 321

Trait O8 Character Disparity Description:

The crown-to-base angle is a semi-continuous trait with perpendicular being coded as closest to obtuse, then parallel, then acute.

Weight in disparity calculation: 1.

O8	1	2	3	4
1	0	1	2	3
2	1	0	1	2
3	2	1	0	1
4	3	2	1	0

Trait O9: Base to crown connection location
Base to crown connection location describes the part of the crown where the base, either directly or via peduncle, is located.

#	Name	Illustration	Image
1	At anterior of crown	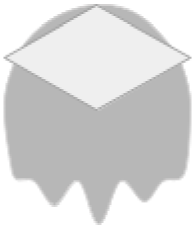	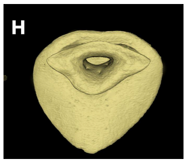 *Centroscymnus coelolepis*, Vaz et al., Figure 13 (H)
2	At center of crown	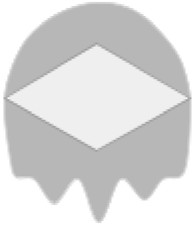	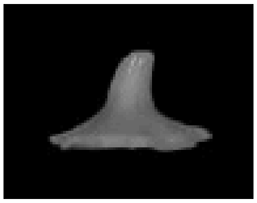 *Dipturus batis,* Gravendeel et al. Fig. 18 (lateral)
3	At posterior of crown	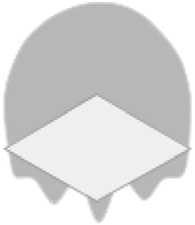	N/A

Trait O9 Character Disparity Description:
Denticles' base to crown connection location are coded as being equally different from each other.
Weight in disparity calculation: 1.

O9	1	2	3
1	0	1	1
2	1	0	1
3	1	1	0

Trait O10: Mound
Mounds are defined by a raised portion below the crown but above the skin surface which is larger in area than the crown.

#	Name	Description	Illustration	Image
1	Not mounded	The base to crown connection is surrounded by a smooth flat area around where the crown emerges.	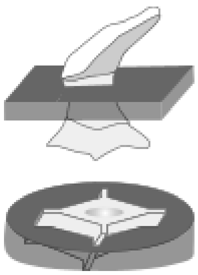	
2	Mounded	The base to crown connection is surrounded by a raised area where the crown emerges.	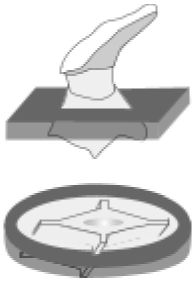	

Trait O10 Character Disparity Description:
Denticles with a mound on the skin surface are coded as different from denticles with only the crown erupting through the skin (non mounded).
Weight in disparity calculation: 0.1.

O10	1	2
1	0	1
2	1	0

References

Castro, J. I. (2010). The sharks of North America, Oxford University Press.

Dillon, E. M. (2022). Reconstructing Historical Shark Communities on Coral Reefs Using Fossil Dermal Denticle Assemblages. University of California, Santa Barbara.

Feichtinger, I., Adnet, S., Cuny, G., Guinot, G., Kriwet, J., Neubauer, T. A., ... & Harzhauser, M. (2021). Comment on “An early Miocene extinction in pelagic sharks”. Science, 374(6573), eabk0632.

Gabler-Smith, M. K., Wainwright, D. K., Wong, G. A., & Lauder, G. V. (2021). Dermal denticle diversity in sharks: novel patterns on the interbranchial skin. Integrative Organismal Biology, 3(1), obab034.

Motta P, Habegger ML, Lang A, Hueter R, Davis J. 2012. Scale morphology and flexibility in the shortfin mako Isurus oxyrinchus and the blacktip shark Carcharhinus limbatus. Journal of Morphology 273:1096–1110.

Reif, W.-E. (1985). Squamation and ecology of sharks, Senckenbergische Naturforschende Gesellschaft.

Vaz, D. F., & De Carvalho, M. R. (2013). Morphological and taxonomic revision of species of Squatina from the Southwestern Atlantic Ocean (Chondrichthyes: Squatiniformes: Squatinidae). Zootaxa, 3695(1), 1-81.

Vaz, D. F. (2021). *Scymnodon plunketi* (Waite, 1910): a junior synonym of Scymnodon macracanthus (Regan, 1906) (*Somniosidae: Elasmobranchii*). Journal of Fish Biology, 99(2), 472-494.

Weigmann, S., Vaz, D. F., White, W. T., de Carvalho, M. R., & Thiel, R. (2016). Distribution and comments on the morphology of *Centroscymnus owstonii* Garman, 1906 (Squaliformes: Somniosidae), with focus on its occurrence in the Indian Ocean. Marine Biodiversity, 46, 641-653.
