# Supplementary material for: Quantifying the Denticle Multiverse: A Standardized Coding System to Capture Three Dimensional Morphological Variations for Quantitative Evolutionary and Ecological Studies of Elasmobranch Denticles
Source: Integr Org Biol. 2025 May 13;7(1):obaf021. doi: 10.1093/iob/obaf021 (PMC12576789; doi:10.1093/iob/obaf021)
Supplement: obaf021_Supplemental_Files [file obaf021_supplemental_files.zip › Appendix_02-Line Drawings.pdf]

Character States for Denticle Morphological description (page 1 of 2)
Corresponds with *ichthyoliths* r package denticles_v0.5 - 15 August 2024

Shape Descriptors (A)

A1. Overall Shape [2]

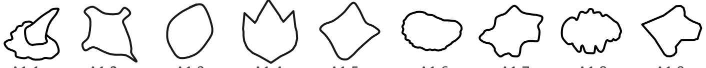

A1.1. Spine; A1.2. Cruciform; A1.3. Circular/Oval; A1.4. Spade; A1.5. Diamond/Rectangle; A1.6. Elongated Asymmetrical; A1.7. Other; A1,8. Fan-like; A1.9. Arrow

A2. Spade Shape [1]

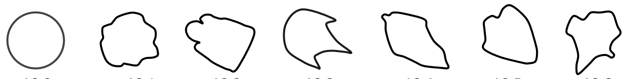

A2.0 Not a Spade; A2.1 Rounded; A2.2. Squared; A2.3. Pointed; A2.4. Stretched; A2.5. Lobed; A2.6. Irregular

Anterior (base, B) & Posterior (tip, C) Characters

B1. Anterior (Base) Shape [1]

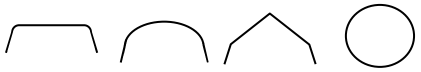

B1.1 Straight (edge); B1.2 Rounded; B1.3 Vertex; B1.4 Lacks Directionality

B2. Anterior (base) Marginal Macro Texture [1]

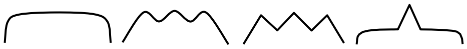

B2.1 Smooth; B2.2 Scalloped; B2.3 Pointed; B2.4 Single vertex

B3. Anterior (base) Marginal Micro Texture [1]

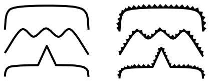

B3.1 Smooth; B3.2 Distinct serrated

C1. Posterior (tip) Shape [1]

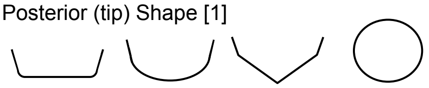

C1.1 Straight (edge); C1.2 Rounded; C1.3 Vertex; C1.4 Lacks Directionality

C2. Posterior (tip) Marginal Macro Texture [1]

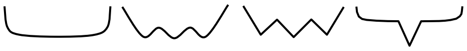

C2.1 Smooth; C2.2 Scalloped; C2.3 Pointed; C2.4 Single vertex

C3. Posterior (tip) Marginal Edge Micro Texture [1]

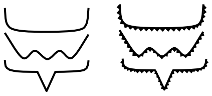

B3.1 Smooth; B3.2 Distinct serrated

D1. Planes of Symmetry [1]

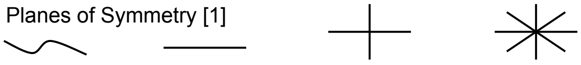

D1.1 Asymmetrical; D1.2 One plane of symmetry; D1.3 Two planes of symmetry; D1.4 3+ or Radial symmetry

Cusps (E)

E1. Number of Cusps [1]

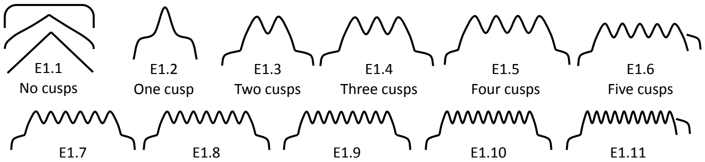

E1.1 No cusps; E1.2 One cusp; E1.3 Two cusps; E1.4 Three cusps; E1.5 Four cusps; E1.6 Five cusps; E1.7 Six cusps; E1.8 Seven cusps; E1.9 Eight cusps; E1.10 Nine cusps; E1.11 Ten+ cusps

E2. Cusp Association [1]

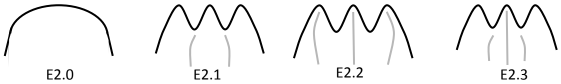

E2.0 No cusps; E2.1 Cusps independent from ridges; E2.2 All Cusps associated with ridges; E2.3 Ridges and cusps sometimes independent

E3. Cusp Similarity [0.5]

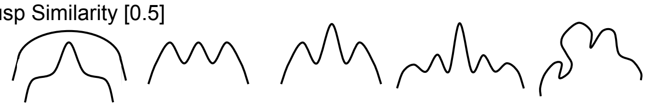

E3.0 No cusps or one cusp; E3.1 Cusps are same length/width/shape; E3.2 Central cusp is distinct; E3.3 Cusps opposite each other are similar; E3.4 Irregular

E4. Relative maximum cusp length [0.5]

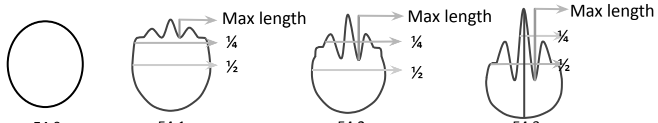

E4.0 No cusps; E4.1 Cusp length < ¼ Crown Length; E4.2 Cusp length between ¼ and < ½ Crown length; E4.3 Cusp length > ½ Crown length

Ridges (F, G, H)

F1. Ridge System [2]

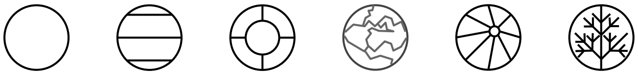

F1.1 Smooth; F1.2 Linear; F1.3 Geometric; F1.4 Meandering; F1.5 Spine; F1.6 Branching

G1. Number of Ridge Segments [1]

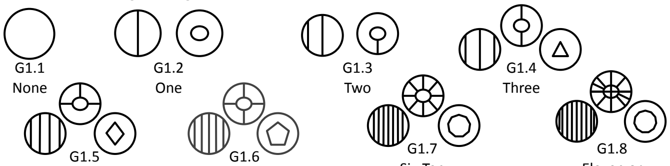

G1.1 None; G1.2 One; G1.3 Two; G1.4 Three; G1.5 Four; G1.6 Five; G1.7 Six-Ten; G1.8 Eleven or more

G2. Number of Independent Ridges [1]

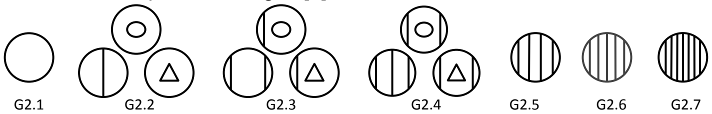

G2.1 None; G2.2 One; G2.3 Two; G2.4 Three; G2.5 Four; G2.6 Five; G2.7 Six+

G3. Ridge Outgrowths [1]

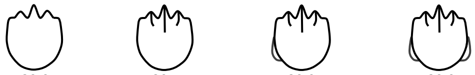

G3.0 No ridges; G3.1 No ridge bumps; G3.2 One ridge bump; G3.3 Two ridge bumps

H1. Ridge Orientation [1]

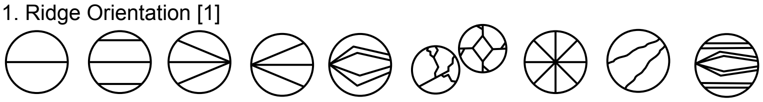

H1.0 Only one Ridge/No Ridges; H1.1 Parallel; H1.2 Converges from base; H1.3 Diverges from base; H1.4 Diverges then converges; H1.5 Ridges intersect/branch; H1.6 Apex Radial (spine); H1.7 Irregular/no discernable base; H1.8 Multiple orientations

Central (I) and Non-Central Ridges (J)

I1. Central Ridge(s) or trough Disparity [1]

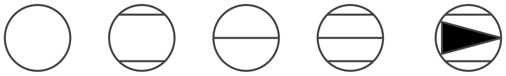

I1.0 No Ridges; I1.1 No central ridge; I1.2 Only one ridge; I1.3 Same as other ridges; I1.4 Distinct from other ridges

I2. Central Ridge Directionality [0.5]

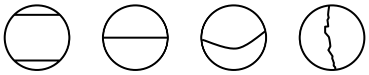

I2.0 No Ridges/No central ridge; I2.1 Straight; I2.2 Curved; I2.7 Meandering

I3. Central Ridge Width [0.5]

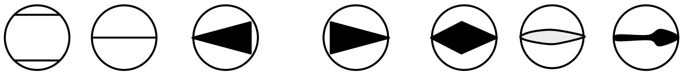

I3.0 None; I3.1 Parallel; I3.3 Triangular widening from anterior; I3.4 Triangular thinning from anterior; I3.5 Diamond; I3.6 Trough; I3.7 Irregular

J1. Non-Central Ridge Directionality [0.5]

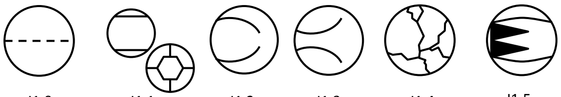

J1.0 No Ridges/only a central ridge; J1.1 Straight; J1.2 Concave Curved; J1.3 Convex Curved; J1.4 Meandering; J1.5 Multiple or Irregular shapes

J2. Non-Central Ridge Width [0.5]

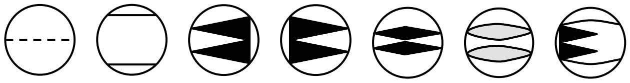

J2.0 No Ridges/only a central ridge; J2.1 Straight; J2.2 Triangular widening from base; J2.3 Triangular thinning from base; J2.4 Diamond-like; J2.5 Multiple or Irregular shapes; J2.6 Multiple or Irregular shapes

Character States for Denticle Morphological description (page 2 of 2)
Corresponds with *ichthyoliths* r package denticles_v0.5 - 15 August 2024

Central Ridge System (K)

K1. Central Ridge System Shape [1]

- K1.1 Ridges present but no central shape
- K1.2 Circular/oval
- K1.3 Triangular
- K1.4 Quadrilateral
- K1.5 Pentagonal
- K1.6 Hexagon
- K1.7 Heptagon
- K1.8 Mound
- K1.9 Octagon
- K1.10 Multiple
- K1.11 Other/Irregular

K2. Central Ridge System Shape Planes of Symmetry [1]

- K2.1 Central shape present, no symmetry
- K2.2 One
- K2.3 Two
- K2.4 Radial

Ridge Size (L)

L1. Ridge length [1]

- L1.1 No ridges
- L1.2 Ridge length = lateral length
- L1.3 Ridge ends mid crown/ Ridge begins mid crown
- L1.4 Ridge begins and ends mid crown
- L1.5 Includes ridges described by a combo

L2. Ridge Definition [1]

- L2.1 Not Clearly Defined
- L2.2 Clearly defined on part of the crown but gets shallower
- L2.3 Clearly Defined
- L2.4 Only the central ridge is clearly defined

L3. Ridge/Ridge Height [1]

- L3.1 Only one ridge
- L3.2 Equal heights
- L3.3 Variable heights

L4. Ridge/Trough Angularity [1]

- L4.1 Profiles are rounded
- L4.2 Profiles are triangular
- L4.3 Profiles are funnel shaped (rounded with a skinnier point)
- L4.4 Profiles are variable or described by another shape
- L4.5 Profiles are rectangular

Depressions & Dimples (M)

M1. Number of depressions [1]

- M1.1 None
- M1.2 One
- M1.3 Two
- M1.4 Three
- M1.5 Four
- M1.6 Five or more
- M1.7 2+ depressions but broken

M2. Depression(s) type [1]

- M2.1 Thumbprint (surface layer)
- M2.2 Open tunnel (partially covered)
- M2.3 Dimples (depressions surrounded by ridges)

M3. Location of depression [1]

- M3.1 Central
- M3.2 Close to edge
- M3.3 Central & edge

M4. Shape of dimple(s) [1]

- M4.1 Circle
- M4.2 Elongated
- M4.3 Teardrop
- M4.4 Quadrilateral
- M4.5 Pentagon
- M4.6 Irregular
- M4.7 Multiple dimples w/ different shapes

Secondary Ridge Features (N)

N1. Secondary ridge features [1]

- N1.0 No ridges
- N1.1 No secondary characteristics
- N1.2 Micro reliefs
- N1.3 Honeycomb
- N1.4 Wavy
- N1.5 Honeycomb/ Wavy Combo
- N1.4 Vertical protrusions

N2. Surface texture location [0.5]

- N2.0 None
- N2.1 Ridges only
- N2.2 Crown & Ridges
- N2.3 Crown only
- N2.4 Anterior
- N2.5 Middle
- N2.6 Posterior
- N2.7 Edges

N3. Surface texture coverage [0.5]

- N3.0 None
- N3.1 <30%
- N3.2 30%<x<70%
- N3.3 >70%

Denticle Base (O)

O1. Overall base shape [0.5]

- O1.1 Kite shaped
- O1.2 Kite/cruci form
- O1.3 Partial Kite/cruciform
- O1.4 Rhombus shaped
- O1.5 Rounded rhombus
- O1.6 Stretched rhombus
- O1.7 Trapezoid /rhombus hybrid
- O1.8 Oval/oval like
- O1.9 Circular
- O1.10 Tree roots
- O1.11 Mirrors crown shape
- O1.12 Base extends crown

O2. Base width/length [0.5]

- O2.1 Equal width and length
- O2.2 Wider than long
- O2.3 Longer than wide
- O2.4 Unequal width and length (no directionality)

O3. Crown to base ratio [0.5]

- O3.1 Crown & root have the same area
- O3.2 Crown has a larger area
- O3.3 Base has a larger area

O4. Number of Grooves [0.5]

- O4.1 No grooves
- O4.2 One
- O4.3 Two
- O4.4 Three
- O4.5 Four
- O4.6 Five
- O4.7 Six (+)

O5. Root foramen opening shape [0.5]

- O5.1 No root opening
- O5.2 Rhombus
- O5.3 Elipse
- O5.4 Arc
- O5.5 Mirrors base shape with rounded edges

O6. Root foramen location [0.5]

- O6.0 No root opening
- O6.1 Center of base
- O6.2 Anterior of base
- O6.3 Posterior of base

O7. Peduncle height:width [0.5]

- O7.1 Equal width and height
- O7.2 Wider than height
- O7.3 Higher than width
- O7.4 No Peduncle (sits flat on surface)

O8. Crown:root angle [0.5]

- O8.1 Perpendicular
- O8.2 Obtuse
- O8.3 Parallel
- O8.4 Acute

O9. Base to crown connection location [0.5]

- O9.1 At anterior of crown
- O9.2 At center of crown
- O9.3 At posterior of crown

O10. Mound [0.5]

- O10.1 Not mounded
- O10.2 Mounded
