# Supplementary material for: Quantifying the Denticle Multiverse: A Standardized Coding System to Capture Three Dimensional Morphological Variations for Quantitative Evolutionary and Ecological Studies of Elasmobranch Denticles
Source: Integr Org Biol. 2025 May 13;7(1):obaf021. doi: 10.1093/iob/obaf021 (PMC12576789; doi:10.1093/iob/obaf021)
Supplement: obaf021_Supplemental_Files [file obaf021_supplemental_files.zip › Appendix_03-Morphotypes.pdf]

References used in this study

Ankhelyi, M. V., D. K. Wainwright and G. V. Lauder (2018). "Diversity of dermal denticle structure in sharks: Skin surface roughness and three-dimensional morphology." Journal of Morphology 279(8): 1132-1154.

Castro, J. I. (2010). The sharks of north America, Oxford University Press.

Dillon, E. M. (2022). Reconstructing Historical Shark Communities on Coral Reefs Using Fossil Dermal Denticle Assemblages. University of California, Santa Barbara.

Dillon, E. M., Norris, R. D., & Dea, A. O. (2017). Dermal denticles as a tool to reconstruct shark communities. Marine Ecology Progress Series, 566, 117-134.

Feichtinger, I., Adnet, S., Cuny, G., Guinot, G., Kriwet, J., Neubauer, T. A., ... & Harzhauser, M. (2021). Comment on “An early Miocene extinction in pelagic sharks”. Science, 374(6573), eabk0632.

Gabler-Smith, M. K., Wainwright, D. K., Wong, G. A., & Lauder, G. V. (2021). Dermal denticle diversity in sharks: novel patterns on the interbranchial skin. Integrative Organismal Biology, 3(1), obab034.

Gravendeel, R., W. Van Neer and D. Brinkhuizen (2002). "An identification key for dermal denticles of Rajidae from the North Sea." International Journal of Osteoarchaeology 12(6): 420-441.

Lourtie, A., Duchatelet, L., Straube, N., Puozzo, N., Grace, M. A., Naylor, G. J., & Delroisse, J. (2022). Placoid scales in bioluminescent sharks: Scaling their evolution using morphology and elemental composition. Frontiers in Marine Science, 9, 908237.

Marshall, A. D., L. J. Compagno and M. B. Bennett (2009). "Redescription of the genus Manta with resurrection of Manta alfredi (Krefft, 1868)(Chondrichthyes; Myliobatoidei; Mobulidae)." Zootaxa 2301(1): 1-28.

Reif, W.-E. (1985). Squamation and ecology of sharks, Senckenbergische Naturforschende Gesellschaft.

Serra-Pereira, B., I. Figueiredo, I. Farias, T. Moura and L. Gordo (2008). "Description of dermal denticles from the caudal region of Raja clavata and their use for the estimation of age and growth." ICES Journal of Marine Science 65(9): 1701-1709.

Vaz, D. F., & De Carvalho, M. R. (2013). Morphological and taxonomic revision of species of Squatina from the Southwestern Atlantic Ocean (Chondrichthyes: Squatiniformes: Squatinidae). Zootaxa, 3695(1), 1-81.

Vaz, D. F., & de Carvalho, M. R. (2018). New Species of Squatina (Squatiniformes: Squatinidae) from Brazil, with comments on the taxonomy of angel sharks from the Central and Northwestern Atlantic. Copeia, 106(1), 144-160.

Vaz, D. F. (2021). Scymnodon plunketi (W aite, 1910): a junior synonym of Scymnodon macracanthus (R egan, 1906)(S omniosidae: E lasmobranchii). Journal of Fish Biology, 99(2), 472-494.

Weigmann, S., Vaz, D. F., White, W. T., de Carvalho, M. R., & Thiel, R. (2016). Distribution and comments on the morphology of Centroscymnus owstonii Garman, 1906 (Squaliformes: Somniosidae), with focus on its occurrence in the Indian Ocean. Marine Biodiversity, 46, 641-653.

White, W. T., Vaz, D. F., Ho, H. C., Ebert, D. A., de Carvalho, M. R., Corrigan, S., ... & Naylor, G. J. (2015). Redescription of Scymnodon ichiharai Yano and Tanaka 1984 (Squaliformes: Somniosidae) from the western North Pacific, with comments on the definition of somniosid genera. Ichthyological Research, 62, 213-229.

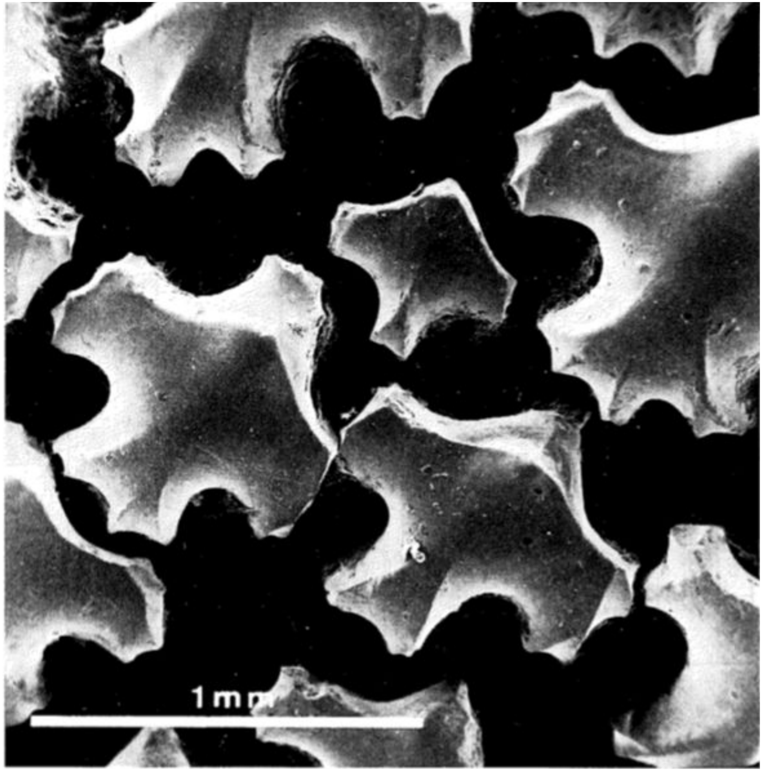

Heterodontus japonicus, Reif, Pg. 25, Fig. 1D

Airplane

The Airplane type has a fusiform shape with one wide central ridge. Perpendicular to and on either side of this center shape are two curved "wings" angled slightly toward each other.

Taxonomic Citation

Heterodontus japonicus, Reif, Pg. 25, Fig. 1D

Oxynotus centrina, Reif, Pg. 120, Fig. B3

Oxynotus centrina, Reif, Pg. 120, Fig. DF

Prionace glauca, Reif, Pg. 214, Fig. B4

Modern Only

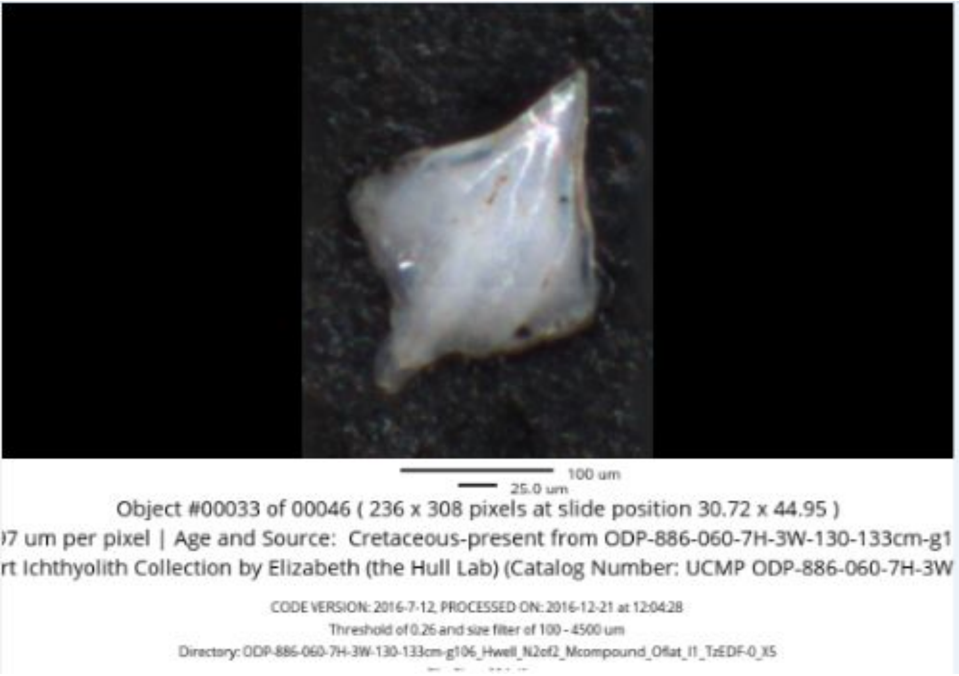

ODP-886-060-7H-3W-130-133cm-g106_obj00033_edf

Angular Edged Diamond

The angular edged diamond has a diamond-like shape with a shallow central ridge which bisects the crown and extends its length. It also has a horizontal line of symmetry.

Fossil Only

Angular Straight Edge

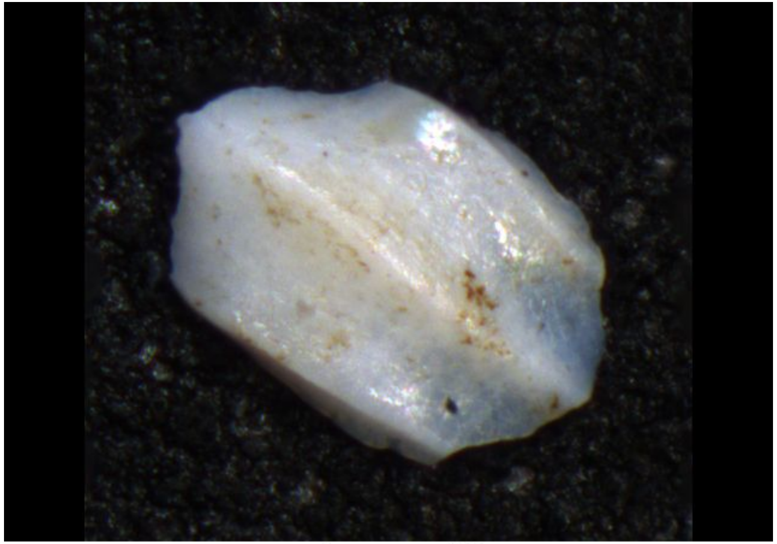

Object #00001 of 00012 (506 x 448 pixels at slide position 52.71 x 12.01)
um per pixel | Age and Source: Cretaceous-present from DSDP-596-P017-L33-2H-3W-31-33cm-
Ichthyolith Collection by Elizabeth (the Hull Lab) (Catalog Number: UCMP DSDP-596-P017-L33-2

CODE VERSION: 2016-7-12, PROCESSED ON: 2016-12-21 at 15:31:16
Threshold of 0.21 and size filter of 100 - 4500 um
Directory: DSDP-596-P017-L33-2H-3W-31-33cm-g106_Hwell_N1of1_Mcompount_Oflat_I1_TzEDF-0_X5

DSDP-596-P017-L33-2H-3W-31-33cm-g106_Hwell_N1of1_obj00001

The angular straight edge type has a diamond-like shape with a central ridge which extends the length of the crown and bisects it. On either side there are two shorter ridges which are straight and define the side edges of the crown.

Fossil Only

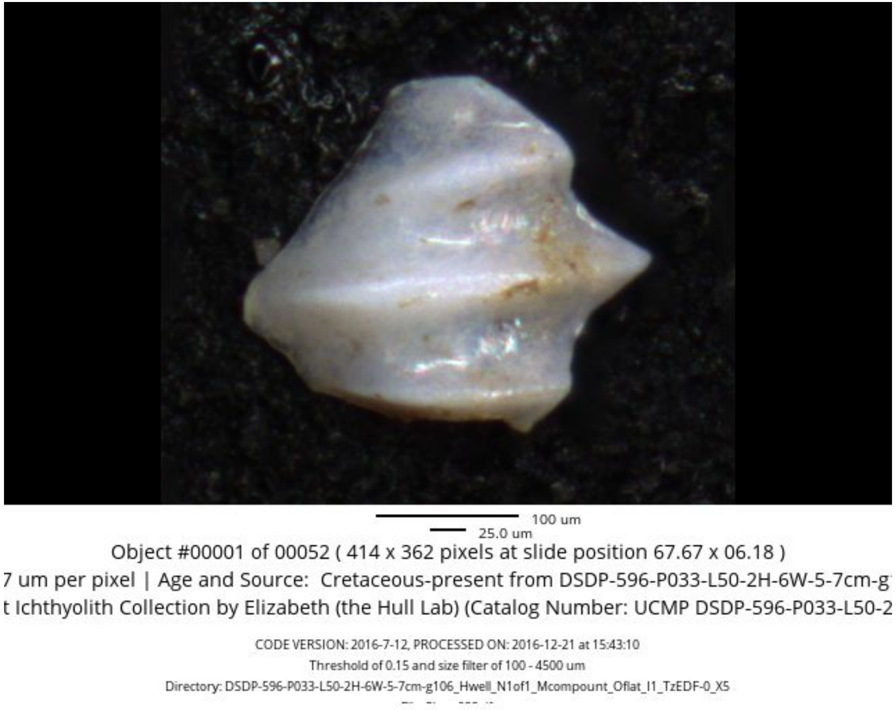

DSDP-596-P033-L50-2H-6W-5-7cm-g106_Hwell_N1of1_obj00001

Arrowhead

The arrowhead type has an arrow like shape with a pointed anterior which is thinner than its pointed posterior. It has a central ridge which extends the length of the crown defining a cusp and two shorter side ridges which may or may not also define cusps.

Fossil Only

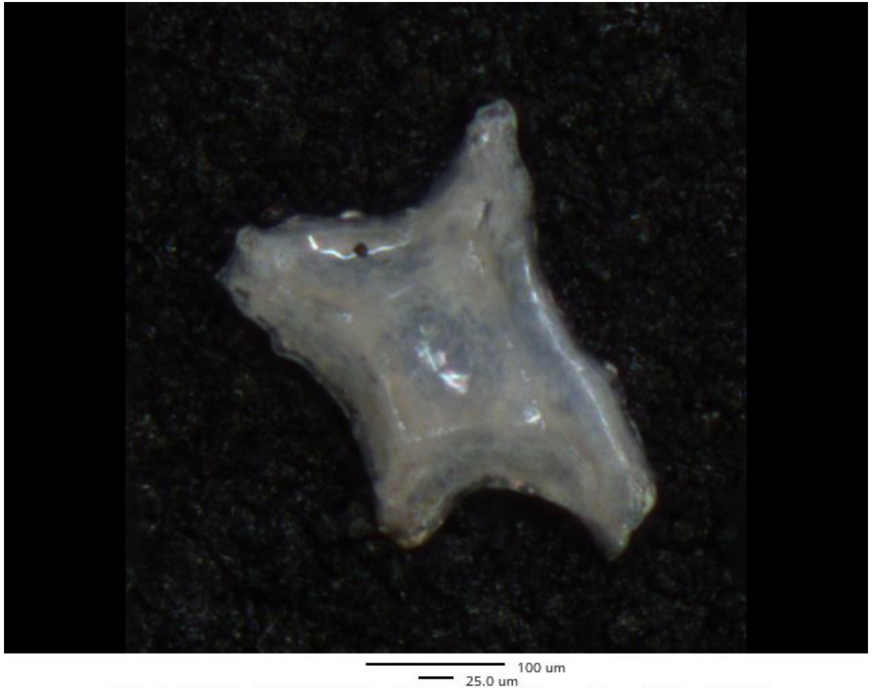

Object #00013 of 00095 (460 x 482 pixels at slide position 43.05 x 08.82)
um per pixel | Age and Source: Cretaceous-present from DSDP-596-P039-L51-2H-6W-57-59cm-[illegible]
Ichthyolith Collection by Elizabeth (the Hull Lab) (Catalog Number: UCMP DSDP-596-P039-L51-2[illegible]

CODE VERSION: 2016-7-12, PROCESSED ON: 2016-12-21 at 15:50:00
Threshold of 0.12 and size filter of 100 - 4500 um
Directory: DSDP-596-P039-L51-2H-6W-57-59cm-g106_Hwell_N1of1_Mcompount_Oflat_I1_TzEDF-0_X5

DSDP-596-P039-L51-2H-6W-57-59cm-g106_Hwell_N1of1_obj00013

Asymmetrical Kite with Oval Center

The asymmetrical kite with oval center has a cruciform shape with a pair of shorter horizontal ridges and a pair of longer vertical ridges on four “vertices” - one of which is longer than the rest. The type is defined by its four sided central ridge system shape with ridges of differing lengths (asymmetrical) and which outlines an elongated dimple in the center.

Fossil Only

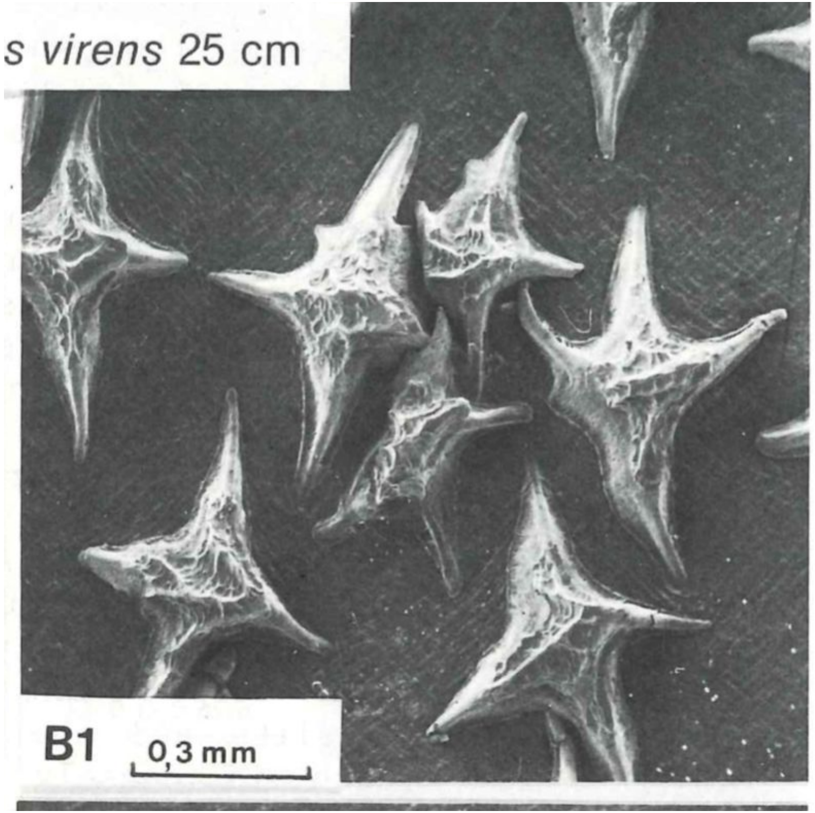

Etmopterus virens, Reif, Pg. 110, Fig. B1

Asymmetrical Kite with Ridged Mound

The asymmetrical kite with ridged mound has a cruciform shape with four distinct sections each composed of a single ridge. It is distinct from other kites because of its increasing height or raised “mound” in the center rather than a depression. This mound is further described by many meandering micro-reliefs.

Taxonomic Citation

Etmopterus virens, Reif, Pg. 110, Fig. B1
Manta birostris, Marshall, Compagno, and Bennett Fig. 6

Modern Only

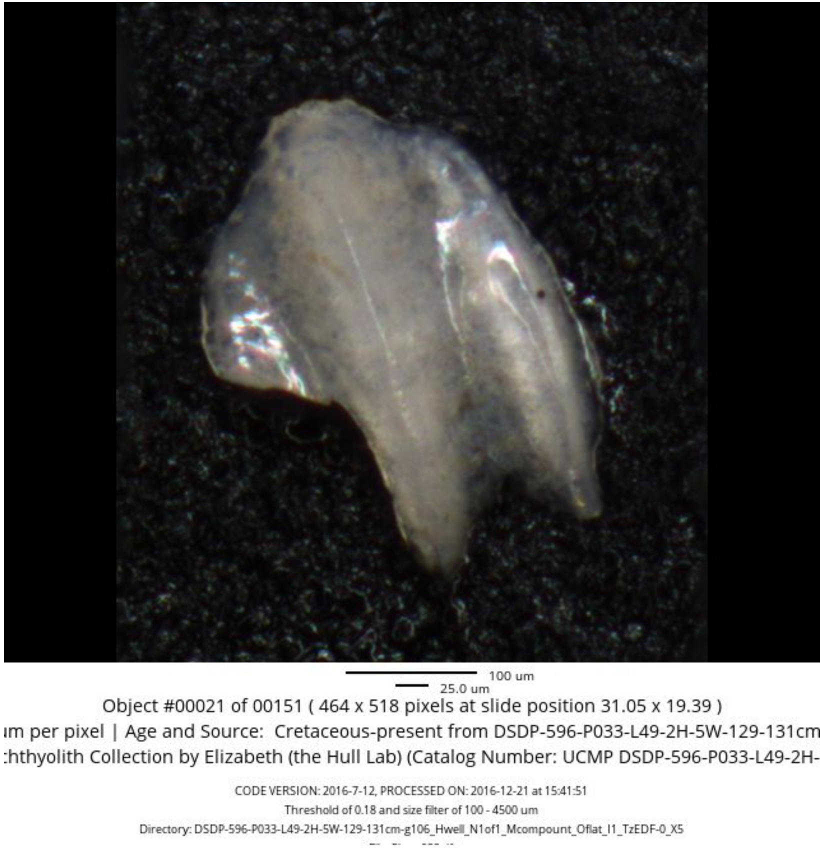

DSDP-596-P033-L49-2H-5W-129-131cm-g106_Hwell_N1of1_obj00021

Big Three Ridge

The big three ridge type has a pointed spade shape with three triangular ridges. The central ridge is wider, taller, and longer than the two side ridges. Each ridge defines a cusp which have triangular ridge profiles.

Fossil Only

Bilateral Fan

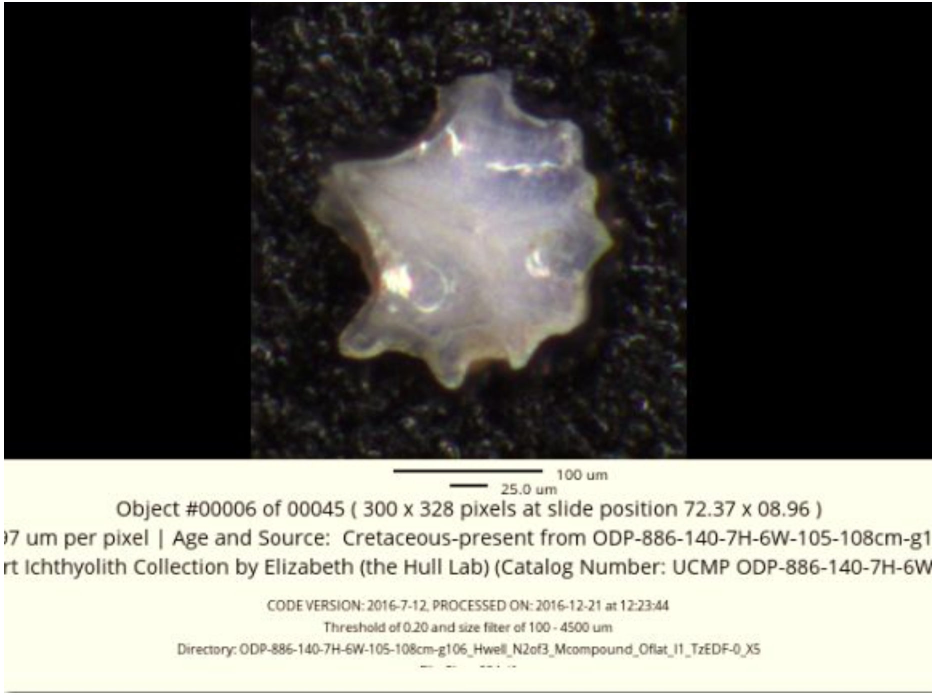

ODP-886-140-7H-6W-105-108cm-g106_obj00006_edf

The bilateral fan has a fan shape with a central ridge which branches out from the anterior into two ridges each diverging to the posterior edge of the crown.

Fossil Only

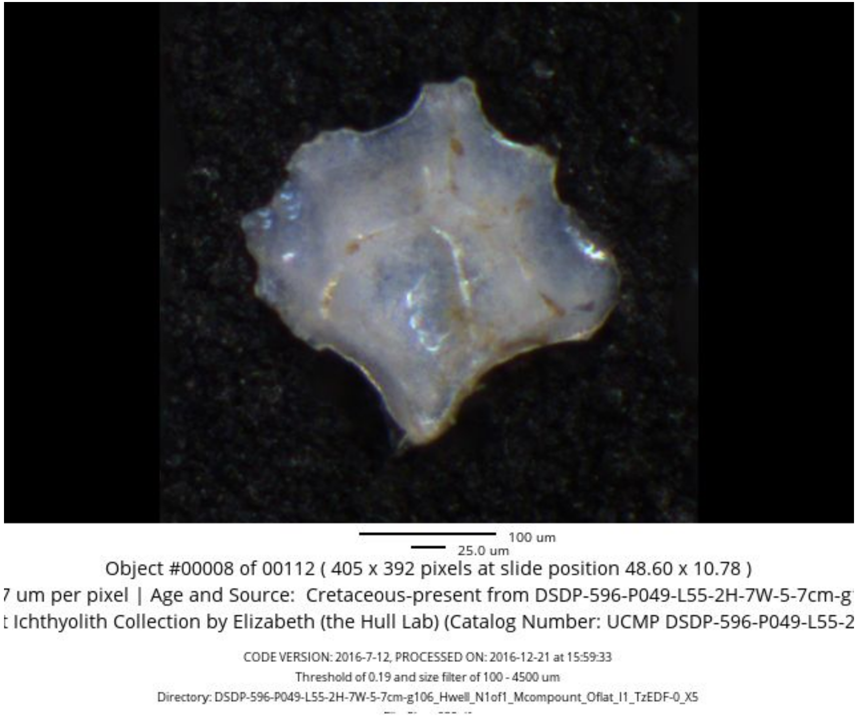

DSDP-596-P025-L45-2H-5W-32-34cm-g106_Hwell_N1of1_obj00008

Bilateral Polygon

The bilateral polygon type has a fan shape with an anterior ridge system shape which outlines a tear drop shaped dimple. Surrounding the system shape are ridges which branch off to the posterior edge of the crown.

Fossil Only

Bilateral Single Ridged

The bilateral single ridged type has an irregular spade shape with a very shallow central meandering ridge. It is thinner at its anterior and widens at its posterior.

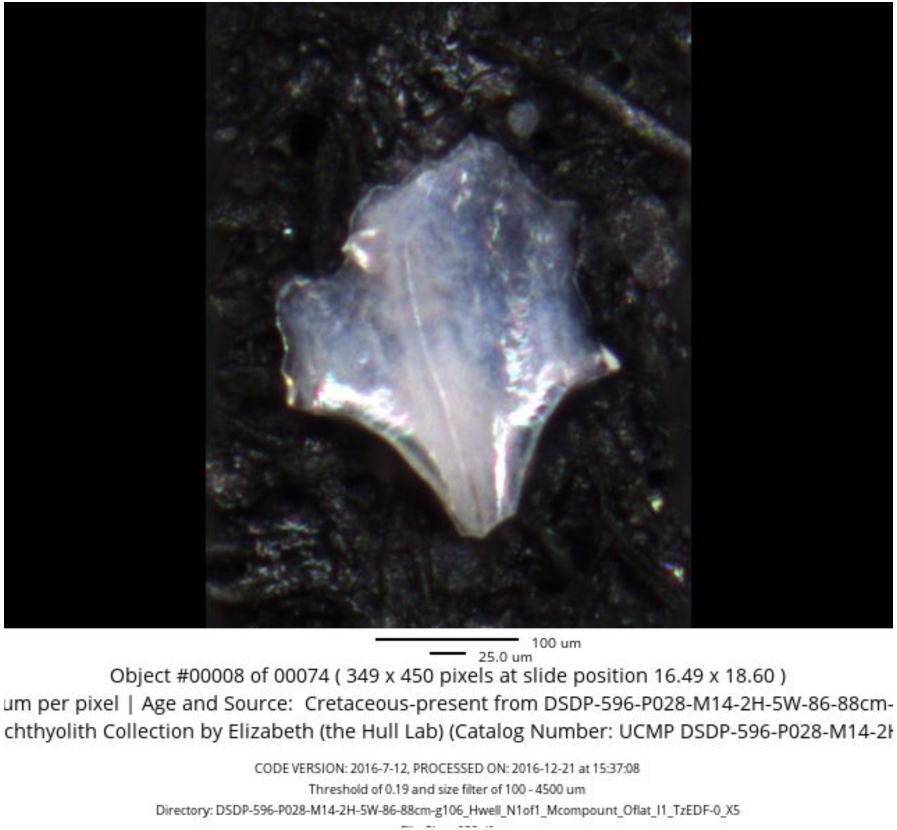

DSDP-596-P028-M14-2H-5W-86-88cm-g106_Hwell_N1of1_obj00008

Fossil Only

Bilateral Single Ridged Arrowhead

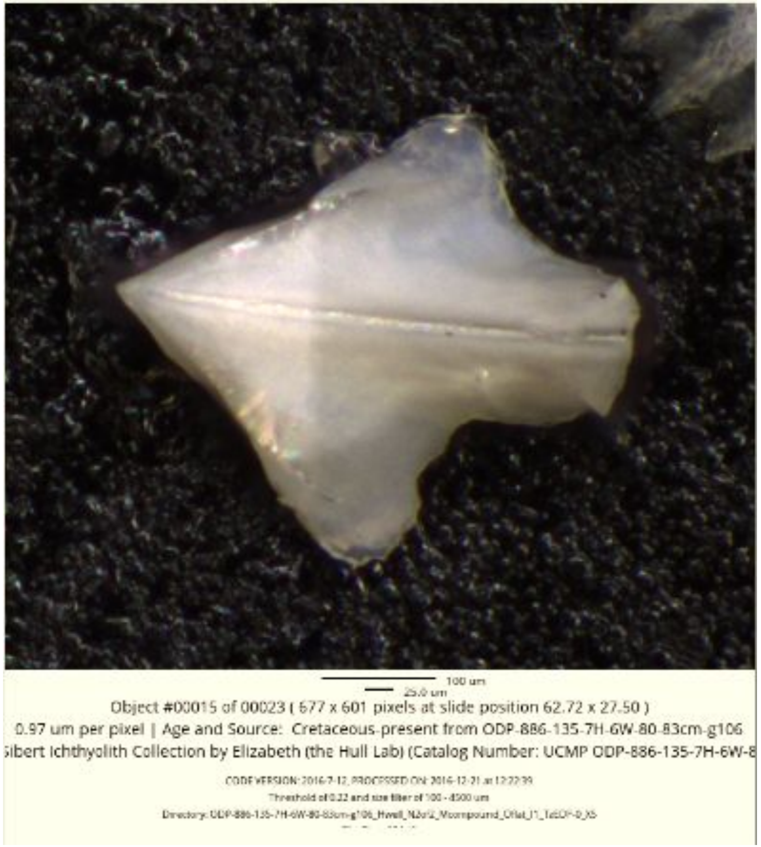

ODP-886-135-7H-6W-80-83cm-g106_obj00015_edf

The bilateral single ridged arrowhead type has an arrow shape with a pointed posterior and two side flanges on either side of a rectangular anterior with a flat smooth edge. The crown has one thin trough that runs the length of the denticle from anterior to posterior.

Fossil Only

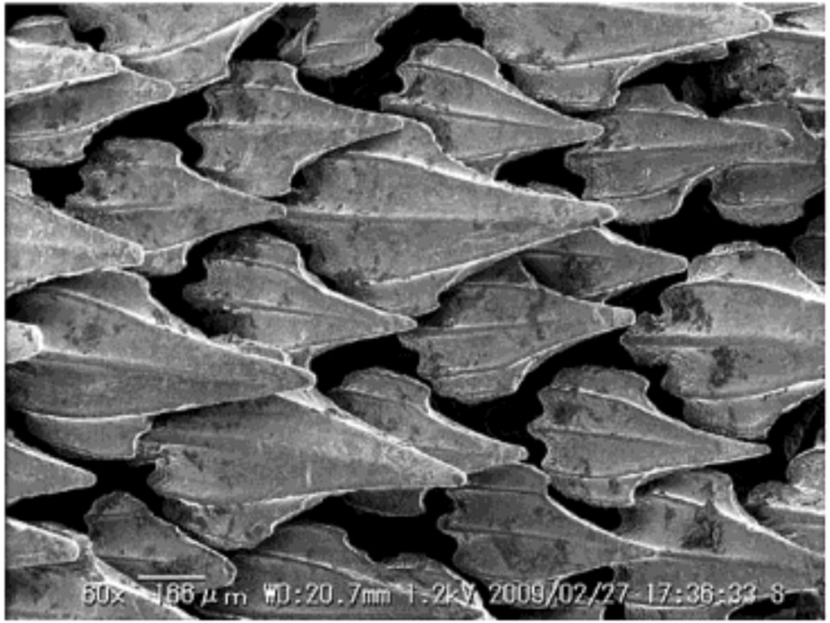

Fig. 89e Dermal denticles.

Scyliorhinus retifer, Castro, Pg. 342, Fig. 89e

Bottom Bulbed Three Ridged Petal

The bottom bulbed three ridged petal has a stretched spade shape with a round anterior and posterior vertex. There are three ridges with a central ridge which bisects the crown and extends its length. The side ridges converge from the anterior and create the posterior vertex. On either side of the crown between the edges and side ridges is an area of flat unridged crown.

Taxonomic Citation

Scyliorhinus retifer, Castro, Pg. 342, Fig. 89e
Scyliorhinus torrei, Castro, Pg. 347, Fig. 91e
Scyliorhinus canicula, Reif, Pg. 159, Fig. H2
Scyliorhinus canicula, Reif, Pg. 159, Fig. B1
Scyliorhinus canicula, Reif, Pg. 157, Fig. H3

Modern Only

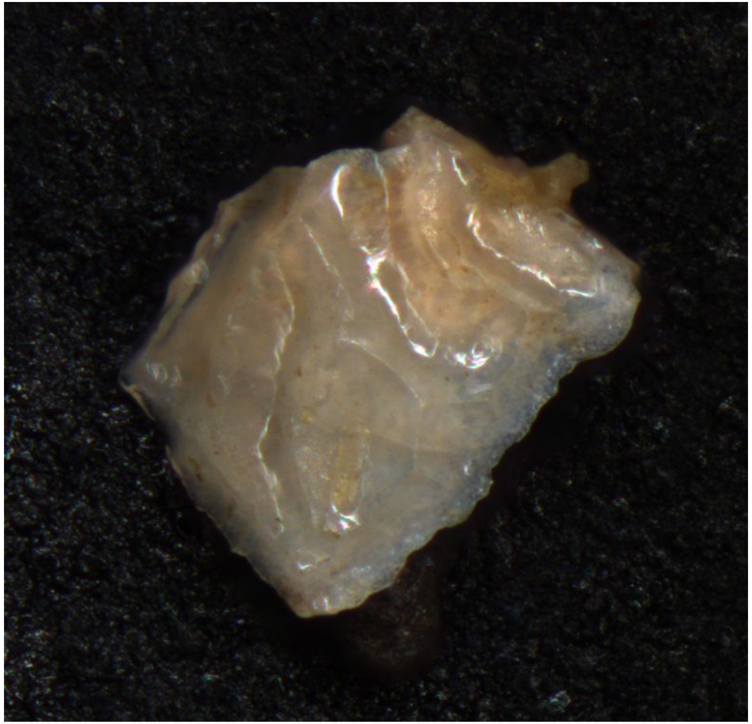

100 um
25.0 um
Object #00060 of 00239 (792 x 824 pixels at slide position 80.33 x 23.33)
0.97 um per pixel | Age and Source: Cretaceous-present from DSDP-596-P020-L39-2H-4W-31-33cm-g106
:d at Sibert Ichthyolith Collection by Elizabeth (the Hull Lab) (Catalog Number: UCMP DSDP-596-P020-L39-2H-4W-31-33c

CODE VERSION: 2016-7-12, PROCESSED ON: 2016-12-21 at 15:32:37
Threshold of 0.12 and size filter of 100 - 4500 um
Directory: DSDP-596-P020-L39-2H-4W-31-33cm-g106_Hwell_N1of1_Mcompount_Oflat_I1_TzEDF-0_X5

DSDP-596-P020-L39-2H-4W-31-33cm-g106_Hwell_N1of1 _obj00060

Branching and Flared Chunk

The branching and flared chunk has a rectangular shape and is defined by shallow meandering and branching ridges and troughs randomly distributed on the crown.

Fossil Only

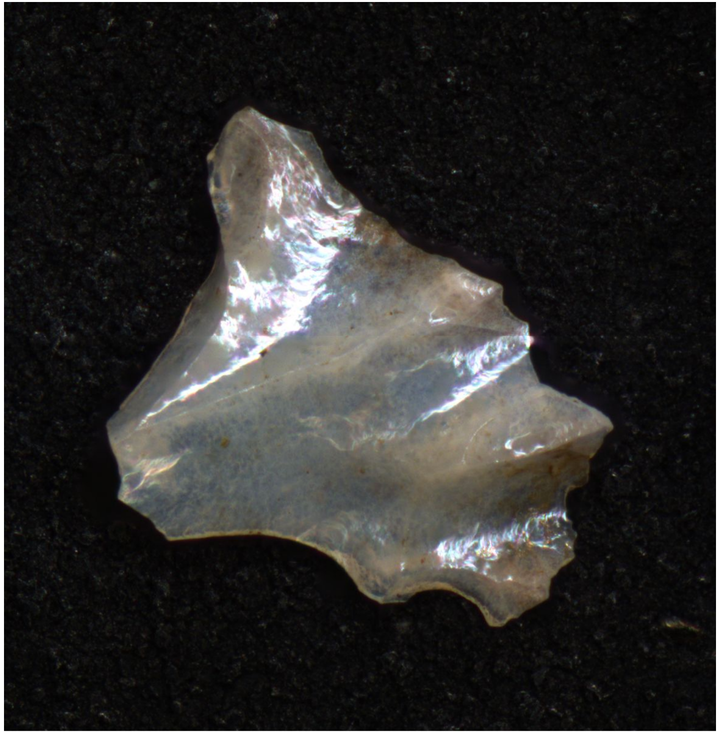

100 um
25.0 um
Object #00082 of 00266 (1167 x 1190 pixels at slide position 85.73 x 38.42)
0.97 um per pixel | Age and Source: Cretaceous-present from DSDP-596-P021-L42-2H-4W-105-107cm-g106
Processed at Sibert Ichthyolith Collection by Elizabeth (the Hull Lab) (Catalog Number: UCMP DSDP-596-P021-L42-2H-4W-105-107cm-g106)

CODE VERSION: 2016-7-12, PROCESSED ON: 2016-12-21 at 15:32:56
Threshold of 0.16 and size filter of 100 - 4500 um
Directory: DSDP-596-P021-L42-2H-4W-105-107cm-g106_Hwell_N1of1_Mcompount_Oflat_I1_TzEDF-0_X5

DSDP-596-P021-L42-2H-4W-105-107cm-g106_Hwell_N1of1_obj00082

Branching Dust Pan

The branching dust pan has a fan shape with a smooth rectangular anterior and a wider flared posterior, which has ridges with triangular profiles.

Fossil Only

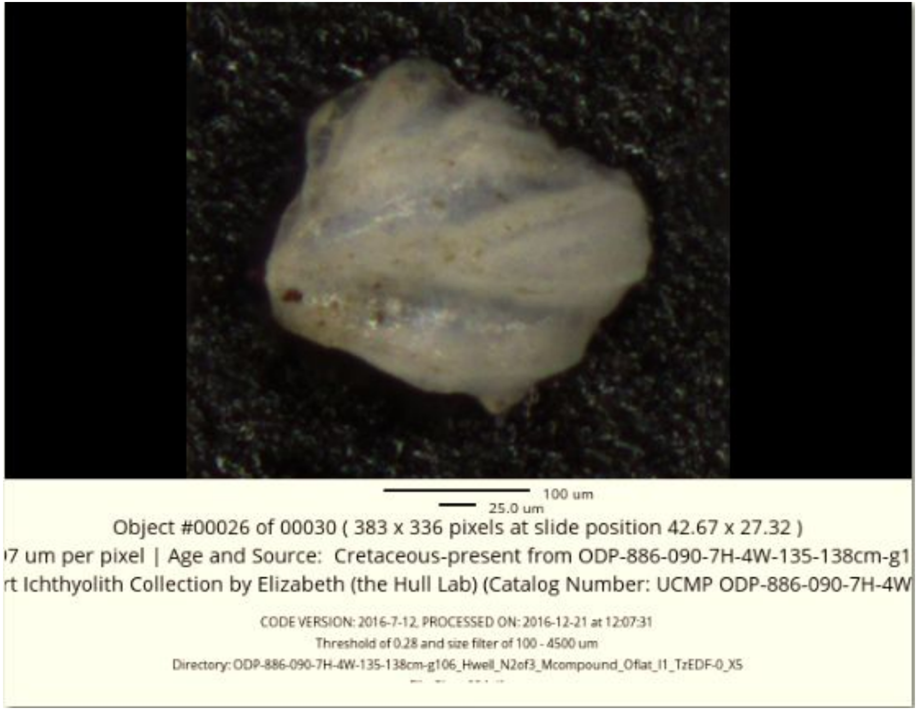

ODP-886-090-7H-4W-135-138cm-g106_obj00026_edf

Branching Fan

The branching fan type has a fan shape with an anterior vertex and multiple ridges which diverge and branch into more ridges reaching the round posterior edge.

Taxonomic Citation

Carcharhinus plumbeus, Reif, Pg. 190, Fig. P1

Branching Fragment

The branching fragment type is a catchall for broken denticles that have some aspect of branching ridges.

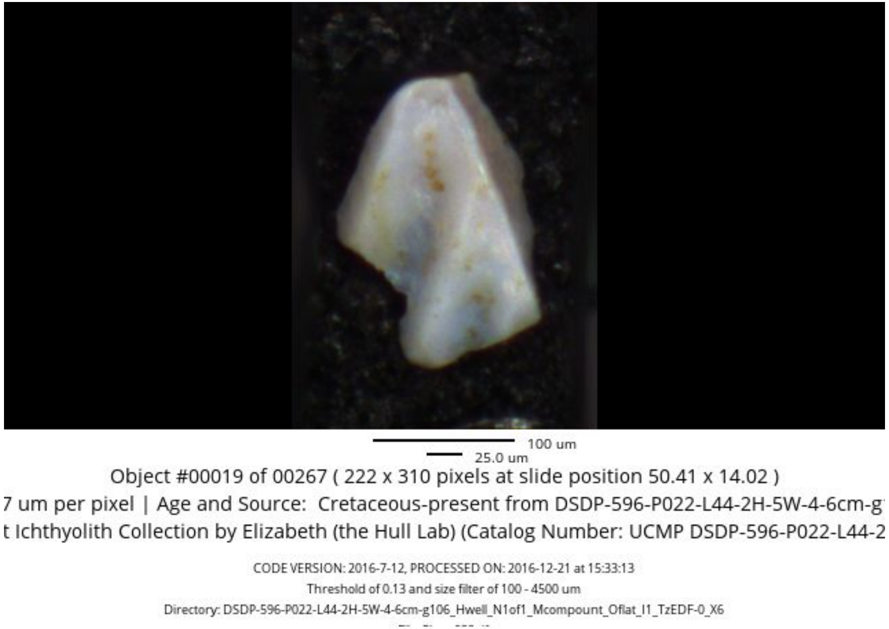

DSDP-596-P022-L44-2H-5W-4-6cm-g106_Hwell_N1of1_obj00019

Fossil Only

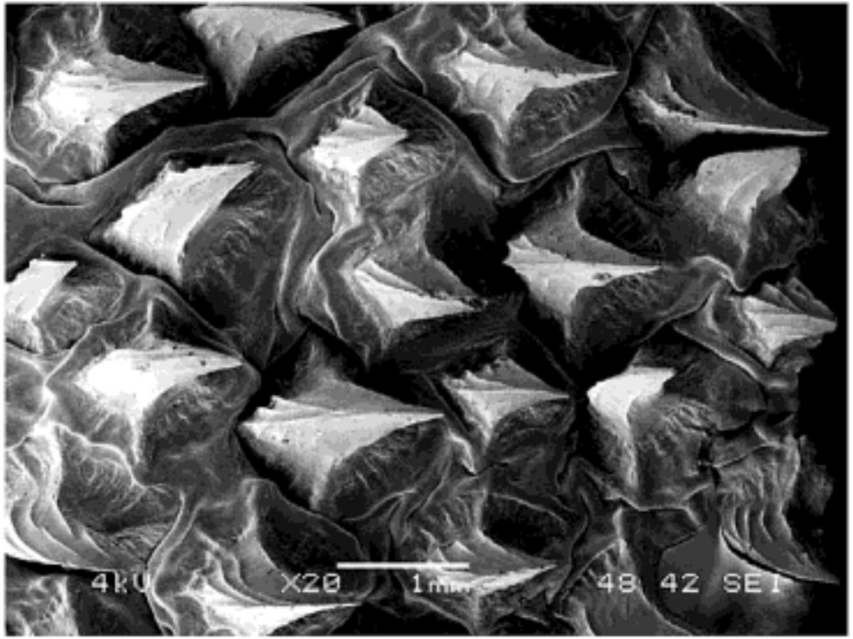

Fig. 33e Dermal denticles.

Somniosus rostratus, Castro, Pg. 135, Fig. 33e

Branching Triangle

The branching triangle type has a triangular shape and is defined by a a central ridge which bisects the crown and which has one to three branching ridges which converge from the anterior to the central ridge where they terminate.

Taxonomic Citation

Somniosus rostratus, Castro, Pg. 135, Fig. 33e

Modern Only

Bunny Ears

The bunny ears type has two elongated dimples on either side of a central ridge which bisects the crown.

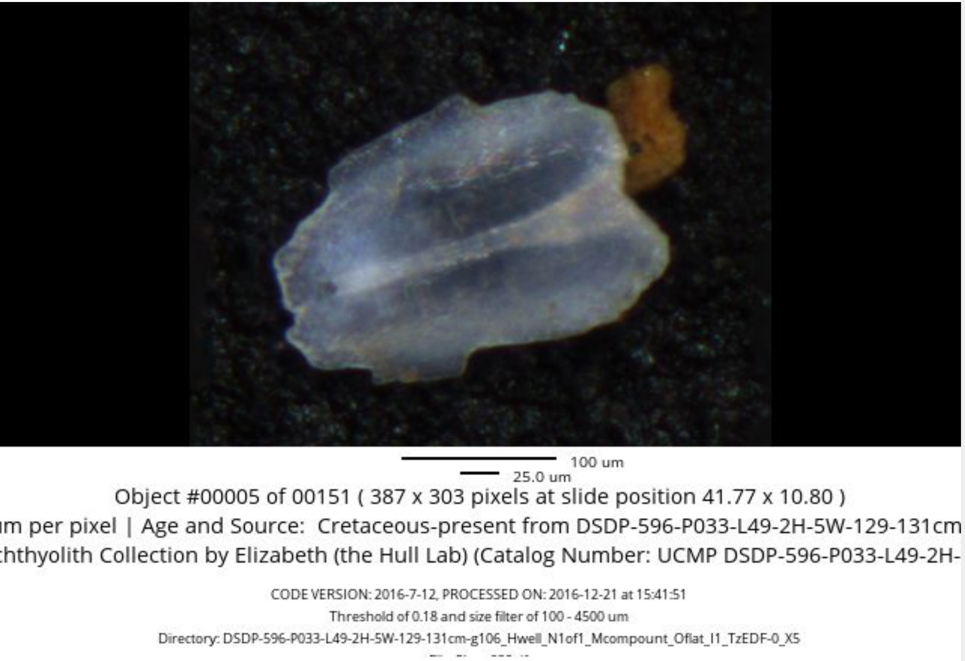

DSDP-596-P033-L49-2H-5W-129-131cm-g106_Hwell_N1of1_obj00005

Fossil Only

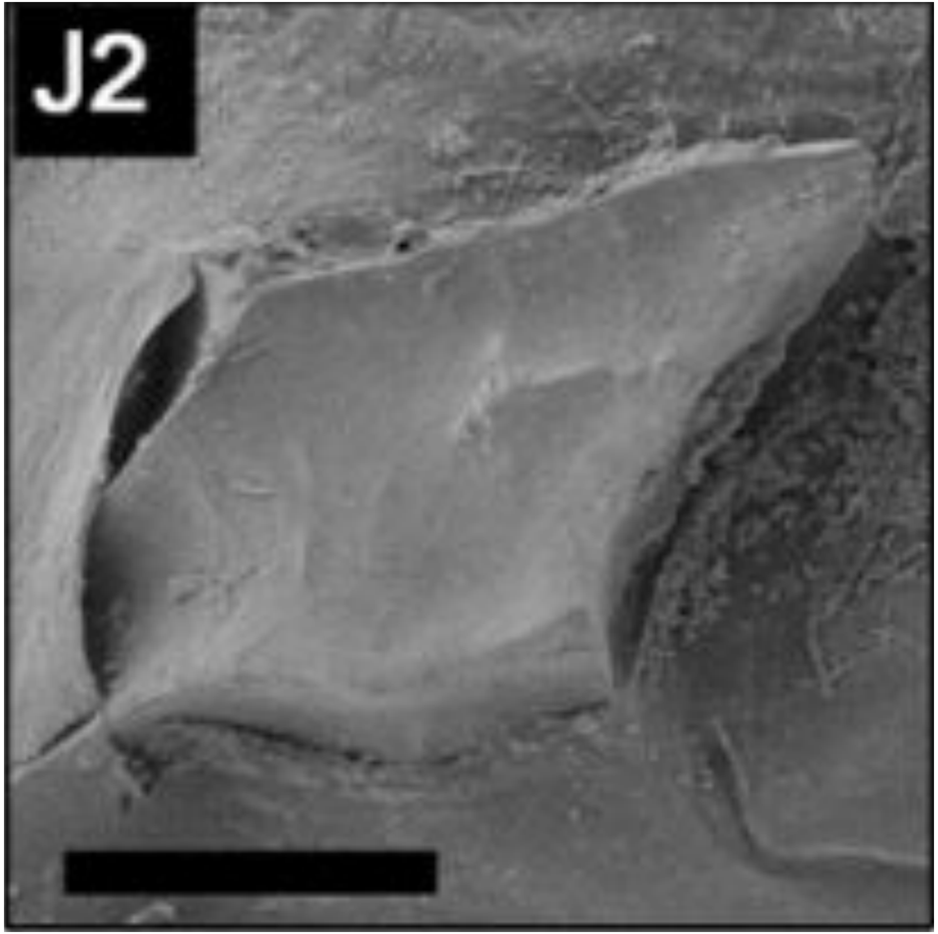

Etmopterus pusillus, Feichtinger et al., Fig. J2

Caldera Diamond

The caldera diamond type has a diamond-like shape and is defined by a large depression at the center of four edge ridges which define the shape of the crown.

Taxonomic Citation

Etmopterus pusillus, Feichtinger et al., Fig. J2

Modern Only

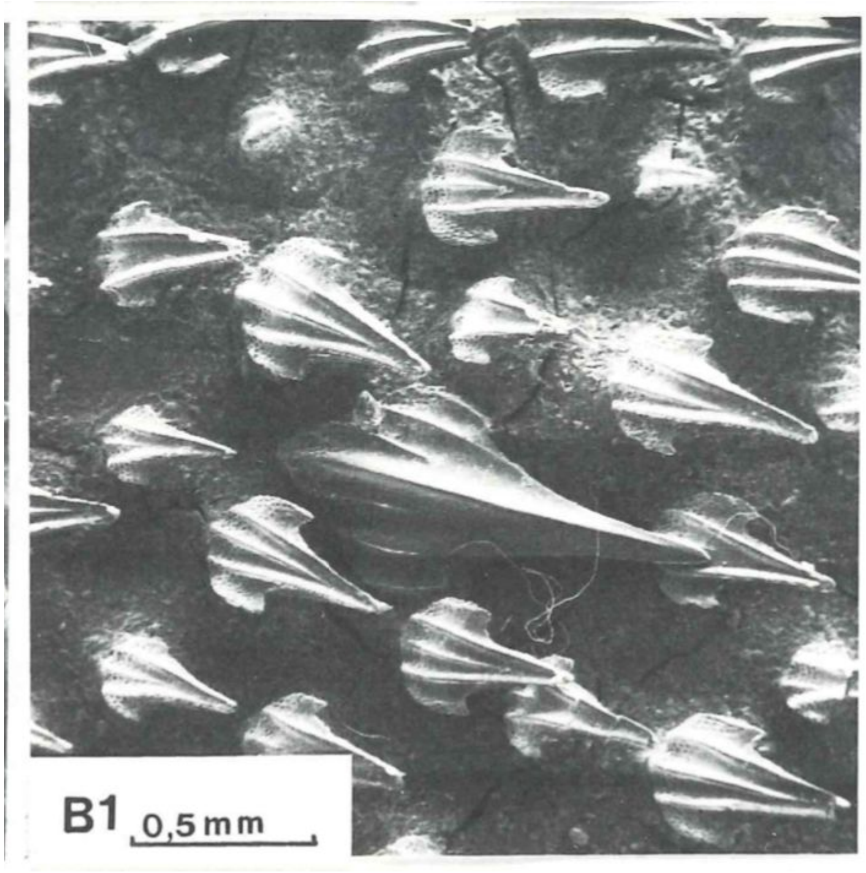

Scyliorhinus canicula, Reif, Pg. 157, Fig. B1

Central Seven Ridged Extended Trident

The central seven ridged extended trident has a pointed spade shape with three cusps and a round anterior. The central cusp extends ~two times the length of the crown and is composed of five ridges, the two side ridges converge at the posterior vertex around the central ridge. The two side cusps are each composed of one ridge which define the edges of the crown.

Taxonomic Citation

Scyliorhinus canicula, Reif, Pg. 157, Fig. B1
Scyliorhinus sp., Castro, Pg. 346, Fig. 90c
Scyliorhinus stellaris, Reif, Pg. 161, Fig. B1
Scyliorhinus stellaris, Reif, Pg. 161, Fig. B3
Scyliorhinus retifer, Gabler-Smith et al., Fig. E

Modern Only

Central Ridged Diamond

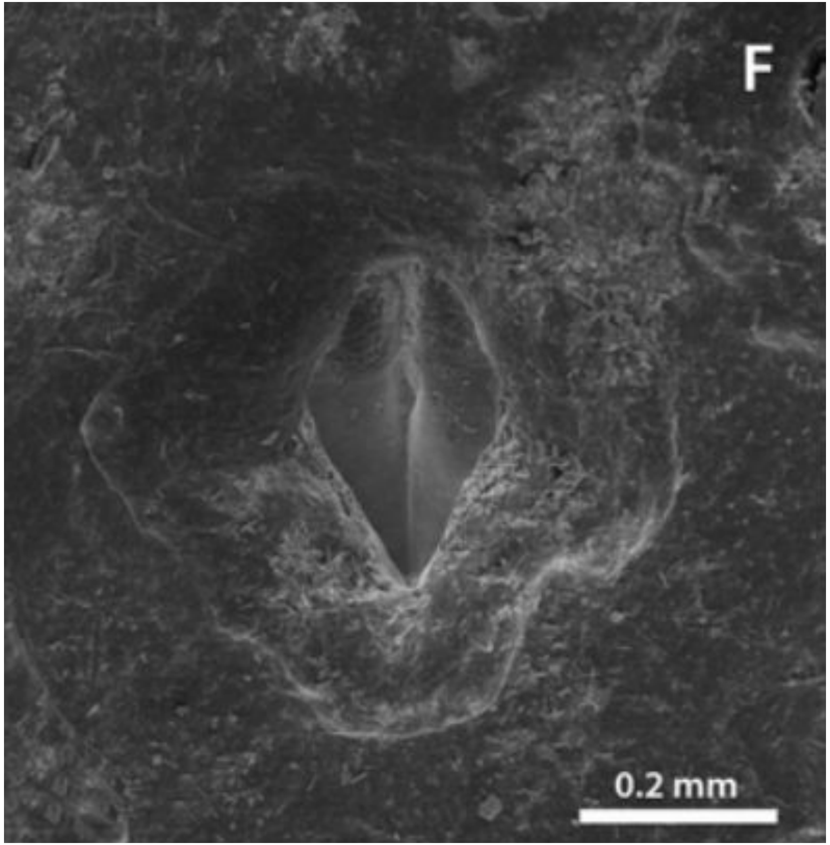

Squatina argentina, Vaz et al. (2013), Fig. F

The central ridged diamond has a diamond-like shape with a single central ridge which bisects the length of the crown. The crown is longer from posterior to anterior than it is wide.

Taxonomic Citation

Squatina argentina, Vaz, D. F., & De Carvalho, M. R. (2013), Fig. F

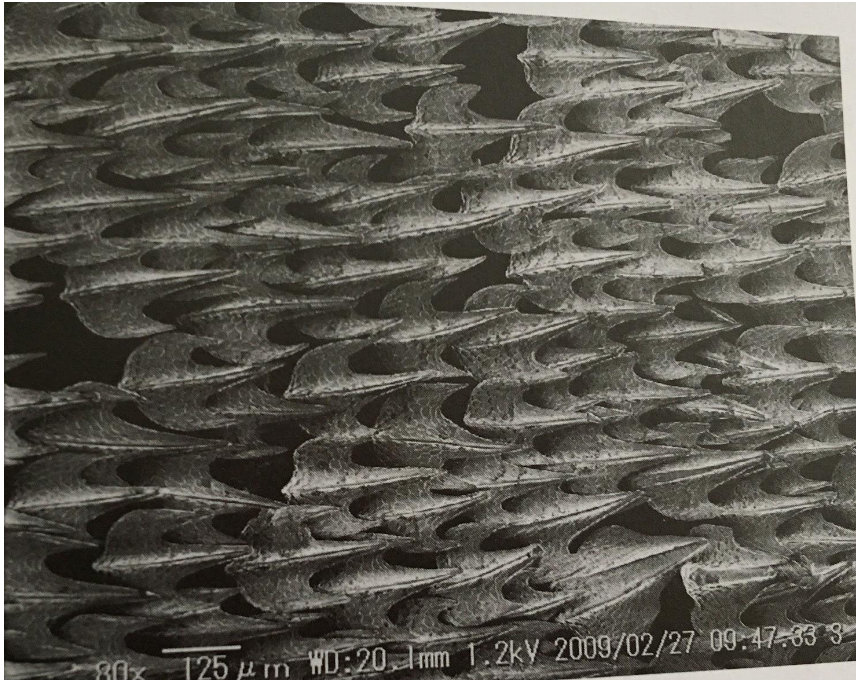

Galeus springeri, Castro, Pg. 326

Central Ridged Extended Trident

The central ridged extended trident has a pointed spade shape with three cusps defined by ridges and a round anterior. The central cusp is longer than the side cusps and has the highest ridge.

Taxonomic Citation

Alopias superciliosus, Reif, Pg. 143, Fig. P1
Galeus melastomus, Reif, Pg. 155, Fig. B4
G. piperatus, Castro, Pg. 328
G. springeri, Castro, Pg. 326
Isurus oxyrinchus, Reif, Pg. 147, Fig. C2
Oxynotus centrina, Reif, Pg. 120, FIg. P3
Prionace glauca, Reif, Pg. 215, Fig. B6
Rhincodon typus, Castro, Pg. 194
Scyliorhinus canicula, Reif, Pg. 157, Fig. B3 and H3
S. retifer, Reif, Pg. 163, Fig. H2 and P2
S. stellaris, Reif, Pg. 161, Fig. C2

Central Ridged Petal

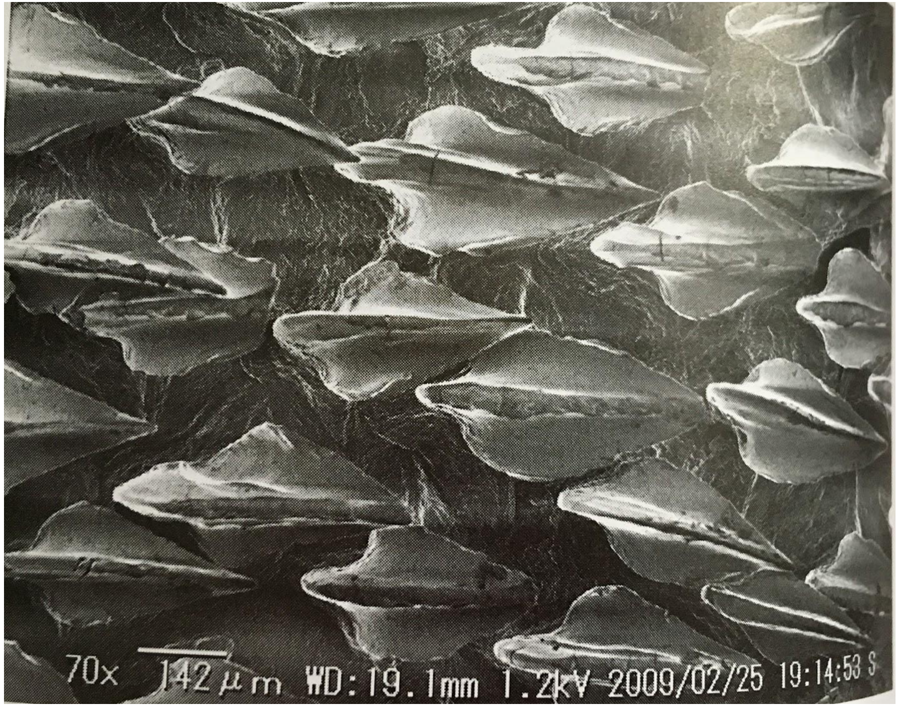

Pseudotriakis microdon, Castro, Pg. 353, Fig. 93e

The central ridged petal has a triangular shape with a central ridge which bisects the crown and extends its length, defining its anterior and reaching the posterior vertex.

Taxonomic Citation

Pseudotriakis microdon, Castro, Pg. 353, Fig. 93e

Squalus acanthias, Reif, Pg.116, Fig. M1

Modern Only

Central Ridged Triangle

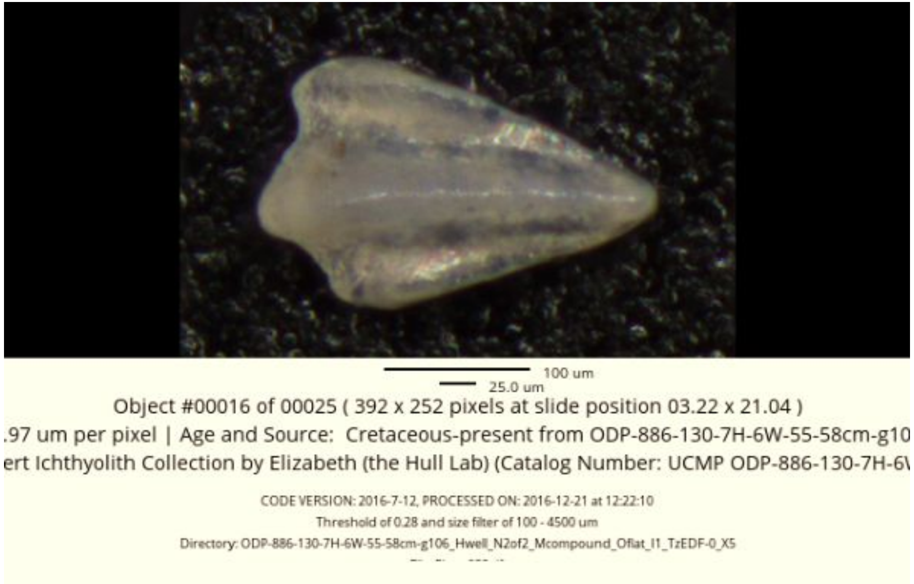

ODP-886-130-7H-6W-55-58cm-g106_obj00016_edf

The central ridged triangle has a triangular shape and a central ridge which extends the length of the crown. The side edges of the crown are defined by shorter ridges which converge from the anterior to a vertex at the posterior.

Taxonomic Citation

Dalatias licha, Castro, Pg. 142

Squatina squatina, Reif, Pg. 126, Fig. H2

Mitsukurina owstoni, Reif, Pg. 163, Fig. M1

Central Troughed Petal

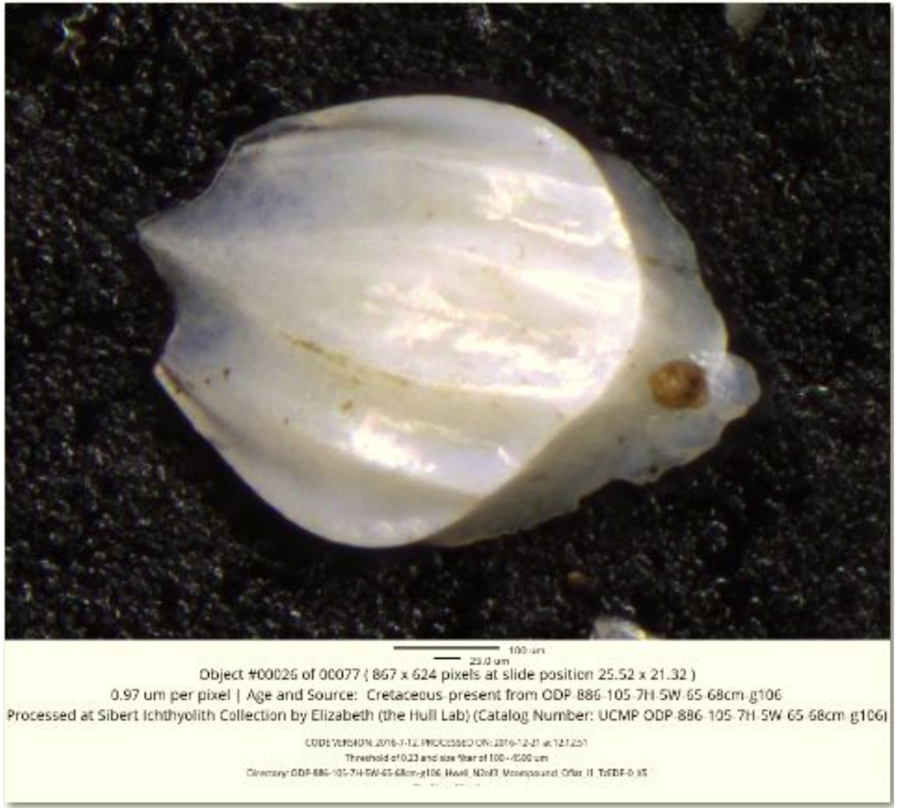

ODP-886-105-7H-5W-65-68cm-g106_obj00026_edf

The central troughed petal type has a rounded spade shape with two central ridges which diverge towards the center and then converge creating a central trough which extends the length of the denticle and ends in a pointed posterior. On either side of the central trough ridges are a straight ridge which do not define the sides of the denticle.

Taxonomic Citation

Carcharhinus obscurus, Reif, Pg. 185, Fig. P3
C. plumbeus, Reif, Pg. 192, Fig. P1
Mustelus canis, Ankhelyi et al., Pg. 5, Fig. c
M. lunulatus, Castro, Pg. 377
M. mustelus, Reif, Pg. 167, Fig. H2
M. norrisi, Castro, Pg. 379
Squatina japonica, Reif, Pg. 128, Fig. B1
Triaenodon obesus, Reif, Pg. 212, Fig. C3
Galeocerdo cuvier, Reif, Pg. 227, Fig. M1

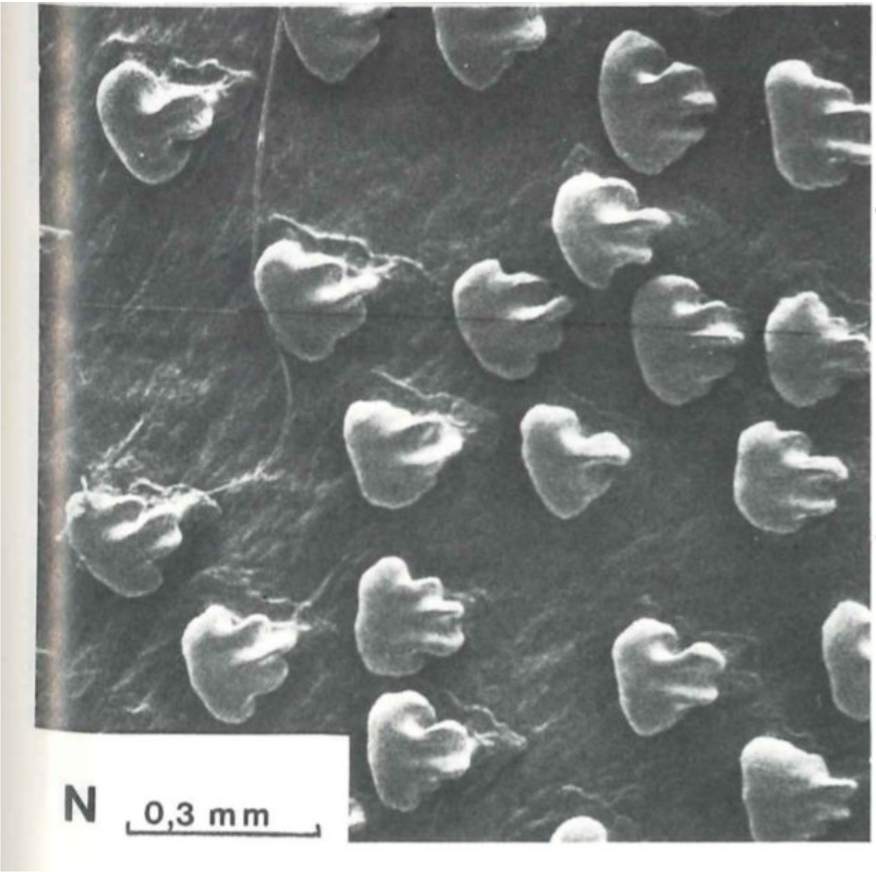

Galeocerdo cuvier, Reif, Pg. 227, Fig. N

Cheeky

The cheeky denticle type has an irregular shape which is wider than long and has a round anterior and a number of rounded cusps which create a scalloped posterior edge texture.

Taxonomic Citation

Galeocerdo cuvier, Reif, Pg. 227 and 229 Fig. N

Modern Only

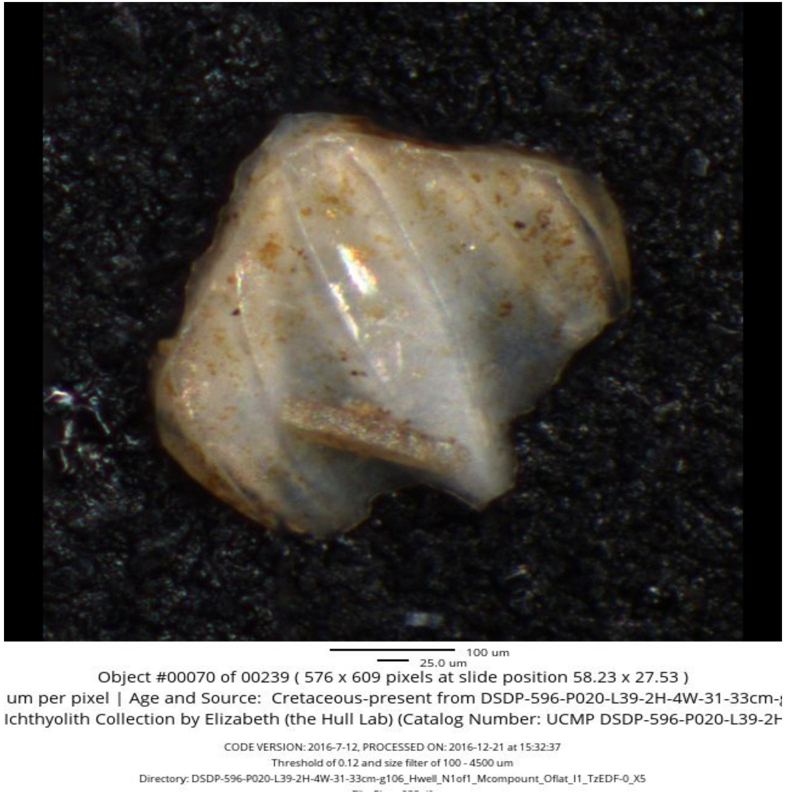

DSDP-596-P020-L39-2H-4W-31-33cm-g106_Hwell_N1of1_obj00070

Chubby Trident

The chubby trident has a rounded spade shape with a round anterior and three cusps. Each cusp is defined on either side by one or more ridges. The side cusps are thinner than the central cusp and curve slightly toward the center.

Taxonomic Citation

Lamna nasus, Reif, Pg. 149, Fig. H3
Prionace glauca, Reif, Pg. 217, Fig. H3

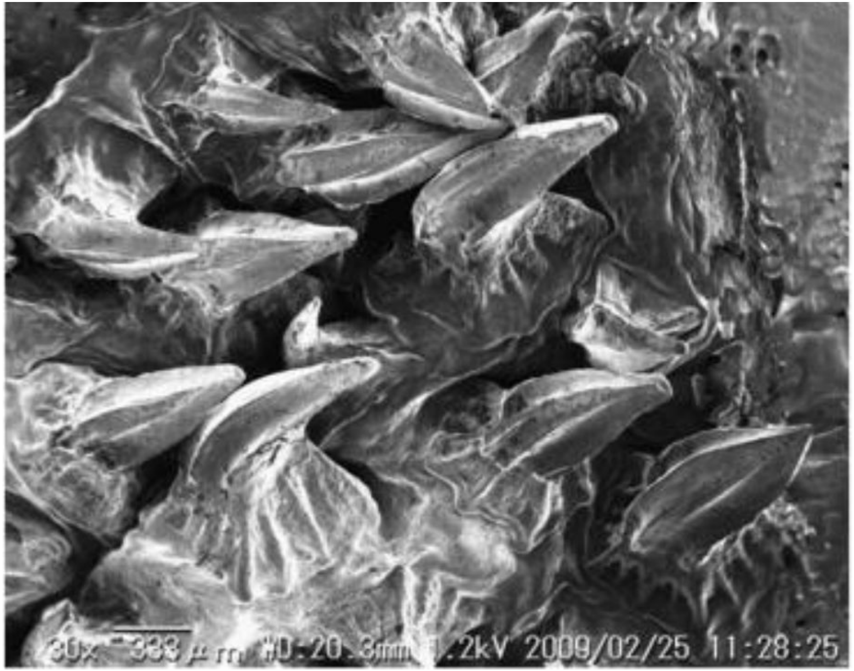

Somniosus pacificus, Castro, Pg. 131

Circular Tipping Spines

The circular tipping spine type has a spine shape with a crown that emerges from the skin at a vertical angle and then curves horizontally over the skin surface. The crown has multiple ridges which extend from the anterior to the posterior of the crown.

Taxonomic Citation

Squatina varii, Vaz & Carvalho, Fig. D

Etmopterus hillianus, Castro, Pg. 105

Etmopterus virens, Castro, Pg. 112

Somniosus pacificus, Castro, Pg. 131

Mitsukurina owstoni, Castro, Pg. 202

Cephalurus cephalus, Castro, Pg. 321

Raja clavata, Gravendeel et a., Fig. 8: ventral buckler, lateral buckler, alar thorn, malar buckler, malar thorn, Fig. 9: median, orbital, fin, and Fig. 10: pectoral fin, straight leaf type prickle

Raja microocellata, Gravendeel et al., Fig. 11 cross type prickle, Fig. 12 claw type prickle and prickle

Modern Only

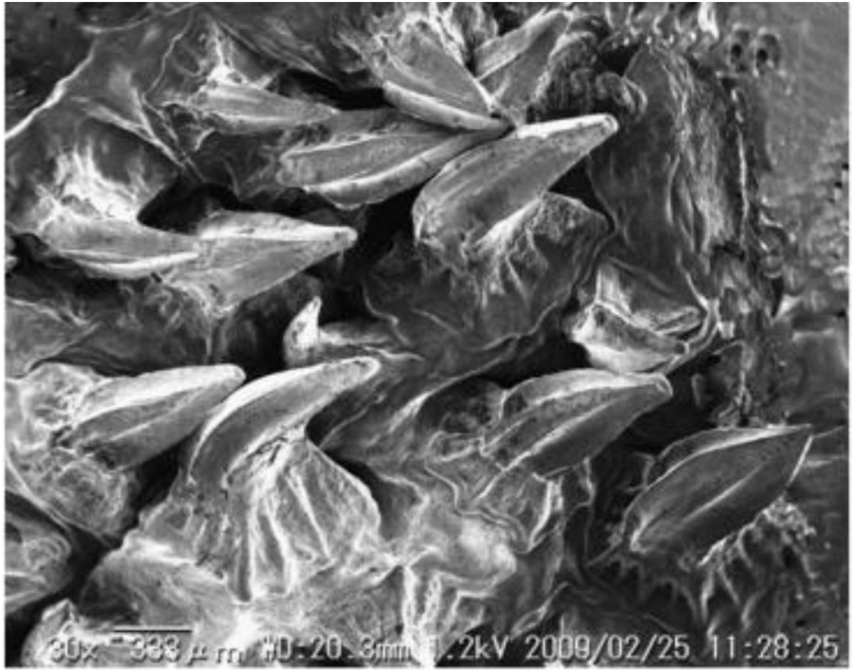

Somniosus pacificus, Castro, Pg. 131

Circular Tipping Spines

The circular tipping spine type has a spine shape with a crown that emerges from the skin at a vertical angle and then curves horizontally over the skin surface. The crown has multiple ridges which extend from the anterior to the posterior of the crown.

Taxonomic Citation

Raja montagui, Gravendeel et al., Fig. 13: parallel, alar, suprascapular, and Fig. 14: pectoral fin and orbital

Raja undulata, Gravendeel et al., Fig. 17: pectoral fin and claw type prickle

Dipturus batis, Gravendeel et al., Fig. 18: cross type

Leucoraja circularis, Gravendeel et al., Fig. 22 pectoral fin

Amblyraja radiata, Gravendeel et al., Fig. 26 pectoral fin and alar

Manta birostris, Marshall, Compagno, and Bennett, Fig. 6 (C)

Etmopterus bullisi, Reif, Pg. 108, Fig. B2

Etmopterus spinax, Reif, Pg. 110, Fig. C2

Squatina occulta, Vaz & Carvalho Fig. F

Squatina guggenheim, Vaz & Carvalho, Fig. C

Etmopterus lucifer, Lourtie et al. 2022

Modern Only

Circular Petal

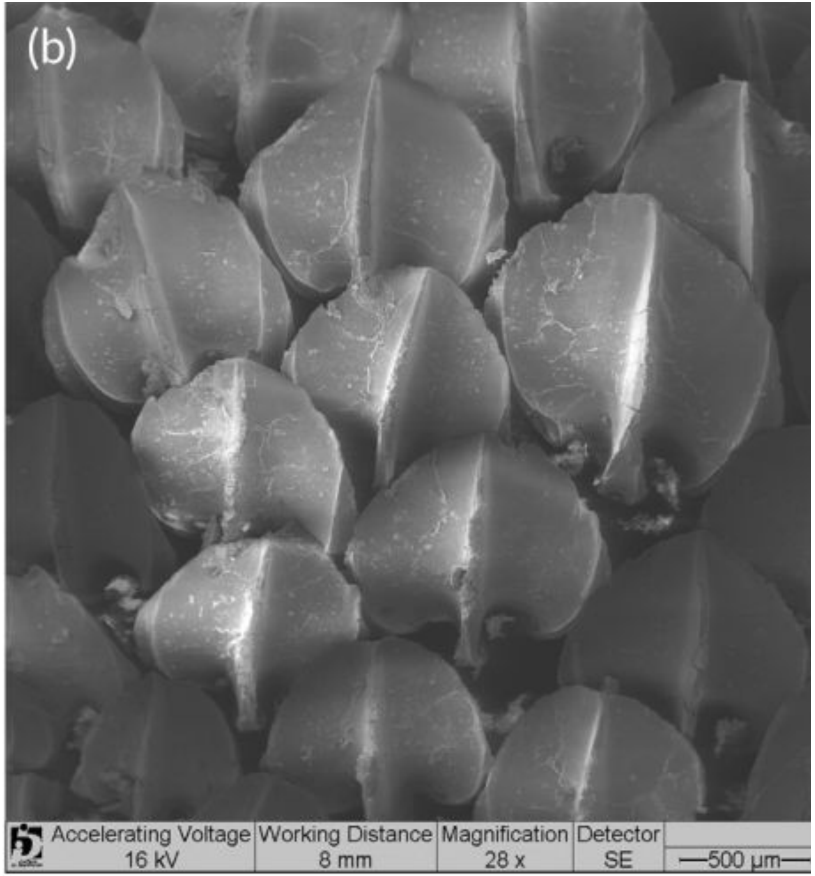

Scymnodon macracanthus, Vaz, Fig. 16 b

The circular petal has a circular shape with an anterior vertex and a round posterior. There is a central ridge which bisects the crown and extends its length. On either side of denticle a side ridge defines its edges.

Taxonomic Citation

Scymnodon macracanthus, Vaz, Fig. 16 a and b

Modern Only

Complex Central Ridged Triangle

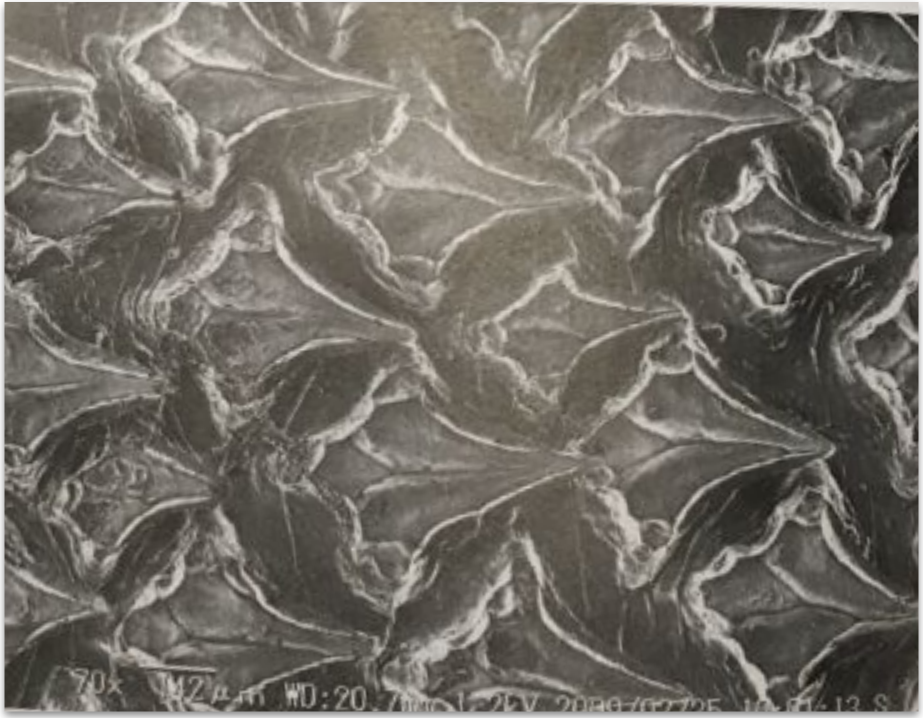

Somniosus rostratus, Castro, Pg. 135, Fig. 35e

The complex central ridged triangle has a cruciform shape and is defined by a central ridge which bisects the crown and extends from the top of a small central ridge system shape, which has four or more edges, at the anterior to the crown's posterior vertex. The anterior central ridge system shape outlines a dimple.

Modern Only

Crinkle Tops

The crinkle top type has a fan-like shape with a straight scalloped posterior and a rounded anterior. The type has three or more ridges with the central ridge being slightly longer and side ridges which get shorter from the center toward the edges.

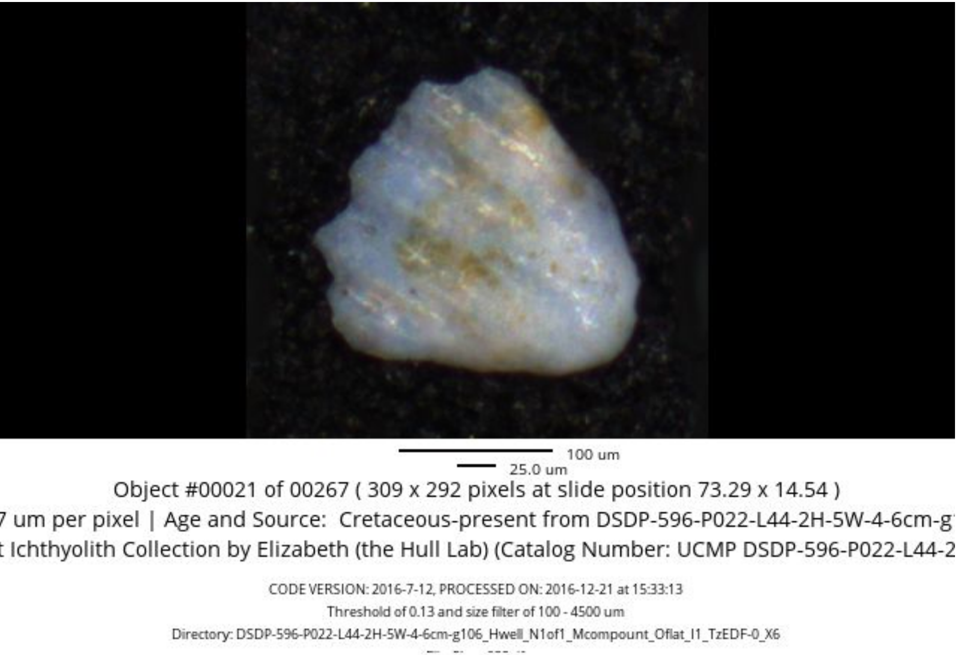

DSDP-596-P022-L44-2H-5W-4-6cm-g106_Hwell_N1of1_obj00021f

Fossil Only

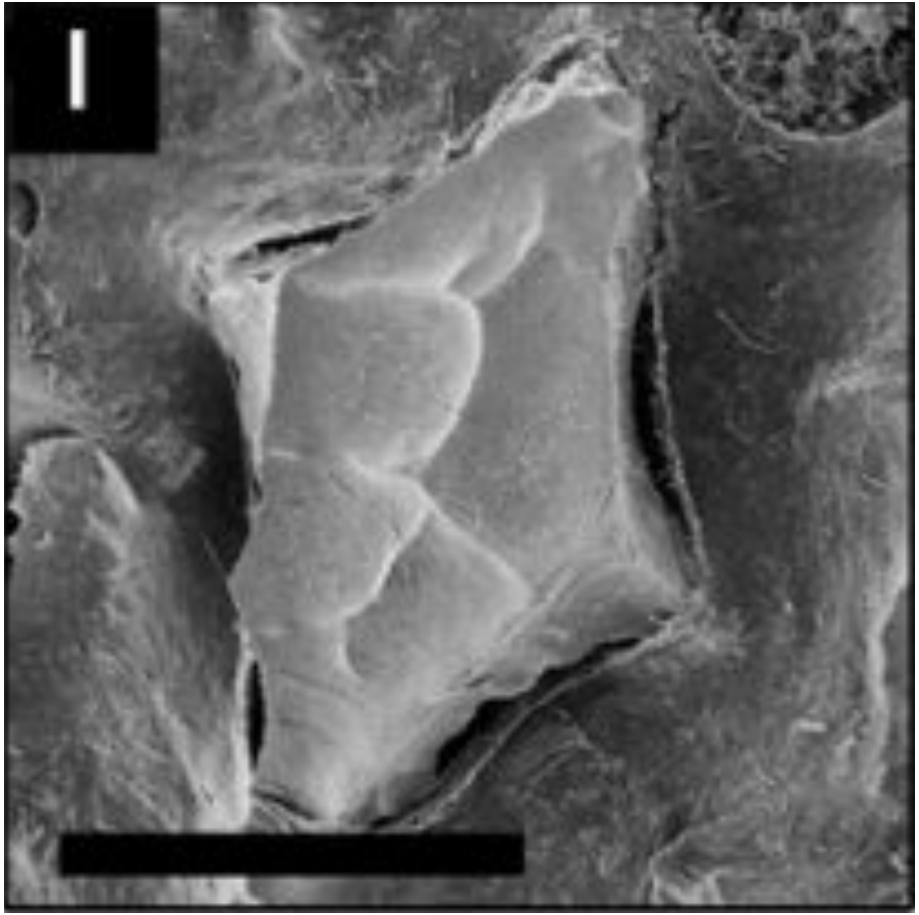

Feichtinger et al. 2021, Fig. I

Cracked Kite

The cracked kite type has a diamond like shape with two ridges which diverge from an anterior vertex where they terminate ~ ½ the length of the crown and two ridges connect to these ridges converging at a posterior vertex. The crown is defined by five or more meandering ridgess is a branching pattern giving the crown a “cracked” appearance.

Modern Only

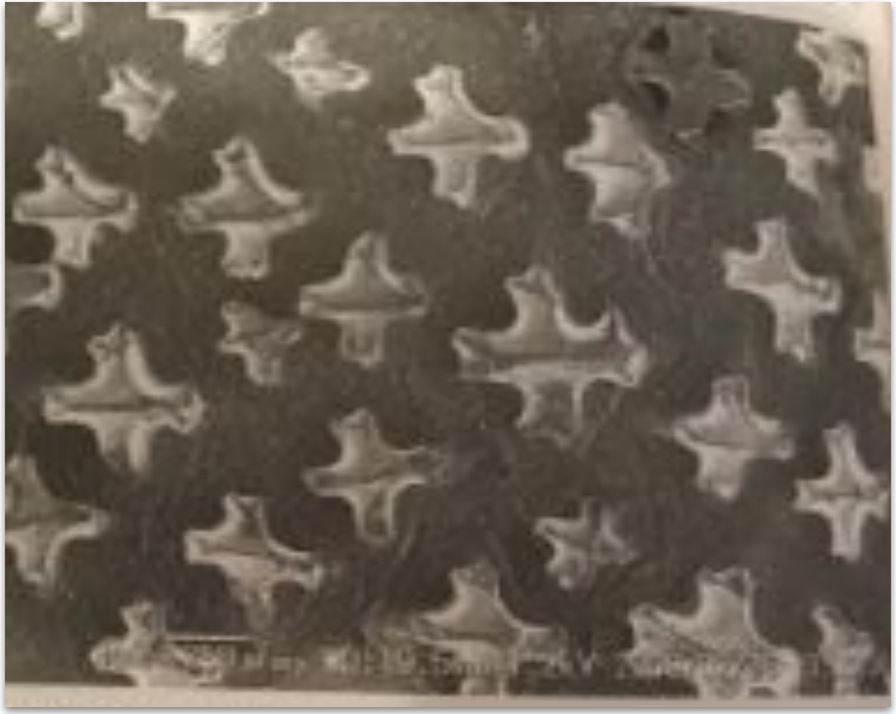

Squatina squatina, Reif, Pg. 126, Fig. M1

Symmetrical Criss Cross

The symmetrical criss cross has an irregular crown shape defined by four ridges with approximate 90 degree angles at their intersection. The crown also lacks directionality.

Taxonomic Citation

Heterodontus francisci, Castro, Pg. 175
H. mexicanus, Castro, Pg. 179

Modern Only

Asymmetrical Criss Cross

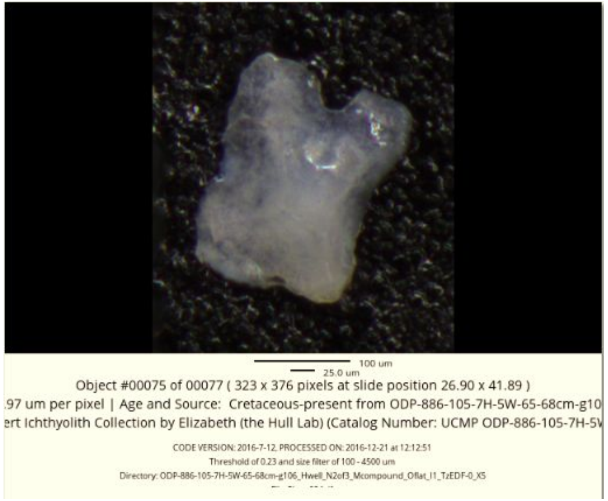

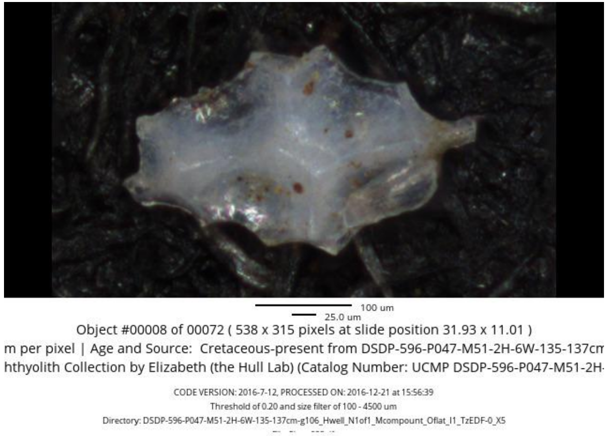

The asymmetrical criss cross has an irregular crown shape defined by four ridges with approximate 90 degree angles between ridges and which radiate from a central shorter ridge. The crown lacks directionality and has no planes of symmetry.

Fossil Only

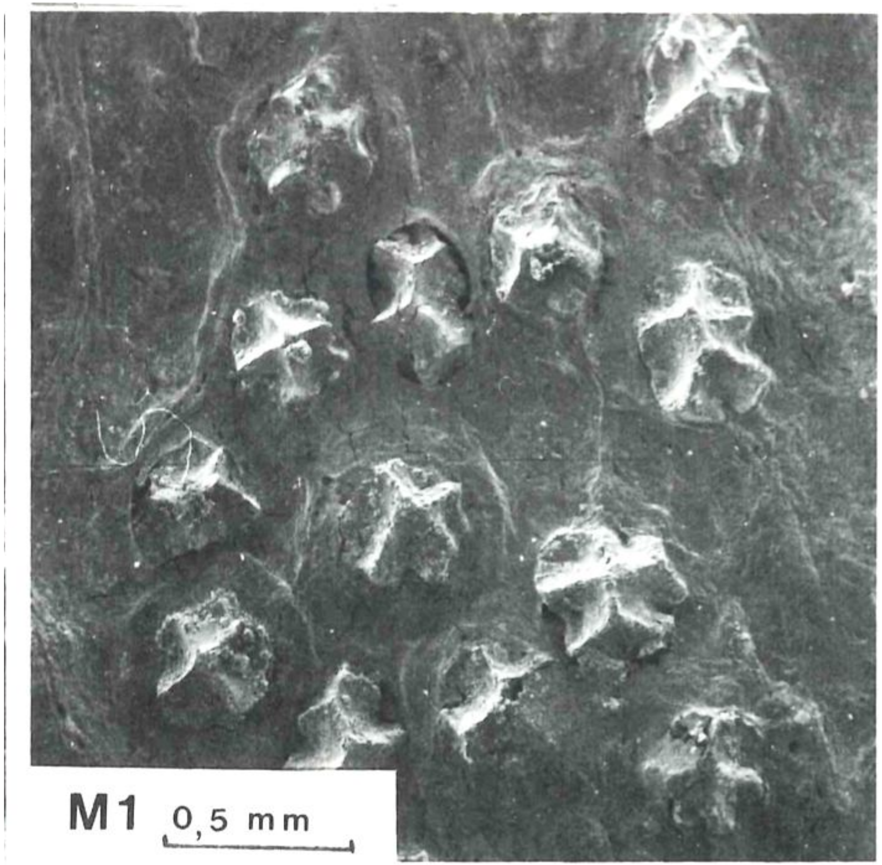

Squatina squatina, Reif, Pg. 126, Fig. M1

Five Ridged Criss Cross

The five ridged criss cross has an irregular crown shape defined by four ridges with approximate 90 degree angles between ridges and which radiate from a shorter central ridge. The crown lacks directionality and may or may not have one plane of symmetry along the length of the shorter central ridge.

Taxonomic Citation

Squatina squatina, Reif, Pg. 126, Fig. M1

Modern Only

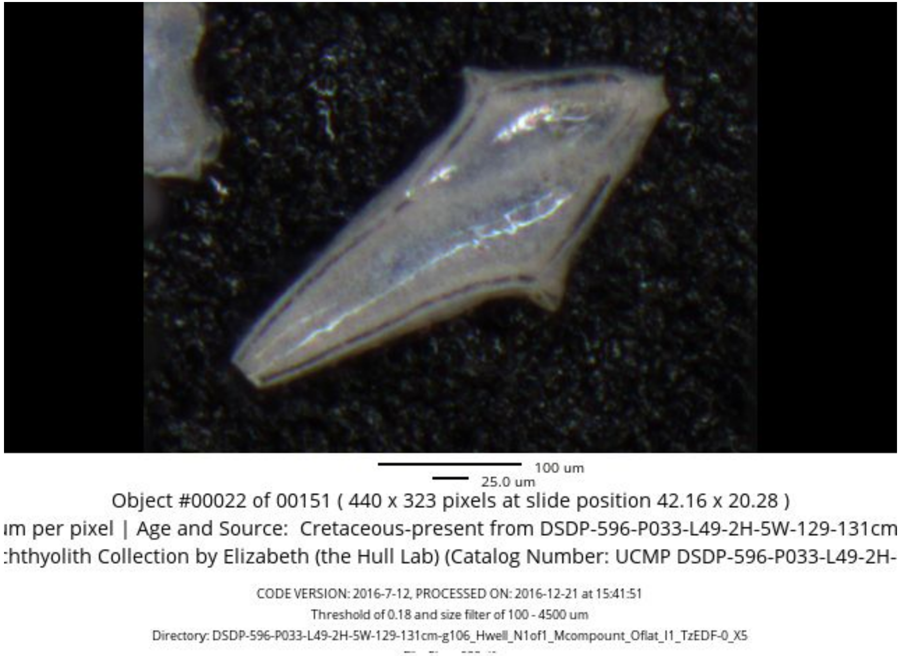

DSDP-596-P033-L49-2H-5W-129-131cm-g106_Hwell_N1of1_obj00022

Cruciform Arrow

The cruciform arrow type has a cruciform shape with no ridges and two vertices on either side at ~ ¼ the length of the denticle, It does not have clear directionality.

Fossil Only

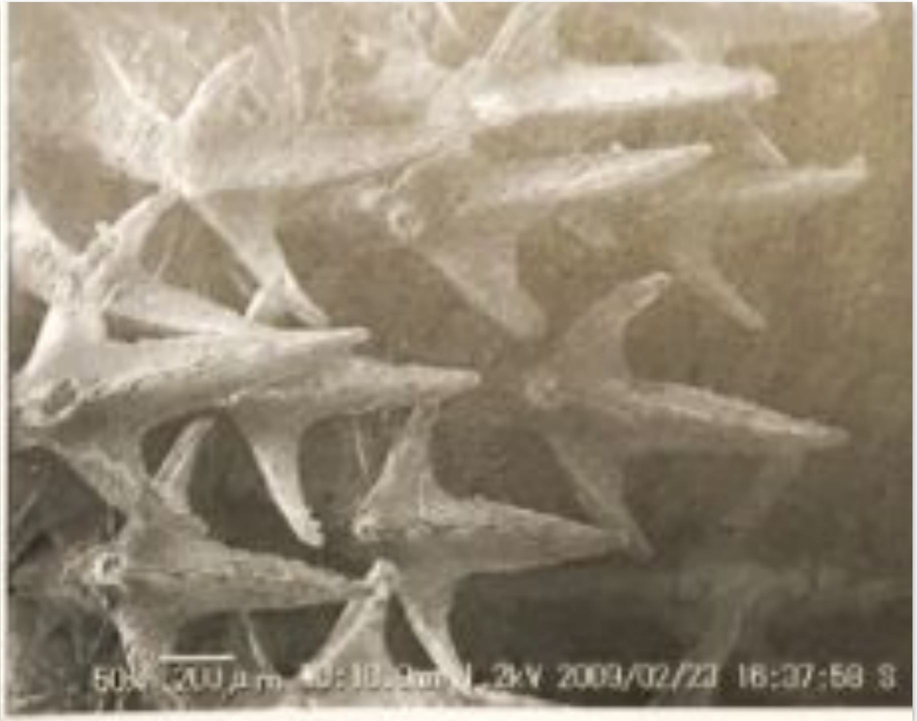

Deania calcea, Castro, Pg. 86, Fig. 19e

Dagger

The dagger type has a pointed spade type and its three triangular ridges each act as cusps which make up the majority of the crown area and diverge from the anterior. The central ridge and cusp is longer than the two side ridges and cusps which are slightly curved.

Taxonomic Citation

Deania profundorum, Castro, Pg. 89
Deania calcea, Castro, Pg. 86
Oxynotus caribbaeus, Castro, Pg.138
Oxynotus centrina, Reif, Pg.120, Fig. B1

Modern Only

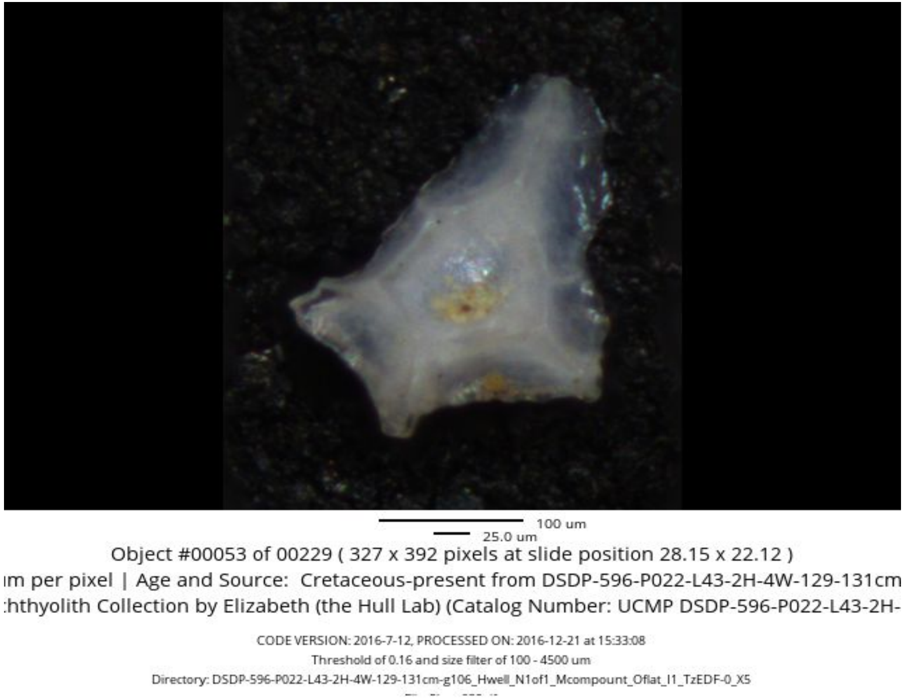

DSDP-596-P022-L43-2H-4W-129-131cm-g106_Hwell_N1of20_obj00053

Diamond Kite

The diamond kite has a cruciform shape and is defined by its central ridge system shape with five or six edges outlining a circular dimple. A long ridge extends from a vertex of the central shape.

Taxonomic Citation

Etmopterus pusillus, Feichtinger et al.,Fig. C2

Eared Trident

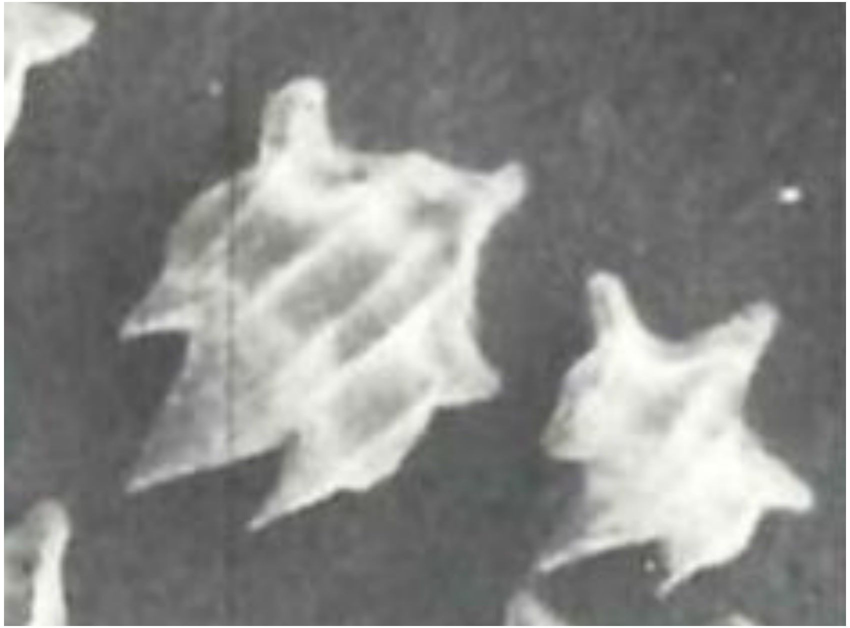

Hexanchus griseus, Reif, Pg. 105, Fig. M3

The eared trident has a pointed spade shape with three cusps, the central cusp is distinct and is larger than the side cusps. There are also three vertices/points at the anterior side of the crown with the central cusp opposite the bottom central anterior point and two anterior side projections which diverge away from the central point. There are five thin ridges which may define some of the cusps. The ridges extend from the anterior points of the crown to the beginning of the posterior cusps, some ridges may extend to the end of the cusps.

Taxonomic Citation

Hexanchus griseus, Reif, Pg. 105, Fig. M3

Modern Only

Elephant

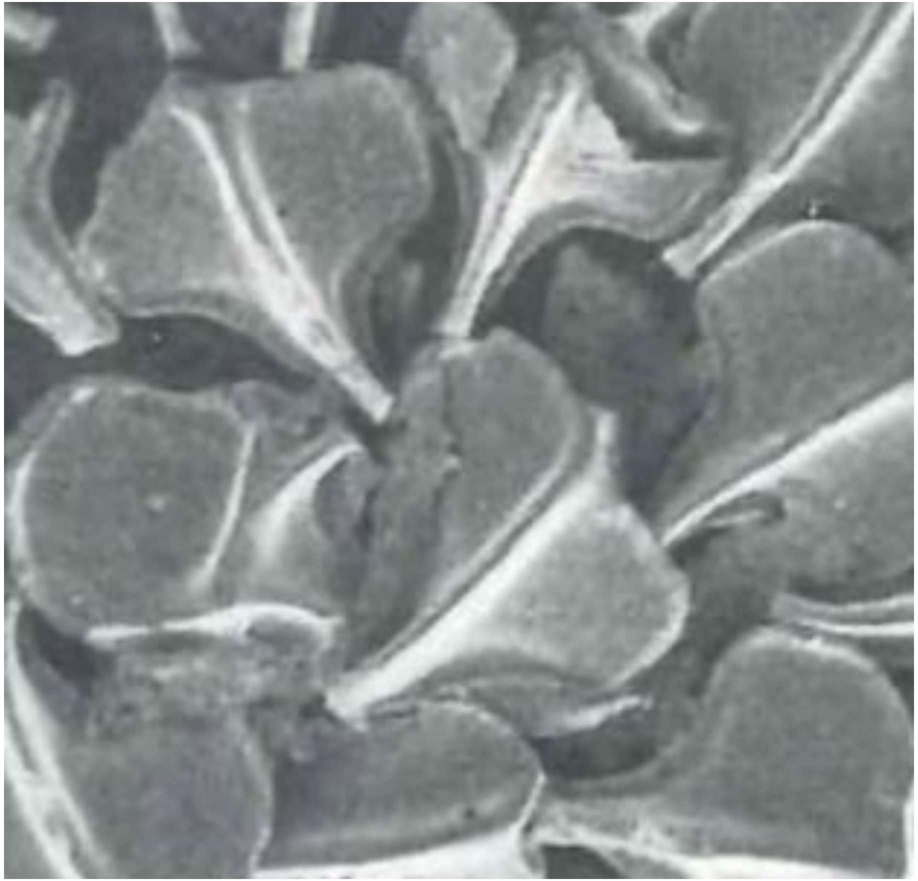

Squalus acanthias, Reif, Pg. 114, Fig. H1

The elephant type has a fan shape with a round posterior and a distinct central ridge which extends to the anterior vertex.

Taxonomic Citation

Squalus acanthias, Reif, Pg. 114, Fig. H1 and B2 and MCZ 32341

Modern Only

Elongated Asymmetrical

The elongated asymmetrical type has an elongated asymmetrical shape with a round posterior and anterior and scalloped texture from the ridges which cover the crown. The ridges are longest near the center of the crown and get shorter towards the sides. The ridges also get shallower towards the posterior of the crown.

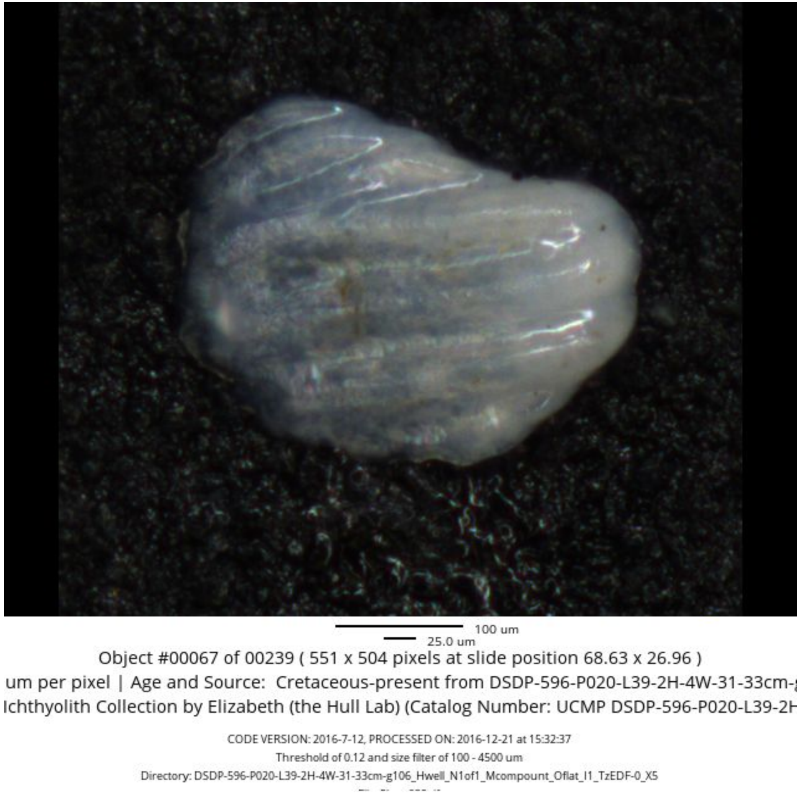

DSDP-596-P020-L39-2H-4W-31-33cm-g106_Hwell_N1of1
_obj00067

Fossil Only

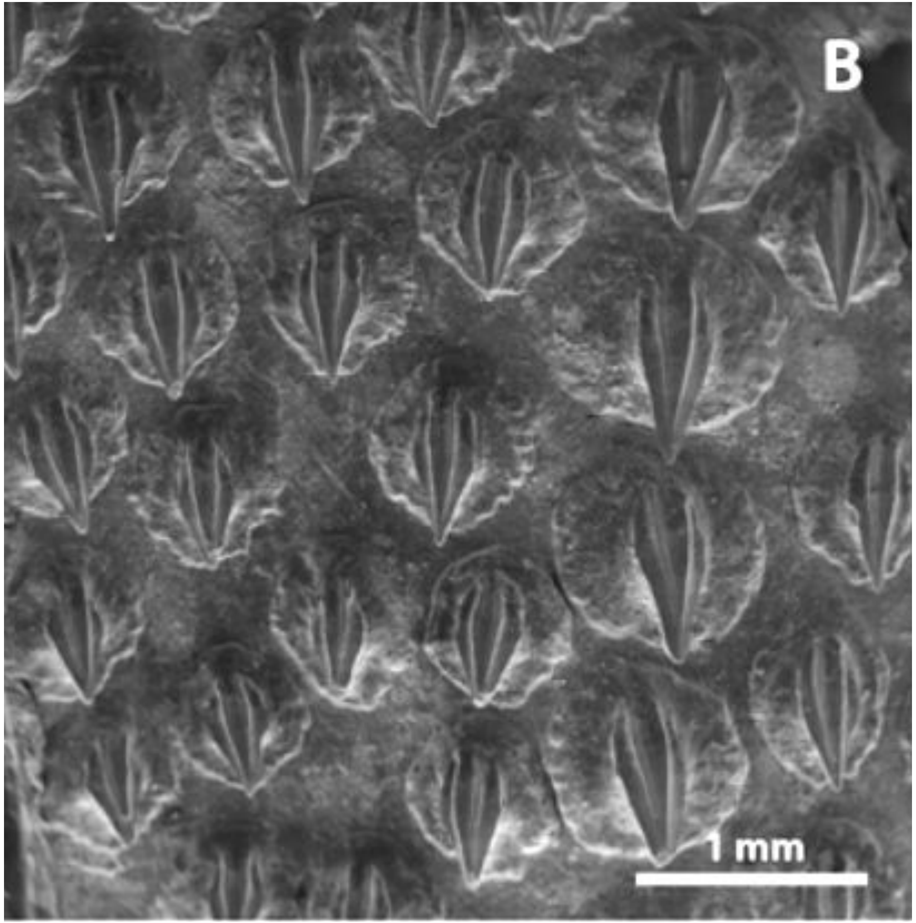

Squatina argentina, Vaz & Carvalho, Fig. B

Extended Central Long Troughed Petal

The extended central long troughed petal has a triangular shape with two ridge segments which converge near the posterior vertex of the crown creating a central trough which begins at the anterior edge of the crown and terminates just before the posterior vertex. The crown is propped up on a mound.

Taxonomic Citation

*Squatina guggenheim,*Vaz & Carvalho, Fig. F
Squatina argentina, Vaz & Carvalho, Fig. B and D

Modern Only

Extended Central Ridged and Textured Trident

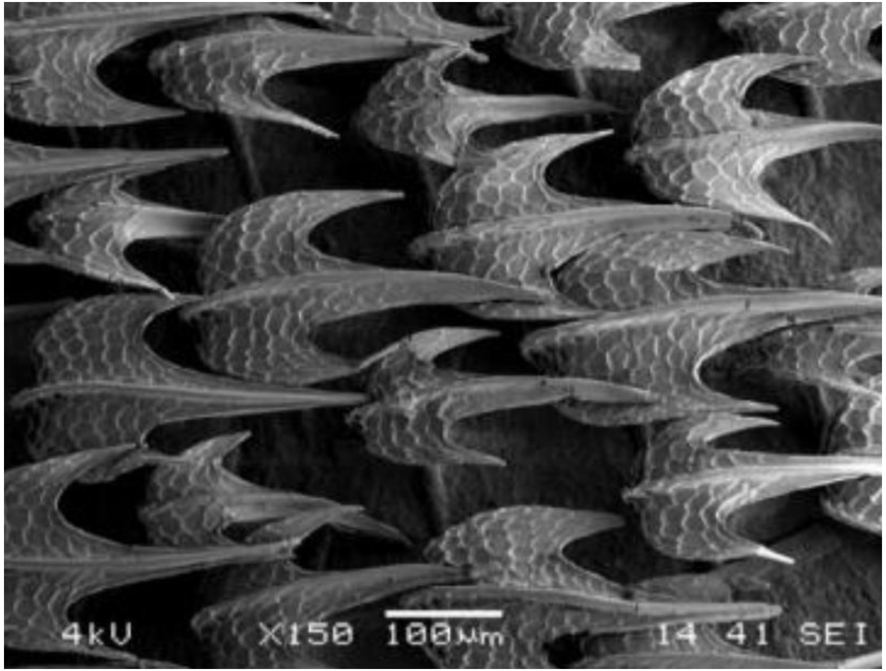

Galeus arae, Castro, Pg. 323

The extended central ridged and textured trident has a pointed spade shape with three cusps with only the central cusp defined by a dingle central ridge. This central cusp is distinct from the side cusps because it is longer. This type is also defined by the honeycomb-like surface texture which covers the entire crown.

Taxonomic Citation

*Apristurus parvipinnis,*Castro, Pg. 309
Galeus arae, Castro, Pg. 323
Parmaturus campechiensis, Castro, Pg. 330
Zameus squamulosus, Vaz, Fig. g

Modern Only

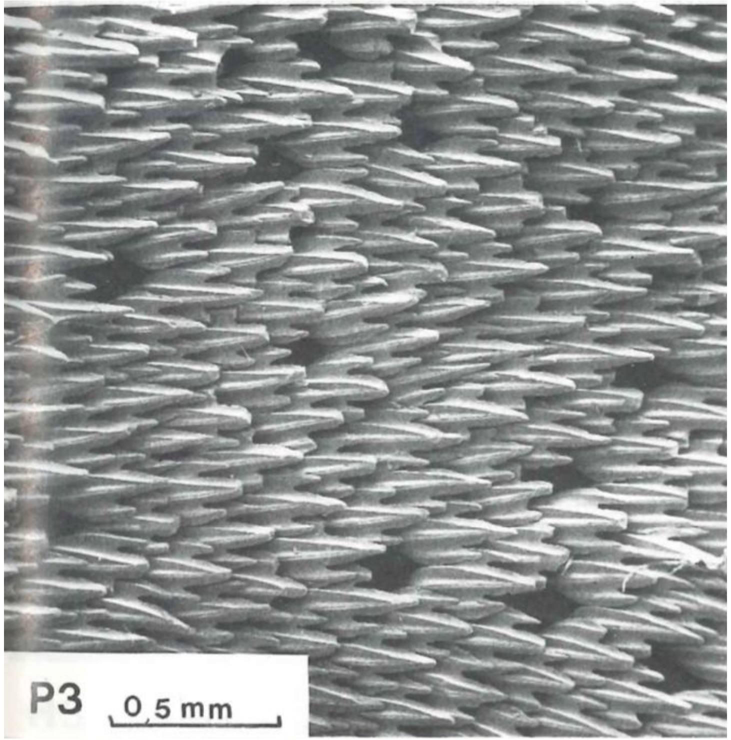

Carcharhinus plumbeus Reif Pg. 197, Fig. P3

Extended Troughed Trident

The extended troughed trident has a pointed spade shape with three cusps at the posterior and four ridges. The central cusp extends longer than the other two and is composed of two ridges which converge at the posterior to create a central trough along the length of the crown.

Taxonomic Citations
Apristurus manis, Castro, Pg. 205
Carcharhinus plumbeus, Reif, Pg. 194, Fig, M2
Carcharhinus plumbeus, Reif, Pg. 197, Fig. P3
Carcharhinus plumbeus, Reif, Pg. 197, Fig. M3
Negaprion brevirostris, Reif, Pg. 225, Fig. P3
Galeocerdo cuvier, Reif, Pg. 227, Fig. M1
Scymnodalatias albicauda, Vaz (2021), Fig. h

Modern Only

Extended Central Troughed Petal

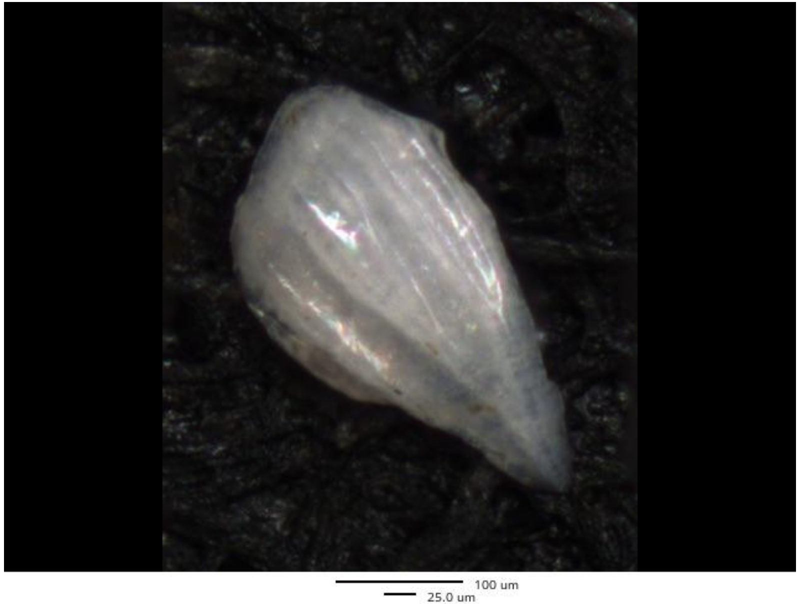

Object #00004 of 00082 (384 x 460 pixels at slide position 61.36 x 06.83)

m per pixel | Age and Source: Cretaceous-present from DSDP-596-P045-M46-2H-6W-110-112cm

hthyolith Collection by Elizabeth (the Hull Lab) (Catalog Number: UCMP DSDP-596-P045-M46-2H-

CODE VERSION: 2016-7-12, PROCESSED ON: 2016-12-21 at 15:54:59

Threshold of 0.17 and size filter of 100 - 4500 um

Directory: DSDP-596-P045-M46-2H-6W-110-112cm-g106_Hwell_N1of1_Mcompount_Oflat_I1_TzEDF-0_X5

DSDP-596-P045-M46-2H-6W-110-112cm-g106_obj00004

The extended central troughed petal has a stretched spade shape with a round anterior and a posterior vertex. The crown has four to five ridges, the inner two ridges create a central trough and converge ~ ⅔ up the length of the crown and the two side ridges are slightly shorter. The side ridges may also create "troughs" by converging towards the posterior.

Fossil Only

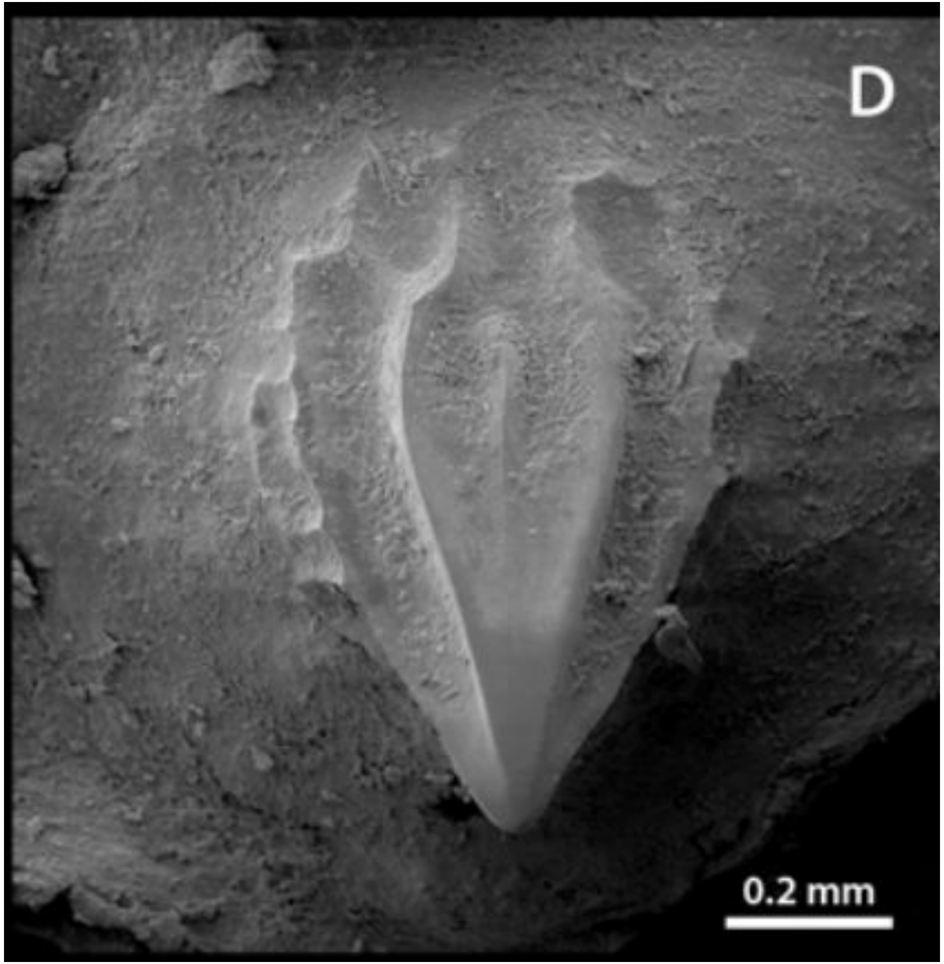

Squatina guggenheim, Vaz, Fig. D

Extended Forked Arrow

The extended forked arrow has an arrow-like shape with a posterior vertex and an anterior which does not have a clear terminating point and instead is composed of two anterior ridges which become gradually shallower and fade into the surrounding skin. These two anterior ridges diverge and then connect to two longer ridges which converge at the posterior vertex creating a triangular shape. Between these two more posterior ridges is a straight central ridge.

Taxonomic Citation

Squatina guggenheim, Vaz, Fig. D

Modern Only

Eyeball

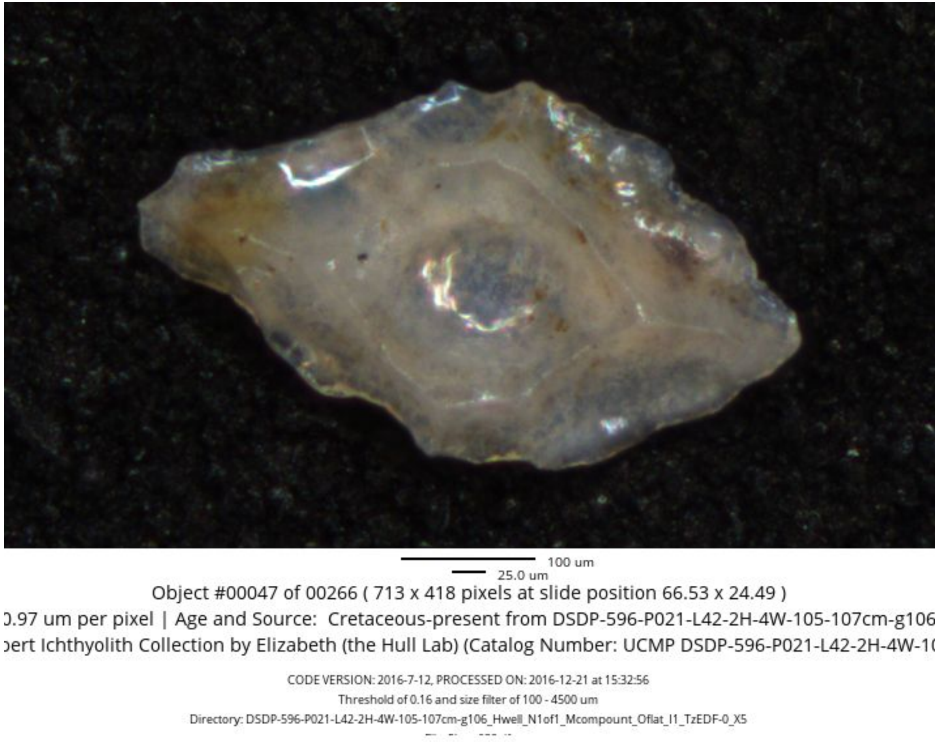

DSDP-596-P021-L42-2H-4W-105-107cm-g106_Hwell_N1of1_obj00047

The eyeball type has a diamond-like shape with a central ridge system shape which outlines a circular dimple. Each vertice of the central ridge system shape has a corresponding ridge which radiates from the vertex to the outer edges of the crown creating a scalloped texture.

Fossil Only

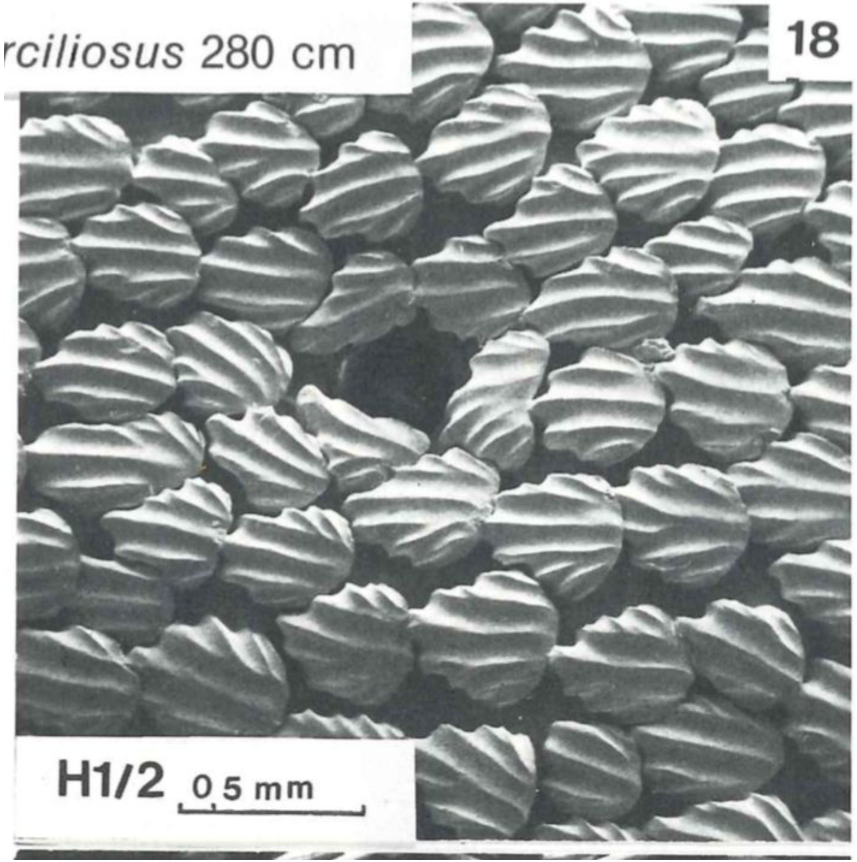

Alopias superciliosus, Reif Pg. 141, H1/2

Five Thin Ridged Branching Fan

The five thin ridged branching fan type has a pointed spade shape with five ridges which diverge from the anterior of the crown. The ridges have differing length with some ridges starting at the anterior and terminating at the posterior and others which branch out and terminate along the edges of the crown.

Taxonomic Citation

Alopias superciliosus, Reif Pg. 141, H1/2

Prionace glauca, Reif, Pg. 215, Fig. C2 (Central) and Pg. 221, Fig. N

Modern Only

Five Thin Ridged Fan

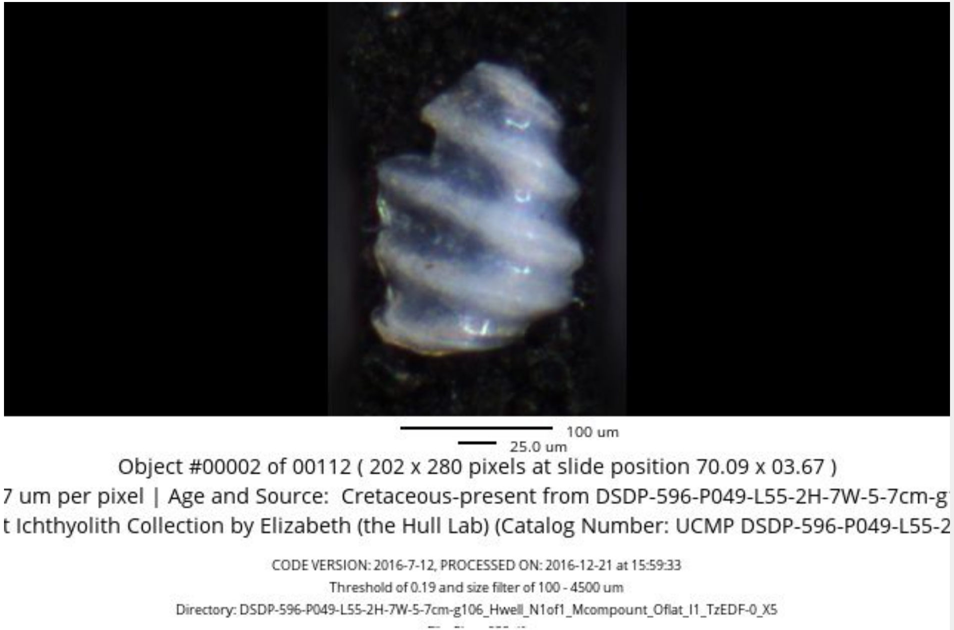

DSDP-596-P049-L55-2H-7W-5-7cm-g106_Hwell_N1of1_obj00002

The five thin ridged fan has a fan shape which is approximately 2 times wider than it is tall with five ridges which cover the crown defining its edges and which are equal in width but get shorter towards the edges. The ridges have equal space between them approximately the width of one ridge.

Taxonomic Citation

Alopias superciliosus, Reif, Pg. 141, Fig. H2

A. vulpinus, Castro, Pg. 241

A. vulpinus, Reif, Pg. 139, Fig. C1

Carcharhinus acronotus, Castro, Pg. 393

C. albimarginatus, Castro, Pg. 397

C. amblyrhynchos, Reif, Pg. 199, Fig. B1, B3, B1, Pg. 200, Fig. P3 and H2, and Pg. 202 B1, B2, and C3

C. brevipinna, Castro, Pg. 407

C. falciformis, Castro, Pg. 413

C. falciformis, Reif, Pg. 177, Fig. H3

C. galapagensis, Castro, Pg. 417

C. limbatus, Castro, Pg. 432

C. longimanus, Castro, Pg. 438

C. melanopterus, Reif, Pg. 204, Fig. B1 and Pg. 208 C2, C3, and M1

C. obscurus, Castro, Pg. 444

C. obscurus, Reif, Pg. 187, Fig. P1 and P2

C. perezii, Castro, Pg. 449

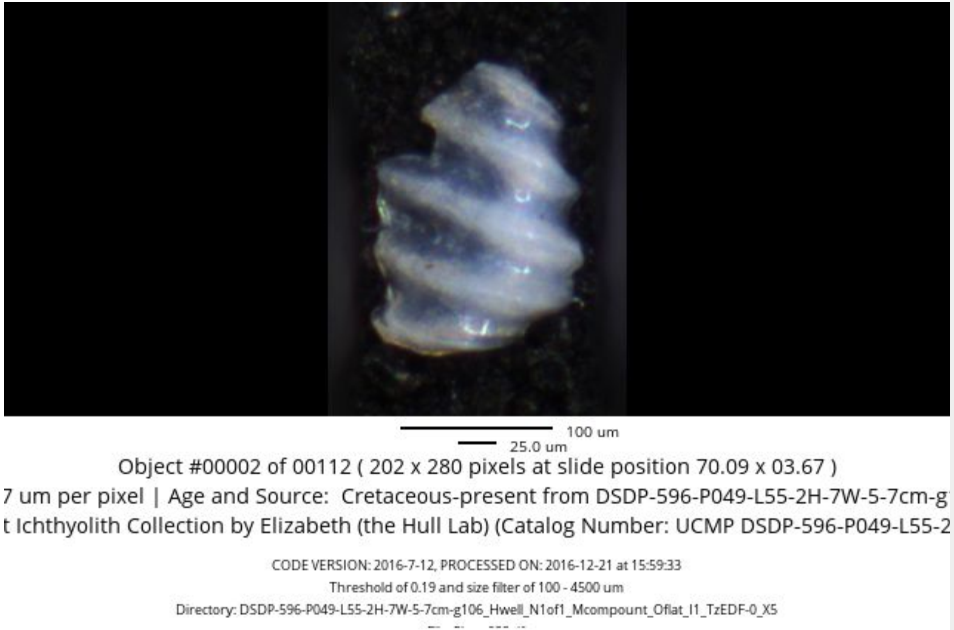

DSDP-596-P049-L55-2H-7W-5-7cm-g106_Hwell_N1of1_obj00002

Five Thin Ridged Fan

The five thin ridged fan has a fan shape which is approximately 2 times wider than it is tall with five ridges which cover the crown defining its edges and which are equal in width but get shorter towards the edges. The ridges have equal space between them approximately the width of one ridge.

Taxonomic Citation

Isurus oxyrinchus, Reif, Pg. 147, Fig. P1

Mustelus canis, Ankhelyi et al., Pg. 5, Fig. b

Nasolamia velox, CastroPg. 478

Negaprion brevirostris, Reif, Pg. 224, Fig. B4 and Pg. 225, Fig. M1

Sphyrna lewini, Castro, Pg. 509

S. tiburo, Castro, Pg. 521

S. tudes, Reif, Pg. 237, Fig. H1 and Pg. 238, Fig. M2

S. zygaena, Castro, Pg. 529

Triaenodon obesus, Reif, Pg. 210, Fig. B1

Flying Squirrel

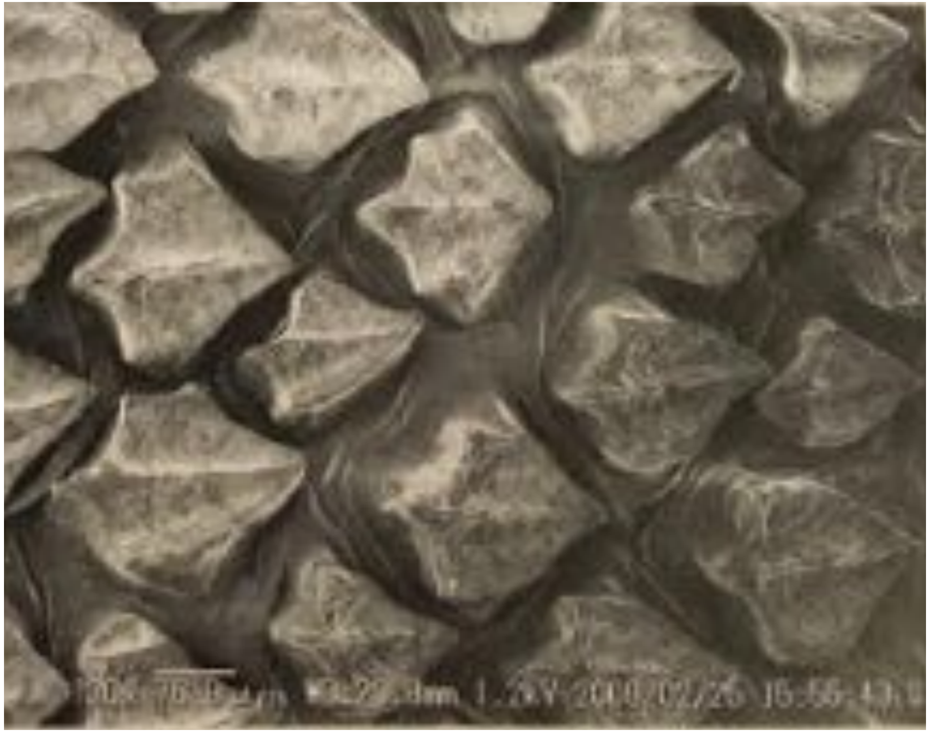

Pseudocarcharias kamoharai, Castro, Pg. 222

The flying squirrel type has a pointed spade shape which is wider than it is long. It has a singular ridge which bisects the crown and extends its length. The side edges of the denticles (dorsal to ventral directions) have a trapezoidal shape.

Taxonomic Citations

Pseudocarcharias kamoharai, Castro, Pg. 222
Chlamydoselachus anguineus, Castro, Pg. 22, Fig. 1d

Modern Only

Forked Oval

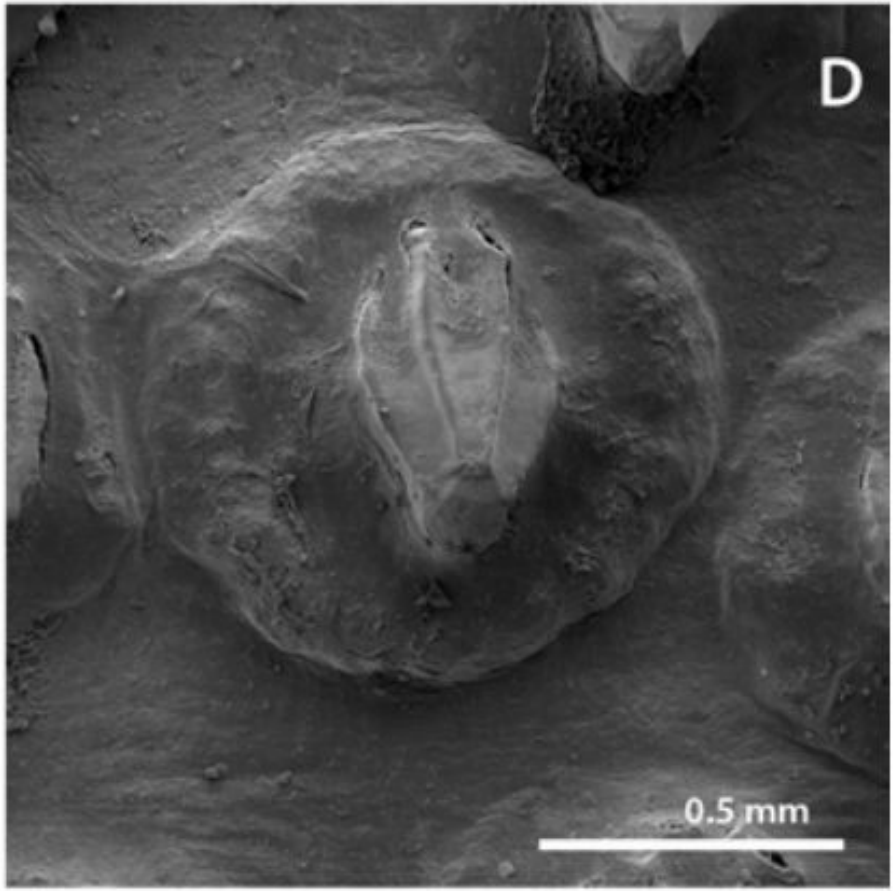

Squatina occulta, Vaz, D. F., & De Carvalho, M. R. (2013), Fig. D

The forked oval has a stretched spade shape with four ridges, two of which define the sides of the crown and two interior ridges which are longer and diverge then converge from the anterior to a rounded posterior.

Taxonomic Citation

Squatina occulta, Vaz, D. F., & De Carvalho, M. R. (2013), Fig. D

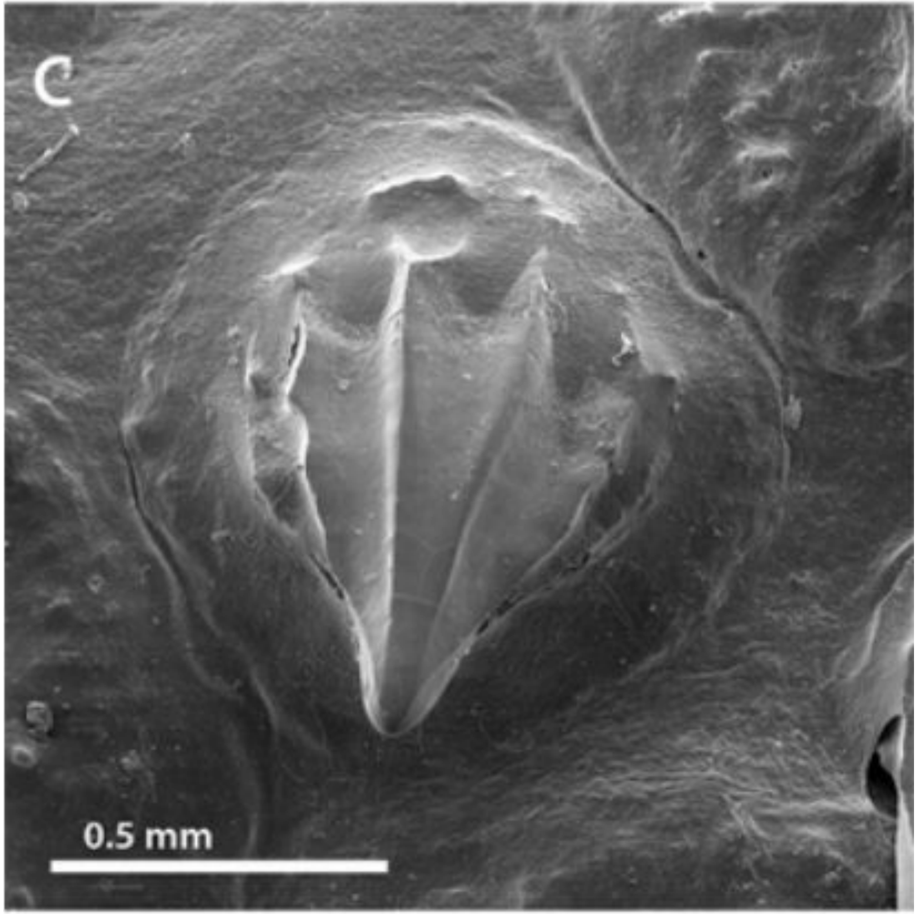

Squatina occulta, Vaz, D. F., & De Carvalho, M. R. (2013), Fig. C

Forked Spade

The forked spade has a triangular shape with an anterior composed of multiple pointed segments composed of ridges which converge at a posterior vertex. The crown is propped up on a mound.

Taxonomic Citation

Squatina occulta, Vaz, D. F., & De Carvalho, M. R. (2013), Fig. C

Modern Only

Four Ridged Linear Spade

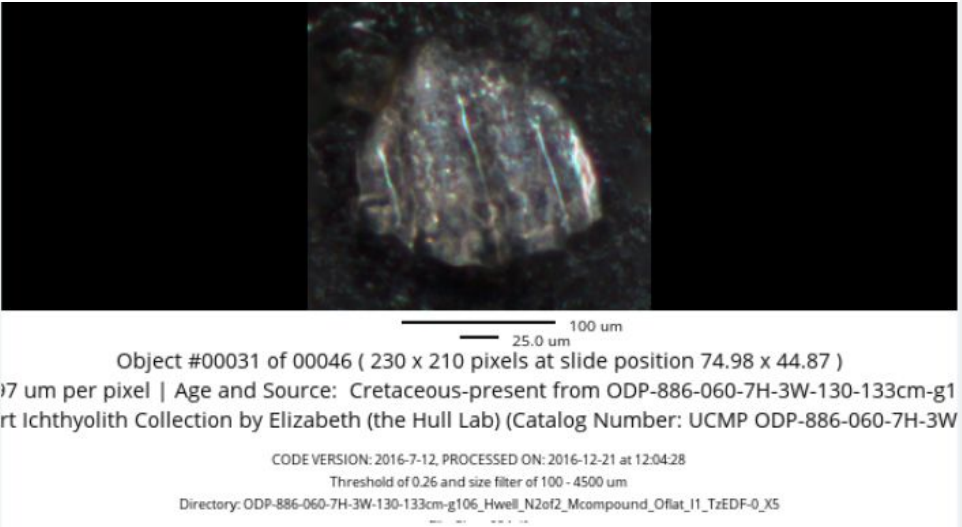

ODP-886-060-7H-3W-130-133cm-g106_obj00031_edf

The four ridged linear spade has a squared spade shape with an anterior vertex and a straight scalloped posterior edge. This type has four wide linear ridges with the two side ridges being slightly shorter than the middle two.

Fossil Only

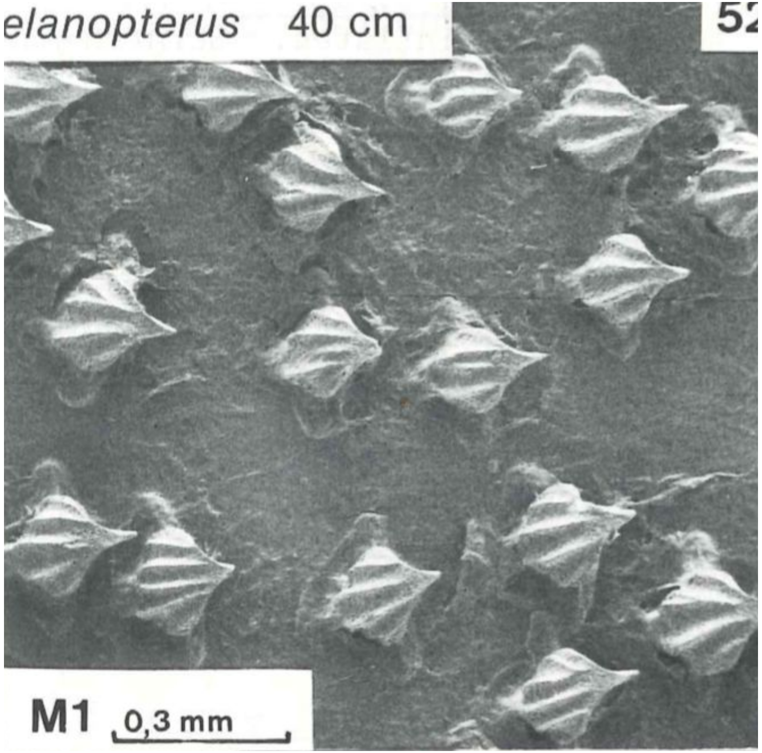

Carcharhinus melanopterus, Reif, Pg. 206, Fig. M1

Four Thin Ridged Petal

The four thin ridged petal has a rounded spade shape with a wide rounded anterior and a posterior vertex which accounts for ~¼ the length of the denticle. The type has four distinct ridges which are equal in length and do not extend the entire length of the crown.
. This type has four ridges with two shorter side ridges and two longer interior ridges.

Taxonomic Citation

Mustelus canis, Castro, Pg. 369
Mustelus canis, Ankhelyi et al., Pg. 5, Fig. d
Carcharhinus melanopterus, Reif, Pg. 206, Fig. M1
Sphyrna zygaena, Reif, Pg. 244, Fig. M1
Squatina varii, Vaz et al. (2018), Fig. A (surrounding)

Modern Only

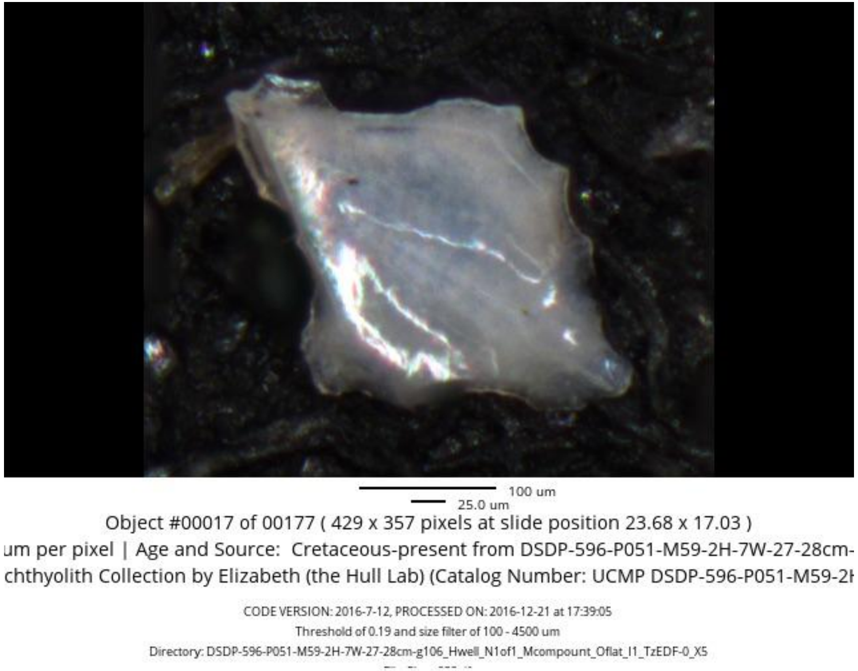

DSDP-596-P051-M59-2H-7W-27-28cm-g106_obj00017_edf.jpeg

Funky Central Ridged Diamond

The Funky Central Ridged Diamond had a diamond shape with one plane of symmetry and meandering side ridges which outline the edge of the denticle and a central triangular ridge.

Taxonomic Citation

Chlamydoselachus anguineus, Reif, Pg. 103, Fig. B5

Chlamydoselachus anguineus, Reif, Pg. 103, Fig. C2

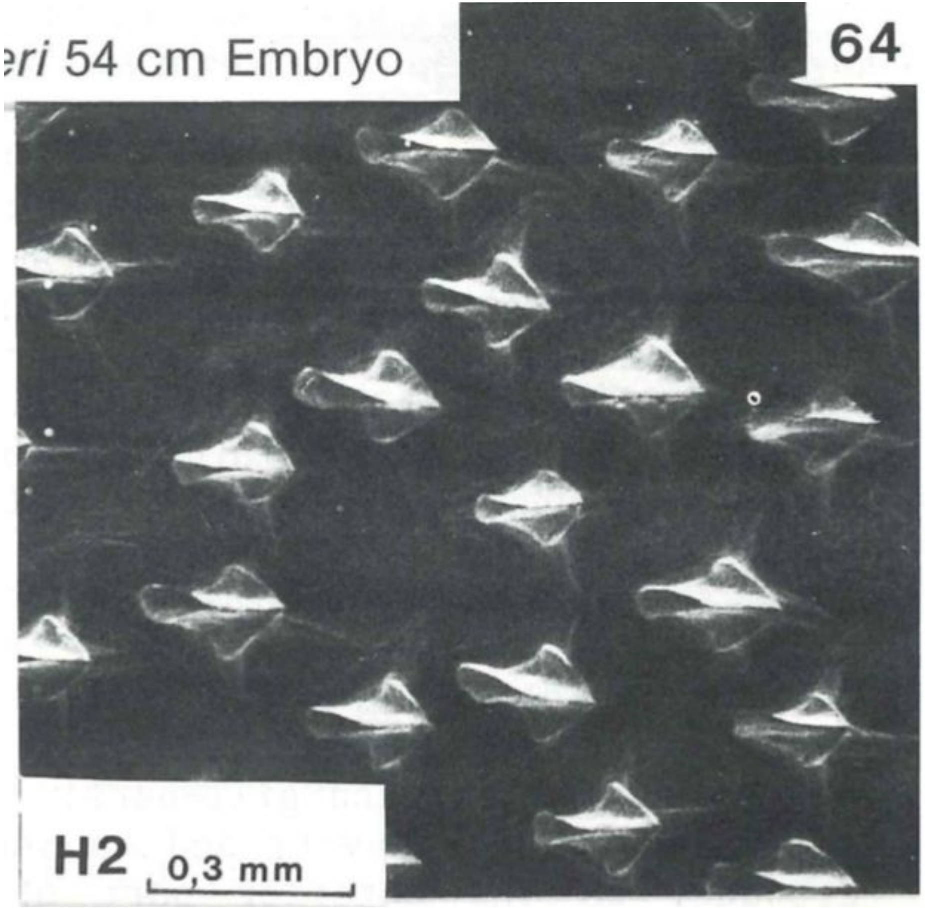

Galeocerdo cuvier, Reif, Pg. 227, Fig. H2

Funky Central Ridged Spade

The funky central ridges spade type is a catch all for pointed spades with a single ridge that has a unique shape compared to other denticles.

Taxonomic Citation

Galeocerdo cuvier, Castro, Pg. 466
Squalus acanthias, Reif, Pg. 116, fig. B2
Galeocerdo cuvier, Reif, Pg. 227, Fig. H2

Modern Only

Triangular Troughed Arrow

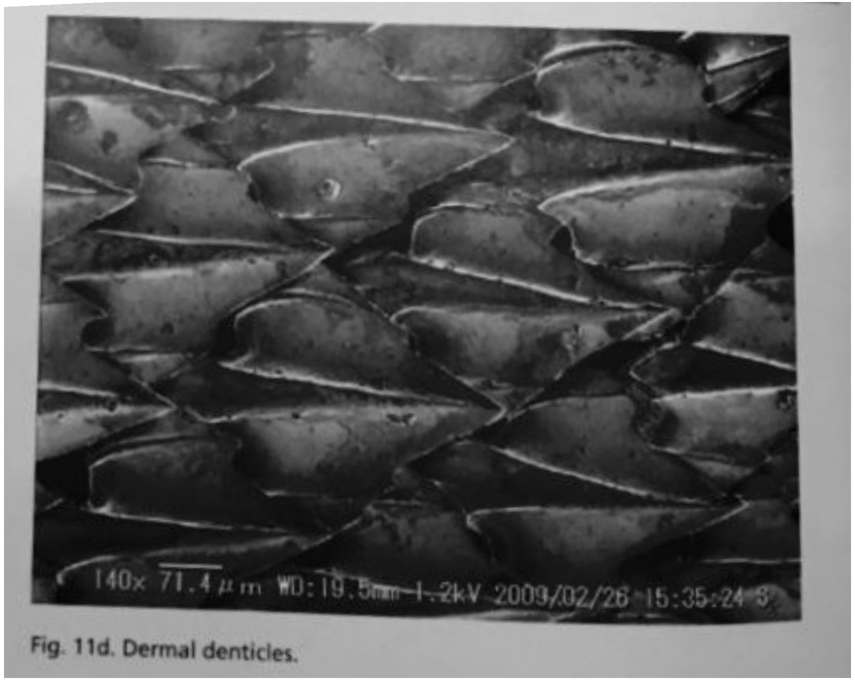

Squalus mitsukuri, Castro, Pg. 65

The triangular troughed arrow has an arrow like shape with a triangular trough which terminates at a posterior vertex. On either side of the central trough are shorter side ridges and a flat unridged curved portion of crown..

Taxonomic Citation

Cirrhigaleus asper, Castro, Pg. 52

Modern Only

Fusiform Dimpled Diamond

The fusiform dimpled diamond has a diamond-like shape. The majority of the crown’s area is composed of a long oval-like central ridge shape with an elongated dimple at its center. The ridges which compose the central ridge system shape are thin and meandering and at either vertix of the diamond crown a ridge connects to the central shape.

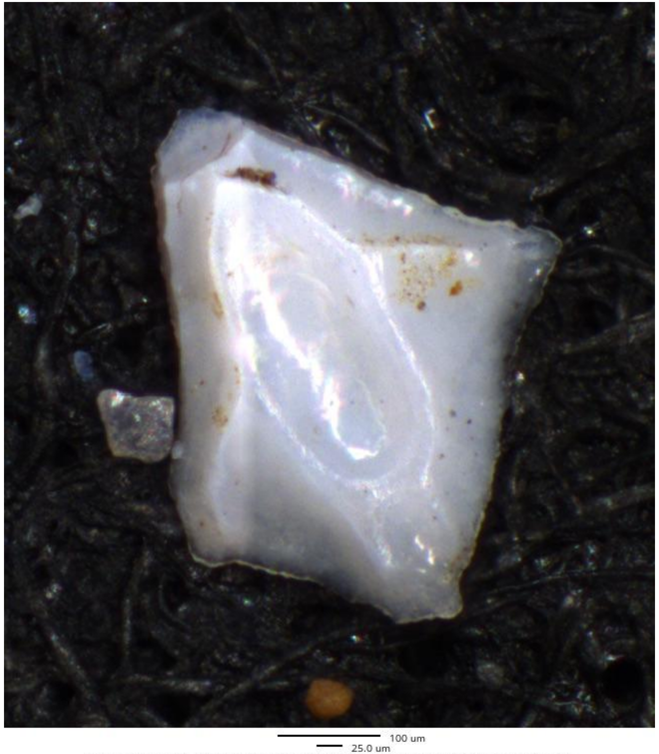

Object #00002 of 00059 (652 x 727 pixels at slide position 47.37 x 06.23)
um per pixel | Age and Source: Cretaceous-present from DSDP-596-P041-M38-2H-6W-71-73cm-g
Ichthyolith Collection by Elizabeth (the Hull Lab) (Catalog Number: UCMP DSDP-596-P041-M38-2H

CODE VERSION: 2016-7-12, PROCESSED ON: 2016-12-21 at 15:50:50
Threshold of 0.19 and size filter of 100 - 4500 um
Directory: DSDP-596-P041-M38-2H-6W-71-73cm-g106_Hwell_N1of1_Mcompount_Oflat_I1_TzEDF-0_X5

DSDP-596-P041-M38-2H-6W-71-73cm-g106_obj00002

Fossil Only

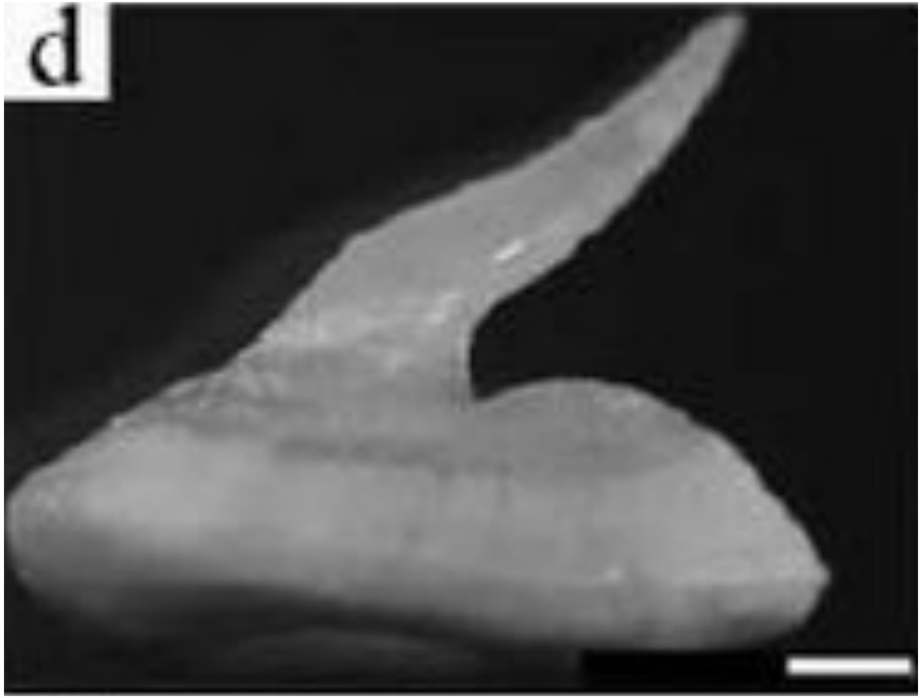

Raja clavata, Serra-Pereira et al., Fig. d

Fusiform Spine

The fusiform spine has a spine shape with a long semi-vertical fusiform crown. The crown does not have ridges and is composed of a smooth round oval anterior and a posterior vertex.

Taxonomic Citation

Amblyraja radiata, Gravendeel, Neer, and Brinkhuizen 26 (lateral)
Raja clavata, Serra-Pereira et al. Fig. d
Raja brachyura, Gravendeel, Neer, and Brinkhuizen, Fig. 6 (median)
Raja brachyura, Gravendeel, Neer, and Brinkhuizen, Fig. 6 (lateral)
Raja brachyura, Gravendeel, Neer, and Brinkhuizen, Fig. 6 (pectoral fin)
Raja brachyura, Gravendeel, Neer, and Brinkhuizen, Fig. 7 (claw type)
Raja clavata, Gravendeel, Neer, and Brinkhuizen, Fig. 9 (parallel)
Raja microocellata, Gravendeel, Neer, and Brinkhuizen, Fig. 11 (median)
Raja montagui, Gravendeel, Neer, and Brinkhuizen, Fig. 13 (median)
Raja montagui, Gravendeel, Neer, and Brinkhuizen, Fig. 13 (malar)
Raja montagui, Gravendeel, Neer, and Brinkhuizen, Fig. 14 (cross type prickle)
Raja undulata, Gravendeel, Neer, and Brinkhuizen, Fig. 17 (alar)
Leucoraja circularis, Gravendeel, Neer, and Brinkhuizen, Fig. 22 (median)
Etmopterus virens, Reif, Pg. 110, Fig. C2
Etmopterus robinsi, Lourtie, A., et al. 2022, Fig.

Modern Only

Generic Double Dimpled Complex Polygons

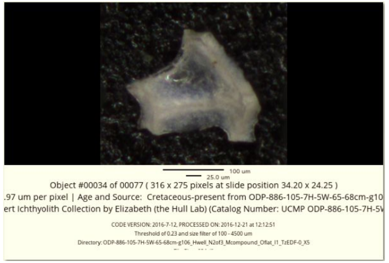

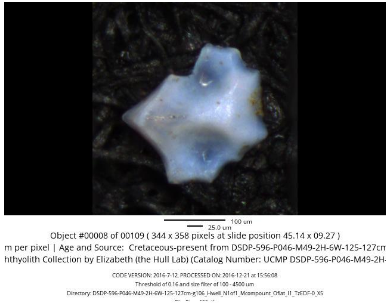

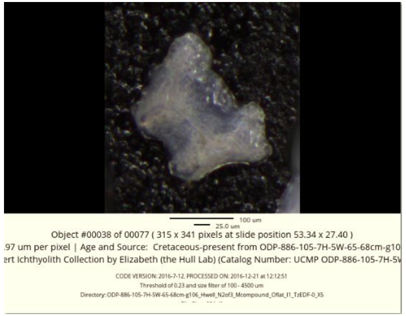

The generic double dimpled complex polygon is a catch all type for denticles with two dimples but which are two broken to code more specifically. These are denticles with a central branching pattern and a central bisecting ridge.

Fossil Only

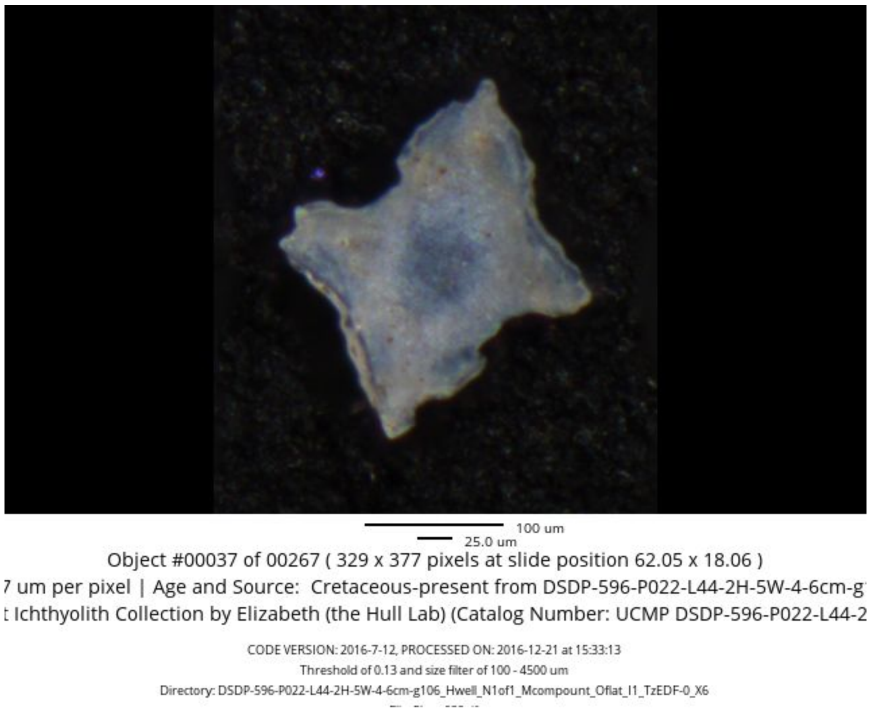

DSDP-596-P022-L44-2H-5W-4-6cm-g106_Hwell_N1of1_obj00037

Generic Kite

The generic kite type is a catch all for denticles that could clearly be identified as having a cruciform shape and a quadrilateral central ridge system shape outlining a central dimple but that were broken or had image quality issues making it difficult to determine a more specific type.

Fossil Only

Generic Single Dimpled Complex Polygon

The generic single dimpled complex polygon is a catch all for the fragmented denticles of the gross type single dimpled complex polygons. It displays a central branching pattern and is defined by a central ridge system shape composed of four or more thin meandering ridges and which outline a central dimple. These denticles typically have more than six ridges.

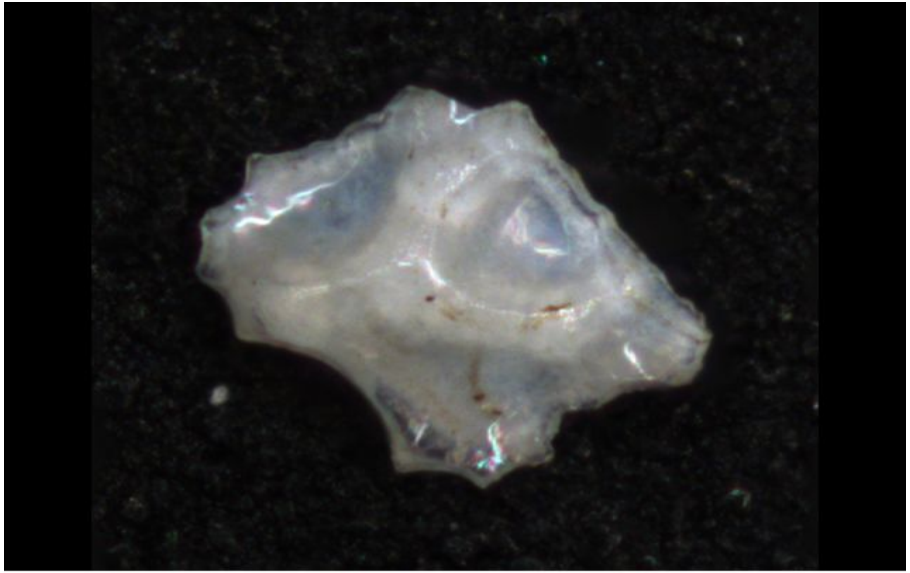

100 um
25.0 um
Object #00020 of 00095 (516 x 429 pixels at slide position 85.76 x 11.89)
um per pixel | Age and Source: Cretaceous-present from DSDP-596-P039-L51-2H-6W-57-59cm-
Ichthyolith Collection by Elizabeth (the Hull Lab) (Catalog Number: UCMP DSDP-596-P039-L51-2H

CODE VERSION: 2016-7-12, PROCESSED ON: 2016-12-21 at 15:50:00
Threshold of 0.12 and size filter of 100 - 4500 um
Directory: DSDP-596-P039-L51-2H-6W-57-59cm-g106_Hwell_N1of1_Mcompount_Oflat_I1_TzEDF-0_X5

DSDP-596-P039-L51-2H-6W-57-59cm-g106_Hwell_N1of1_obj00020

Fossil Only

GenGeo (Generic Geometric Denticle)

Categorized as a geometric denticle with a branching ridge orientation but too fragmented to type it more specifically

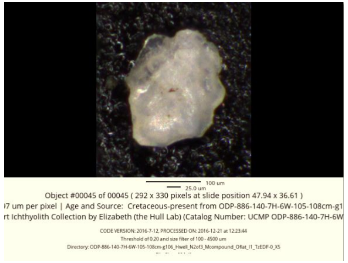

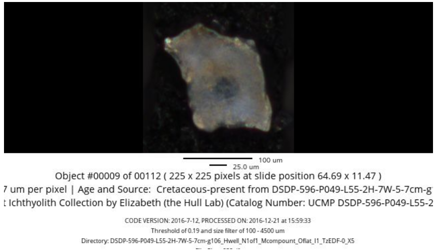

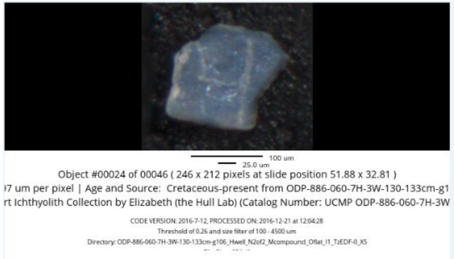

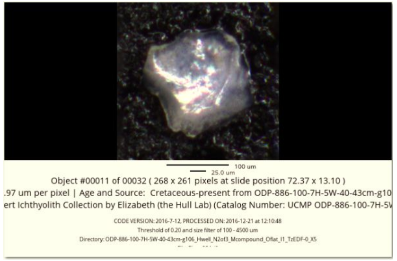

GenLin (Generic Linear Denticle)

Categorized as a linear denticle with unidirectional ridges but too fragmented to type it more specifically

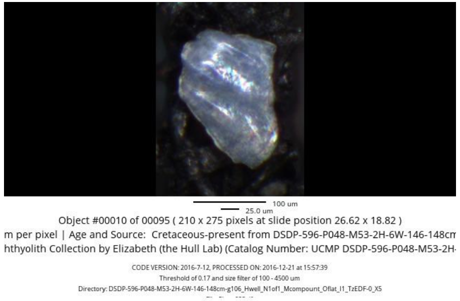

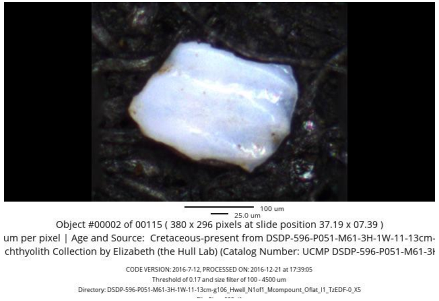

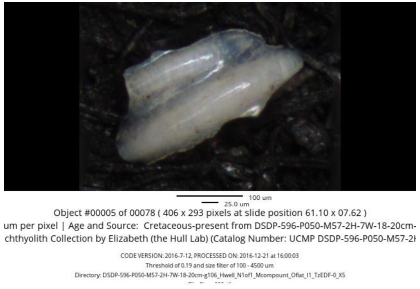

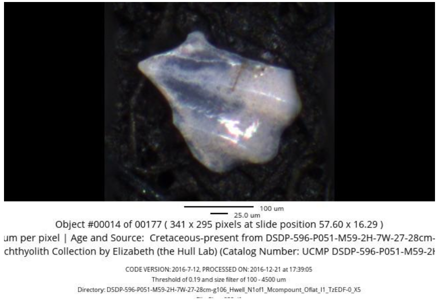

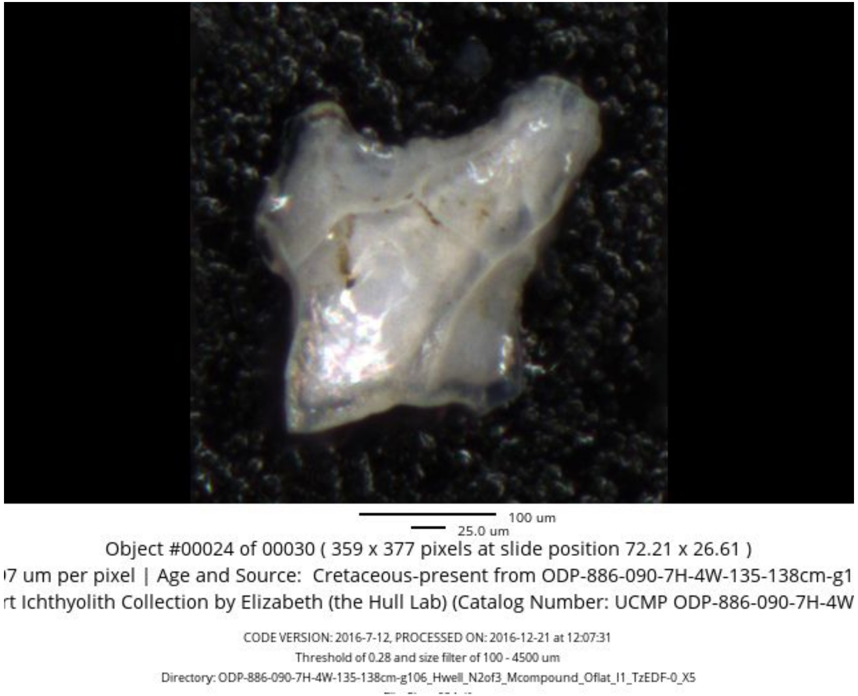

ODP-886-090-7H-4W-135-138cm-g106_obj00024_edf

Geometric Arrowhead

The geometric arrowhead type has an arrow-like shape with an anterior vertex and a round posterior cusp which is longer and wider than the side cusps. The crown has two meandering shallow ridges which run the length of the denticle from either side of the anterior vertex to the posterior.

Fossil Only

Helter Skelter

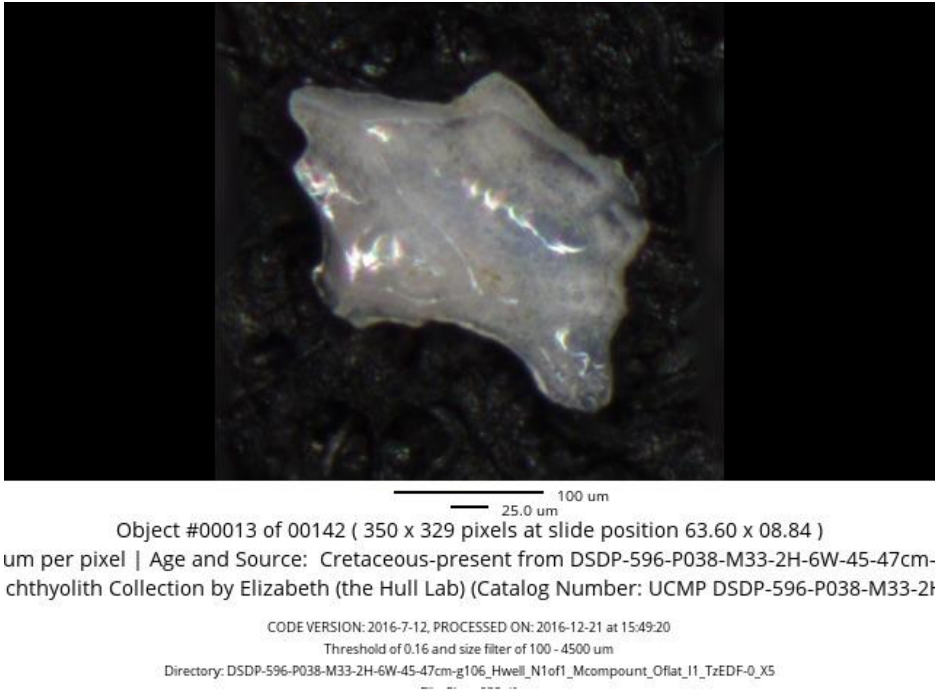

DSDP-596-P038-M33-2H-6W-45-47cm-g106_obj00013

The helter skelter type has a squared spade shape with a pentagonal central ridge system shape outlining a central dimple. On one or more of the sides of the central ridge system shape there is a distinctly serrated marginal texture.

Fossil Only

High Ridged Diamond

The high ridged diamond type has a diamond-like shape with 2 or more distinctly tall ridges which run parallel along the length of the crown.

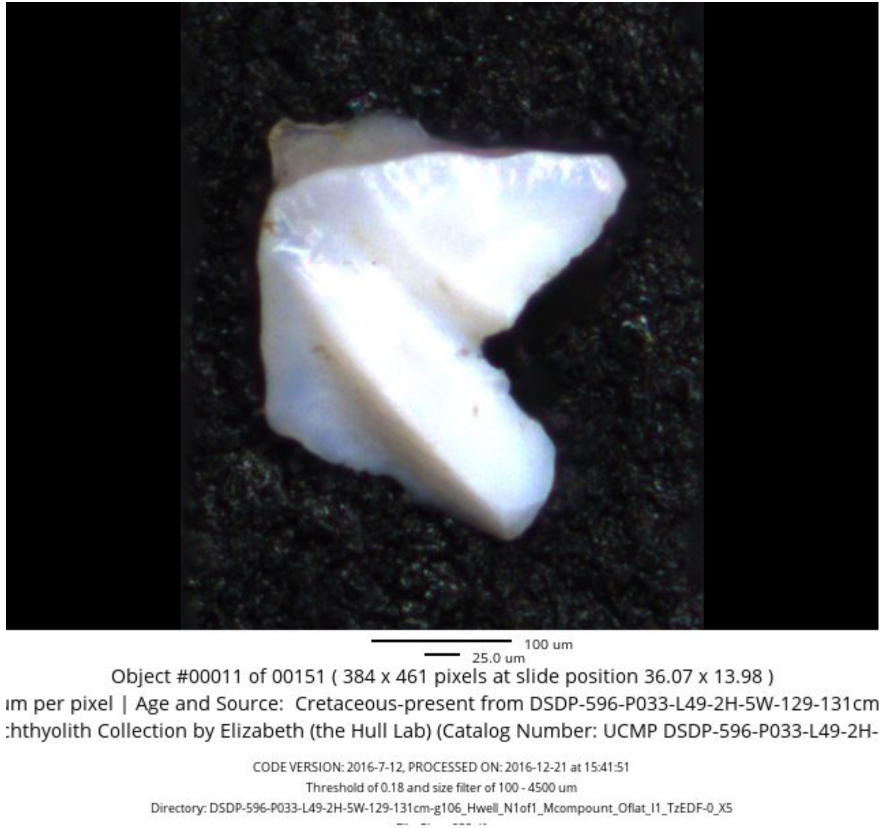

DSDP-596-P033-L49-2H-5W-129-131cm-g106_Hwell_N1of1_obj00011

Fossil Only

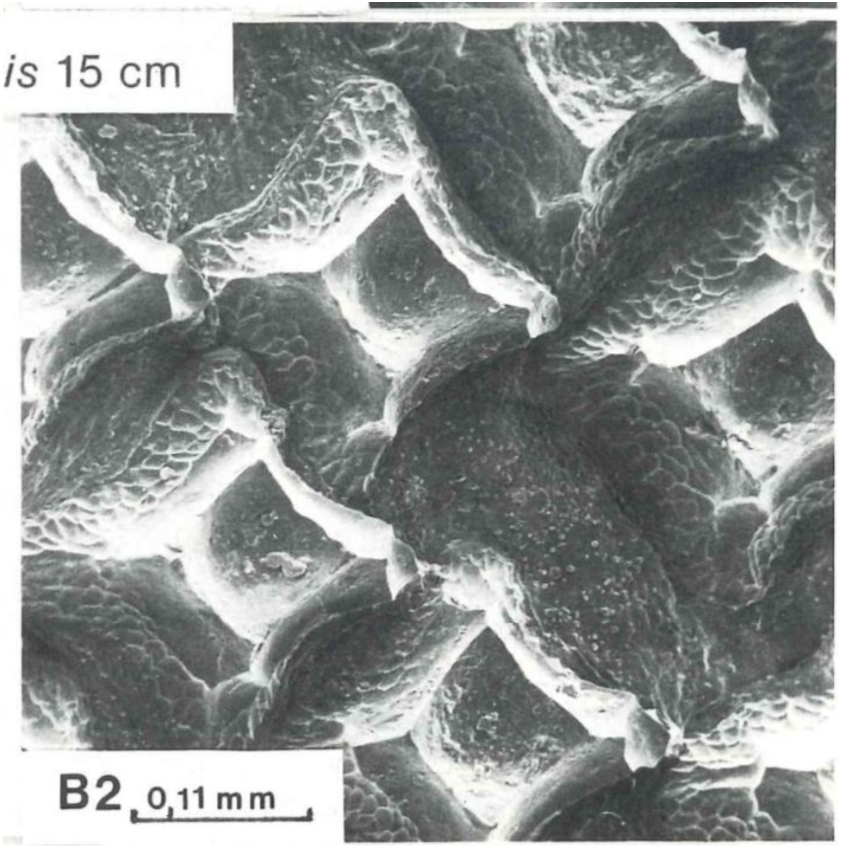

Isistius brasiliensis, Reif, Pg. 124, Fig. B2

Rounded Cone

The rounded cone type has a diamond like shape with two slightly curved anterior ridges which diverge from an anterior vertex and two straight posterior ridges which terminate in a posterior vertex. At each of the four vertices are shorter ridges which branch out to the edge of the crown. The central four ridges surround a central circular dimple.

Taxonomic Citation
Isistius brasiliensis, Reif, Pg. 124, Fig. B2
Squaliolus laticaudus, Castro, Pg. 156, Fig. 41e

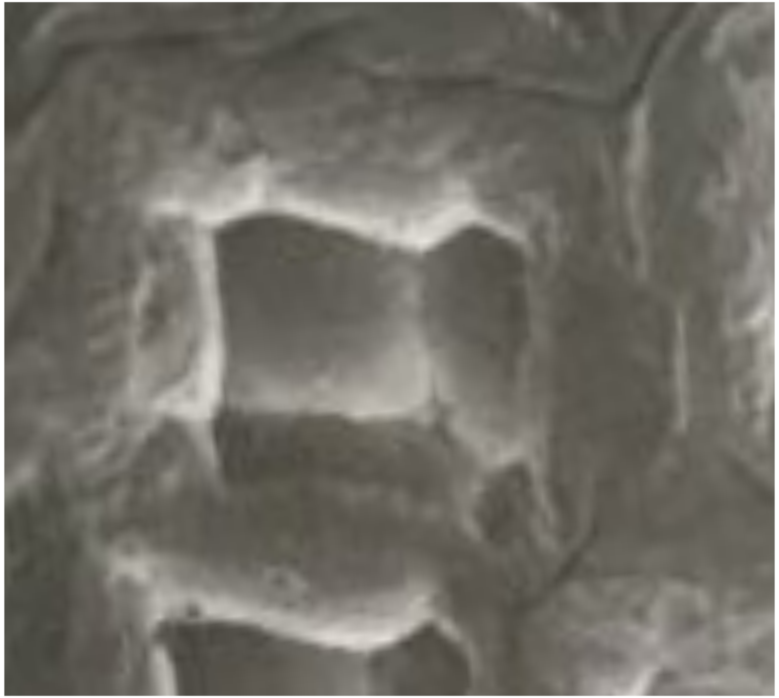

Isistius brasiliensis, Castro, Pg. 145

Kite with Bubble

The kite with bubble type has a cruciform shape with four ridges making up its central ridge system shape outlining a central dimple. This type is defined by a small ‘bubble” or ridge structure composed of 3 or more ridges at the end of one vertex which is separated by a straight ridge from the central ridge system shape.

Taxonomic Citation

Isistius brasiliensis, Castro, Pg. 145

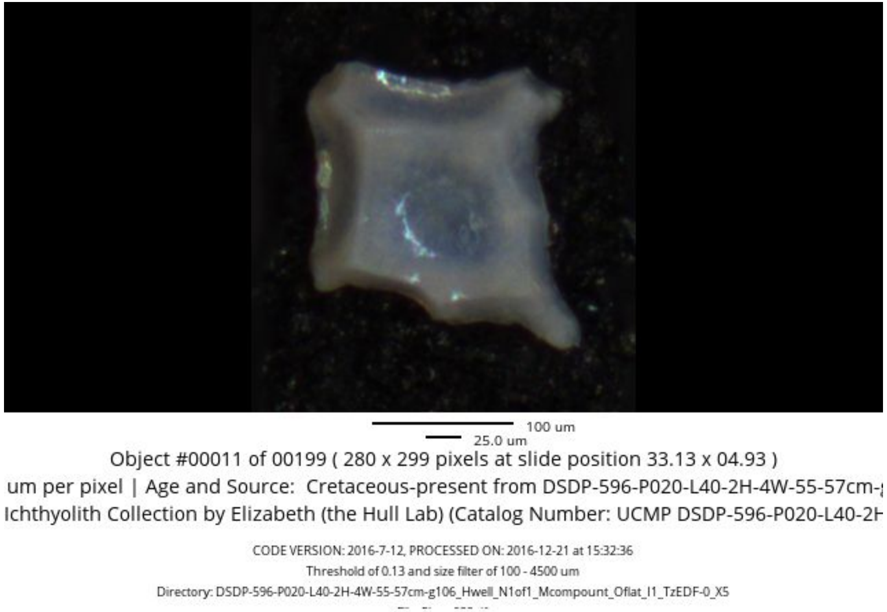

DSDP-596-P020-L40-2H-4W-55-57cm-g106_Hwell_N1of1_obj00011

Kite-Like

The kite-like type has a cruciform shape and is longer than it is wide with a central ridge system shape outlining a circular dimple. The difference between this type and other kite types is that they have more than four ridges which compose this central shape and can have more than 4 ridges which branch out from the central shape to the edges of the crown.

Taxonomic Citation

Etmopterus bigelowi, Castro, Pg. 98
E. schultzi, Reif, Pg. 108, Fig. B3

Llama

The llama type has a squared spade shape with straight side edges defined by semi-rectangular ridges that get slightly thinner from the rounded anterior to the straight posterior. The central ridge is wider and longer and the ridges get shorter towards the edge.

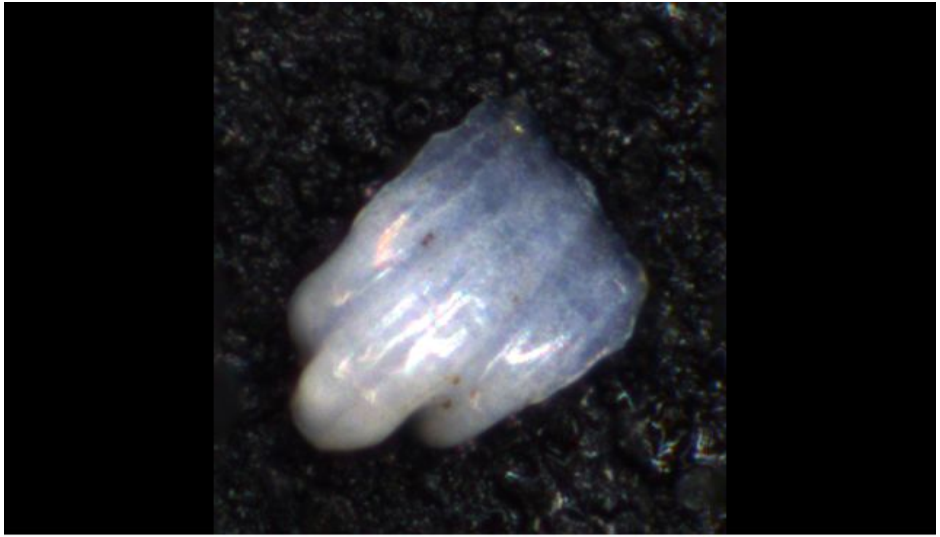

Object #00077 of 00239 (352 x 366 pixels at slide position 77.36 x 31.99)
um per pixel | Age and Source: Cretaceous-present from DSDP-596-P020-L39-2H-4W-31-33cm-
Ichthyolith Collection by Elizabeth (the Hull Lab) (Catalog Number: UCMP DSDP-596-P020-L39-2H

CODE VERSION: 2016-7-12, PROCESSED ON: 2016-12-21 at 15:32:37
Threshold of 0.12 and size filter of 100 - 4500 um
Directory: DSDP-596-P020-L39-2H-4W-31-33cm-g106_Hwell_N1of1_Mcompount_Oflat_I1_TzEDF-0_X5

DSDP-596-P020-L39-2H-4W-31-33cm-g106_Hwell_N1of1_obj00077

Fossil Only

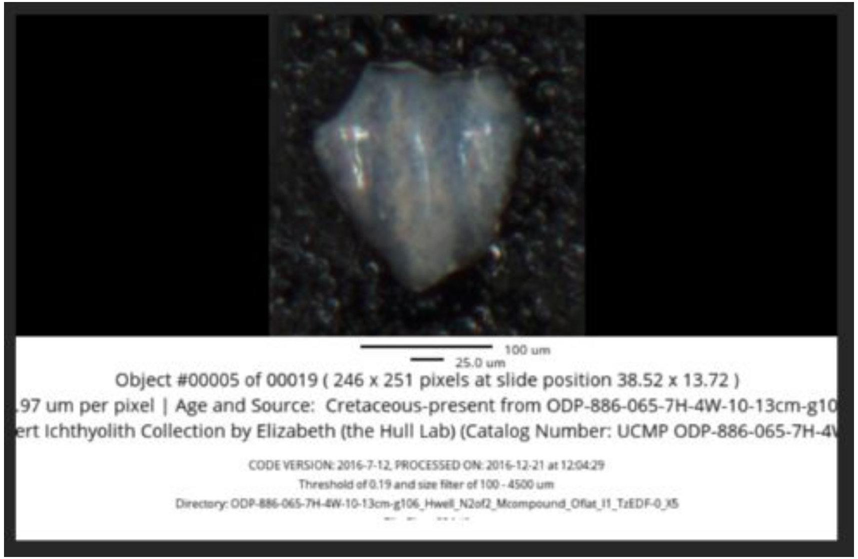

ODP-886-065-7H-4W-10-13cm-g106_obj00005_edf

Lobular Trident

The lobular trident has a lobed spade shape with one rounded cusps defined by the central ridge and two side ridges which are not associated with cusps and differ slightly in size and shape from eachother. The central cusp is longer and wider than the side cusps and defines the length of the crown.

Fossil Only

Lopsided Trident

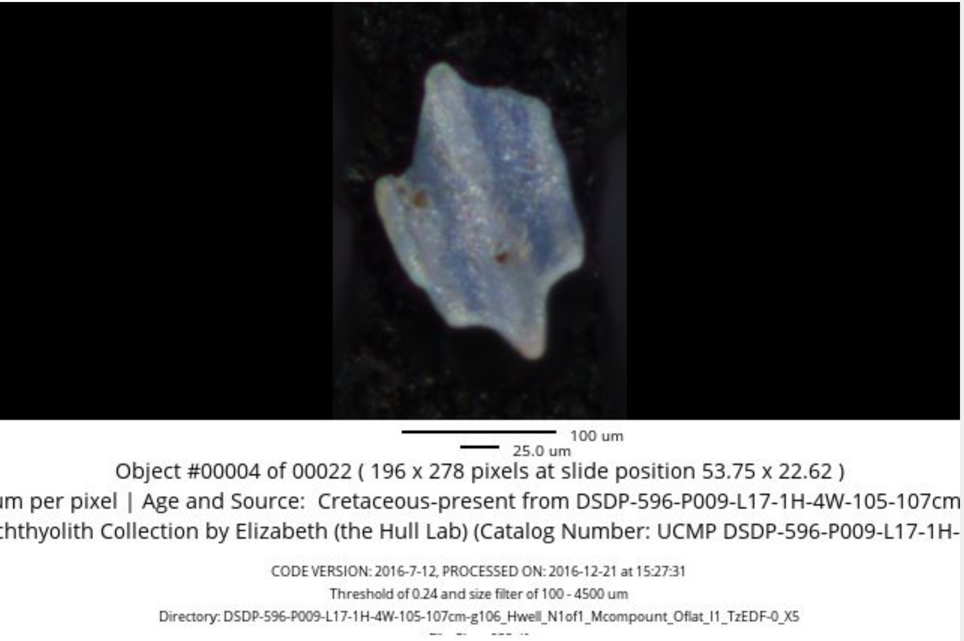

DSDP-596-P009-L17-1H-4W-105-107cm-g106_Hwell_N1of1_obj00004

The lopsided trident has a squared spade shape with three ridges and two cusps, associated with the central ridge and one of the side ridges. The crown is defined by parallel straight side ridges and a long central ridge which extends the length of the crown from the anterior vertex to posterior vertex.

Taxonomic Citation

Megachasma pelagios, Castro, Pg. 226
Squalus acanthias, Reif, Pg. 116, Fig. B3

Madeleine

The madeleine type has a stretched spade with a shape with six or more shallow linear ridges which begin at the rounded anterior creating a scalloped anterior edge texture and which terminate mid-crown and do not reach the posterior vertex.

Taxonomic Citation

Hexanchus griseus, Reif, Pg. 103, Fig. C1

Scyliorhinus retifer, Reif, Pg. 163, Fig. Cla

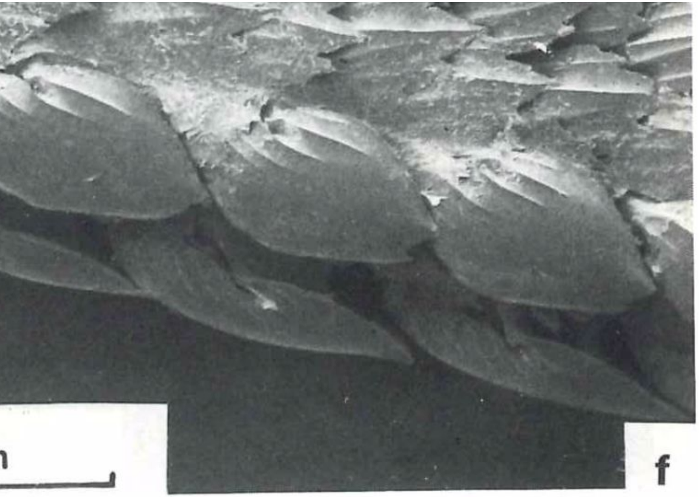

Hexanchus griseus, Reif, Pg. 103, Fig. C1

Modern Only

Madeleine Trident

The madeleine trident type has a stretched spade shape and three cusps with a longer and wider central cusp and two shorter side cusps which attach at the anterior of the crown and terminate ~⅔ of the length of the central section. Each cusp is associated with multiple shallow ridges which begin at the anterior of the crown and may or may not terminate mid-crown or at the posterior vertex.

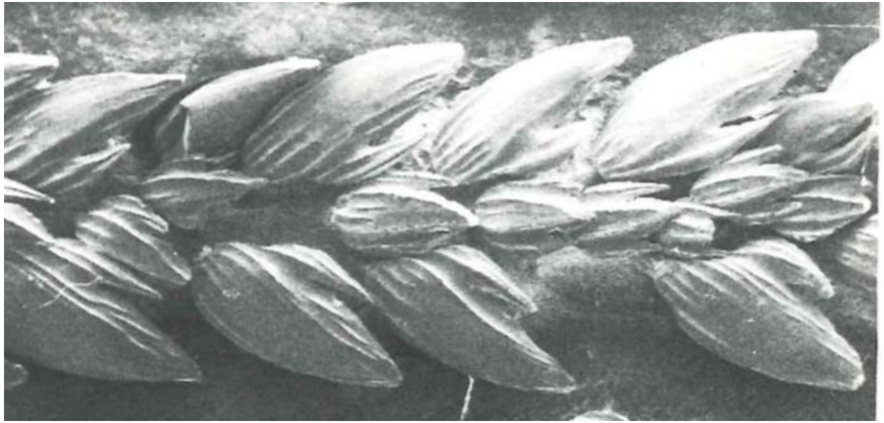

Galeus melastomus, Reif, Pg. 153, Fig. C1

Taxonomic Citation

Galeus melastomus, Reif, Pg. 153, Fig. C1

Galeus melastomus, Reif, Pg. 155 , Fig. C1

Modern Only

Many Celled

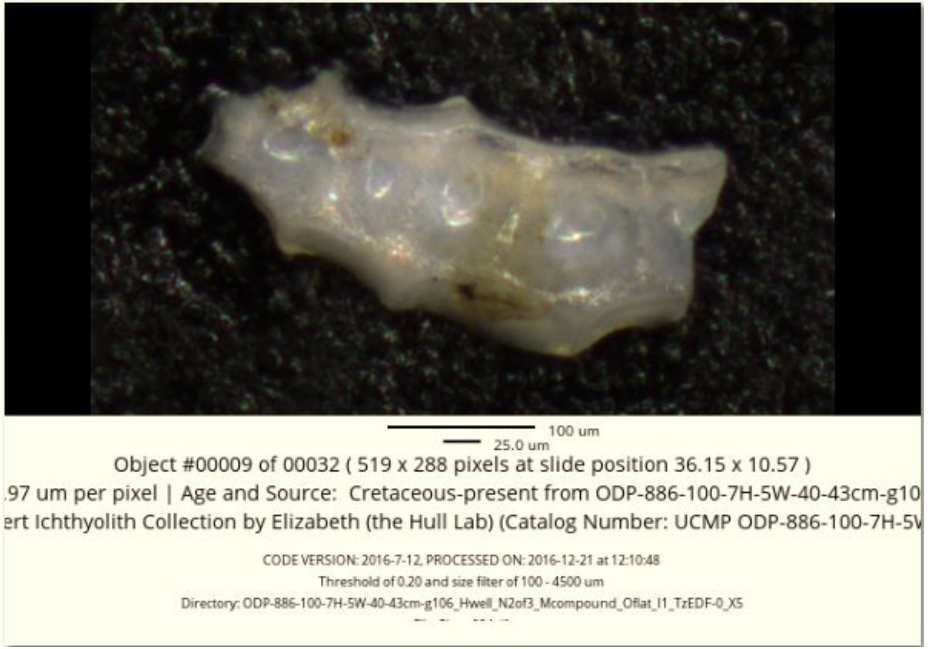

ODP-886-100-7H-5W-40-43cm-g106_obj00009_edf

The many celled denticle has a rectangular shape with unequal length and width. The crown is entirely composed of three or more dimples outlined by central ridge system shapes composed of four or five thin ridged edges.

Taxonomic Citation

Mollisquama mississippiensis, Lourtie et al. (2022)

Many Celled Central Complex Polygon

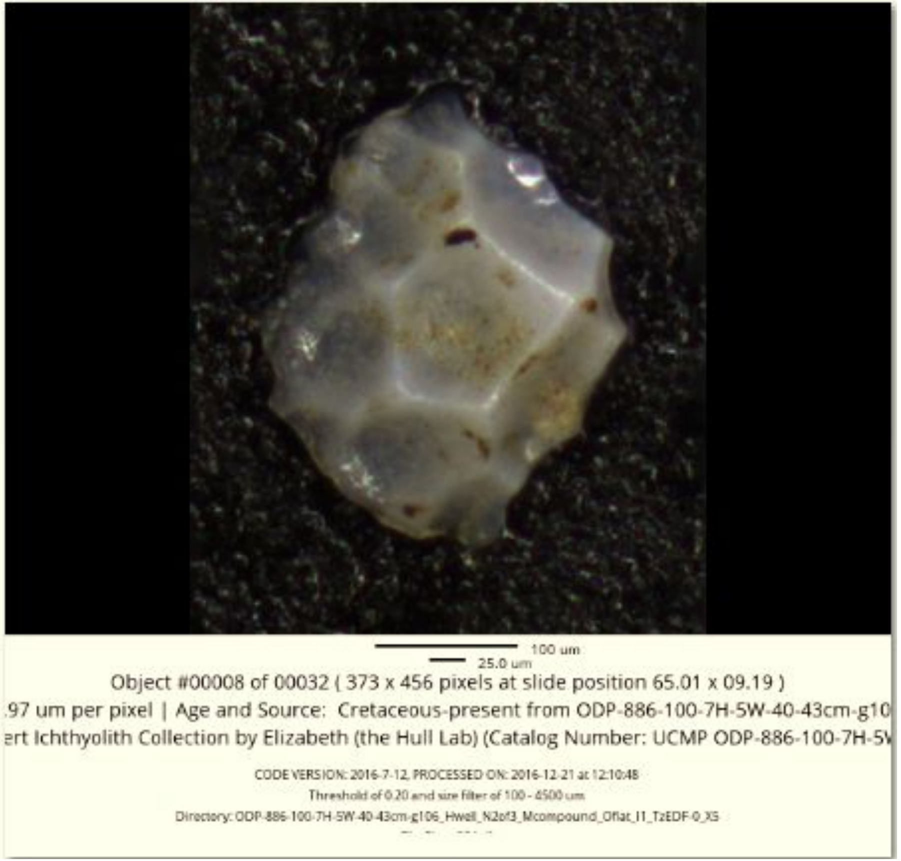

ODP-886-100-7H-5W-40-43cm-g106_obj00008_edf

The many celled central complex polygon has an irregular shape with no clear directionality. The crown is entirely composed of three of more dimples outlined by thin ridges surrounding a central ridge system shape with five or more edges and a central circular dimple.

Taxonomic Citation

Euprotomicrus bispinatus, Castro, Pg. 153
E. bispinatus, Reif, Pg. 124, Fig. B1

Many Ridged Linear Fan

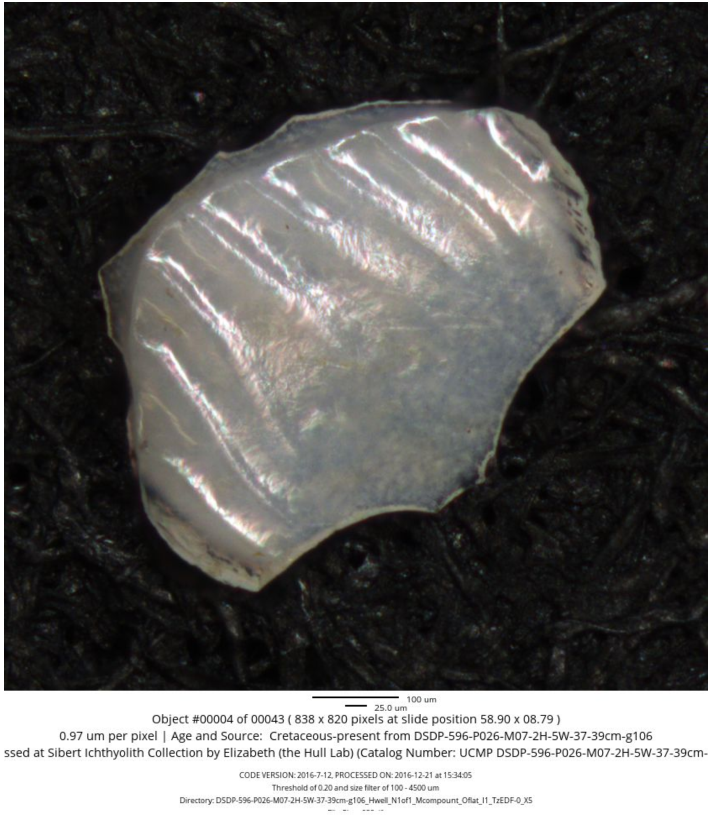

DSDP-596-P026-M07-2H-5W-37-39cm-g106_Hwell_N1of2_obj00004

The many ridged linear fan has a fan shape and is at least twice as wide as it is long. It has a rounded anterior and six or more ridges linear running ridges which may or may not terminate at the posterior or mid-crown. The crown may or may not have a central ridge but all ridges are similar in shape and length.

Taxonomic Citation

Alopias superciliosus, Reif, Pg. 141, Fig. H2

Scyliorhinus stellaris, Reif, Pg. 161, Fig. H1

Carcharhinus falciformis, Reif, Pg. 177, Fig. H1, H2, B1, and B4

Carcharhinus galapagensis, Reif, Pg. 181, Fig. H2, H3, and B1, Pg. 183, Fig. B2, B3, P2, P3

Carcharhinus obscurus, Reif, Pg. 187, Fig. C1 and C2

Carcharhinus amblyrhynchos, Reif, Pg. 202, Fig. B2, C1

Carcharhinus melanopterus, Reif, Pg. 206, Fig. B3 and Pg. 208, Fig. P1

Negaprion brevirostris, Reif, Pg. 225, Fig. N

Sphyrna tudes, Reif, Pg. 238, Fig. C1

Many Ridged Trident

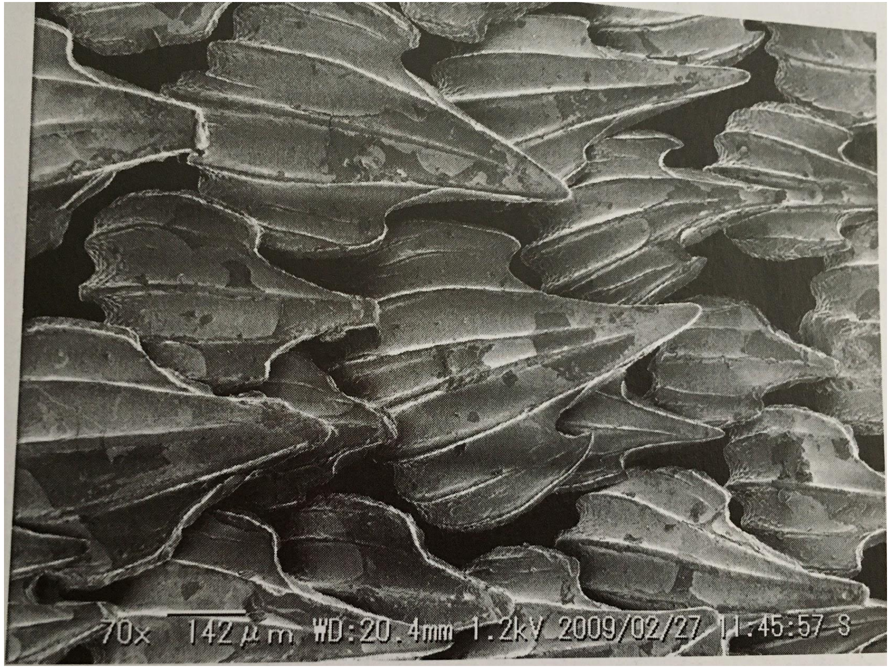

Scyliorhinus boa, Castro, Pg. 336

The many ridged trident has a pointed spade shape with a round scalloped anterior and a posterior vertex. The crown displays three cusps and five or more ridges. The three central ridges define the sides and center of the central cusp which is much longer and wider than the side cusps which are also each associated with a ridge. These ridges begin at the anterior but may or may not terminate mid-crown or at the posterior vertex.

Taxonomic Citation

Scyliorhinus boa, Castro, Pg. 336
Carcharhinus plumbeus, Reif, Pg. 194, Fig. M1
Scyliorhinus retifer, Gabler-Smith, M. et al. (2021), Fig. E (right)

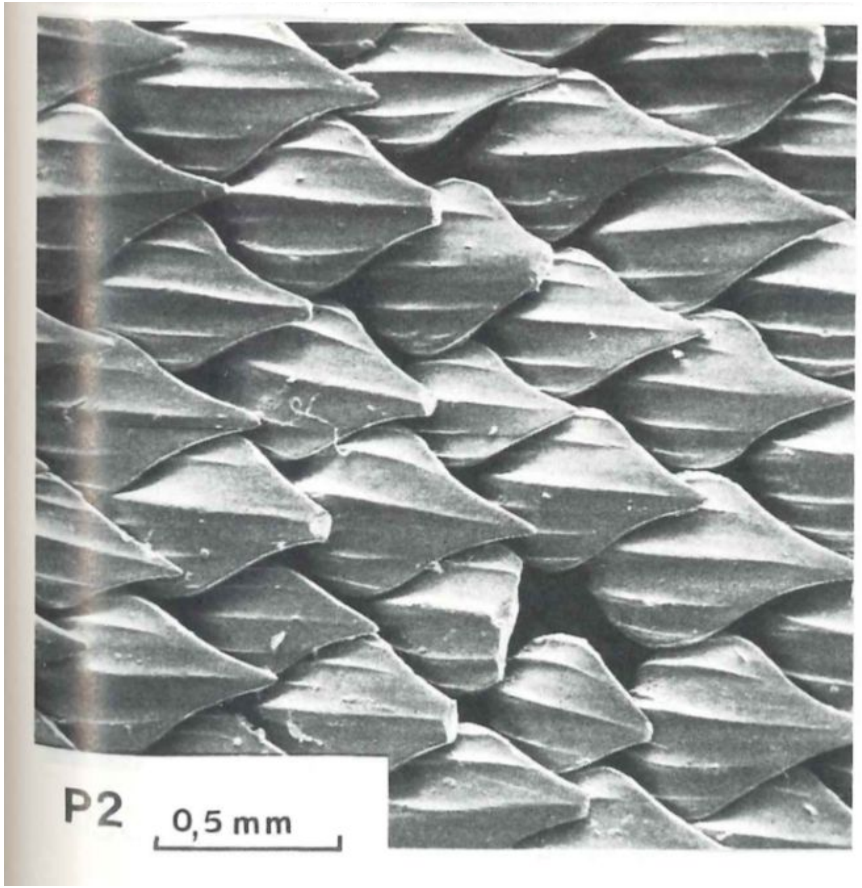

Scyliorhinus canicula, Reif, Pg. 159, Fig. P2

Many Ridged Petal

The many ridged petal has a stretched spade shape with a round scalloped anterior and a posterior vertex. The crown displays three cusps and five or more ridges. The three central ridges define the sides and center of the central cusp which is much longer and wider than the side cusps which are also each associated with a ridge. These ridges begin at the anterior but may or may not terminate mid-crown or at the posterior vertex.

Taxonomic Citation

Scyliorhinus canicula, Reif, Pg. 159, Fig. P2
Galeus melastomus, Reif, Pg. 155, Fig. B4
Scyliorhinus stellaris, Reif, Pg. 161, Fig. C3
Scyliorhinus retifer, Gabler-Smith, M. et al. (2021), Fig. B
Scyliorhinus retifer, Gabler-Smith, M. et al. (2021), Fig. E (left)

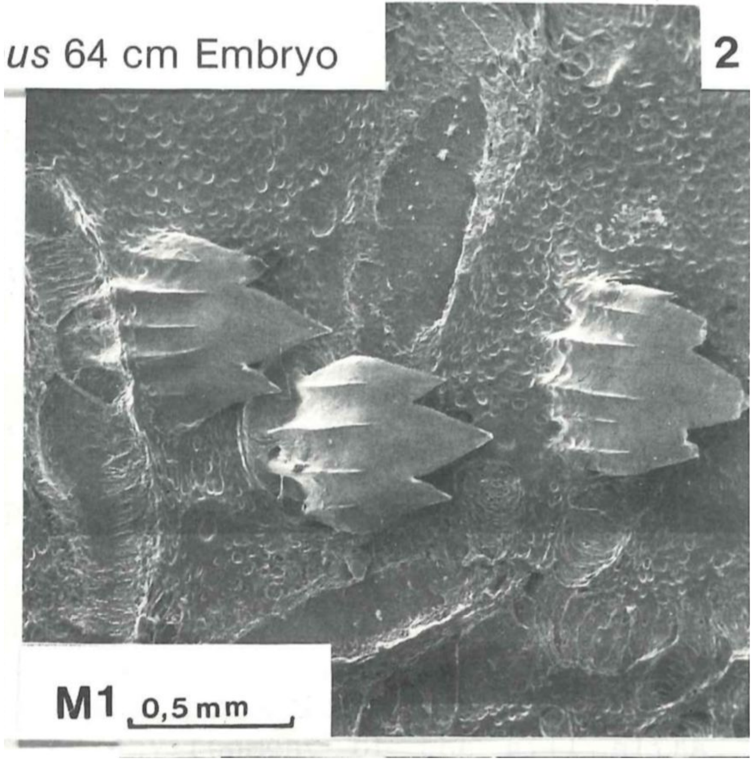

Hexanchus griseus, Reif, Pg. 105, Fig. M1

Short Many Ridged Trident

The many ridged trident's crown has three distinct cusps at the posterior and 5 or more ridges which run from the anterior towards the posterior but do not define the cusps.

Taxonomic Citation

Hexanchus griseus, Reif, Pg. 105, Fig. M1
Carcharhinus galapagensis, Reif, Pg. 183, Fig. M1
Carcharhinus plumbeus, Reif, Pg. 197, Fig. M1
Prionace glauca, Reif, Pg. 218, Fig. P3
Galeocerdo cuvier, Reif, Pg. 23, Fig. M1
Sphyrna zygaena, Reif, Fig. M2

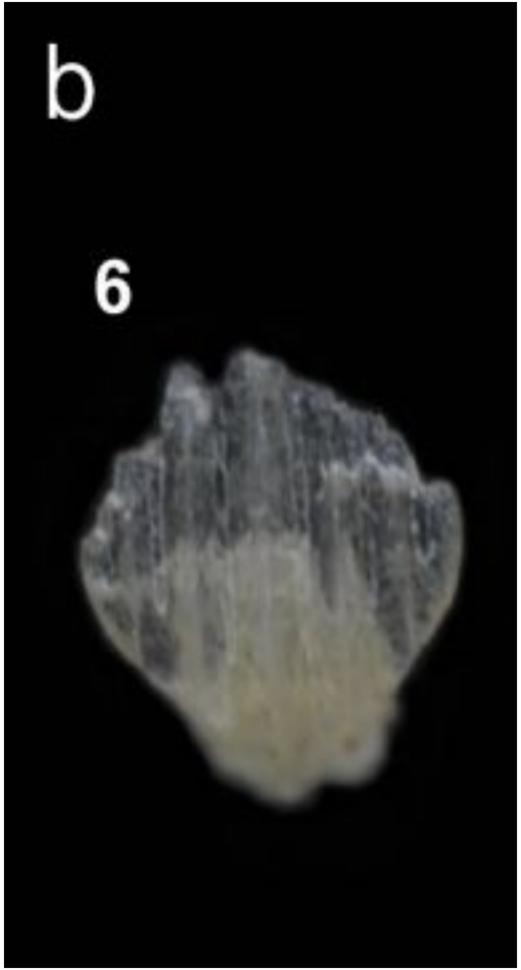

Fossil b.6, Dillon, E. M., Norris, R. D., & Dea, A. O. (2017)

Many Thin Ridged Spade

The many thin ridged spade has a pointed spade shape and five or more ridges which extend from the anterior vertex to the posterior creating a scalloped posterior edge texture.

Taxonomic Citation

Carcharhinus isodon, Castro, Pg. 421

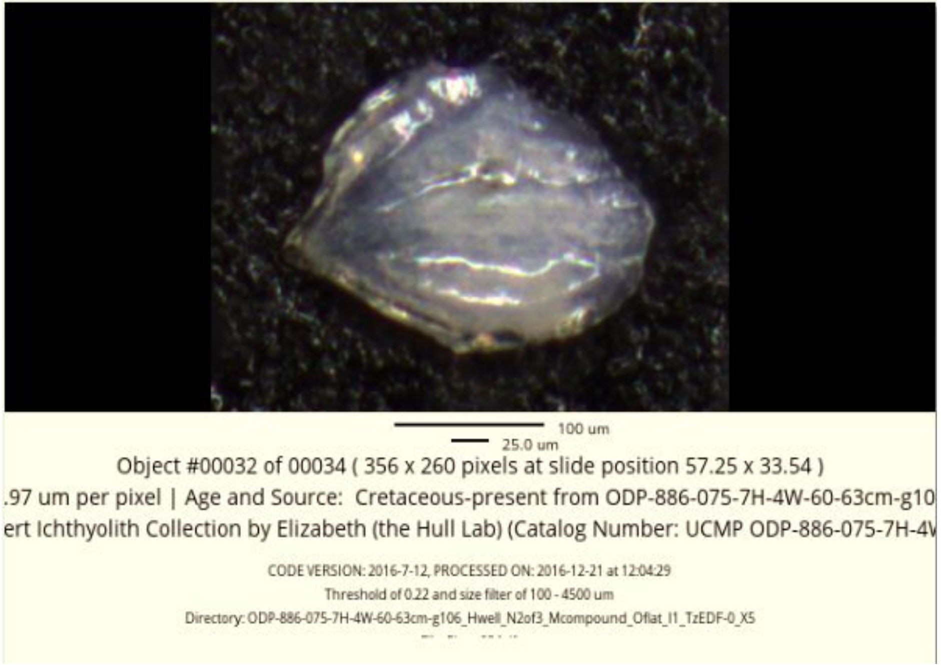

ODP-886-075-7H-4W-60-63cm-g106_obj00032_edf

Minnie Indented Shell

The minnie indented shell has a rounded spade shape with an anterior vertex and a rounded posterior. This type is characterized by shallow linear ridges diverging from the anterior vertex and which may or may not begin halfway down the length of the crown.

Taxonomic Citation

Alopias superciliosus, Reif, Pg. 143, Fig. M1
Carcharhinus amblyrhynchos, Reif, Pg. 200, Fig. H1
C. falciformis, Reif, Pg. 175, Fig. N
C. galapagensis, Reif, Pg. 181, Fig. H1
C. melanopterus, Reif, Pg. 208, Fig. C1
C. plumbeus, Reif, Pg. 190, Fig. N
Centrophorus a., Castro, Pg. 84, Fig. 17 d
C. b., Castro, Pg. 85, Fig. 18 d
C. tessellatus, Castro, Pg. 79, Fig. 15 D
C. niaukang, Castro, Pg. 73, Fig. 13 d
C. isodon, Castro, Pg. 71, Fig. 12 e
C. uyato, Castro, Pg. 81, Fig. 16 e
Chiloscyllium plagiosum, Reif, Pg. 130, Fig. B1
C. punctatum, Reif, Pg. 132, Fig. H2
Galeorhinus galeus, Reif, Pg. 171, Fig. M2
Mustelus canis, Ankhelyi et al., Pg. 6, Fig. a, b, and f
M. mustelus, Reif, Pg. 167, Fig. M2 and N

Minnie Indented Shell

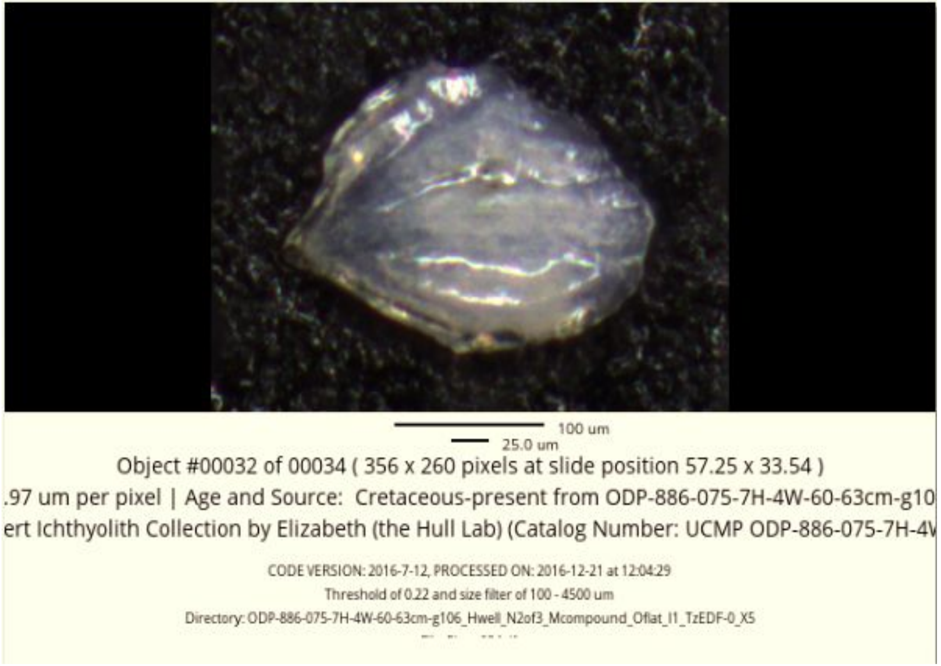

ODP-886-075-7H-4W-60-63cm-g106_obj00032_edf

The minnie indented shell has a rounded spade shape with an anterior vertex and a rounded posterior. This type is characterized by shallow linear ridges diverging from the anterior vertex and which may or may not begin halfway down the length of the crown.

Taxonomic Citation

Negaprion brevirostris, Reif, Pg. 225, Fig. P4

Odontaspis taurus, Reif, Pg. 137, Fig. H1

Prionace glauca, Reif, Pg. 217, Fig. N, Pg. 218, Fig. M1, Pg. 220, Fig. N

Sphyrna zygaena, Reif, Pg. 244, Fig. N

Triaenodon obesus, Reif, Pg. 210, Fig. H2, N and Pg. 212, Fig. H3

Minnie Ridged Circle

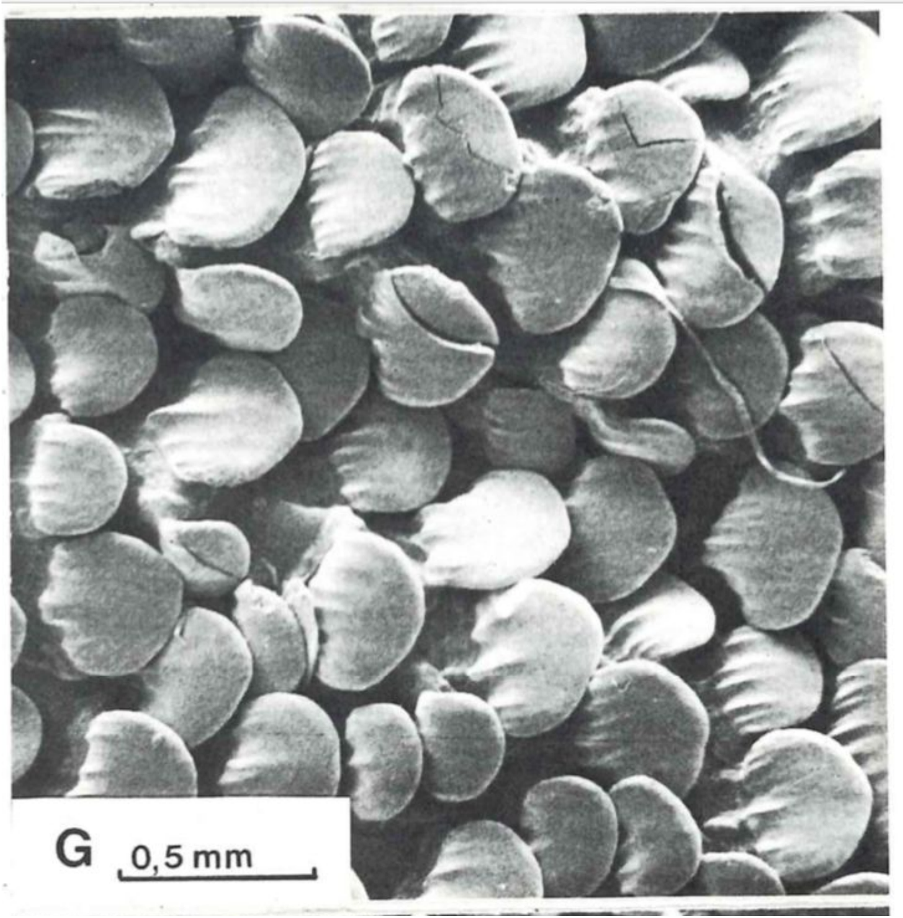

Alopias superciliosus, Reif, Pg. 143, Fig. G

The minnie ridged circle type has a circular shape with a round scalloped anterior and a smooth rounded posterior. The crown has six or more shallow thin ridges which begin at the anterior and terminate ~ ⅓ the length of the crown.

Taxonomic Citation

Alopias superciliosus, Reif, Pg. 143, Fig. G
Carcharhinus falciformis, Reif, Pg. 175, Fig. N
Carcharhinus melanopterus, Reif, Pg. 204, Fig. H1
Carcharhinus melanopterus, Reif, Pg. 206, Fig. N
Negaprion brevirostris, Reif, Pg. 224, Fig. H1

Nub Spine

The nub spine type has a spine shape with a smooth circular anterior and a vertically projecting round posterior vertex, or “nub”

Taxonomic Citation

Dipturus batis, Gravendeel, Neer, and Brinkhuizen 18 (lateral)
Centroscyllium nigrum, Lourtie et al. (2022)

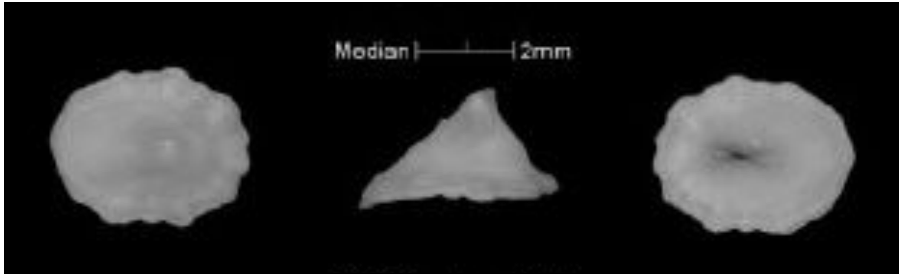

Dipturus batis, Gravendeel, Neer, and Brinkhuizen, Fig. 18 (lateral)

Modern Only

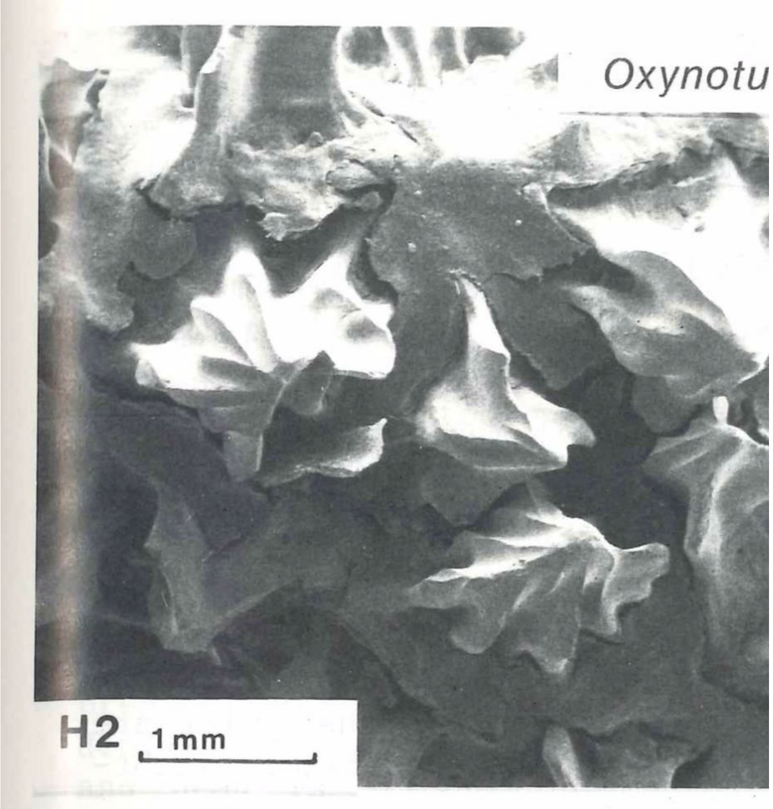

Oxynotus centrina, Reif, Pg. 120, Fig. H2

Oak Leaf

The oak leaf type has an irregular shape with a round scalloped anterior and multiple posterior "bumps" or points which create a scalloped edge texture. The center of the crown is elevated and has approximately 6 meandering ridges in a pinnate pattern which branch away from this central area diverging from the anterior.

Taxonomic Citation

Squalus cubensis, Castro, Pg. 63
Oxynotus centrina, Reif, Pg. 120, Fig. H2

Modern Only

Oblong Branching Crown

The oblong branching crown has a squared spade shape and the crown is completely defined by five thin outer ridges creating a pentagon shape. The denticle lacks clear directionality but has three shorter ridges, one of which is flat and on either side is a longer straight ridge which connects to two shorter ridges which create a vertex opposite the flat ridge.

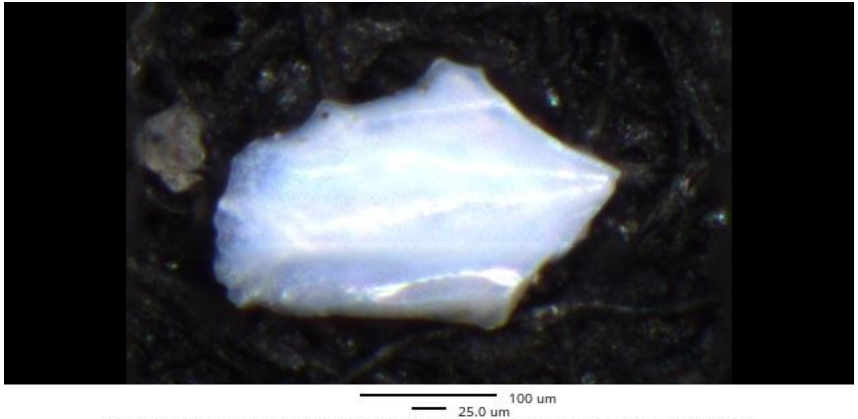

Object #00011 of 00069 (456 x 288 pixels at slide position 50.41 x 18.09)
um per pixel | Age and Source: Cretaceous-present from DSDP-596-P042-M41-2H-6W-85-87cm-
chthyolith Collection by Elizabeth (the Hull Lab) (Catalog Number: UCMP DSDP-596-P042-M41-2H

CODE VERSION: 2016-7-12, PROCESSED ON: 2016-12-21 at 15:53:43
Threshold of 0.19 and size filter of 100 - 4500 um
Directory: DSDP-596-P042-M41-2H-6W-85-87cm-g106_Hwell_N1of1_Mcompount_Oflat_I1_TzEDF-0_X5

DSDP-596-P042-M41-2H-6W-85-87cm-g106_obj00011

Fossil Only

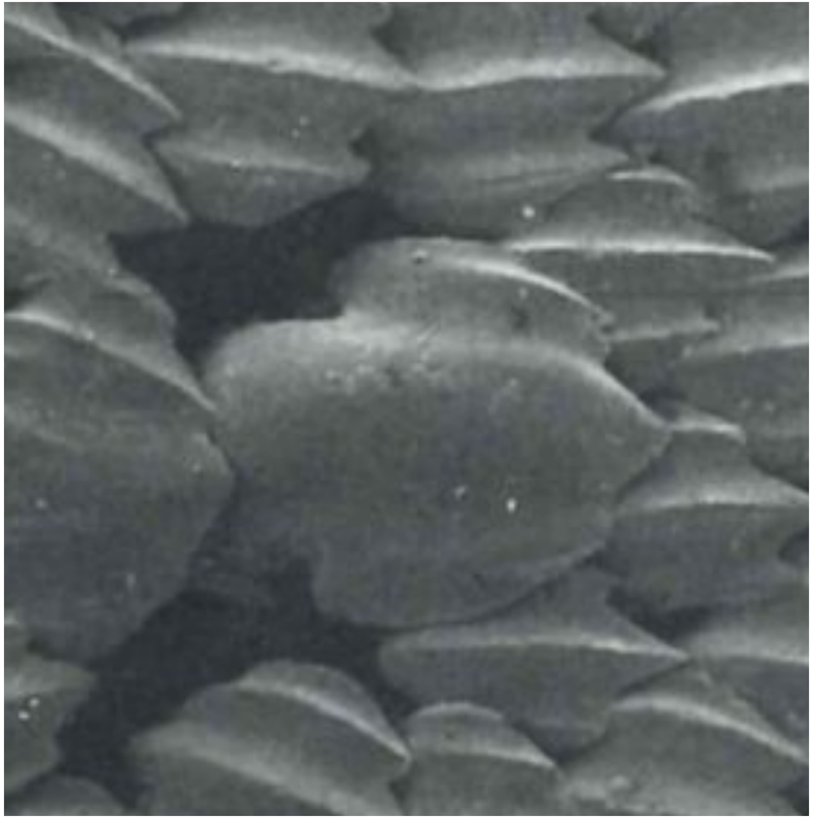

Carcharodon carcharias, Reif, Pg. 151, Fig. B5
(Central Large)

Pear Shaped Wedge

The pear shaped wedge has a pointed spade shape with one plain of symmetry and three cusps which are all defined by ridges. The central ridge is about two times longer and wider than the two side ridges and has a rounded anterior and a posterior vertex. The two side ridges begin ~a quarter of the length of the central ridge and converge slightly posterior.

Taxonomic Citation

Isurus oxyrinchus, Reif, Pg. 145, Fig. B5
Carcharodon carcharias, Reif, Pg. 151, Fig. B5

Modern Only

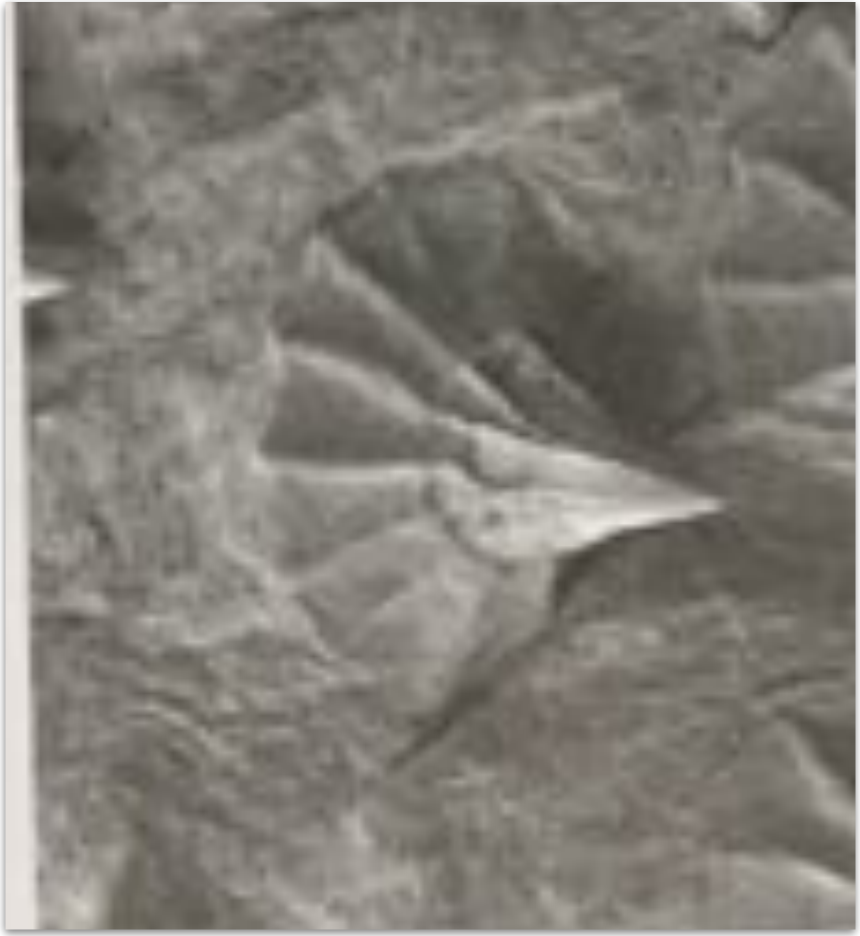

Centroscyllium nigrum, Castro, Pg. 96

Radial Angled Spines

The radial angled spine type is characterised by a vertical projecting crown with a posterior vertex and a round circular anterior. Ridges encircle the crown converging from the anterior to the posterior vertex.

Taxonomic Citation

Echinorhinus brucus, Castro, Pg. 44
Echinorhinus cookei, Castro, Pg. 47
Centroscyllium fabricii, Castro, Pg. 93
Centroscyllium nigrum, Castro, Pg. 96
Echinorhinus brucus, Reif, Pg. 135
Scyliorhinus retifer, Reif, Pg. 163, Fig. C3
Etmopterus molleri, Lourtie et al. (2022)

Modern Only

Raised Dimple

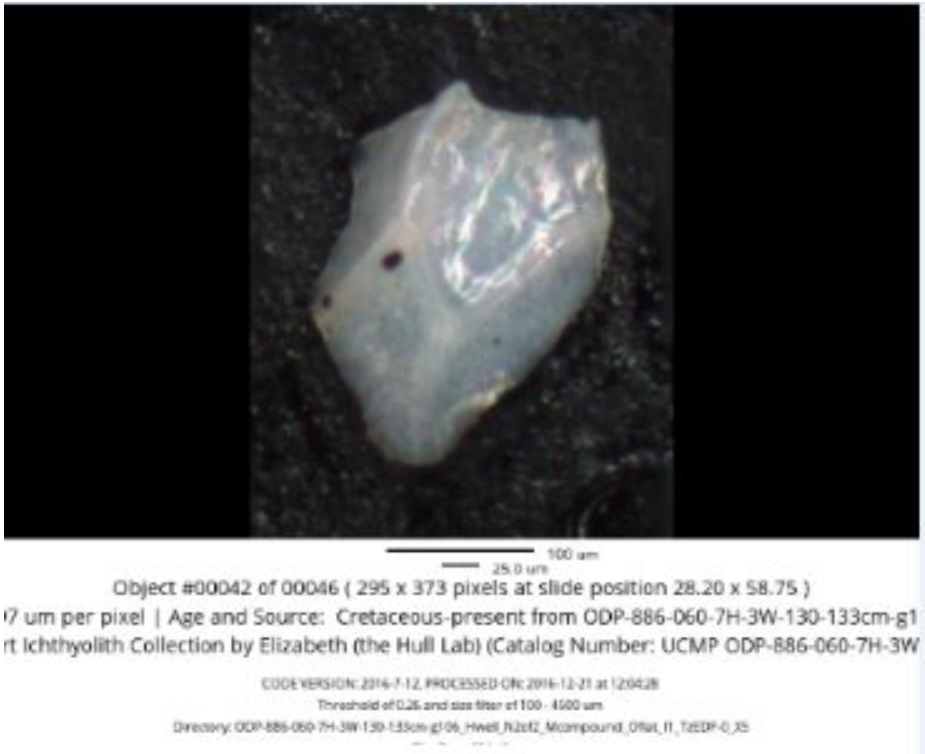

ODP-886-060-7H-3W-130-133cm-g106_obj00042_edf

The raised dimple type has an oval-like shaped crown which is composed entirely by one dimple surrounded by thin raised ridges which connect to vertical ridges that run down the height of the crown.

Fossil Only

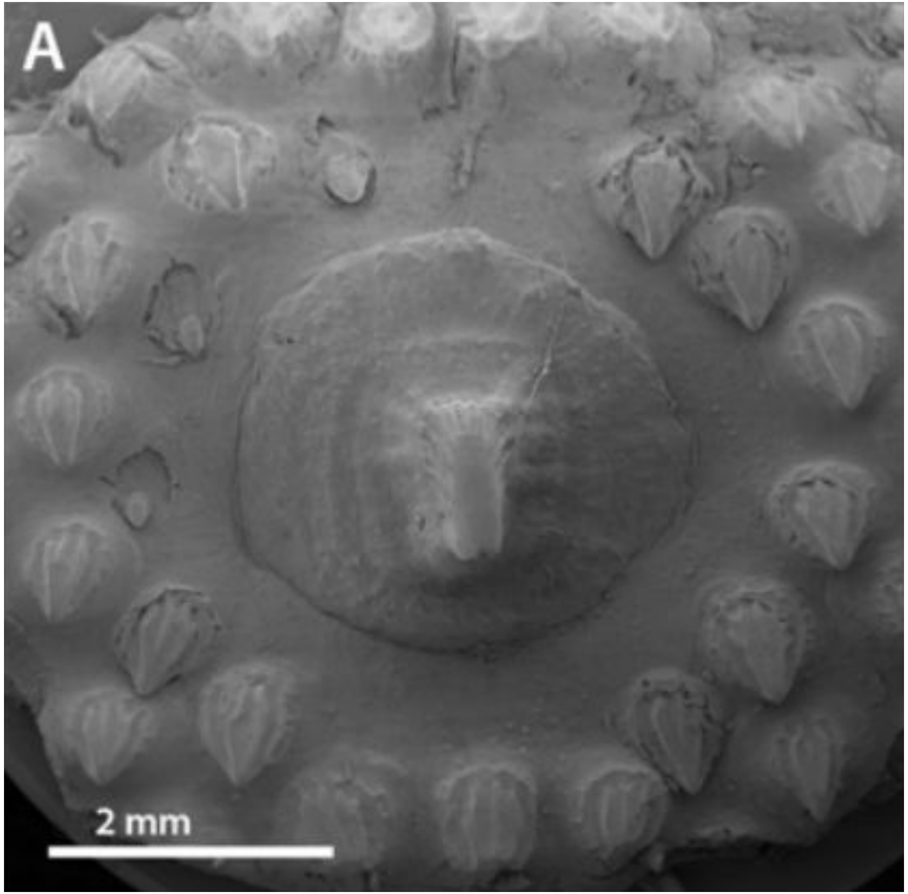

Squatina guggenheim, Vaz, D. F., & De Carvalho, M. R. (2013), Fig. A (center)

Raised Rectangle

The raised rectangle has a rectangular shape at the center of a circular mound. The crown is composed of a smooth flat plateau with striations along its vertical edges.

Taxonomic Citation

Squatina guggenheim, Vaz, D. F., & De Carvalho, M. R. (2013), Fig. A (center)

Modern Only

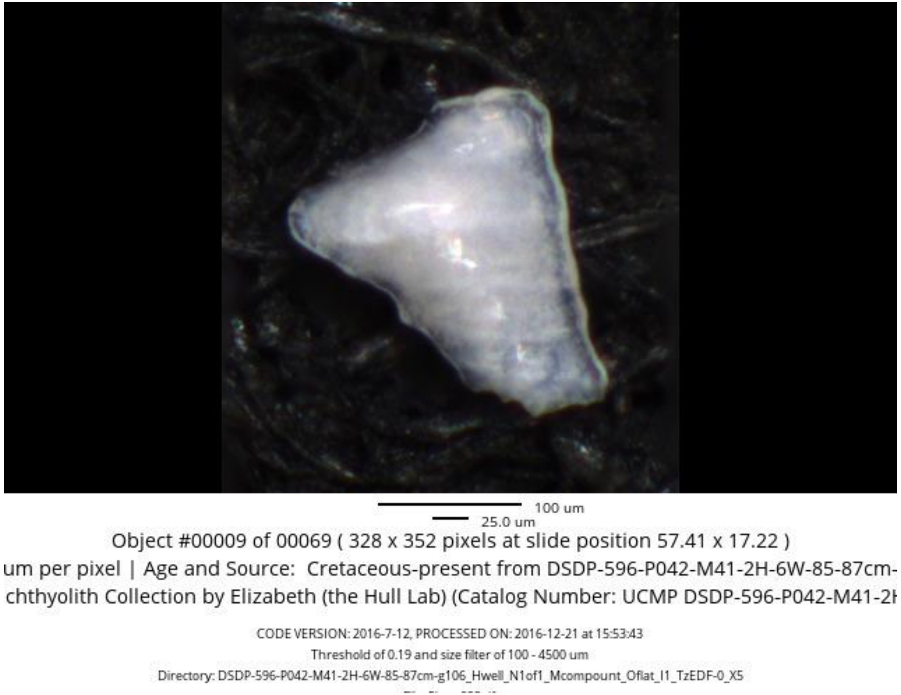

DSDP-596-P042-M41-2H-6W-85-87cm-g106_obj00009

Rake

The rake type has a squared spade shape with an anterior vertex and a flat posterior edge. The crown is defined by its asymmetry and has 5 ridges, the longest and widest of which is off to one of the sides of the crown rather than found in the center as is more common. The ridges define the entire crown and are clearly defined.

Fossil Only

Ridged Circle

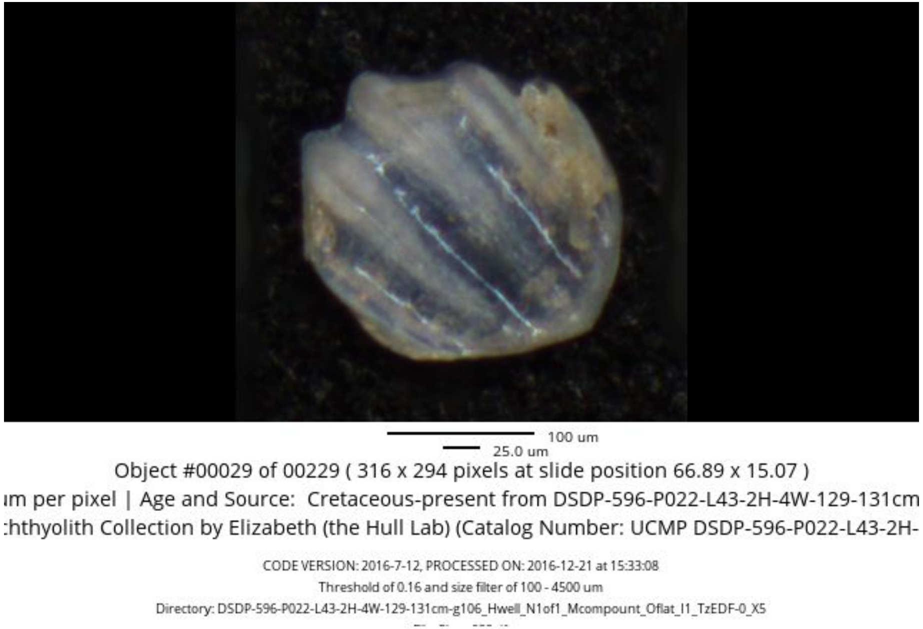

DSDP-596-P022-L43-2H-4W-129-131cm-g106_Hwell_N1of42_obj00029

The ridged circle type has a circular shape with shallow scalloped anterior and a very shallow wide posterior vertex. There can be three to five ridges which all extend the length of the crown with a longer central ridge and ridges which become gradually shorter towards the side.

Taxonomic Citation

Alopias superciliosus, Reif, Pg. 141, Fig. H and H2

A. vulpinus, Reif, Pg. 139, Fig. P1

Carcharhinus altimus, Castro, Pg. 400

C. amblyrhynchos, Reif, Pg. 199, Fig. H1, H2, H3, C1, H1, H2, H3, C1, and Pg. 200, Fig. P1 and N

C. cerdale, Castro, Pg. 410

C. falciformis, Reif, Pg. 175, Fig. P1

C. melanopterus, Reif, Pg. 206, Fig. N

C. plumbeus, Castro, Pg. 453

C. plumbeus, Reif, Pg. 189, Fig. H1, B1 and Pg. 190, Fig. P1

Carcharodon carcharias, Reif, Pg. 151, Fig. C1

Ginglymostoma cirratum, Castro, Pg. 184

G. unami, Castro, Pg. 191

Nebrius ferrugineus, Reif, Pg. 132, Fig. C1

Prionace glauca, Reif, Pg. 214, Fig. H2 and Pg. 218, Fig. B3

Sphyrna tudes, Reif, Pg. 235, Fig. H1

S. zygaena, Reif, Pg. 240, Fig. P1

Triaenodon obesus, Reif, Pg. 210 H2

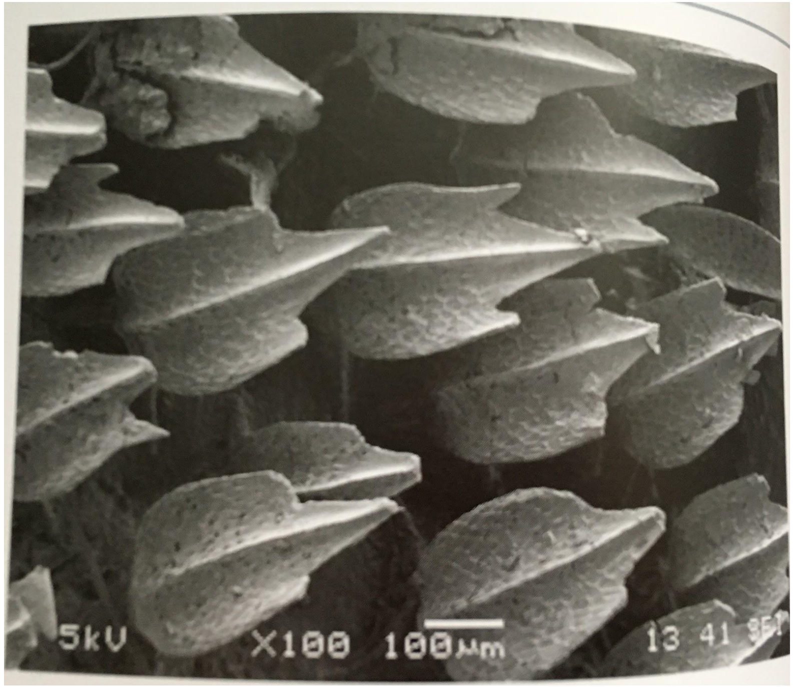

Apristurus profundorum, Castro, Pg. 311

Round Bottomed Textured Tulip

The round bottomed textured tulip has a pointed spade shape with a rounded anterior and a pointed posterior. The posterior is composed of three cusps the middle of which is longer and wider and defined by the crowns singular central ridge. The crown also has a honeycomb-like surface texture which covers the entire crown.

Taxonomic Citation

Apristurus profundorum, Castro, Pg. 311
Apristurus riveri, Castro, Pg. 314
Apristurus sp., Castro, Pg. 316

Modern Only

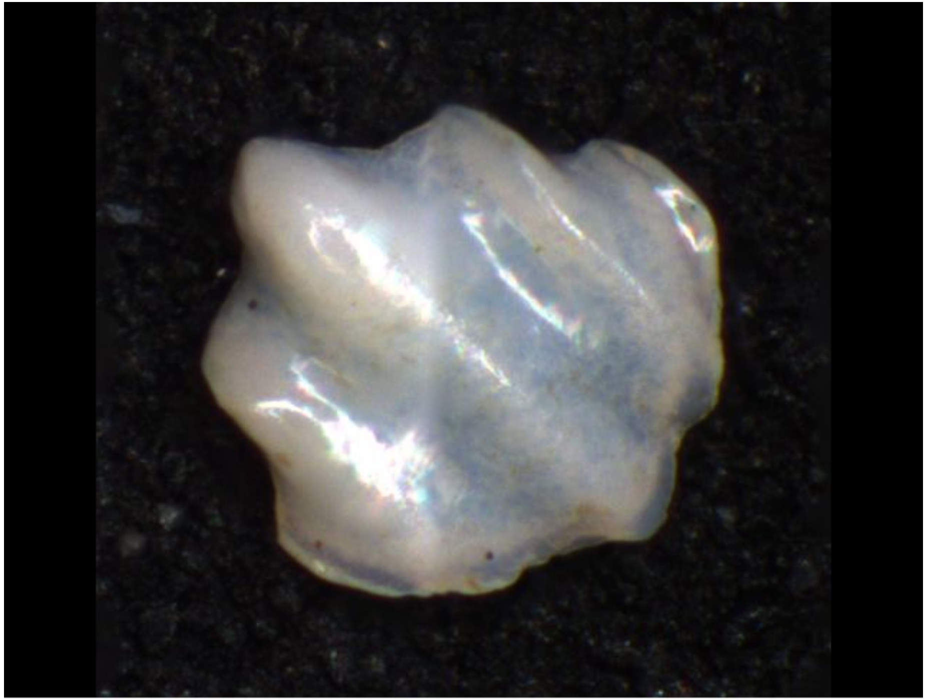

100 um
25.0 um
Object #00015 of 00199 (512 x 484 pixels at slide position 37.95 x 07.20)
um per pixel | Age and Source: Cretaceous-present from DSDP-596-P020-L40-2H-4W-55-57cm-[illegible]
Ichthyolith Collection by Elizabeth (the Hull Lab) (Catalog Number: UCMP DSDP-596-P020-L40-2[illegible]

CODE VERSION: 2016-7-12, PROCESSED ON: 2016-12-21 at 15:32:36
Threshold of 0.13 and size filter of 100 - 4500 um
Directory: DSDP-596-P020-L40-2H-4W-55-57cm-g106_Hwell_N1of1_Mcompount_Oflat_I1_TzEDF-0_X5
[illegible]

DSDP-596-P020-L40-2H-4W-55-57cm-g106_Hwell_N1of1_obj00015

Round Ridged Trident

The round ridged trident has a round spade shape with three wide and round ridges and one short cusp defined by the central ridge. The central ridge is wider and longer than the side ridges and the crown may or may not have a ridge outgrowth at the posterior.

Fossil Only

Round Sided Trident

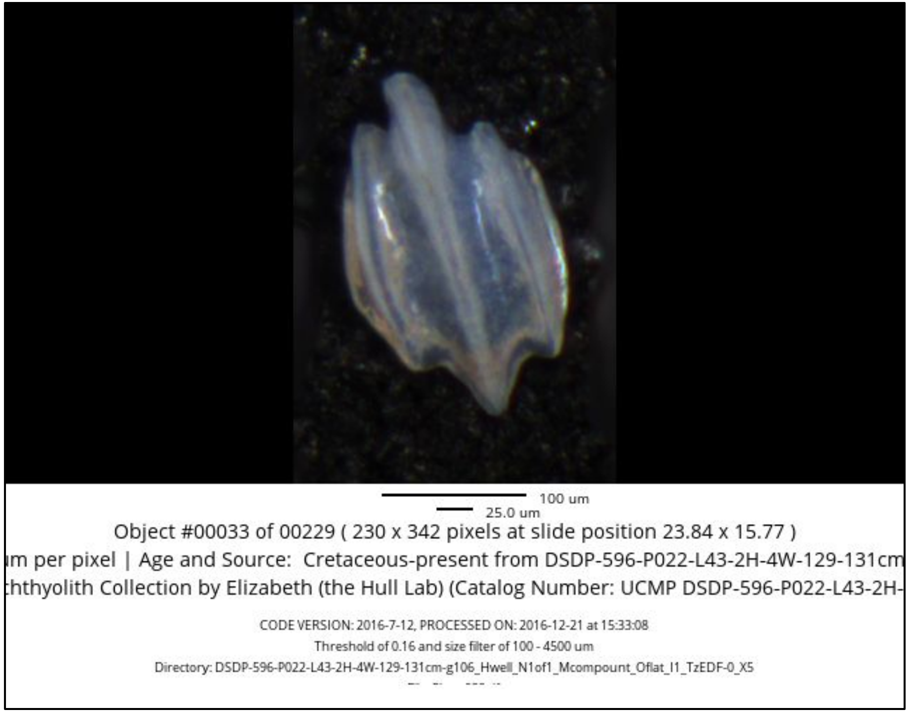

DSDP-596-P022-L43-2H-4W-129-131cm-g106_Hwell_N1of43_obj00033

The round sided trident has a rounded spade shape with three cusps defined by three ridges which extend the length of the crown. The central ridge bisects the crown and is longer than the side ridges. The side ridges may be straight or may define the curved lateral edges of the crown, diverging and then converging from the anterior.

Taxonomic Citation

Hexanchus griseus, Castro, Pg. 31

H. nakamurai, Castro, Pg. 36

Odontaspis ferox, Castro, Pg. 215

O. taurus, Reif, Pg. 137, Fig. M2

Squalus acanthias, Castro, Pg. 55

Triakis scyllium , Reif, Pg. 169, Fig. B2

T. semifasciata Gabler-Smith et al. (2021), Fig. F

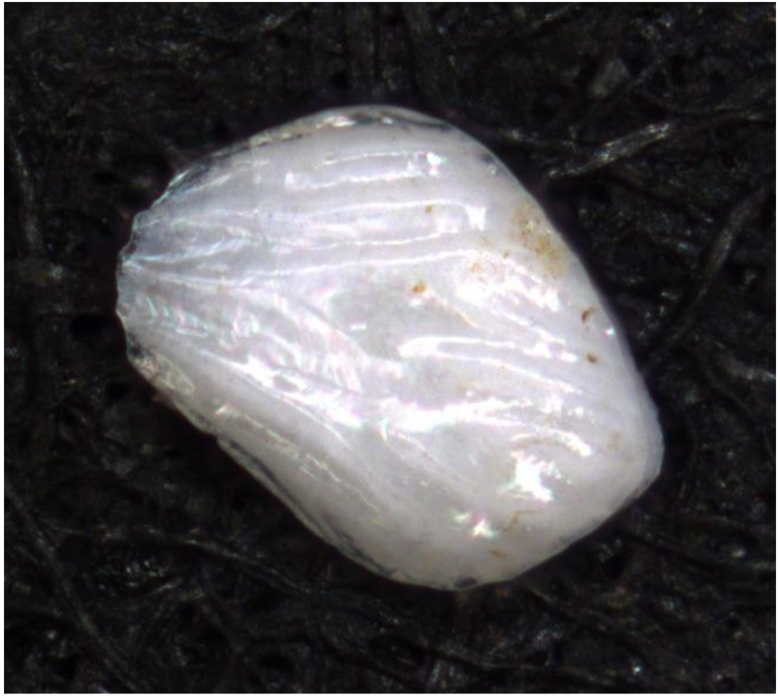

100 um
25.0 um
Object #00014 of 00108 (663 x 594 pixels at slide position 17.23 x 09.77)
7 um per pixel | Age and Source: Cretaceous-present from DSDP-596-P043-M43-2H-6W-95-97cm-g
t Ichthyolith Collection by Elizabeth (the Hull Lab) (Catalog Number: UCMP DSDP-596-P043-M43-2H-

CODE VERSION: 2016-7-12, PROCESSED ON: 2016-12-21 at 15:53:43
Threshold of 0.16 and size filter of 100 - 4500 um
Directory: DSDP-596-P043-M43-2H-6W-95-97cm-g106_Hwell_N1of1_Mcompount_Oflat_I1_TzEDF-0_X5

DSDP-596-P043-M43-2H-6W-95-97cm-g106_obj00014

Shallow Ridged Branching Fan

The shallow ridged branching fan has a fan shape and is asymmetric with 5 or more ridges which diverge into more ridges from the thinner anterior of the denticle. The ridges are meandering and while their width is consistent their length is random. Nearly the entire crown is covered in ridges.

Fossil Only

Shallow Ridged Circle

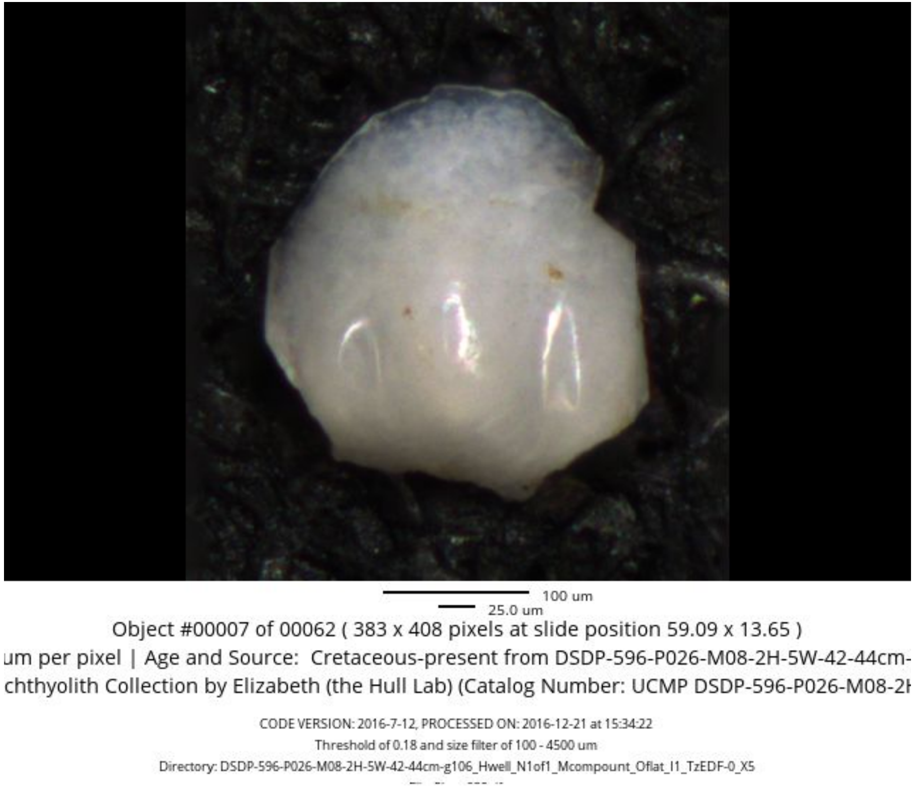

DSDP-596-P026-M08-2H-5W-42-44cm-g106_Hwell_N1of1_obj00007

The shallow ridged circle type has a circular shape with three wide shallow ridges which extend ~ ⅓ the length of the crown.

Fossil Only

Shallow Ridged Wide Crown

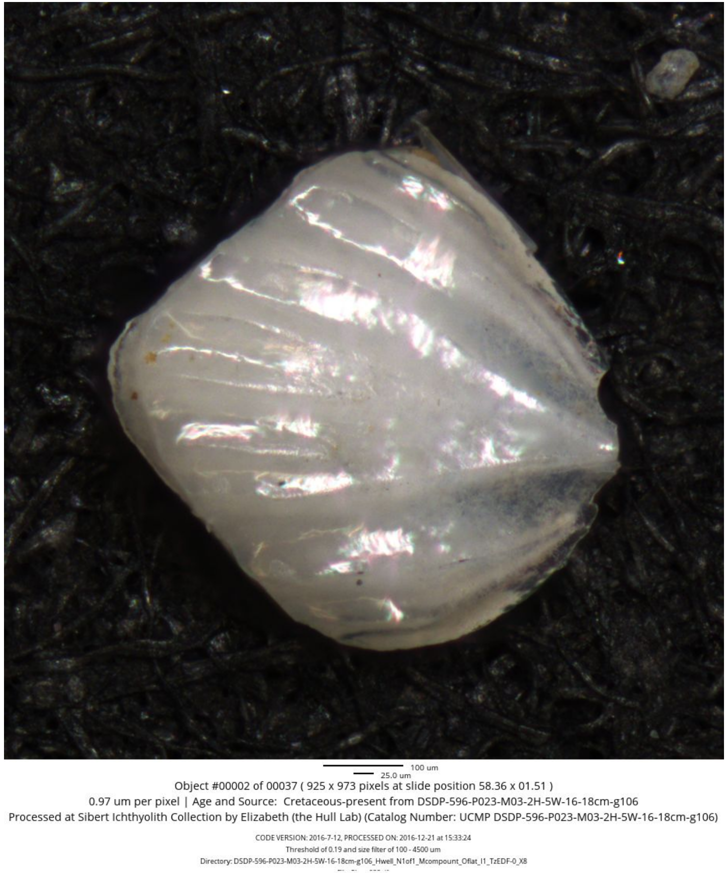

DSDP-596-P023-M03-2H-5W-16-18cm-g106_Hwell_N1of1_obj00002

The shallow ridged wide crown type has a fan shape, which is wider than it is long, and has five or more ridges which radiate from the anterior to the edges of the crown and run differing lengths.

Taxonomic Citation

Carcharhinus amblyrhynchos, Reif, Pg. 202, Fig. B1
C. obscurus, Reif, Pg. 185, Fig. C1
C. plumbeus, Reif, Pg. 192, Fig. C1, Pg. 194, Fig. C1, and Pg. 196, Fig. H2, H3, B4, and C1
Centrophorus granulosus, Reif, Pg. 112, Fig. H1, B1, and Cla
Chiloscyllium punctatum, Reif, Pg. 132, Fig. B1
Odontaspis taurus, Reif, Pg. 137, Fig. H3
Prionace glauca, Reif, Pg. 214, Fig. H3, Pg. 215, Fig. C2, Pg. 217, Fig. H3, B1, and Pg. 220, Fig. H3
Triaenodon obesus, Reif, Pg. 212, Fig. B1

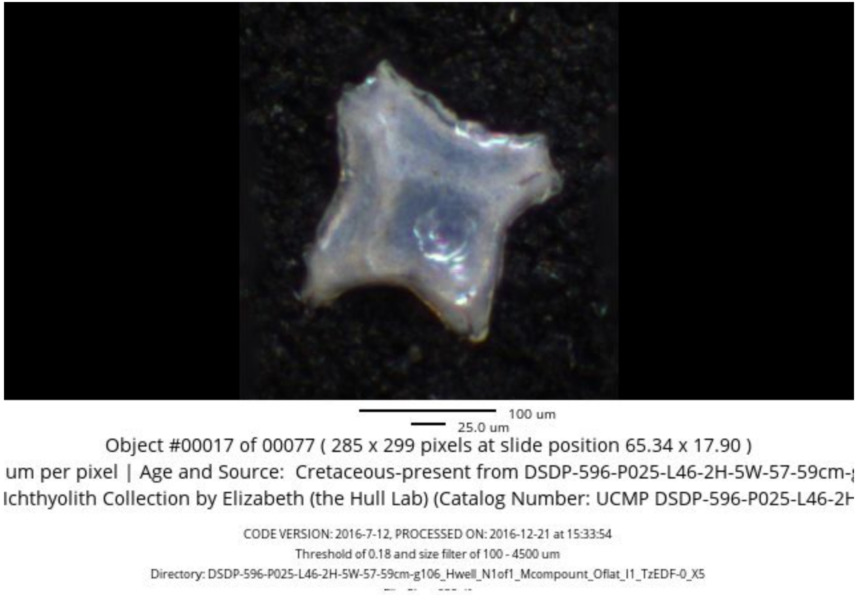

DSDP-596-P025-L46-2H-5W-57-59cm-g106_Hwell_N1of1_obj00017

Sharp Kite

The sharp kite type has a fusiform shape with a roughly equal length and width. It is defined by its four sided central ridge system shape that outlines one central circular dimple. This type is also distinguishable based on the pronounced ridges with a triangular profile which make the central shape appear “sharp”.

Taxonomic Citation

Isistius brasiliensis, Reif, Pg. 124, Fig. B2

I. plutodus, Castro, Pg. 151

Squaliolus laticaudus, Castro, Pg. 156

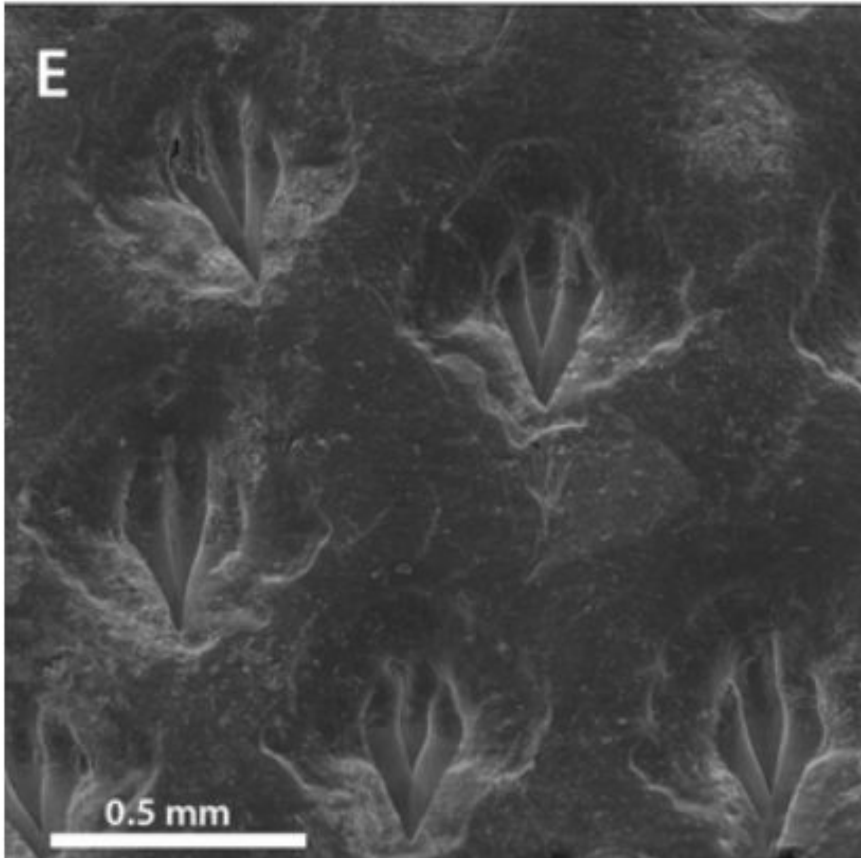

Squatina argentina, Vaz, D. F., & De Carvalho, M. R. (2013), Fig. E

Short Troughed Diamond

The short troughed diamond has a diamond-like shape and is defined by its two ridges which extend and converge from the crown's anterior ~⅔ the length of the crown. The crown emerges from the skin on a raised mound.

Taxonomic Citation

Squatina argentina, Vaz, D. F., & De Carvalho, M. R. (2013), Fig. E
Somniosus cf. pacificus, Vaz, D. F. (2021). Fig. i

Modern Only

Short Troughed Trident

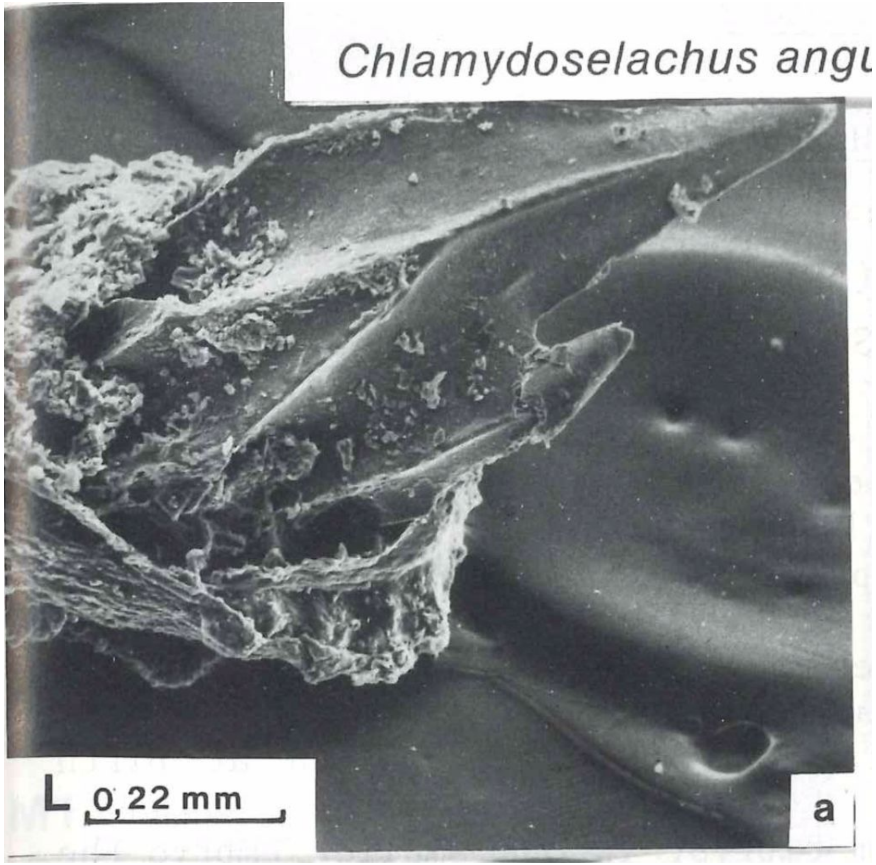

Chlamydoselachus anguineus, Reif, Pg. 103, Fig. L

The short troughed trident type has a pointed spade shape and three (?) cusps. The two side cusps are shorter and are defined by a single ridge and the central cusp is longer and is defined by two ridges which converge to create a central trough.

Taxonomic Citation

Chlamydoselachus anguineus, Reif, Pg. 103, Fig. L

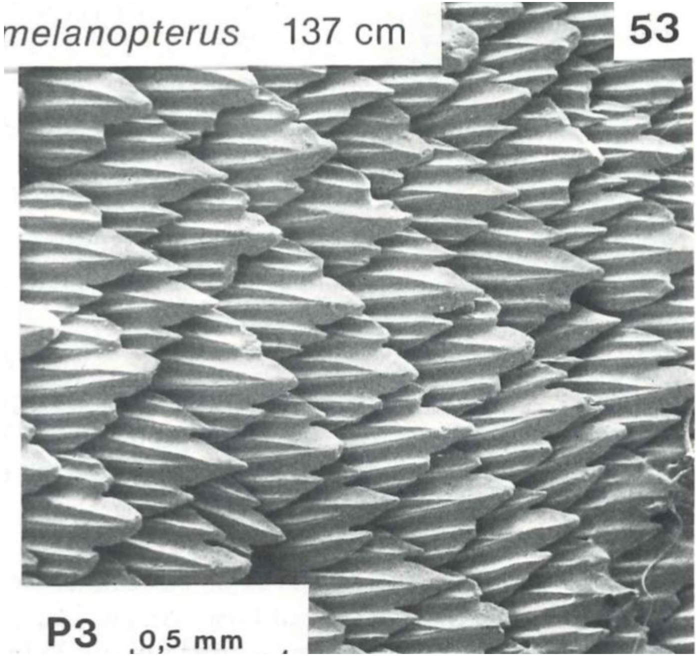

Carcharhinus melanopterus, Reif, Pg. 208, Fig. P3

Six Ridged Troughed Trident

The six ridged troughed trident type has a pointed spade shape with three cusps. Each cusp is composed of two ridges with the central ridge being defined by two converging ridges which create a central trough.

Taxonomic Citation

Carcharhinus melanopterus, Reif, Pg. 208, Fig. P3

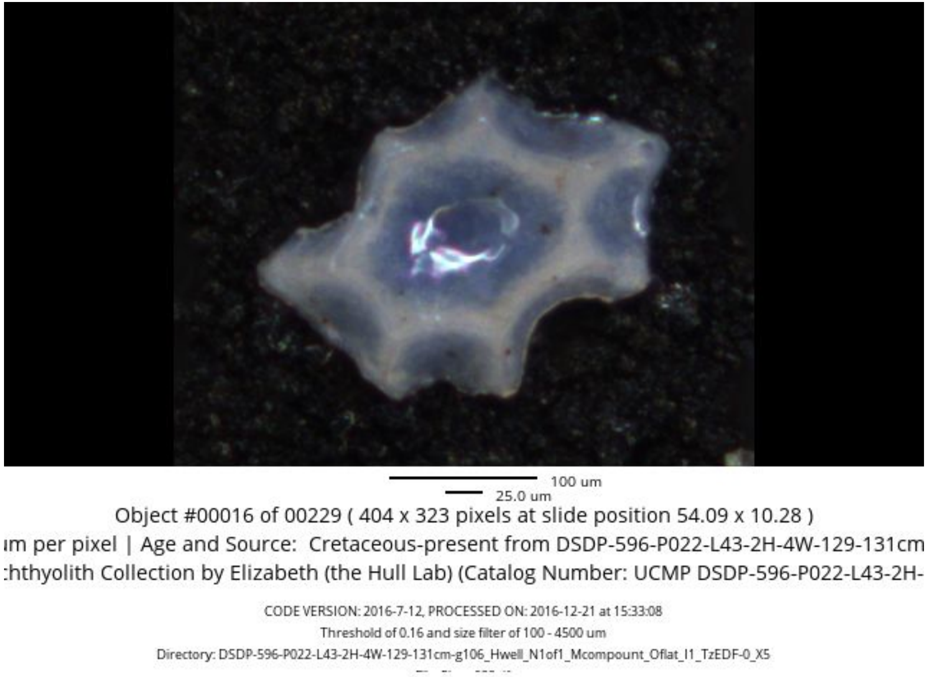

DSDP-596-P022-L43-2H-4W-129-131cm-g106_Hwell_N1of39_obj00016

Single Dimpled Heptagon

The single dimpled heptagon has an irregular shape and is defined by its seven sided central ridge system shape which surrounds a round elongated dimple. Each vertice of the central shape branches out to the edge of the crown.

Taxonomic Citation

Chlamydoselachus anguineus, Reif, Pg. 103, Fig. B1

Etmopterus pusillus, Feichtinger et al. (2021), Fig. F and G

Etmopterus pusillus, Lourtie et al. (2022)

Single Dimpled Quadrilateral

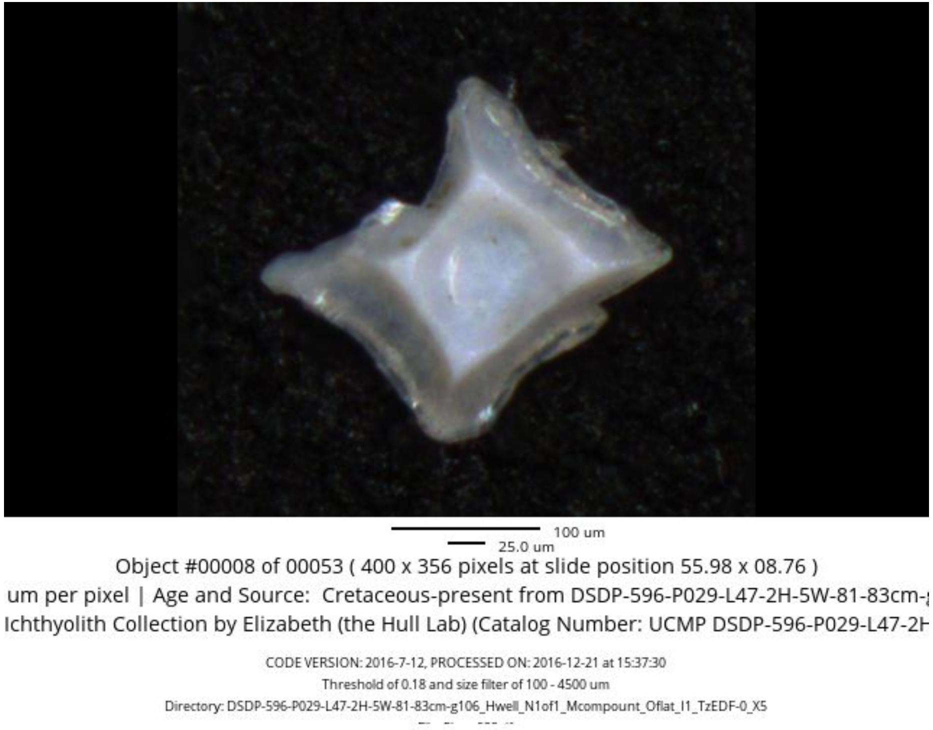

DSDP-596-P029-L47-2H-5W-81-83cm-g106_Hwell_N1of1
_obj00008

The single dimpled quadrilateral has a diamond-like shape and is defined by its four sided central ridge system shape outlining a elongated or circular dimple. The central shape is surrounded by flat crown and each vertice of the central shape has a ridge which branches out to the edge of the crown.

Taxonomic Citation

Isistius plutodus, Castro, Pg. 151

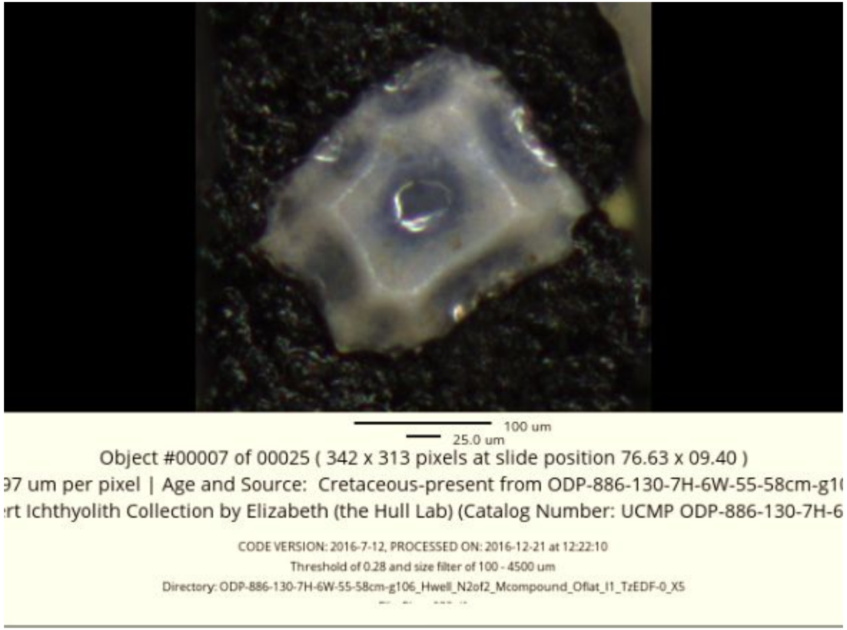

ODP-886-130-7H-6W-55-58cm-g106_Hwell_N2of2_Mcompound_Oflat_I1_TzEDF-0_X5

Single Dimpled Hexagon

The single dimpled hexagon has a diamond-like shape and is defined by its six sided central ridge system shape outlining a elongated round dimple. The central shape is surrounded by flat crown and each vertice of the central shape has one or two ridges which branch out to the edge of the crown.

Taxonomic Citation

Etmopterus pusillus, Feichtinger et al. (2021), Fig. A, C1, and G
Etmopterus bigelowi, Lourtie et al. (2022)

Double Dimpled Hexagon

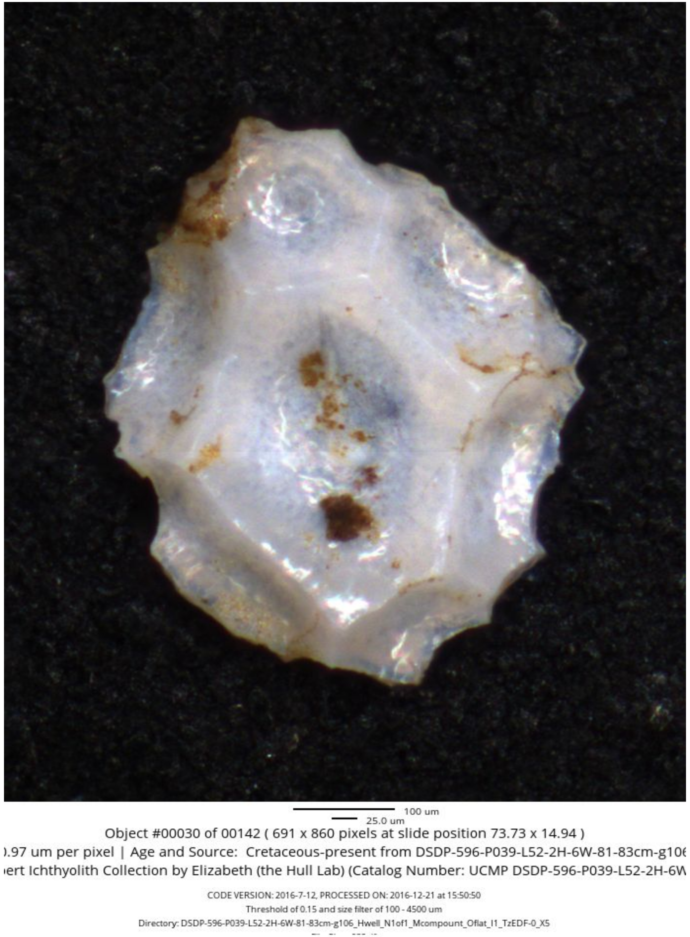

DSDP-596-P039-L52-2H-6W-81-83cm-g106_Hwell_N1of1_obj00030

The double dimpled hexagon has a diamond-like shape and is defined by its six sided central ridge system shape outlining an elongated round dimple. The central shape is surrounded by flat crown and each vertice of the central shape has a ridge which branches out to the edge of the crown. At the end of one side of the crowns axis is a second much smaller central ridge system shape outlining a round dimple.

Single Dimpled Hexagon with Serrated Edges

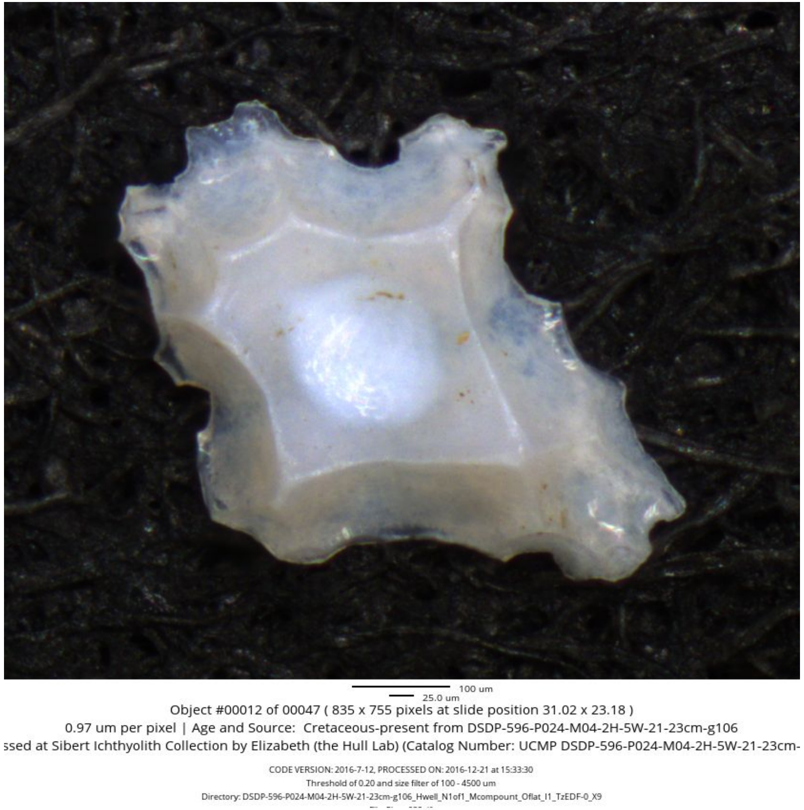

DSDP-596-P024-M04-2H-5W-21-23cm-g106_Hwell_N1of1_obj00012

The single dimpled hexagon with serrated edges has a cruciform shape and is defined by its six sided central ridge system shape outlining a circular dimple and its distinctly serrated edges.

Fossil Only

Single Dimpled Octagon

The single dimpled octagon has a diamond-like shape and is defined by its eight sided central ridge system shape which surrounds a circular dimple. The octagon central shape is composed of ridges of approximately equal length. Most central shape ridges have a shorter ridge which branches off of it and terminates at the edge of the crown.

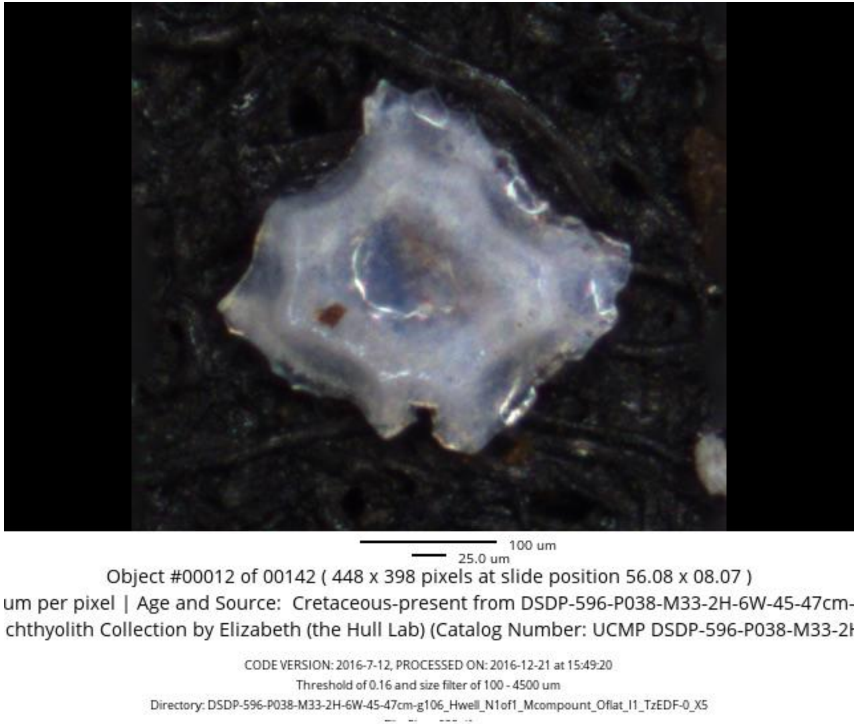

DSDP-596-P038-M33-2H-6W-45-47cm-g106_obj00012

Fossil Only

Single Dimpled Pentagon

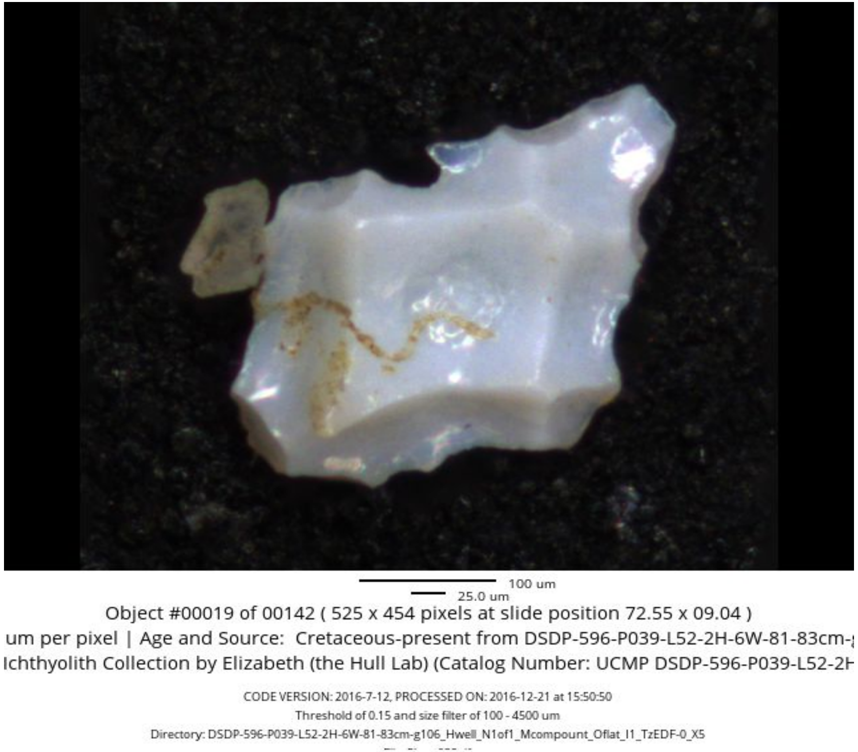

DSDP-596-P039-L52-2H-6W-81-83cm-g106_Hwell_N1of1
_obj00019

The single dimpled pentagon has a diamond-like shape and is defined by its pentagonal central ridge system shape which surrounds a circular dimple.

Taxonomic Citation

Etmopterus pusillus, Feichtinger et al. (2021), Fig. B1 and D
Etmopterus pusillus, Lourtie et al. (2022)

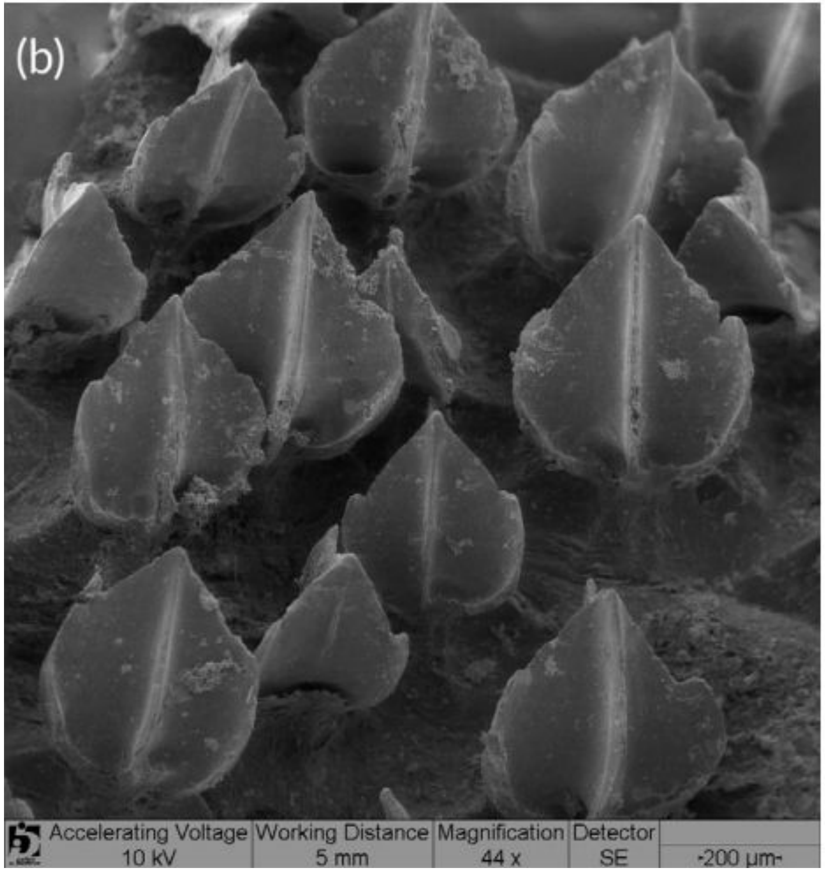

Scymnodon macracanthus, B. Vaz, D. F. (2021), Fig. 15.b

Tall Central Ridged Petal

The tall central ridged petal has a pointed spade shape with a clearly defined central ridge which extends the length of the crown. It has a rounded anterior and a posterior vertex. Shorter curved and less defined side ridges are possible.

Taxonomic Citation

Scymnodon macracanthus, B. Vaz, D. F. (2021), Fig. 15.b
Scymnodon macracanthus, B. Vaz, D. F. (2021), Fig. 14.a
Scymnodon macracanthus, B. Vaz, D. F. (2021), Fig. 15.a
Centrophorus squamosus, Castro, Pg. 76
Pristiophorus schroederi, Castro, Pg. 160
Squalus acanthias, Reif, Pg. 118, Fig. H3

Modern Only

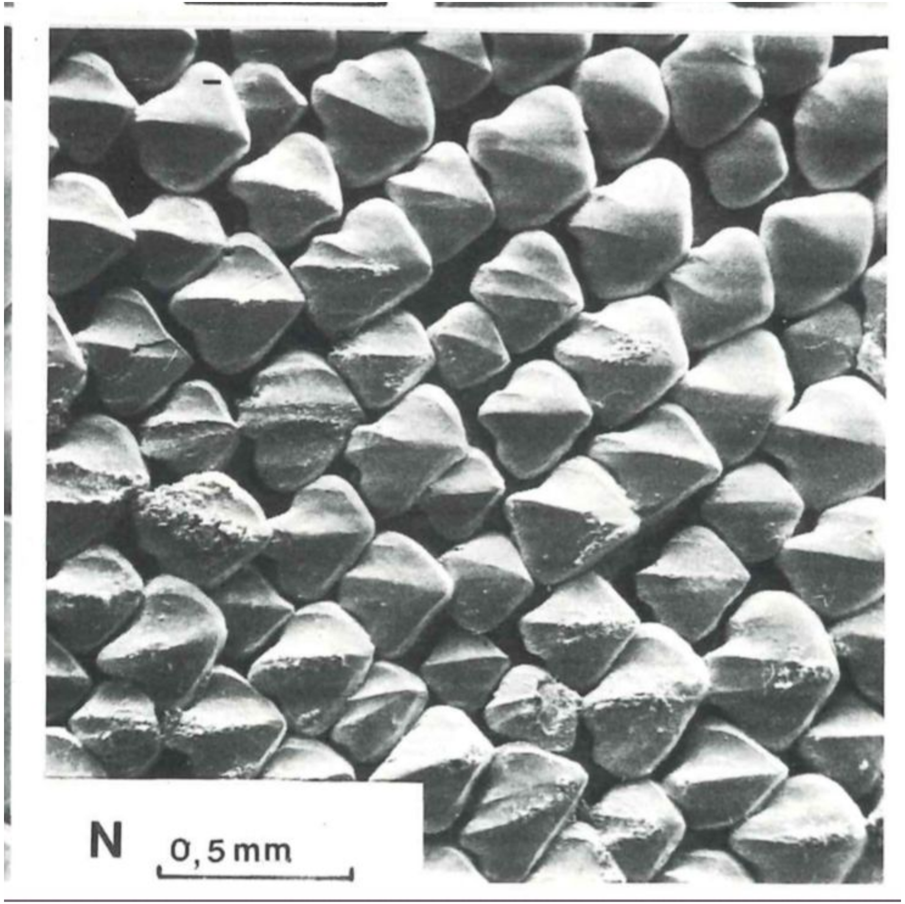

Triakis scyllium, Reif, Pg. 169, Fig. N

Single Ridged Spade

The single ridged spade has a round spade shape with one clearly defined central ridge, shorter and less defined side ridges are possible. It has a posterior with smooth edges which make a vertex.

Taxonomic Citation

Triakis scyllium, Reif, Pg. 169, Fig. N
Nebrius ferrugineus, Reif, Pg. 132, Fig. C1
Odontaspis noronhai, Castro, Pg. 218
Triakis semifasciata, Castro, Pg. 384

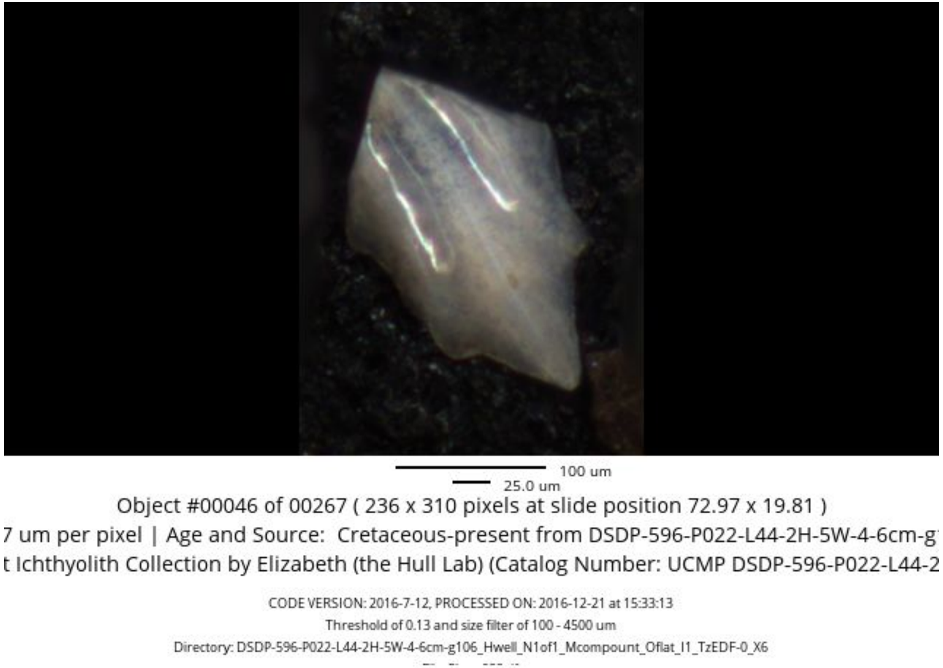

DSDP-596-P022-L44-2H-5W-4-6cm-g106_Hwell_N1of1_obj00046

Skinny Pear Shaped Wedge

The skinny pear shaped wedge has a diamond shape with three ridges. The central ridge extends the length of the crown and has a diamond like shape largest at the anterior and then thinning at the posterior. The two side ridges have triangular points at the posterior.

Taxonomic Citation

Alopias superciliosus, Reif, Pg. 141, Fig. H3
Galeocerdo cuvier, Reif, Pg. 227, Fig. B5

Smooth

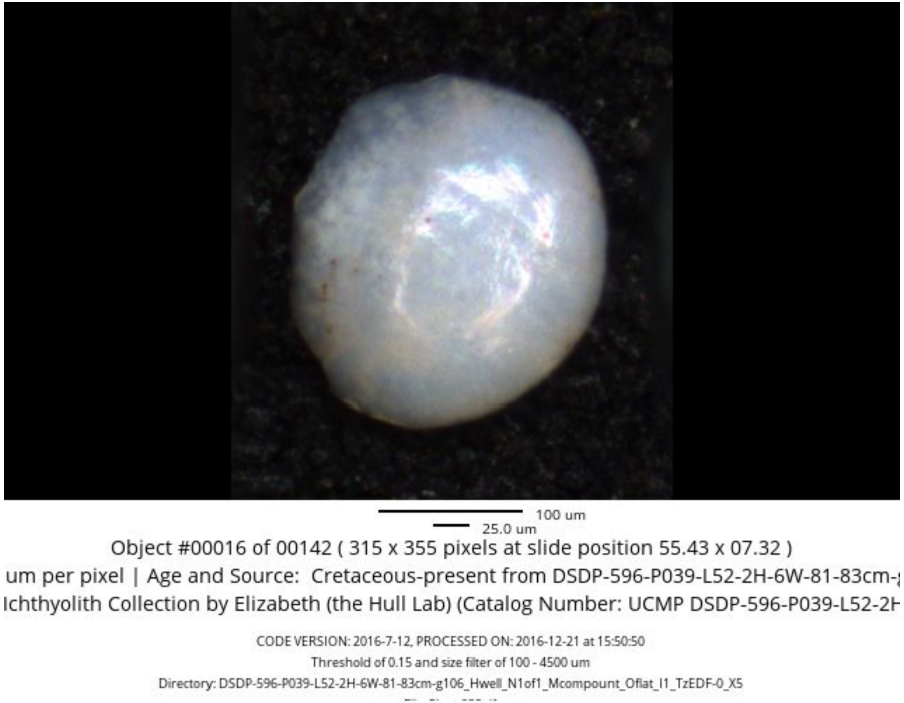

DSDP-596-P039-L52-2H-6W-81-83cm-g106_Hwell_N1of1 _obj00016

The smooth type is a catch all that consists of all smooth denticles that do not fall into a more specific smooth shape category.

Taxonomic Citations

Alopias superciliosus, Reif, Pg. 141, Fig. H1
Carcharhinus falciformis, Reif, Pg. 179, Fig. M2 and M3
C. melanopterus, Reif, Pg. 206, Fig. H1
C. plumbeus, Reif, Pg. 194, Fig. H1
Chiloscyllium plagiosum, Reif, Pg. 130, Fig. G
Dipturus batis, Gravendeel et. al., Pg. 435
Galeocerdo cuvier, Reif, Pg. 229, Fig. H1 and Fig. N
Hexanchus griseus, Reif, Pg. 105, Fig. H1
Leucoraja circularis, Gravendeel et al., Pg. 437
Mustelus canis, Ankhelyi et al. a, Pg. 5, Fig. a
M. mustelus, Reif, Pg. 165, Fig. H1 and Cla
Nebrius ferrugineus, Reif, Pg. 132, Fig. H1
Scyliorhinus canicula, Reif, Pg. 157, Fig. H1
Sphyrna lewini, Reif, Pg. 233, Fig. N
S. tudes, Reif, Pg. 238, Fig. N
S. zygaena, Reif, Pg. 244, Fig. Cla
Squalus acanthias, Reif, Pg. 114, Fig. H1 and Pg. 116, Fig. G and H1
Squatina japonica, Reif, Pg. 128, Fig. P4
S. squatina, Reif, Pg. 126, Fig. H3 and H1
Triaenodon obesus, Reif, Pg. 210, Fig. H1
Triakis scyllium, Reif, Pg. 169, Fig. H1

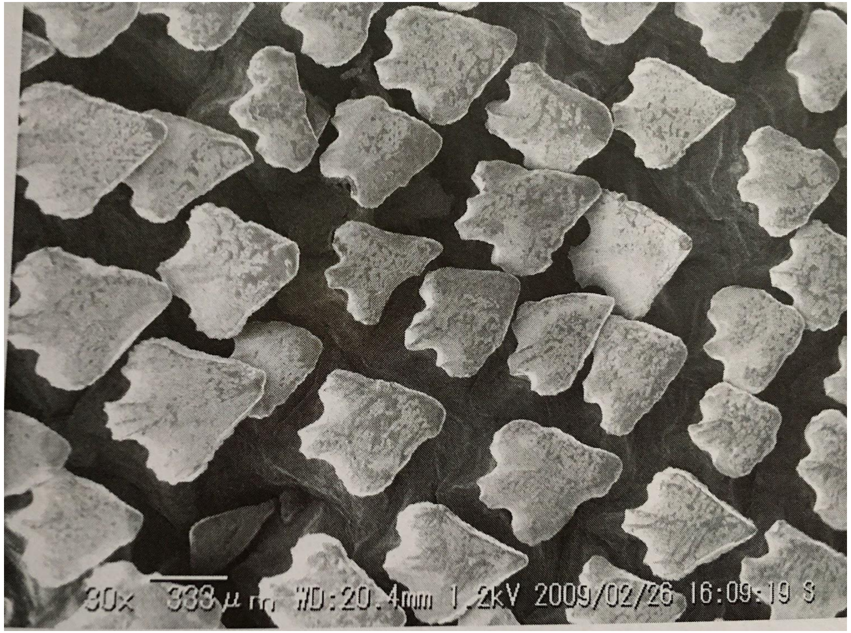

Cephaloscyllium ventriosum, Castro, Pg. 318

Smooth Arrow

The smooth arrow type has an arrow shape with no ridges.

Taxonomic Citation

Cephaloscyllium ventriosum, Castro, Pg. 318
Mustelus canis, Ankhelyi et al., Pg. 40, Fig. C
Squalus acanthias, Reif, Pg. 118, Fig. Cla
Triakis scyllium, Reif, Pg. 169, Fig. H1
Galeorhinus galeus, Reif, Pg. 171, Fig. M3
Carcharhinus falciformis, Reif, Pg. 179, Fig. M1
Carcharhinus galapagensis, Reif, Pg. 183, Fig. M3
Carcharhinus melanopterus, Reif, Pg. 206, Fig. M3
Triaenodon obesus, Reif, Pg. 212, Fig. M1
Prionace glauca, Reif, Pg. 220, Fig. M3
Prionace glauca, Reif, Pg. 221, Fig. P3
Galeocerdo cuvier, Reif, Pg. 229, Fig. G
Galeocerdo cuvier, Reif, Pg. 229, Fig. M3
Sphyrna zygaena, Reif, Pg. 244, Fig. M3
Scymnodon macracanthus, B. Vaz, D. F. (2021), Fig. 14.c

Modern Only

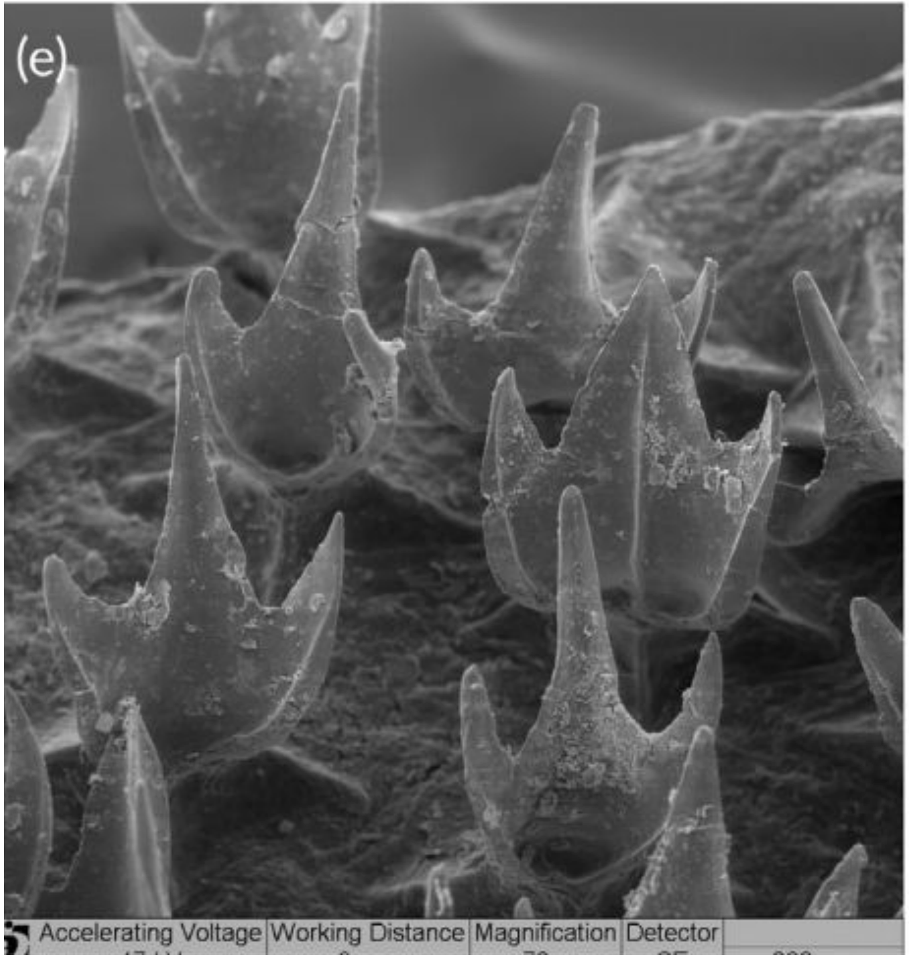

Scymnodon macracanthus, B. Vaz, D. F. (2021), Fig. 14.e

Two Ridged Trident

The Two Ridged Trident type has a pointed spade shape and three cusps, the central cusp is ~2 times the length of the side cusps and smooth while the side cusps are defined by shorter curved ridges. The crown is nearly perpendicular to the skin and the crowns side ridges are raised in a way that creates a concave cup (or thumbprint depression) at the posterior edge of the crown.

Taxonomic Citations

Scymnodon macracanthus, B. Vaz, D. F. (2021), Fig. 14.e
Scymnodon macracanthus, B. Vaz, D. F. (2021), Fig. 14.d
Scymnodon macracanthus, B. Vaz, D. F. (2021), Fig. 14.f

Modern Only

Smooth Nubbed

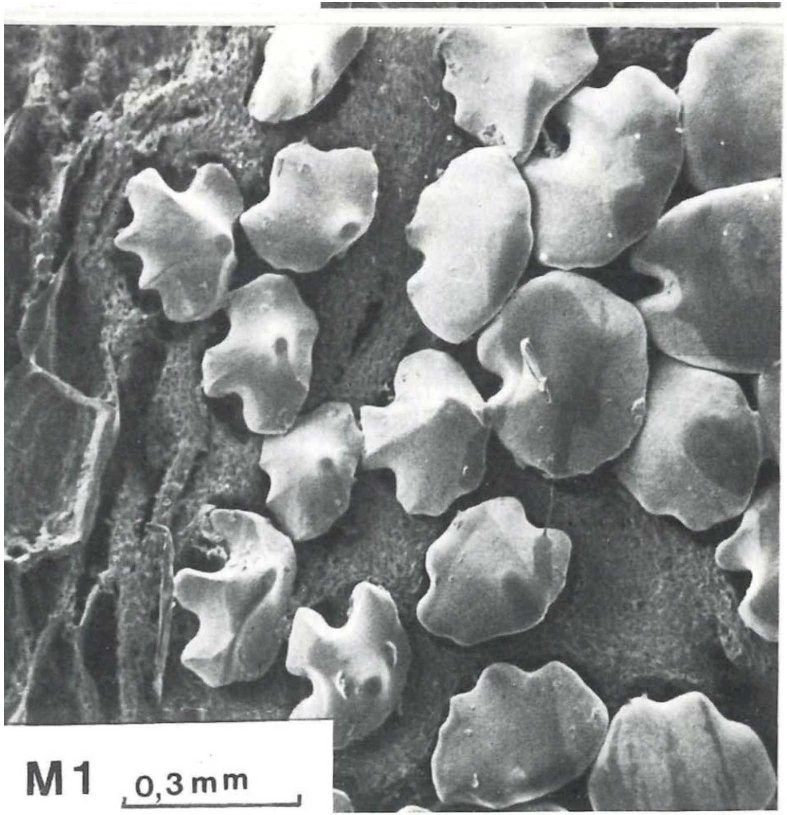

Chiloscyllium plagiosum, Reif, Pg. 130, Fig. M1

The smooth nubbed type has an irregular shape and no ridges. It is characterized by a vertical peak or "nub" on one edge of the denticle.

Taxonomic Citations

Chiloscyllium plagiosum, Reif, Pg. 130, Fig. M1
Chiloscyllium punctatum, Reif, Pg. 132, Fig. M1

Modern Only

Smooth Petal

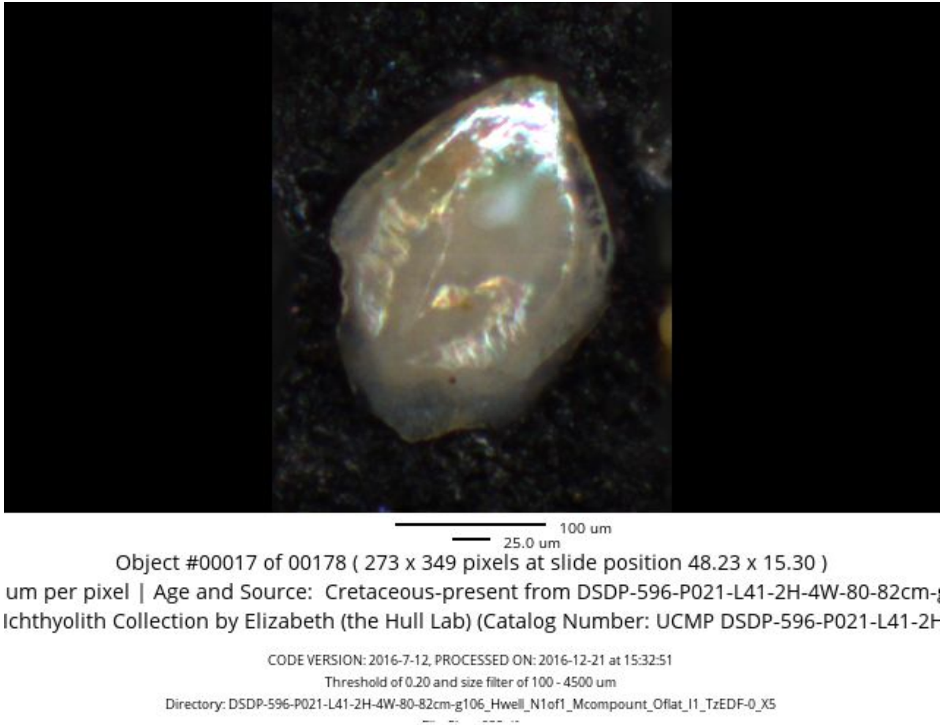

DSDP-596-P021-L41-2H-4W-80-82cm-g106_Hwell_N1of1 _obj00017

The smooth petal type has a stretched spade shape with no ridges.

Taxonomic Citation

Centroscymnus coelolepis, Castro, Pg. 117
C. owstoni, Castro , Pg. 120
Chiloscyllium plagiosum, Reif, Pg. 130, Fig. B3 and C3
C. punctatum, Reif, Pg. 132, Fig. B3 and M1
Galeus melastomus, Reif, Pg. 153, Fig. B6 and Pg. 155, Fig. Cla
Isurus oxyrinchus, Reif, Pg. 145, Fig. M1 and Pg. 147, Fig. M1
Mustelus californicus, Castro, Pg. 366
M. canis, Ankhelyi et al., Pg. 5, Fig. h
M. mustelus, Reif, Pg 165, Fig. B3 and Pg. 167, Fig. H3
Nebrius ferrugineus, Reif, Pg. 132, Fig. H3
Negaprion brevirostris, Reif, Pg. 225, Fig. P6
Raja microocellata, Gravendeel et al., Pg. 433, Fig. 11 and 12
Scyliorhinus canicula, Reif, Pg. 157, Fig. H1
S. canicula, Reif, Pg. 159, Fig. B3 and P5
S. stellaris, Reif, Pg. 161, Fig. Cla
Squatina californica, Castro, Pg. 163
S. dumeril, Castro, Pg. 167
S. heteroptera, Castro, Pg. 170
S. mexicana, Castro, Pg. 172
Triakis scyllium, Reif, Pg. 169, Fig. H3

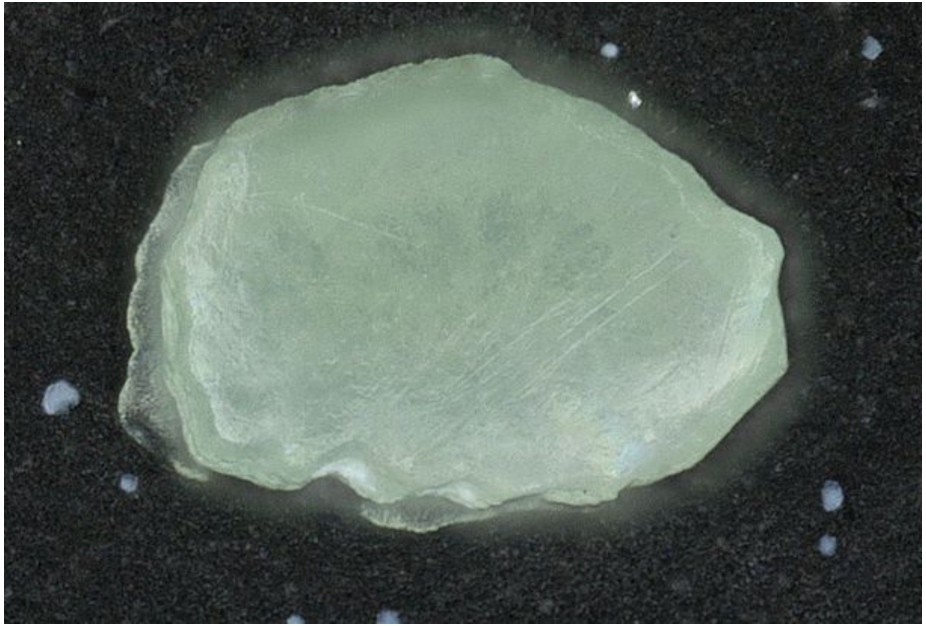

Rhizoprionodon terraenovae, MCZ S-432

Smooth Scalloped

The smooth scalloped type has a round oval like shape. The crown is characterized by a scalloped texture on at least half of the crown which may or may not associated with with very short shallow ridges which do not extend more than ⅛ the length of the crown. These denticles are often found on the snout.

Taxonomic Citation

Rhizoprionodon terraenovae, MCZ S-432
Hemiscyllium ocellatum , MCZ 155795
Chiloscyllium plagiosum. Reif, Pg. 130, Fig. H2
Isurus oxyrinchus, Reif, Pg. 145, Fig. H1
Lamna nasus, Reif, Pg. 149, Fig. Cla
Galeorhinus galeus, Reif, Pg. 171, Fig. H1
Carcharhinus galapagensis, Reif, Pg. 181, Fig. H1
Carcharhinus plumbeus, Reif, Pg. 194, Fig. H1
Prionace glauca, Reif, Pg. 214, Fig. H1
Galeocerdo cuvier, Reif, Pg. 231, Fig. H3
Sphyrna zygaena, Reif, Fig. N
Triakis semifasciata, Gabler-Smith, M. K., Wainwright, D. K., Wong, G. A., & Lauder, G. V. (2021), Fig. F

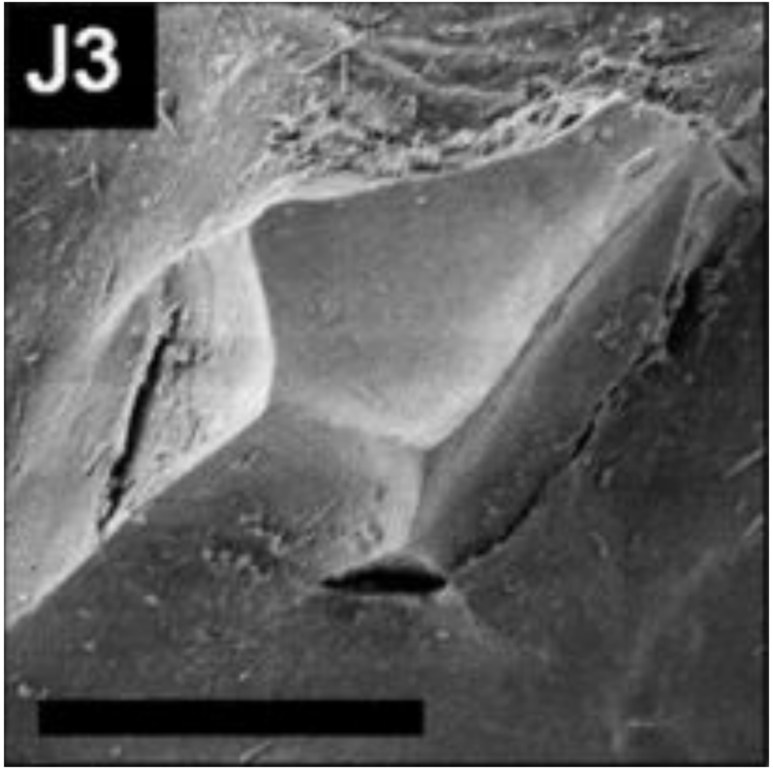

Etmopterus pusillus, Feichtinger et al. (2021), Fig. J3

Spear

The spear type has a stretched spade shape with a pentagonal central ridge system shape. The crown is longer on one axis than the other. On the longer axis there is an extended tail-like ridge which connects to two ridges which make up the wider angle of the central ridge system shape. From these two ridges two longer side ridges stretch the length of the crown converging at a fifth ridge at the opposite edge.

Taxonomic Citation

Etmopterus pusillus, Feichtinger et al. (2021), Fig. J3

Modern Only

Square Kite with Nodules

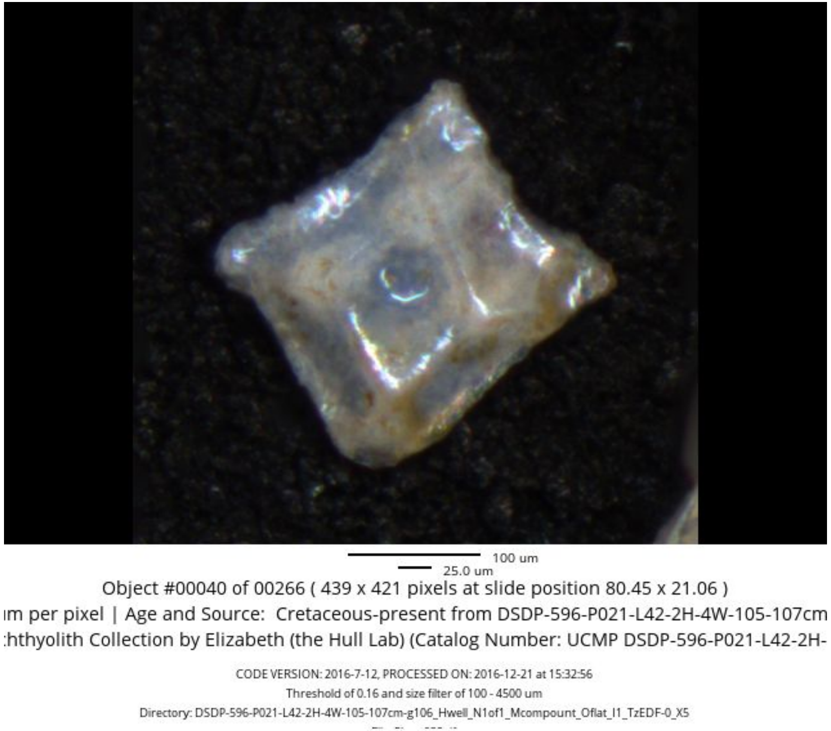

DSDP-596-P021-L42-2H-4W-105-107cm-g106_Hwell_N1of1_obj00040

The square kite with nodules type has a square shaped crown and a diamond central ridge system shape which encapsulates a circular dimple in the center. On one axis the vertices of the central ridge system shape have two branching ridges which extend to the overall denticle shape. On the other axis the central shape vertices each have a singular ridge which extends to the overall shape. Where the ridges intersect they become more pronounced and are described as “nodules”.

Fossil Only

Squished Kite

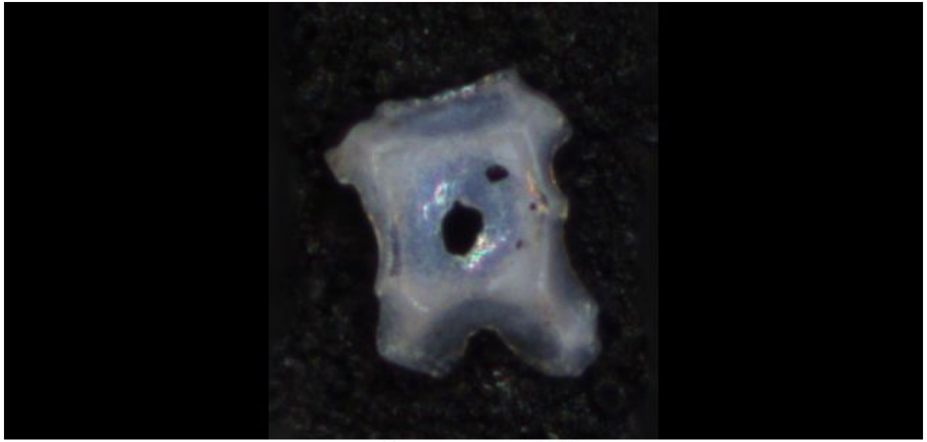

100 um
25.0 um
Object #00033 of 00239 (271 x 296 pixels at slide position 52.66 x 17.10)
um per pixel | Age and Source: Cretaceous-present from DSDP-596-P020-L39-2H-4W-31-33cm-
Ichthyolith Collection by Elizabeth (the Hull Lab) (Catalog Number: UCMP DSDP-596-P020-L39-2H

CODE VERSION: 2016-7-12, PROCESSED ON: 2016-12-21 at 15:32:37
Threshold of 0.12 and size filter of 100 - 4500 um
Directory: DSDP-596-P020-L39-2H-4W-31-33cm-g106_Hwell_N1of1_Mcompount_Oflat_I1_TzEDF-0_X5

DSDP-596-P020-L39-2H-4W-31-33cm-g106_Hwell_N1of1_obj00033

The squished kite type has a square-like shape with a square-like central ridge system shape defined by four thin ridges. Each vertice of the central shape has a shorter ridge which extends to the overall crown vertices. At the center of the central ridge system shape is one circular shaped dimple.

Fossil Only

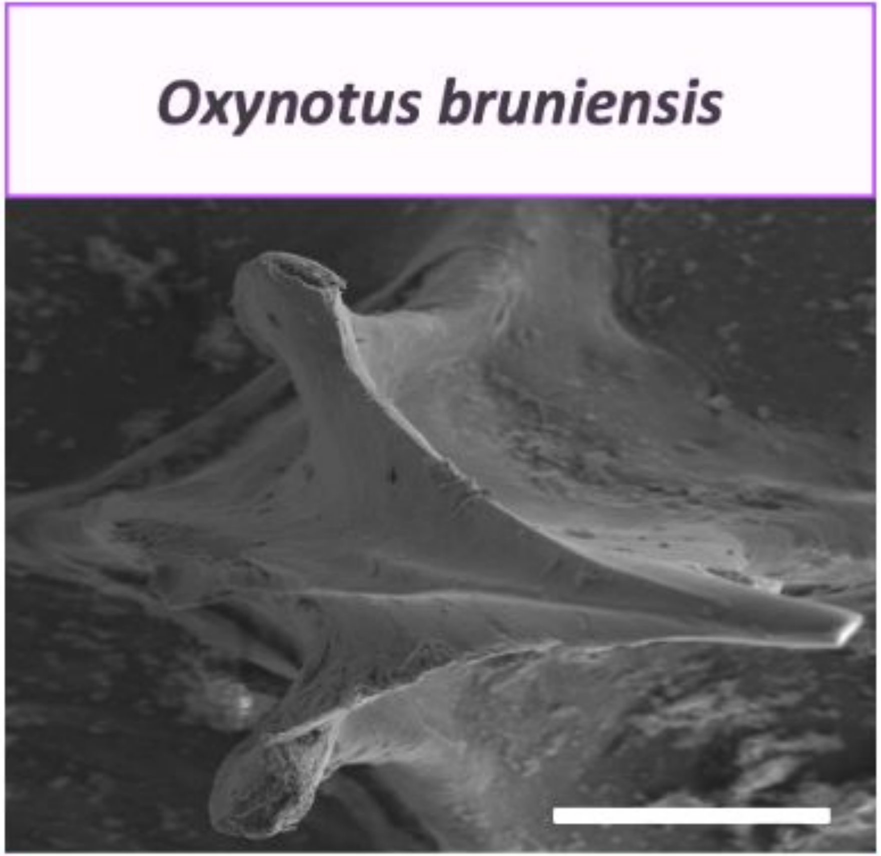

Oxynotus bruniensis, Lourtie et al. 2022

Stretched Airplane

The stretched airplane type has a triangular shape with a wide anterior edge and sides which stretch to a pointed posterior. This type has one central ridge and two r/curved ridge outgrowths on either side at the anterior.

Taxonomic Citations
Oxynotus bruniensis, Lourtie et al. 2022

Modern Only

Stretched Bilateral Central Ridged

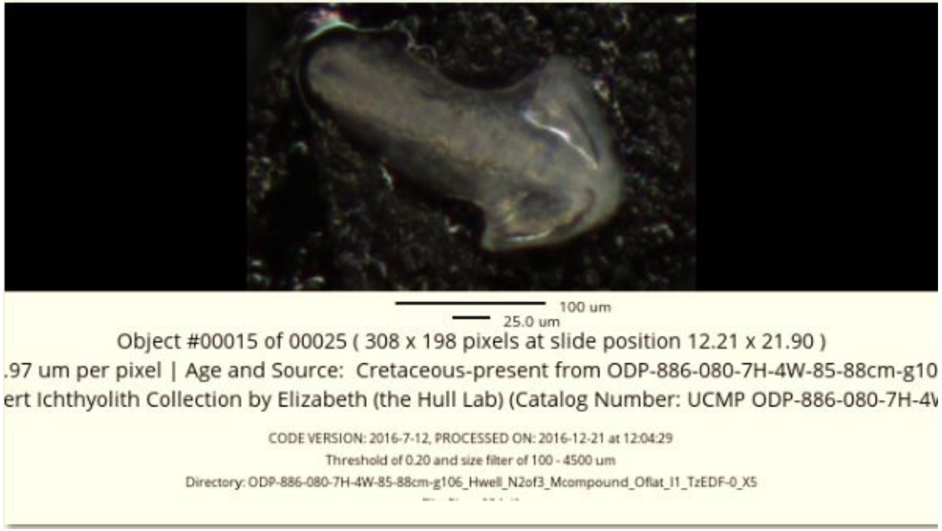

ODP-886-080-7H-4W-85-88cm-g106_obj00015_edf

The stretched bilateral ridged type has a stretched spade shape and is longer along the anterior/posterior axis than wide. This type is characterized by a long central ridge which defines ~ ⅔ of the crown and has two shorter side ridges which diverge from the anterior and curve up towards the posterior of the crown.

Fossil Only

Stretched Branching Crown

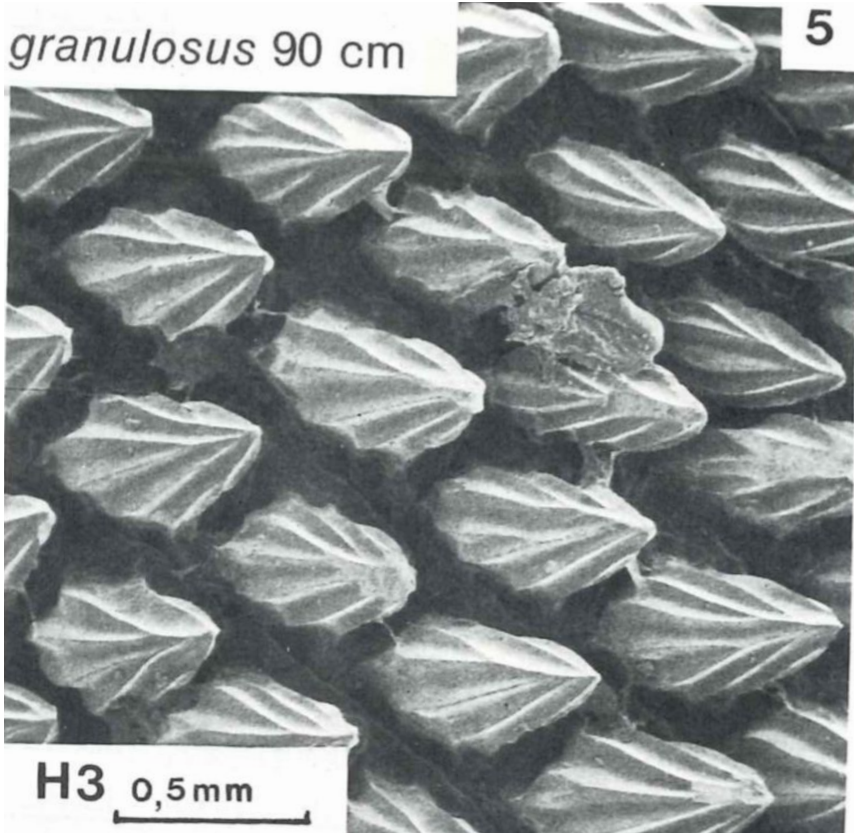

Centrophorus granulosus, Reif, Pg. 112, Fig. H3

The stretched branching crown type has an oval shape with more than 6 ridges which branch out from the anterior vertex in a pinnate arrangement on either side of a straight central ridge which extends ~½ the length of the crown.

Taxonomic Citation

Centrophorus granulosus, Reif, Pg. 112, Fig. H3

Stretched Minnie Indented Shell

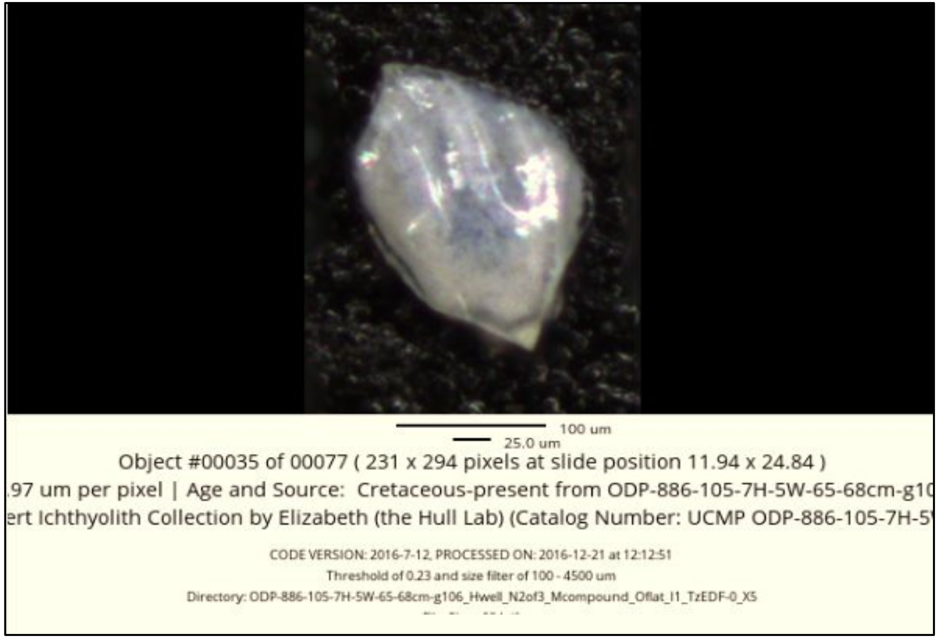

ODP-886-105-7H-5W-65-68cm-g106_obj00035_edf

The stretched minnie indented shell has a stretched spade shape with shallow linear ridges which begin ~ ½ down the length of the crown and extend to the rounded posterior.

Taxonomic Citation

Mustelus albipinnis, Castro, Pg. 364
Mustelus canis, Ankhelyi et al., Pg. 5, Fig. e
Mustelus canis, Ankhelyi et al., Pg. 5, Fig. f
Mustelus canis, Ankhelyi et al., Pg. 5, Fig. g
Mustelus canis, Ankhelyi et al., Pg. 5, Fig. i
Mustelus canis, Ankhelyi et al., Pg. 39
Prionace glauca, Reif, Pg. 217, Fig. P3
Negaprion brevirostris, Reif, Pg. 225, Fig. P6
Centroscymnus coelolepis, Weigmann et al., (2016), Fig. C

Stretched Two Dimpled Spade

The stretched two dimpled spade has a stretched shape with a round anterior or posterior and a pointed anterior or posterior shape. The crown is bisected by a central ridge and has four side ridges (two on either side) which branch out from the center. Between the two side ridges and the central ridge on either side is a dimple.

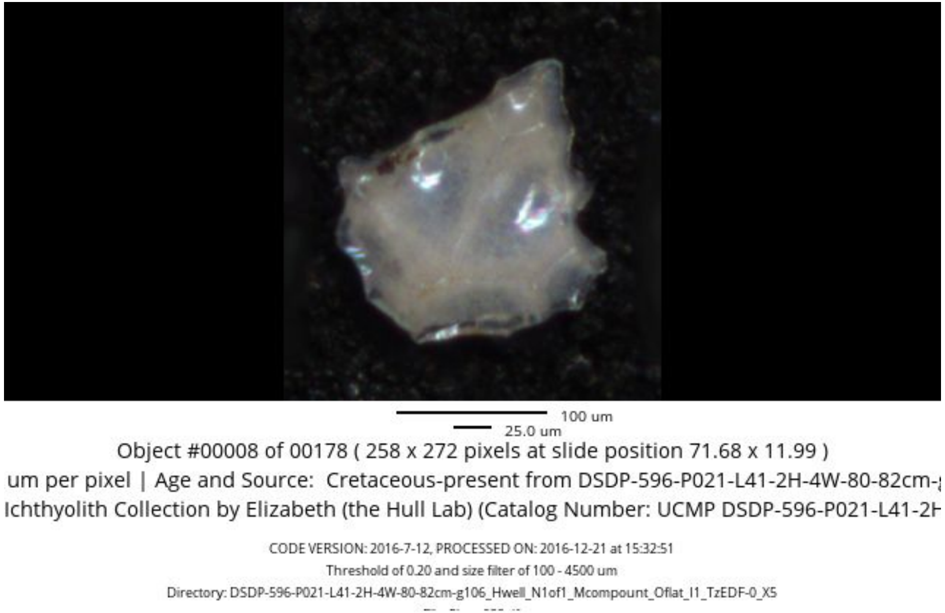

DSDP-596-P021-L41-2H-4W-80-82cm-g106_Hwell_N1of1_obj00008

Fossil Only

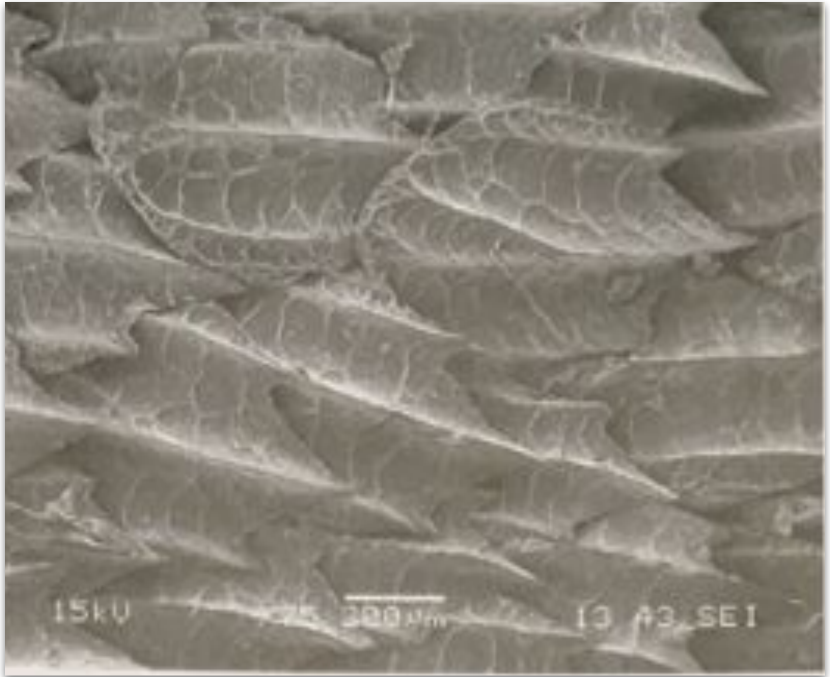

Zameus squamulosus, Castro, Pg. 122

Textured Trident

The textured trident type has a pointed spade shape with three cusps which are defined by ridges. This type is characterized by a honeycomb or wavy surface texture which covers the crown.

Taxonomic Citations

Zameus squamulosus, Castro, Pg. 122
Apristurus canutus, Castro, Pg. 299
Apristurus kampae, Castro, Pg. 301
Apristurus laurussonii, Castro, Pg. 303
Apristurus melanoasper, Castro, Pg. 307
Galeus melastomus, Reif, Pg. 153, Fig. H2
Galeus melastomus, Reif, Pg. 153, Fig. B4
Zameus squamulosus, Lourtie et al. 2022

Modern Only

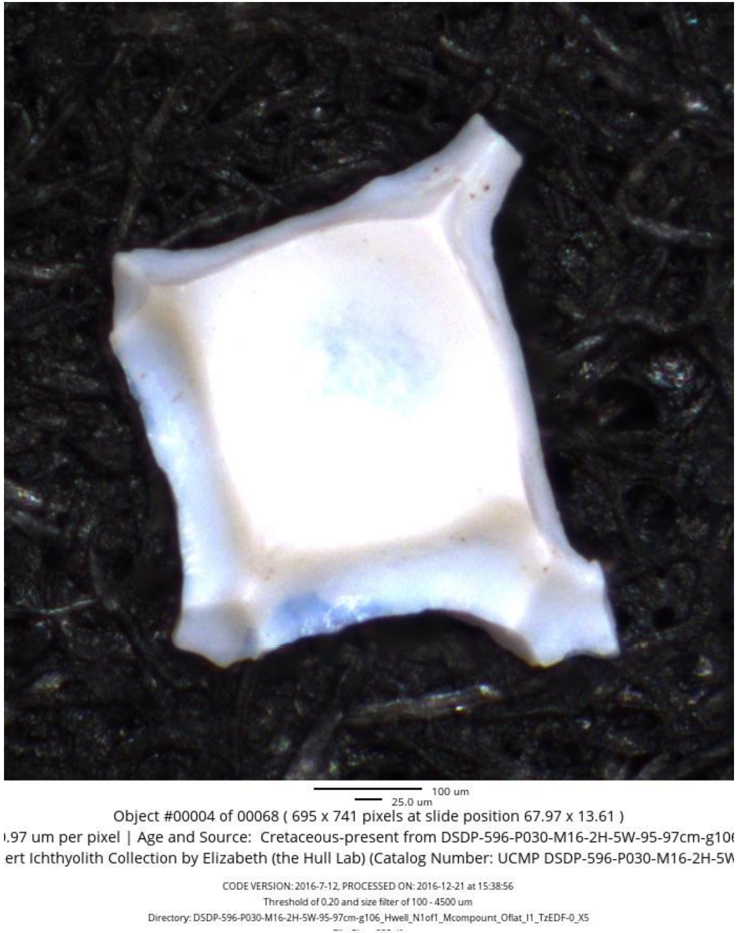

DSDP-596-P030-M16-2H-5W-95-97cm-g106_obj00004

Thin Ridged Kite

The thin ridged kite type has a square shape and has a square central shape defined by 4 thin and clearly defined ridges which encapsulate a central circular dimple. Each vertice of the central shape has one shorter ridge which branches off at ~45 degrees. The edges of the shape are outlined by a thin flat area of crown.

Taxonomic Citation

Etmopterus pusillus, Feichtinger et al. (2021), Fig. B2

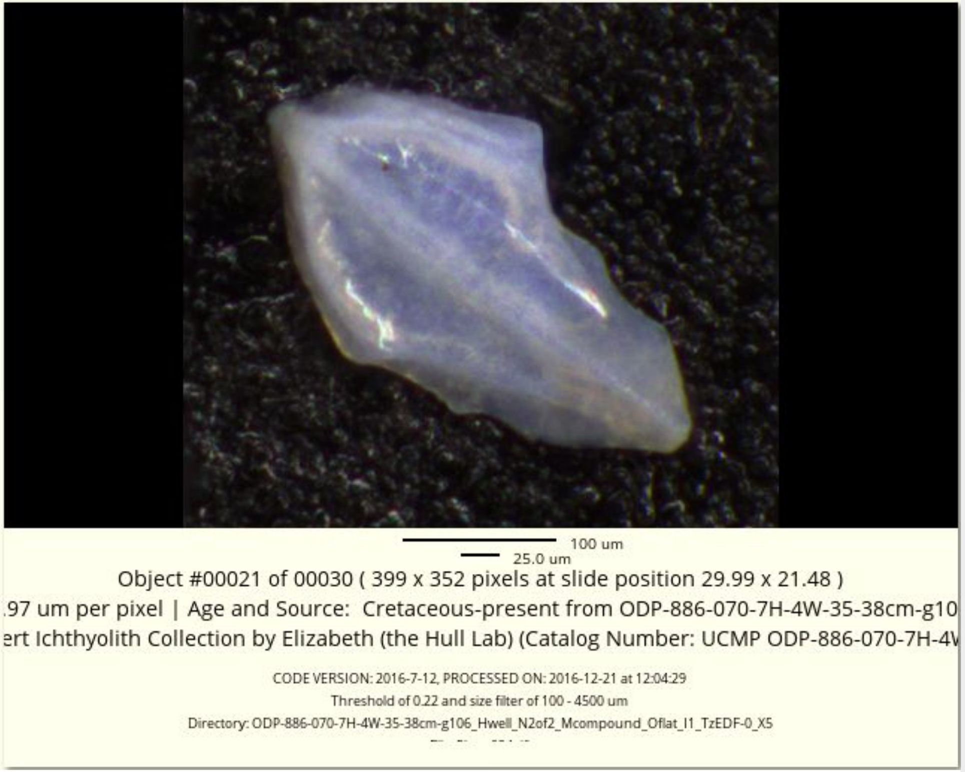

ODP-886-070-7H-4W-35-38cm-g106_obj00021_edf

Thin Ridged Trident

The thin ridged trident has a stretched spade shape with three thin ridges which define the length of the crown. The central ridge is straight and bisects the crown and the two side ridges are meandering and diverge from the anterior, curving out to the center of the crown and then converging at the posterior.

Taxonomic Citations

Centrophorus granulosus, Reif, Pg. 112, Image M3

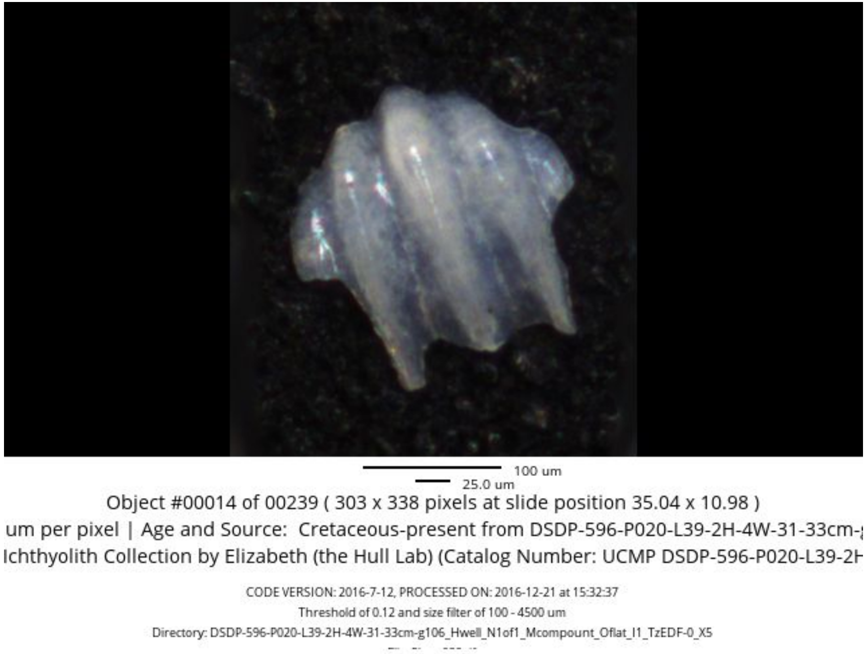

DSDP-596-P020-L39-2H-4W-31-33cm-g106_Hwell_N1of1 _obj00014

Three Ridged Linear Fan with Ridge Outgrowths

The three ridged linear fan with ridge outgrowths has a squared spade shape with three ridges which are equal in width and length and ridge “outgrowths” at either side edge of the crown which are are much shorter than the ridges but the same width.

Taxonomic Citation

Carcharhinus amblyrhynchos, Reif, Pg. 199, Fig. B3
C. brachyurus, Castro, Pg. 403
C. falciformis, Reif, Pg. 177, Fig. B3
C. galapagensis, Reif, Pg. 181 H3, B3 and Pg. 183, Fig. P3
C. isodon, Castro, Pg. 421
C. leucas, Castro, Pg. 426
C. melanopterus, Reif, Pg. 206, Fig. B2
C. obscurus, Reif, Pg. 185, Fig. C1 and C3
C. plumbeus, Reif, Pg. 192, Fig. C1, Pg. 194, Fig. C1, and Pg. 196, Fig. H2, H3, B3, B4, B6, C1, and 197, Fig. C3, P1, and N
Isurus oxyrinchus, Reif, Pg. 147, Fig. H2
Lamna nasus, Reif, Pg. 149, Fig. H1, H2, H3, P1, and P3
Negaprion brevirostris, Castro, Pg. 481
N. brevirostris, Reif, Pg. 224, Fig. B6 and Pg. 225, Fig. P1
Sphyrna media, Castro, Pg. 514
S. mokarran, Castro, Pg. 516
S. tudes, Castro, Pg. 525
S. tudes, Reif, Pg. 237, Fig. H2 and Pg. 238, Fig. C2, C3, and M3
S. zygaena, Reif, Pg. 240, Fig. B1, B3, H2, and P1
Triaenodon obesus, Reif, Pg. 212, Fig. B1

Three Ridged Petal

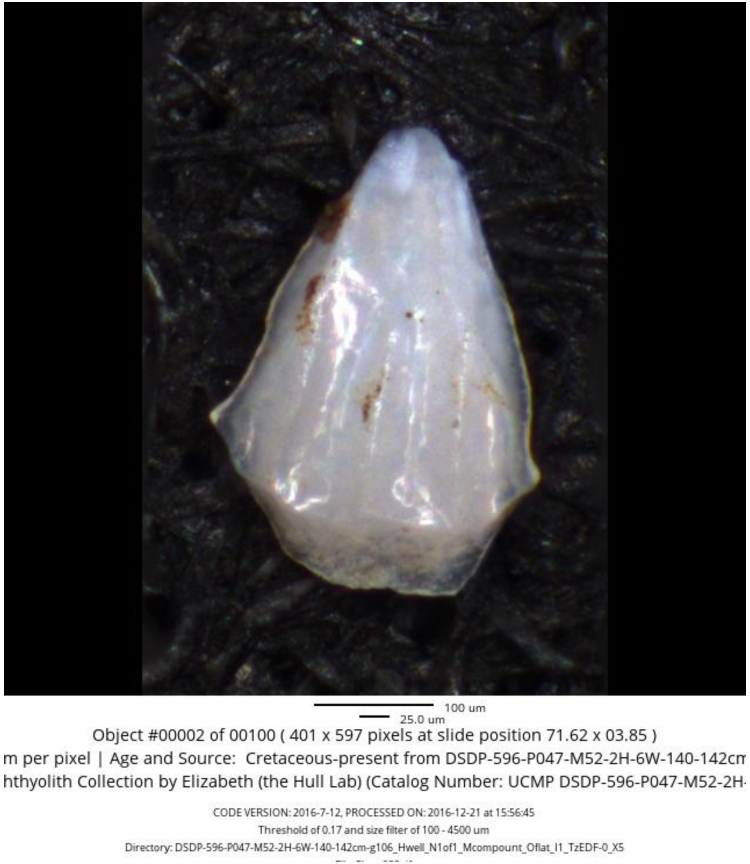

DSDP-596-P047-M52-2H-6W-140-142cm-g106_obj00002

The three ridged petal type has a stretched spade shape with a wide rounded anterior which tapers to a smooth posterior vertex. This type can have three to five linear ridges converging at the posterior.

Taxonomic Citations

Mustelus dorsalis, Castro, Pg. 372
Scyliorhinus meadi, Castro, Pg. 340
Scyliorhinus retifer, Reif, Pg. 163, Fig. H3
Triaenodon obesus, Reif, Pg. 212, Fig. C2
Scyliorhinus hesperius, Castro, Pg. 338

Thumbprint disc

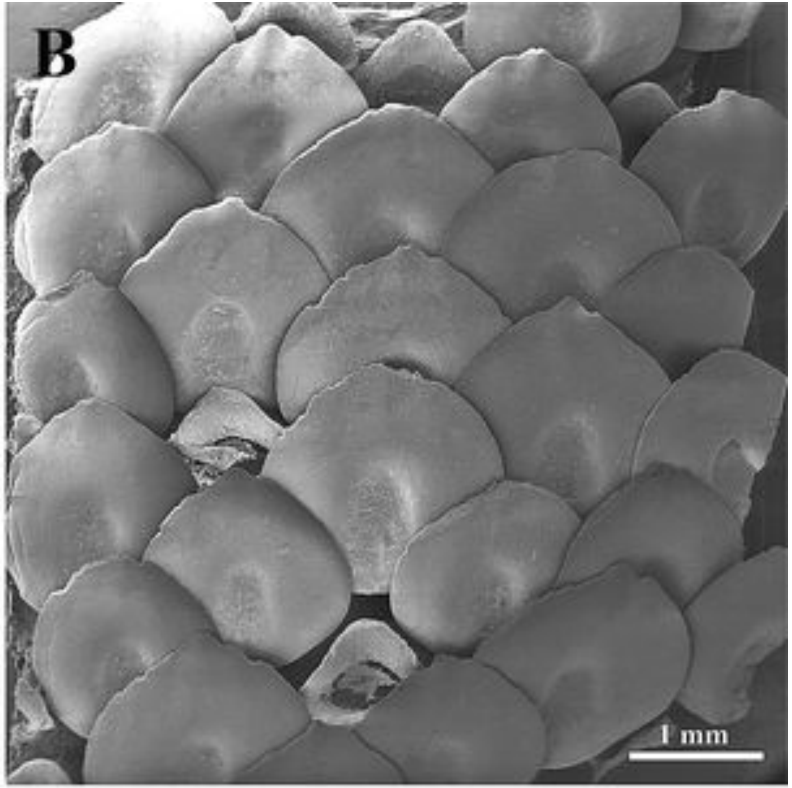

Centroscymnus owstonii, Weigmann et al. (2016), Fig. B

The thumbprint disc type has a circular shape with a slightly tapered posterior. The type is characterized by having no ridges and a oval shaped depression near the anterior of the crown.

Taxonomic Citations

Centroscymnus coelolepis, Castro, Pg. 117
Centroscymnus owstoni, Castro, Pg. 120
Centroscymnus owstonii, Weigmann et al. (2016), Fig. B
Centroscymnus coelolepis, Weigmann et al. (2016), Fig. D
Centroscymnus owstonii, Vaz et al. (2021), Fig. d
Centroscymnus coelolepis, Vaz et al. (2021), Fig. e

Modern Only

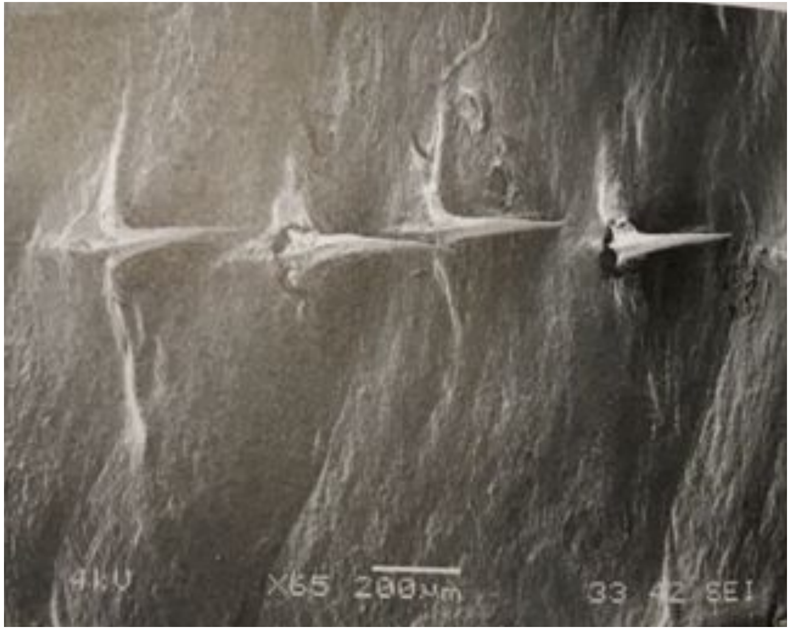

Etmopterus bullisi, Castro, Pg. 101

Triangular Spines

The triangular spine type has a long vertical triangular projection and four ridges arranged at 90 degree angles to each other surrounding the base of the crown which run to ~⅓ the height of the crown.

Taxonomic Citations

Etmopterus bullisi, Castro, Pg. 101
Etmopterus gracilispinis, Castro, Pg. 103
Etmopterus princeps, Castro, Pg. 107
Etmopterus schultzi, Castro, Pg. 110
Alopias superciliosus, Castro , Pg. 236
Cetorhinus maximus, Castro, Pg. 249
Parmaturus xaniurus, Castro, Pg. 332
Raja brachyura, Gravendeel et al. Fig. 6 (cross-type prickle)
Raja brachyura, Gravendeel et al., Fig. 7 (star type)
Raja brachyura, Gravendeel et al., Fig. 7 (leaf type)
Raja clavata, Gravendeel et al., Fig. 10 (myrmecoid lead type prickle)
Raja microocellata, Gravendeel et al., Fig. 12 (leaf type prickle)

Modern Only

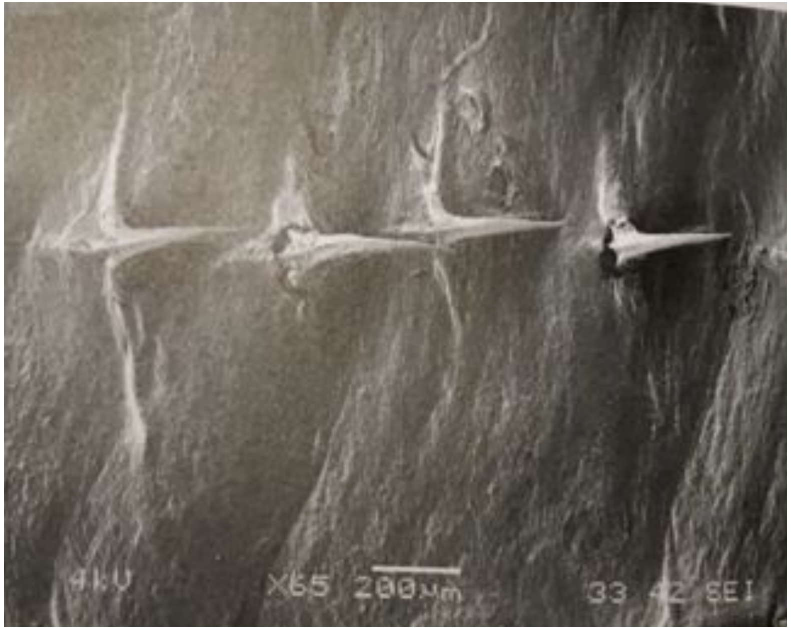

Etmopterus bullisi, Castro, Pg. 101

Triangular Spines

The triangular spine type has a long vertical triangular projection and four ridges arranged at 90 degree angles to each other surrounding the base of the crown which run to ~⅓ the height of the crown.

Taxonomic Citations

Amblyraja radiata, Gravendeel et al., Fig. 26 (star type prickle)
Squalus acanthias, Reif, Pg. 114, Fig. C2
Nebrius ferrugineus, Reif, Pg. 132, Fig. M1
Galeocerdo cuvier, Reif, Pg. 227, Fig. B3
Etmopterus spinax, Lourtie et al. 2022
Etmopterus polli, Lourtie et al. 2022
Etmopterus brachyurus, Lourtie et al. 2022

Modern Only

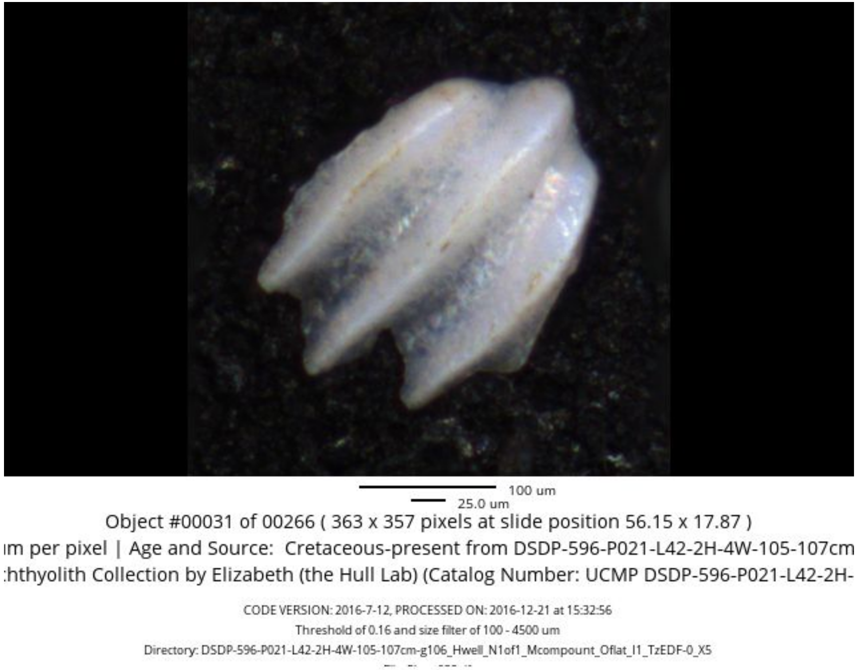

DSDP-596-P021-L42-2H-4W-105-107cm-g106_Hwell_N1of1_obj00031

Trident

The trident type has a pointed spade shape with an anterior vertex and three identical cusps defined by ridges which make up the pointed posterior. The side ridges define the edges of the crown. The ridges are similar in shape but the central ridge is longer than the side ridges.

Taxonomic Citations

Alopias pelagicus, Castro, Pg. 233
A. superciliosus, Reif, Pg. 141, Fig. H2 and C1 and Pg. 143, Fig. P3 and M3
A. vulpinus, Reif, Pg. 139, Fig. C2, C3, and P3
Carcharhinus amblyrhynchos, Reif, Pg. 200, Fig. C3
C. falciformis, Reif, Pg. 173, Fig. H1, H2, and H3, Pg. 173, Fig. B4, and Pg. 175, Fig. B6, C1, C2, and C3
C. melanopterus, Reif, Pg. 204, Fig. B2, H2, H3, and B3, and Pg. 206, Fig. M3
C. obscurus, Reif, Pg. 185, Fig. C1, C3, and P1
C. plumbeus, Reif, Pg. 189, Fig. H2, H3, and B3, Pg. 190, Fig. B6 and P3, and Pg. 192, Fig. B2, B4, M1, B5, and P1
C. porosus, Castro, Pg. 459
C. signatus, Castro, Pg. 463
Carcharodon carcharias, Castro, Pg. 258
Carcharodon carcharias, Reif, Pg. 151, Fig. B5 and C2
Galeocerdo cuvier, Reif, Pg. 229, Fig. M1 and Pg. 231, Fig. B1, B2, and B4
Galeorhinus galeus, Castro, Pg. 361
Galeorhinus galeus, Reif, Pg. 171, Fig. H2 and H3
Heptranchias perlo, Castro, Pg. 28
Hexanchus griseus, Reif, Pg. 103, Fig. B1 and Pg. 105, Fig. H3

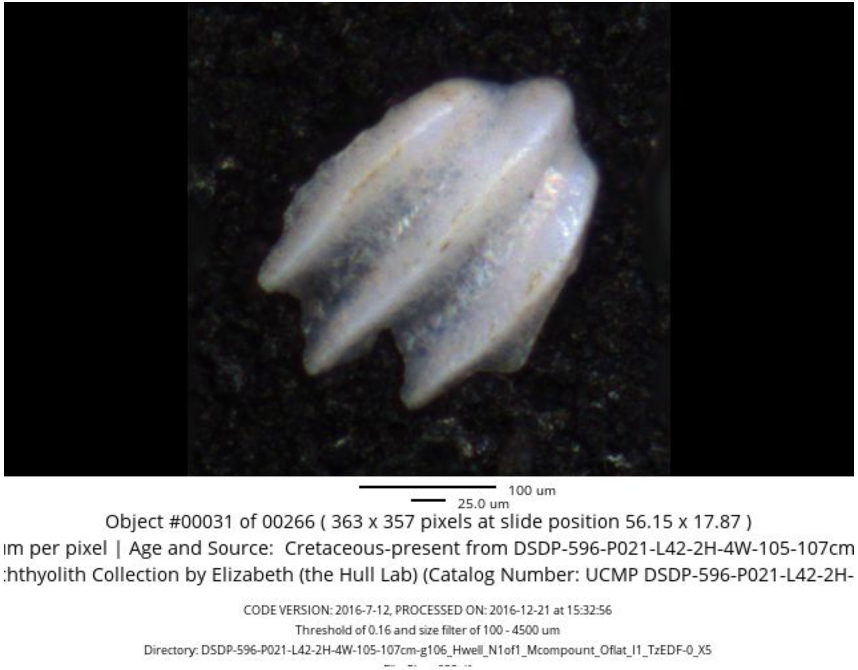

DSDP-596-P021-L42-2H-4W-105-107cm-g106_Hwell_N1of1_obj00031

Trident

The trident type has a pointed spade shape with an anterior vertex and three identical cusps defined by ridges which make up the pointed posterior. The side ridges define the edges of the crown. The ridges are similar in shape but the central ridge is longer than the side ridges.

Taxonomic Citations

Isogomphodon oxyrhynchus, Castro, Pg. 475
Isurus oxyrinchus, Reif, Pg. 145, Fig. H1, H3, B5, and B2, and Pg. 147, Fig. H3 and C1
Negaprion brevirostris, Reif, Pg. 224, Fig. H2, H3, and B5
Notorynchus cepedianus, Castro, Pg. 38
Odontaspis taurus, Reif, Pg. 137, Fig. H2, H3, and C3
Oxynotus centrina, Reif, Pg. 120, Fig. P1
Prionace glauca, Reif, Pg. 214, Fig. H3, B4, and B5, Pg. 215, Fig. C1, C2, C3, P1, and P2, Pg. 217, Fig. H3, B1, Pg. 218, Fig. B2 and P3, Pg. 220, Fig. H2, H3, and B1, and Pg. 221, Fig. B2, B3, and P2
Pristiophorus schroederi, Castro, Pg. 160
Sphyrna lewini, Reif, Pg. 233, Fig. H1, B2, and M2
S. tudes, Reif, Pg. 235, Fig. H2, H3, B4, and P1, and Pg. 237, Fig. P3
S. zygaena, Reif, Pg. 240, Fig. B1 and P3, Pg. 242, Fig. H1 and H3, Pg. 243, Fig. P3, and Pg. 244, Fig. M1 and M2
Squalus acanthias, Reif, Pg. 114, Fig. DF, Pg. 118, Fig. DF and B3
Squatina japonica, Reif, Pg. 128, Fig. B4
S. squatina, Reif, Pg. 126, Fig. B4
Triaenodon obesus, Reif, Pg. 212, Fig. C2
Triakis scyllium, Reif, Pg. 169, Fig. P2 and B2

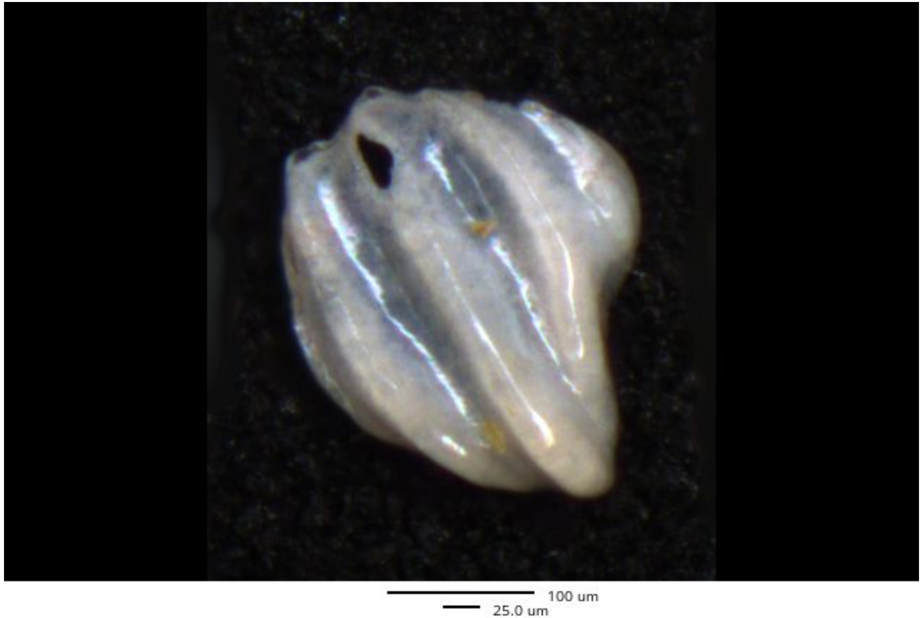

Object #00040 of 00229 (356 x 405 pixels at slide position 62.19 x 17.61)
ım per pixel | Age and Source: Cretaceous-present from DSDP-596-P022-L43-2H-4W-129-131cm
:hthyolith Collection by Elizabeth (the Hull Lab) (Catalog Number: UCMP DSDP-596-P022-L43-2H-

CODE VERSION: 2016-7-12, PROCESSED ON: 2016-12-21 at 15:33:08
Threshold of 0.16 and size filter of 100 - 4500 um
Directory: DSDP-596-P022-L43-2H-4W-129-131cm-g106_Hwell_N1of1_Mcompount_Oflat_I1_TzEDF-0_X5

DSDP-596-P022-L43-2H-4W-129-131cm-g106_Hwell_N1of25_obj00040

Trident with One Side Outgrowth

The trident with one side outgrowth has a rounded spade shape with three ridges, the middle of which is longer than the two side ridges, and a significantly smaller “outgrowth” ridge next to one of the side ridges. Unlike many other trident types the trident with one side outgrowth does not have cusps and the ridge “outgrowth” can be found either at the posterior or anterior of the crown.

Fossil Only

Trident with Pointed Base

The trident with pointed base has a triangular shape with three ridges which run parallel to each other. The anterior is a vertex and the posterior has a pointed edge texture with three identical short cusps.

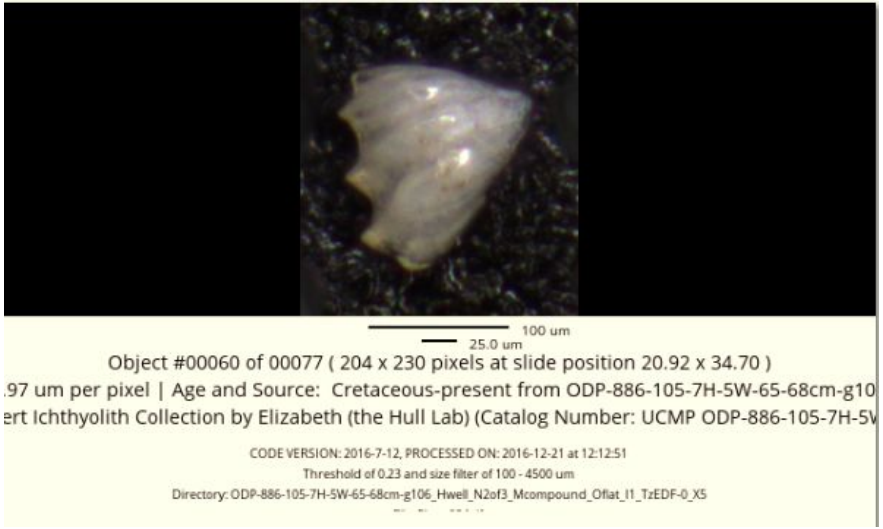

ODP-886-105-7H-5W-65-68cm-g106_obj00060_edf

Fossil Only

Trident with Rounded Base

The trident with rounded base has a rounded spade shape. It has a rounded anterior and pointed posterior. This type has three ridges with a slightly longer central ridge and shorter side ridges which define the edges of the crown.

Taxonomic Citation
Scymnodon ichiharai, White et al. (2015), Fig. a

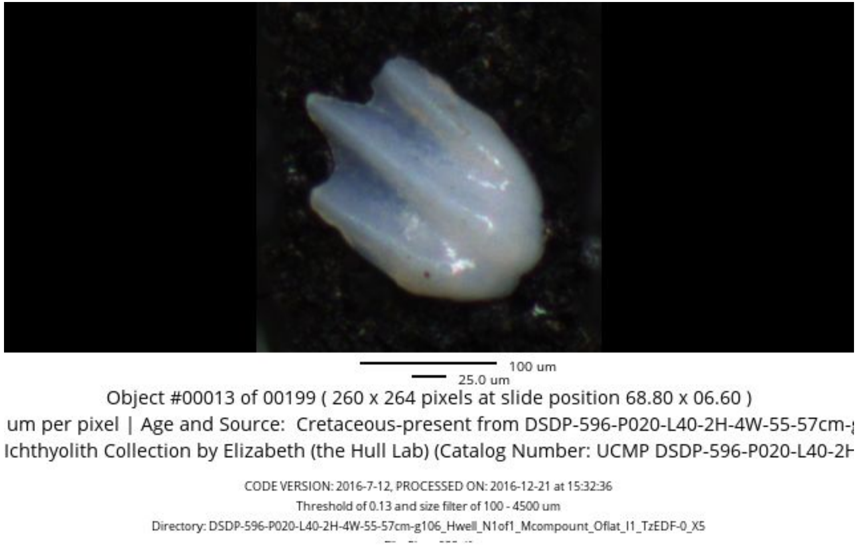

DSDP-596-P020-L40-2H-4W-55-57cm-g106_Hwell_N1of1

Triple Dimpled Crown

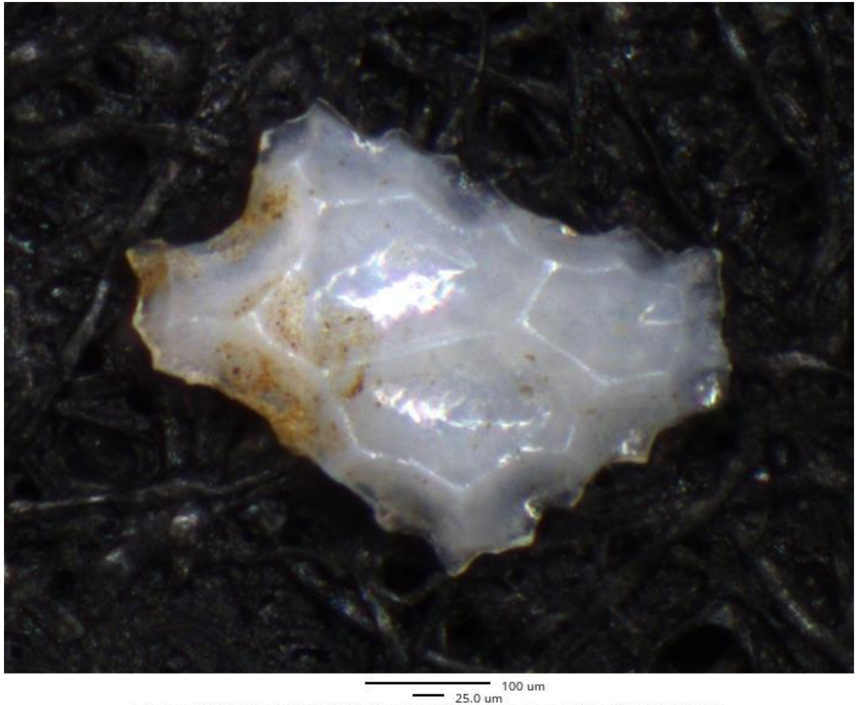

Object #00002 of 00072 (699 x 552 pixels at slide position 37.84 x 04.24)
97 um per pixel | Age and Source: Cretaceous-present from DSDP-596-P047-M51-2H-6W-135-137cm-g1(
ert Ichthyolith Collection by Elizabeth (the Hull Lab) (Catalog Number: UCMP DSDP-596-P047-M51-2H-6W-

CODE VERSION: 2016-7-12, PROCESSED ON: 2016-12-21 at 15:56:39
Threshold of 0.20 and size filter of 100 - 4500 um
Directory: DSDP-596-P047-M51-2H-6W-135-137cm-g106_Hwell_N1of1_Mcompount_Oflat_I1_TzEDF-0_X5

DSDP-596-P047-M51-2H-6W-135-137cm-g106_obj00002

The triple dimpled crown has a diamond-like shape with distinctly serrated edges. It has over 20 thin meandering ridge segments which create surround the crown and three ridges which divide the interior of the crown. One longer ridge segment branches into two shorter segments that create three hexagonal or heptagonal cells with a dimple in each.

Fossil Only

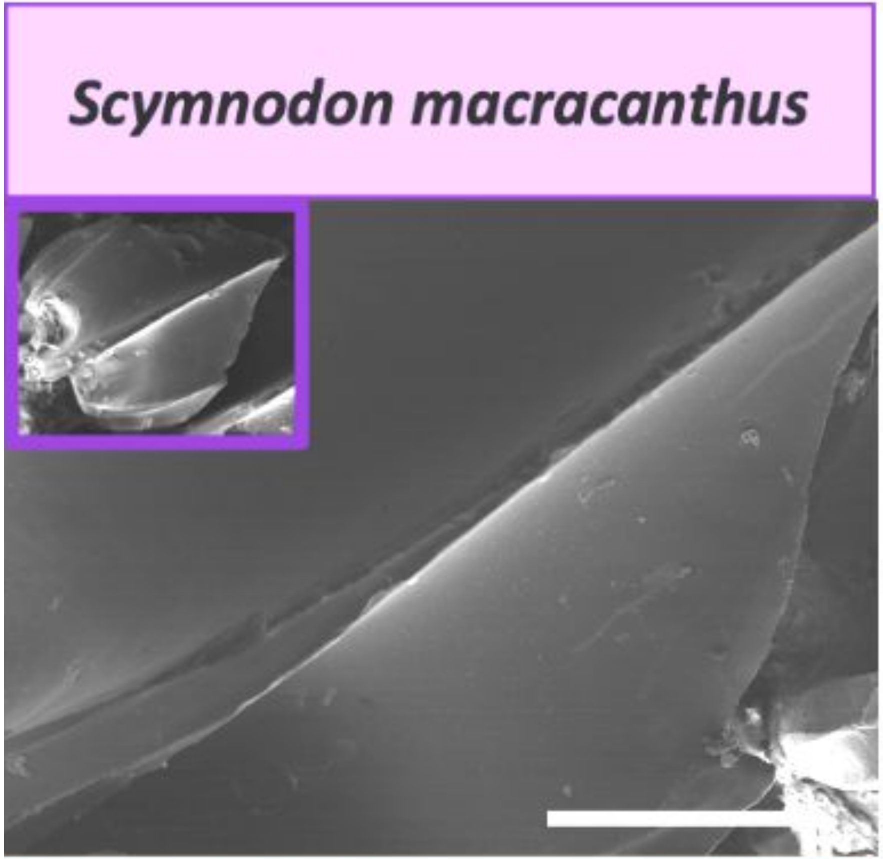

Scymnodon macracanthus, Lourtie et al. 2022

Triple Ridged Petal

The triple ridged petal type had a pointed spade shape and no cusps. It has a longer central ridge and two shorter side ridges which diverge from the anterior.

Taxonomic Citations

Scymnodon macracanthus, Lourtie et al. 2022

Modern Only

Troughed Diamond

The troughed diamond type has a diamond shape with 2 central ridges which run the length of the crown and diverge from the anterior until reaching the crown's center before converging at the posterior creating a central trough.

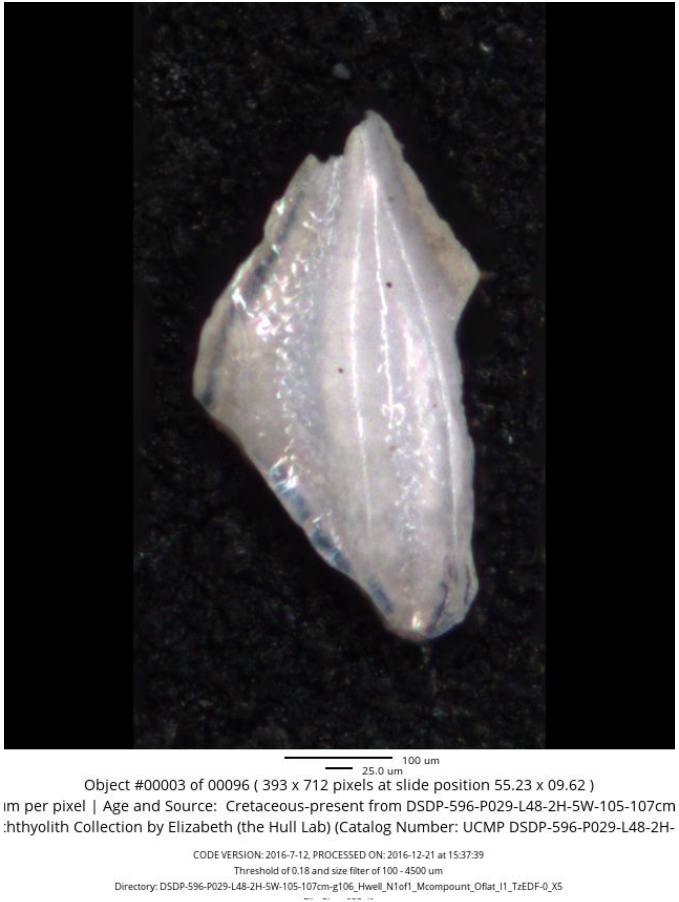

DSDP-596-P029-L48-2H-5W-105-107cm-g106_Hwell_N1of1_obj00003

Fossil Only

Central Ridged Textured Circle

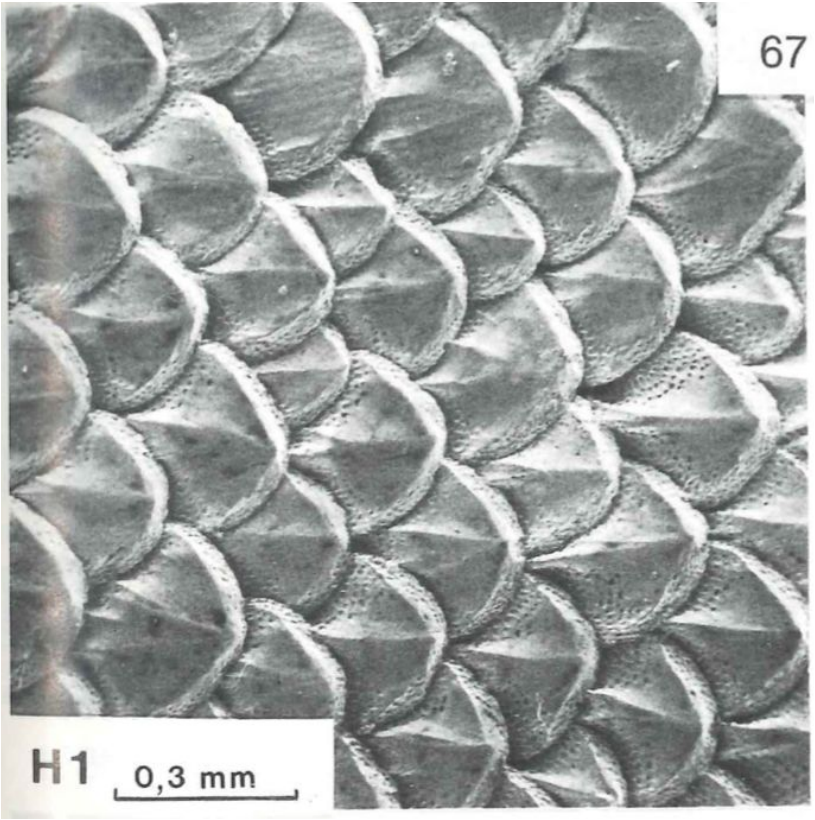

Galeus melastomus, Reif, Pg. 155, Fig. H1

The Central Ridged Textured Circle has a circular shape and either one central ridge or two central ridges which converge from the anterior creating a central trough. If the crown has two central ridges they converge at ~ ¾ the length of the denticle and continue as one ridge the rest of the length. The crown also displays honeycomb-like surface texture.

Taxonomic Citations
Galeus melastomus, Reif, Pg. 155, Fig. H1
Galeus melastomus, Reif, Pg. 153, Fig. H1

Modern Only

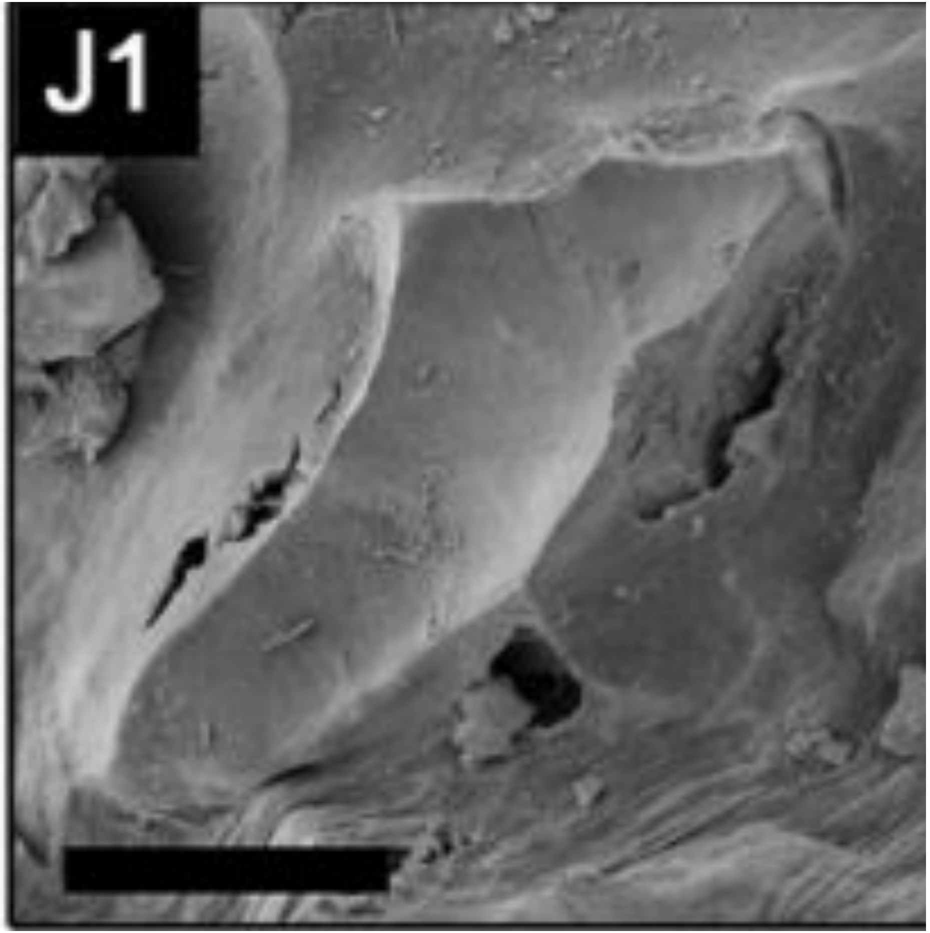

Etmopterus pusillus, Feichtinger et al. (2021), Fig. J1

Tunnel

The tunnel type has an elongated asymmetrical shaped crown which is composed almost entirely of one irregularly shaped "thumbprint" depression, meaning that it does not have clearly definable surrounding ridges. The depression has more than five vertices around the vertical sides of the crown where thin ridge-like lines run down the vertical sides of the crown, This type is very similar to the volcano type and only differs in shape and height above the skin surface (with the volcano being higher above).

Taxonomic Citations

Etmopterus pusillus, Feichtinger et al. (2021), Fig. J1

Modern Only

Two Big Lobes

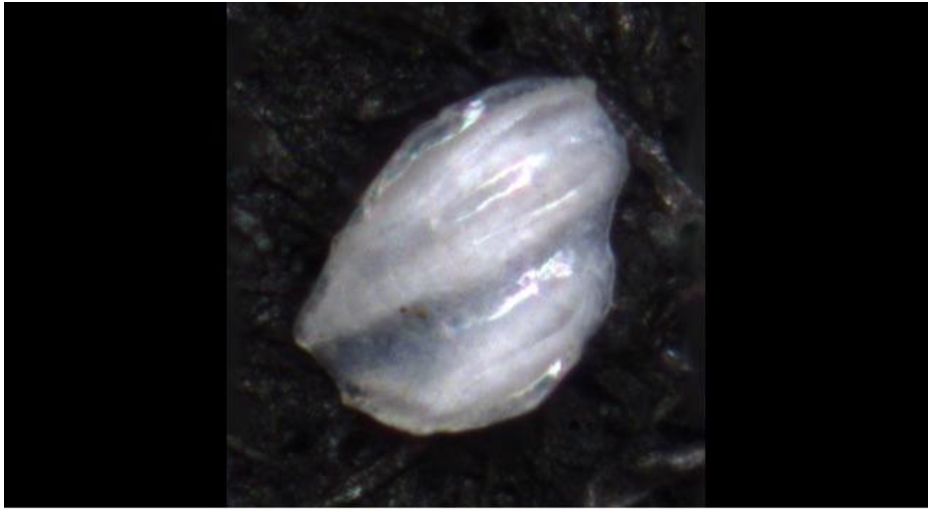

100 um
25.0 um
Object #00003 of 00072 (331 x 350 pixels at slide position 56.88 x 05.92)
m per pixel | Age and Source: Cretaceous-present from DSDP-596-P047-M51-2H-6W-135-137cm
hthyolith Collection by Elizabeth (the Hull Lab) (Catalog Number: UCMP DSDP-596-P047-M51-2H-

CODE VERSION: 2016-7-12, PROCESSED ON: 2016-12-21 at 15:56:39
Threshold of 0.20 and size filter of 100 - 4500 um
Directory: DSDP-596-P047-M51-2H-6W-135-137cm-g106_Hwell_N1of1_Mcompount_Oflat_I1_TzEDF-0_X5

DSDP-596-P047-M51-2H-6W-135-137cm-g106_obj00003

The two big lobes has a round/oval shape with two distinct sections; the larger section is composed of two ridges which converge from the anterior and create a central trough and the second is composed of one ridge which is curved toward the larger section at the anterior. The edges of the crown are defined by the ridges and extend the entire length of the crown.

Fossil Only

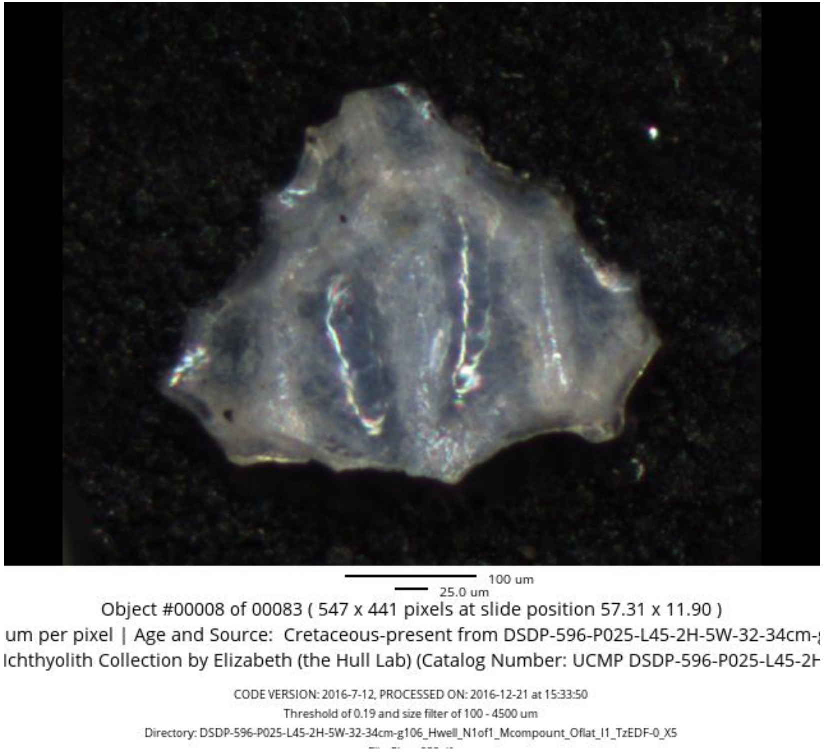

DSDP-596-P025-L45-2H-5W-32-34cm-g106_Hwell_N1of1_obj00008

Two Dimpled Fan

The two dimpled fan type has a fan shape and is wider than it is long. The crown is surrounded by ridges and three ridges extend through the interior of the crown. One central ridge bisects the crown creating two oblong non-circular dimples on either side. The edge texture around the denticle is scalloped at each edge ridge termini.

Taxonomic Citations

Chlamydoselachus anguineus, MCZ 34247
Etmopterus sheikoi, Lourtie et al. 2022

Two Dimpled Fan with Tail

The two dimpled fan with tail type has a fan like shape with a pointed anterior and rounded posterior. The denticle is bisected by a central ridge which defines the length of the crown with four side ridges (two on either side) which branch out from the center. Between the two side ridges and the central ridge on either side is a dimple.

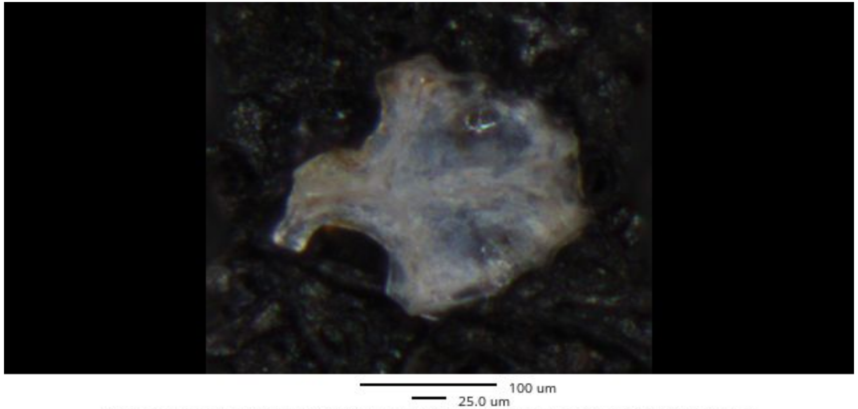

Object #00006 of 00062 (336 x 280 pixels at slide position 26.14 x 12.36)
um per pixel | Age and Source: Cretaceous-present from DSDP-596-P026-M08-2H-5W-42-44cm-
chthyolith Collection by Elizabeth (the Hull Lab) (Catalog Number: UCMP DSDP-596-P026-M08-2H

CODE VERSION: 2016-7-12, PROCESSED ON: 2016-12-21 at 15:34:22
Threshold of 0.18 and size filter of 100 - 4500 um
Directory: DSDP-596-P026-M08-2H-5W-42-44cm-g106_Hwell_N1of1_Mcompount_Oflat_I1_TzEDF-0_X5

DSDP-596-P026-M08-2H-5W-42-44cm-g106_Hwell_N1of1_obj00006

Fossil Only

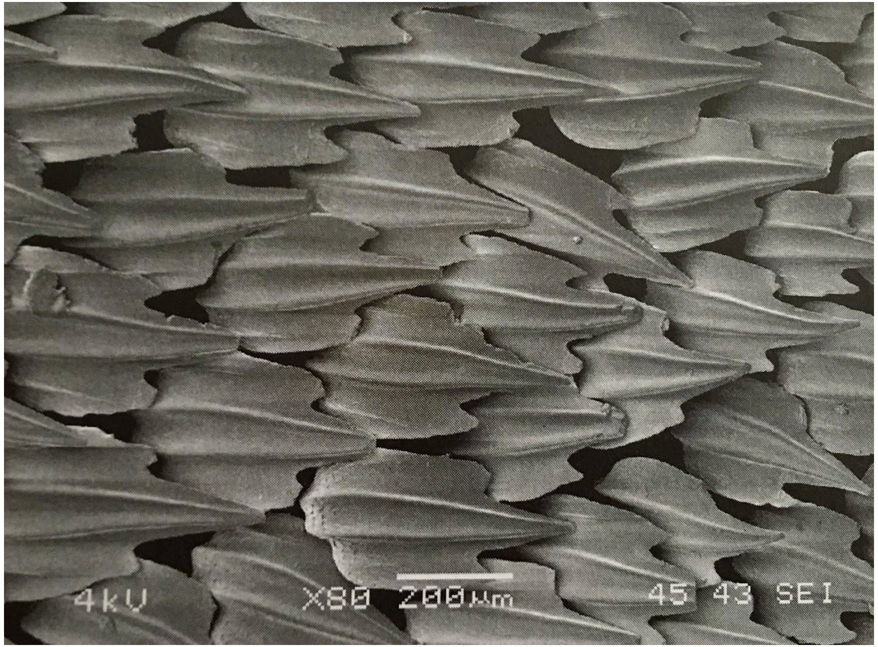

Mustelus henlei, Castro, Pg. 374

Two Ridged Troughed Trident

The Two Ridged Troughed Trident type has a pointed spade shape with an elongated central cusp composed of two ridges converging from the anterior which create a central trough. On either side of this central trough are smooth ridgeless cusps.

Taxonomic Citations

Mustelus henlei, Castro, Pg. 374
Squatina heteroptera, Castro, Pg. 170
Squatina mexicana, Castro, Pg. 172
Schroederichthys maculatus, Castro, Pg. 334
Eridacnis barbouri, Castro, Pg. 350
Mustelus sinusmexicanus, Castro, Pg. 382
Mustelus canis, Ankhelyi et al., Pg. 41, Fig. D
Mustelus canis, Ankhelyi et al., Pg. 42, Fig. E
Galeus melastomus, Reif, Pg. 153, Fig. C1
Galeus melastomus, Reif, Pg. 155, Fig. C2
Mustelus mustelus, Reif, Pg. 165, Fig. H2
Mustelus mustelus, Reif, Pg. 165, Fig. H3
Carcharhinus obscurus, Reif, Pg. 187, Fig. P3
Carcharhinus plumbeus, Reif, Pg. 194, Fig. P3
Negaprion brevirostris, Reif, Pg. 224, Fig. B4
Scyliorhinus retifer, Gabler-Smith et al. (2021), Fig. C

Modern Only

Two Dimpled Rectangle

The two dimpled rectangle type has a rectangular shape and is surrounded on all sides by ridges. One ridge bisects the crown creating two dimples on either side.

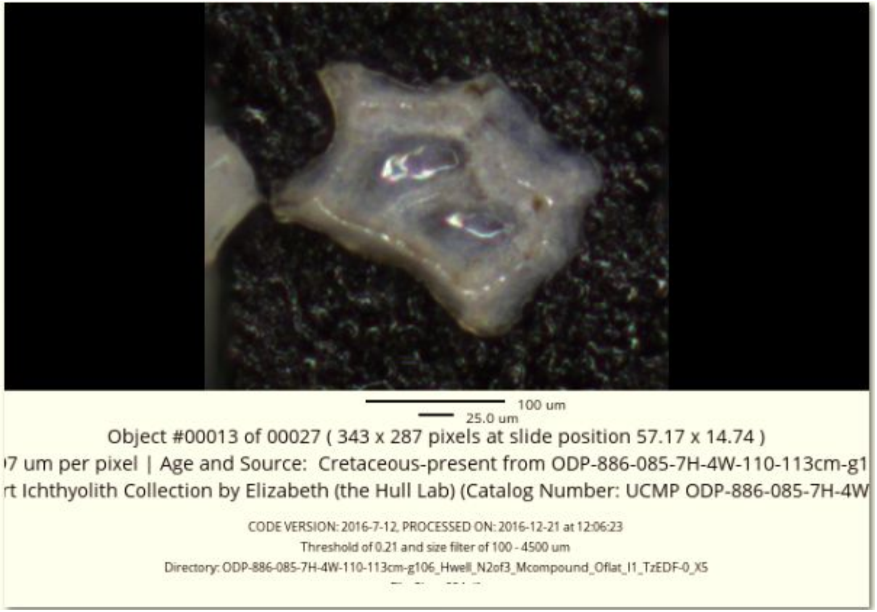

ODP-886-085-7H-4W-110-113cm-g106_obj00013_edf

Fossil Only

Two Ridged Wedge

The two ridged wedge has an arrow shape with two defined ridges which extend the length of the crown. The ridges diverge from the posterior or anterior and one ridge is closer to the center of the crown than the other and ~⅓ longer.

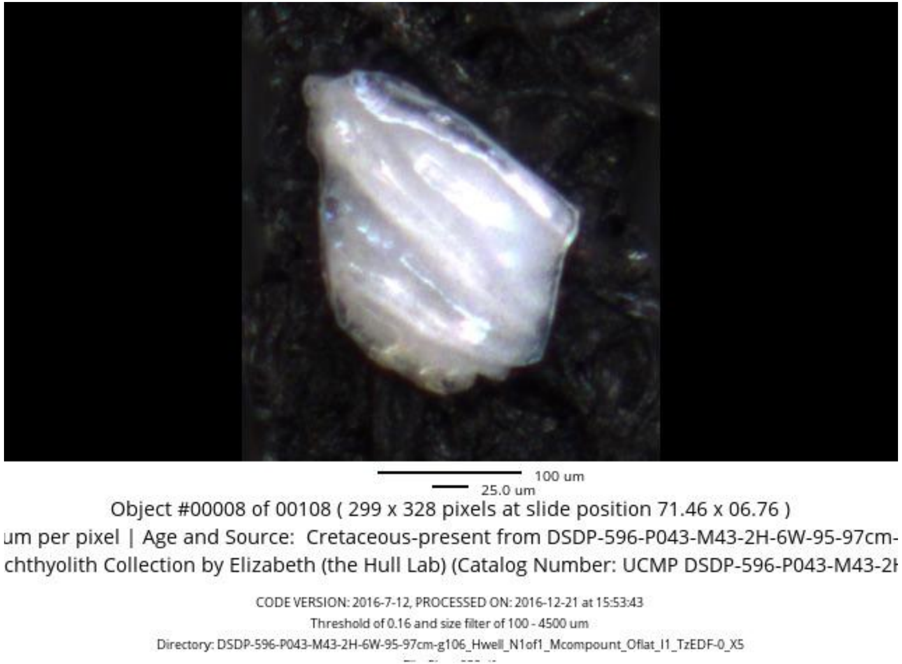

DSDP-596-P043-M43-2H-6W-95-97cm-g106_obj00008

Fossil Only

Two Sectioned Wedge

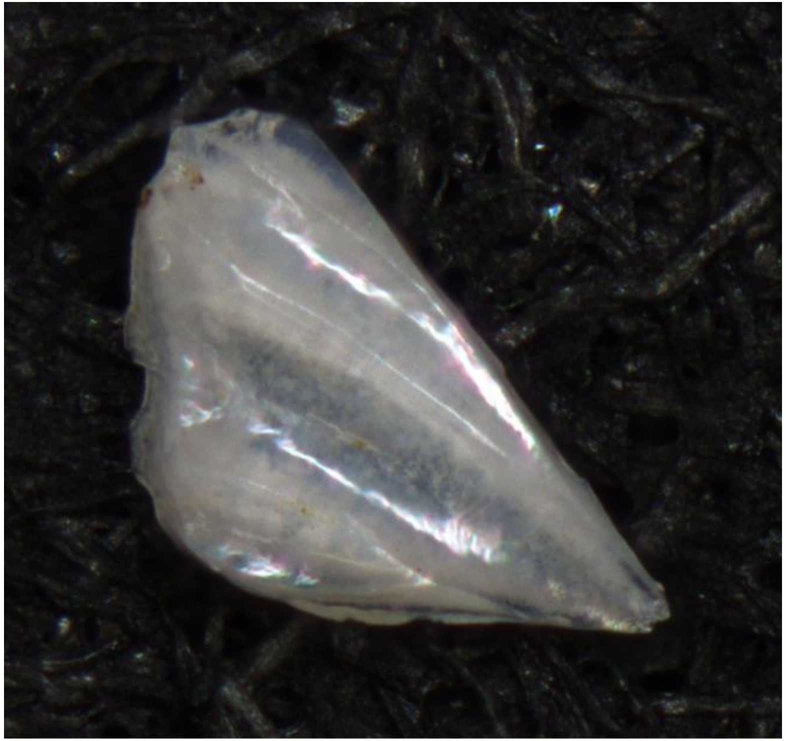

100 um
25.0 um
Object #00015 of 00069 (657 x 622 pixels at slide position 30.01 x 22.53)
' um per pixel | Age and Source: Cretaceous-present from DSDP-596-P042-M41-2H-6W-85-87cm-g
: Ichthyolith Collection by Elizabeth (the Hull Lab) (Catalog Number: UCMP DSDP-596-P042-M41-2H-

CODE VERSION: 2016-7-12, PROCESSED ON: 2016-12-21 at 15:53:43
Threshold of 0.19 and size filter of 100 - 4500 um
Directory: DSDP-596-P042-M41-2H-6W-85-87cm-g106_Hwell_N1of1_Mcompount_Oflat_I1_TzEDF-0_X5

DSDP-596-P042-M41-2H-6W-85-87cm-g106_obj00015

The two sectioned wedge has an obtuse triangular shape with two distinct sections which diverge from the anterior or posterior; each section is composed of two converging or diverging ridges with a central trough. One section is larger and runs the length of the crown while the other is ~ ½ its length and width. Between the two sections is a deep trough ~ ⅓ the width of the crown.

Fossil Only

Volcano

The volcano type has an irregular shaped crown which is composed almost entirely of one irregularly shaped “thumbprint” depression, meaning that it does not have clearly definable surrounding ridges. The depression has more than four vertices around the vertical sides of the crown where thin ridge-like lines run down the vertical sides of the crown at each of these vertices. This type is similar to the “tunnel” type but differs in length and height above the skin surface (with volcano being shorter in length and taller above the skin).

Taxonomic Citations

Etmopterus schultzi, Reif, Pg. 108, Fig. H1
Etmopterus pusillus, Feichtinger (2021), Fig. E
Manta alfredi, Marshall, Compagno, and Bennett, Fig. 14 (C)

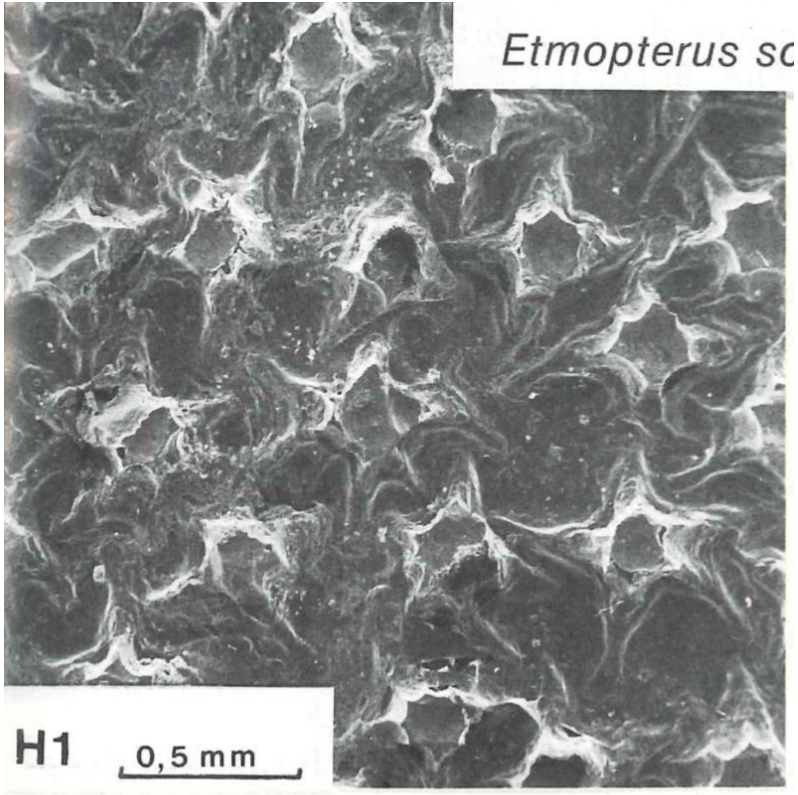

Etmopterus schultzi, Reif, Pg. 108, Fig. H1

Modern Only

Wavey Trident

The wavey trident has a squared spade shape with a rounded anterior and three thin ridges, with a shorter central ridge and longer side ridges which curve slightly towards the anterior. The anterior of the crown has a distinctly serrated texture that is not defined by the ridge termini.

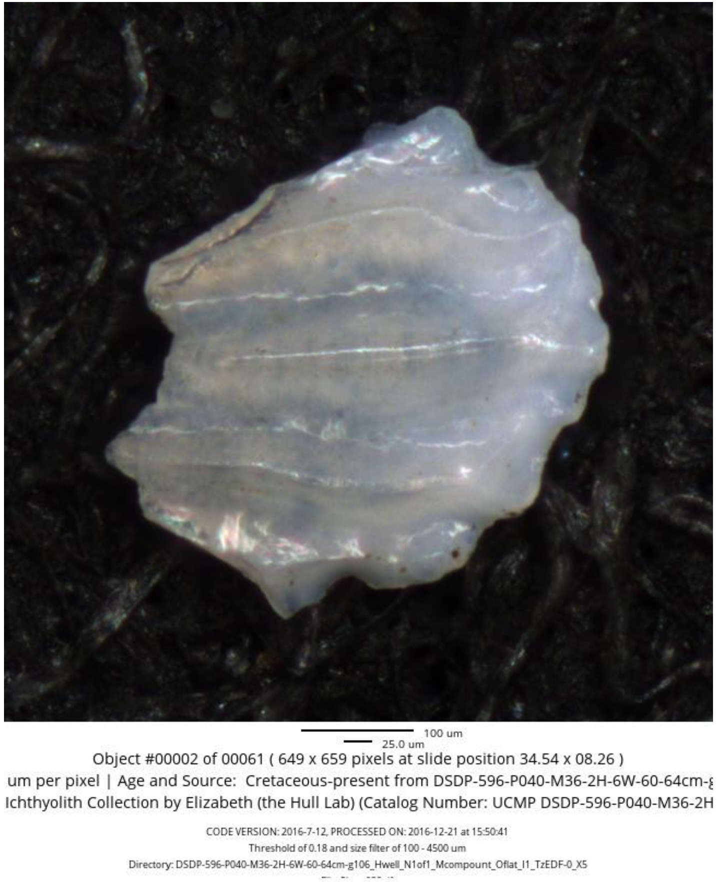

DSDP-596-P040-M36-2H-6W-60-64cm-g106_obj00002

Fossil Only

Wedged Branching Crown

The wedged branching crown has an obtuse triangular shape with a flat posterior or anterior and a pointed posterior or anterior. There are two ridges which begin at either the anterior or posterior and branch out into 5+ ridges which diverge from two ridges. The branching ridges extend along the length of the crown.

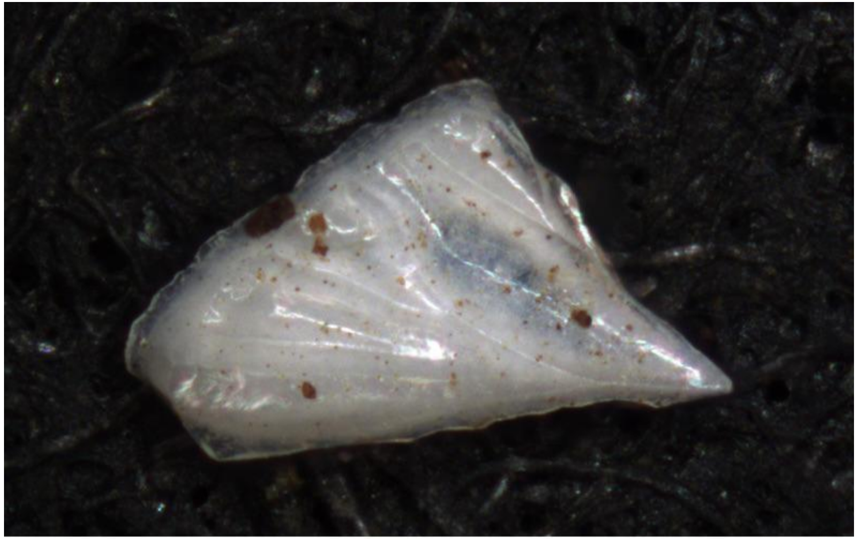

100 um
25.0 um
Object #00003 of 00082 (793 x 499 pixels at slide position 63.23 x 08.95)
0.97 um per pixel | Age and Source: Cretaceous-present from DSDP-596-P035-M25-2H-5W-141-143cm-g106
at Sibert Ichthyolith Collection by Elizabeth (the Hull Lab) (Catalog Number: UCMP DSDP-596-P035-M25-2H-5W-141-14[illegible]

CODE VERSION: 2016-7-12, PROCESSED ON: 2016-12-21 at 15:46:03
Threshold of 0.21 and size filter of 100 - 4500 um
Directory: DSDP-596-P035-M25-2H-5W-141-143cm-g106_Hwell_N1of1_Mcompount_Oflat_I1_TzEDF-0_X5
[illegible]

DSDP-596-P035-M25-2H-5W-141-143cm-g106_obj00003

Fossil Only

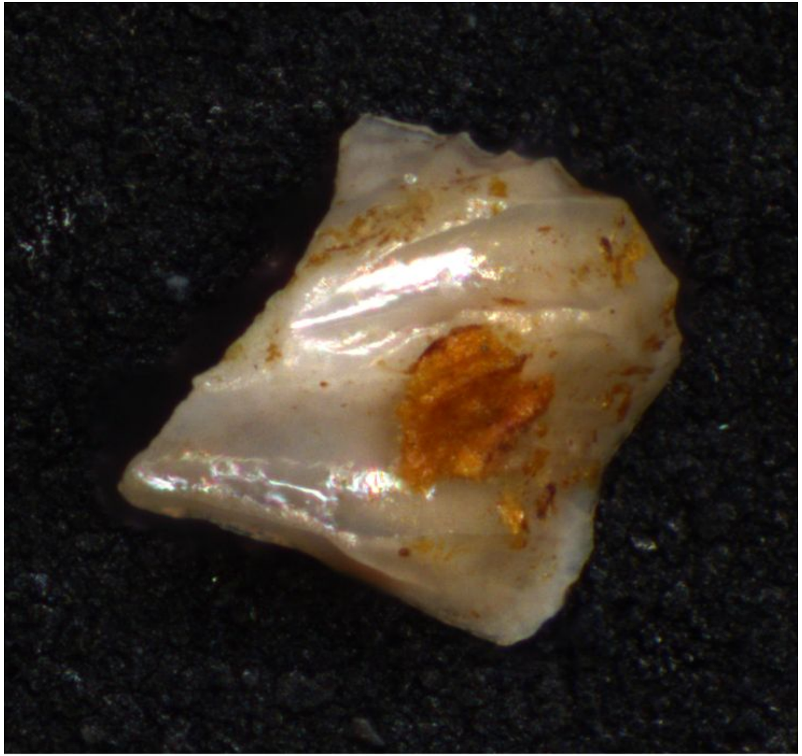

DSDP-596-P033-L50-2H-6W-5-7cm-g106_Hwell_N1of1_obj00004

Whale Blow

The whale blow type has a fan shape and an anterior with an acute angle which fans out towards the posterior. This type can be broken up into three sections with two small triangular side areas and one large bulbous central ridge. The side areas may be broken up into multiple ridges or may stand alone as an individual ridge. This type is characterised by the meandering micro-reliefs found on the central ridge which are shallow and begin ~½ way on the length the ridge..

Fossil Only

Wide Thin Ridged Trident

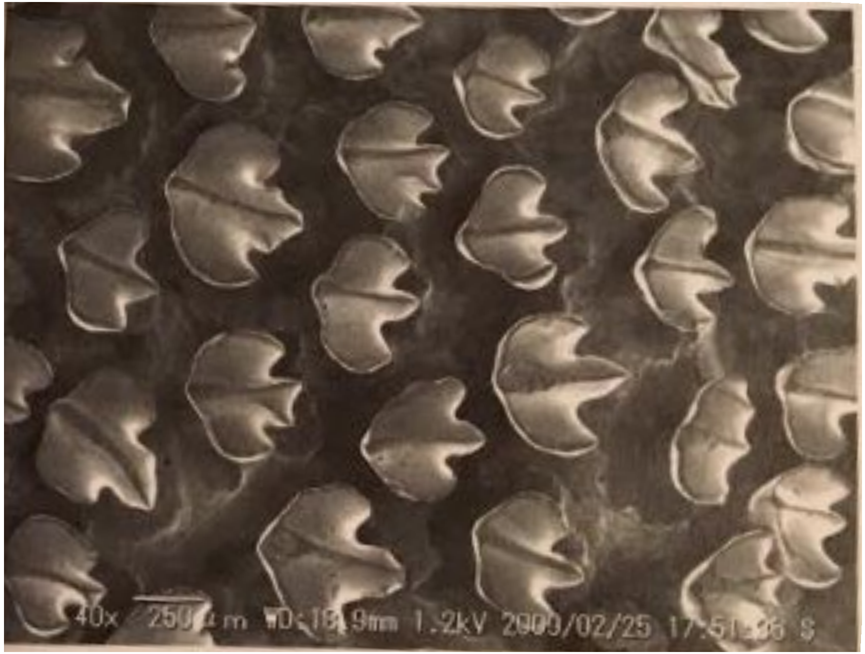

Carcharias taurus, Castro, Pg. 208

The wide thin ridges trident has a rounded spade type and is wider than it is long. The type has three cusps which are defined by ridges. The middle cusp and ridge are distinct and straight while the side ridges are slightly curved. The ridges are relatively thin and have more space between them than other trident types.

Taxonomic Citation
Carcharias taurus, Castro, Pg. 208

Widespread Branching Crown

The widespread branching crown has an irregular shape with a central ridge and four ridges which branch off on either side. The ridges are roughly symmetric on either side of the central ridge with the two lower branches being ~2 times the length of the top branches.

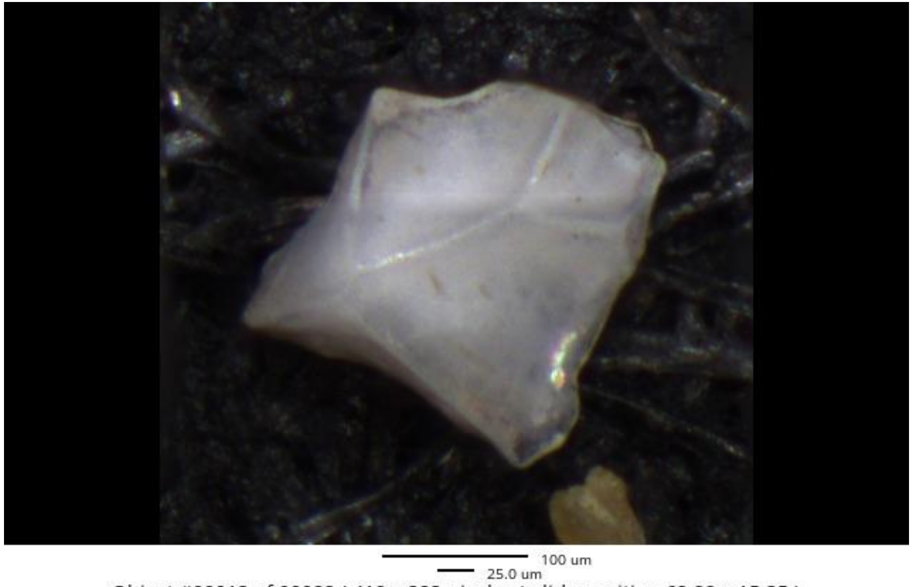

Object #00013 of 00082 (419 x 383 pixels at slide position 62.22 x 15.35)

m per pixel | Age and Source: Cretaceous-present from DSDP-596-P034-M24-2H-5W-135-137cm

hthyolith Collection by Elizabeth (the Hull Lab) (Catalog Number: UCMP DSDP-596-P034-M24-2H-

CODE VERSION: 2016-7-12, PROCESSED ON: 2016-12-21 at 15:43:47

Threshold of 0.22 and size filter of 100 - 4500 um

Directory: DSDP-596-P034-M24-2H-5W-135-137cm-g106_Hwell_N1of1_Mcompount_Oflat_I1_TzEDF-0_X5

DSDP-596-P034-M24-2H-5W-135-137cm-g106_obj00013

Fossil Only

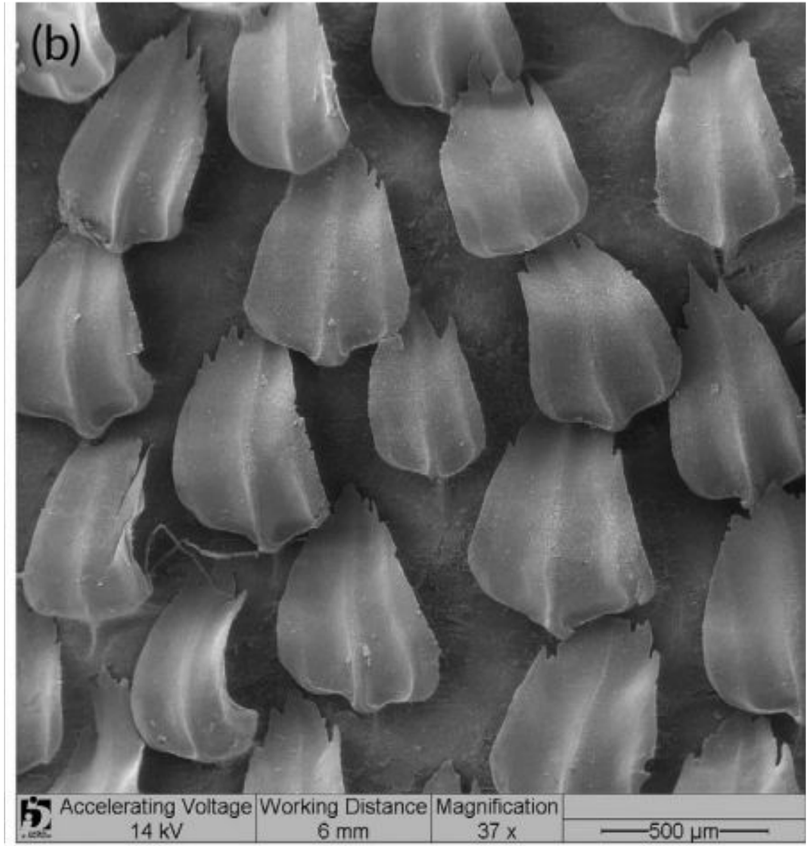

Scymnodon ichiharai, Vaz (2021), Fig. b

Wilted tulip

The wilted tulip type has a stretched spade shape and a distinctly serrated posterior. The crown is longer than wide and has three ridges which extend the length of the crown. This type is characterized by the angle of the crown which protrudes from the skin peaking in height and then curving back towards the skin.

Taxonomic Citation
Scymnodon ichiharai, Vaz (2021), Fig. b

Modern Only

Wishbone

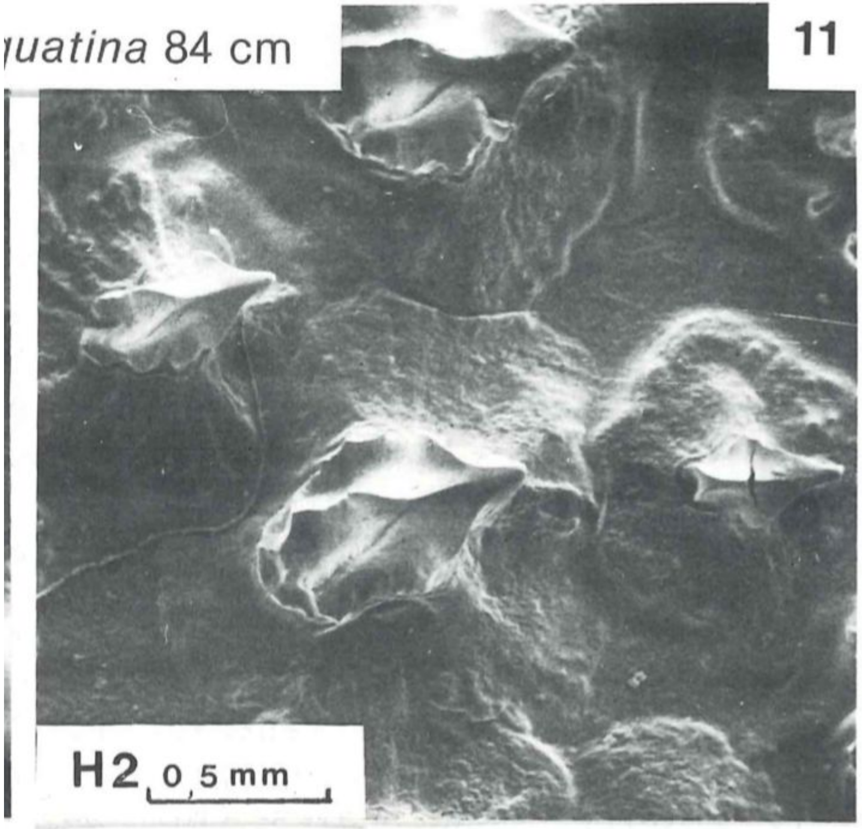

Squatina squatina, Reif, Pg. 126 Fig. H2

The wishbone type is characterized by an oval shape with one central ridge which begins at the posterior or anterior of the crown and ~ ½ the length of the crown branches off into two ridges that stretch to the posterior or anterior of the crown.

Modern Only

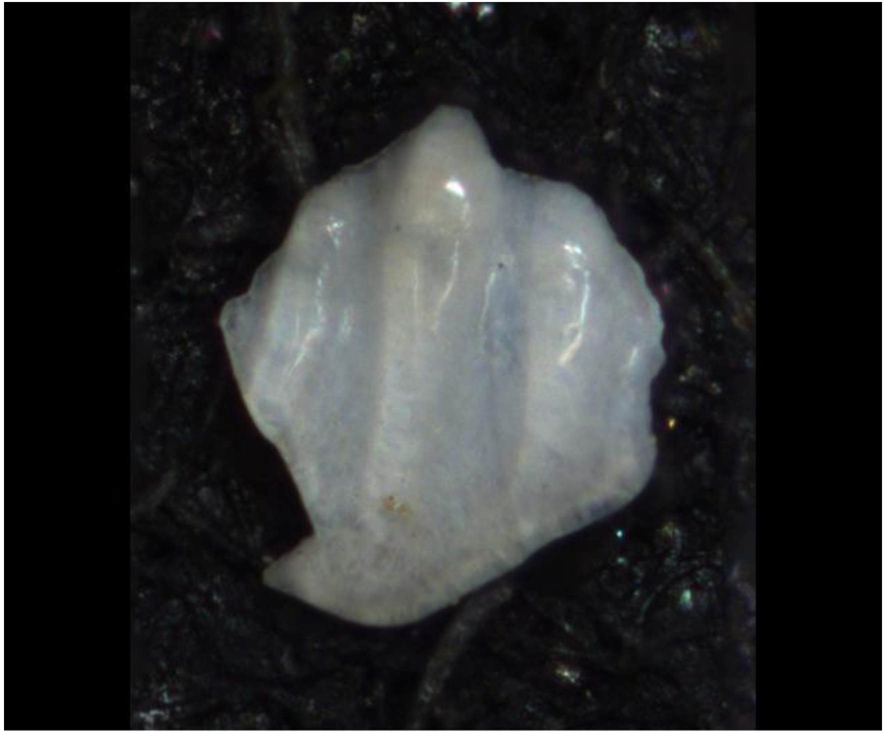

100 um
25.0 um

Object #00006 of 00082 (456 x 531 pixels at slide position 16.51 x 11.58)

m per pixel | Age and Source: Cretaceous-present from DSDP-596-P034-M24-2H-5W-135-137cm

hthyolith Collection by Elizabeth (the Hull Lab) (Catalog Number: UCMP DSDP-596-P034-M24-2H-

CODE VERSION: 2016-7-12, PROCESSED ON: 2016-12-21 at 15:43:47

Threshold of 0.22 and size filter of 100 - 4500 um

Directory: DSDP-596-P034-M24-2H-5W-135-137cm-g106_Hwell_N1of1_Mcompount_Oflat_I1_TzEDF-0_X5

DSDP-596-P034-M24-2H-5W-135-137cm-g106_obj00006

Wrapped Trident

The wrapped trident has a pointed spade shape with three parallel triangular ridges which thin towards the posterior. The central ridge is longer and wider than the two side ridges and the ridges define only the anterior of the crown. The posterior and side edges are composed of flat smooth surface which encapsulate the ridges.

Fossil Only

Wrinkley

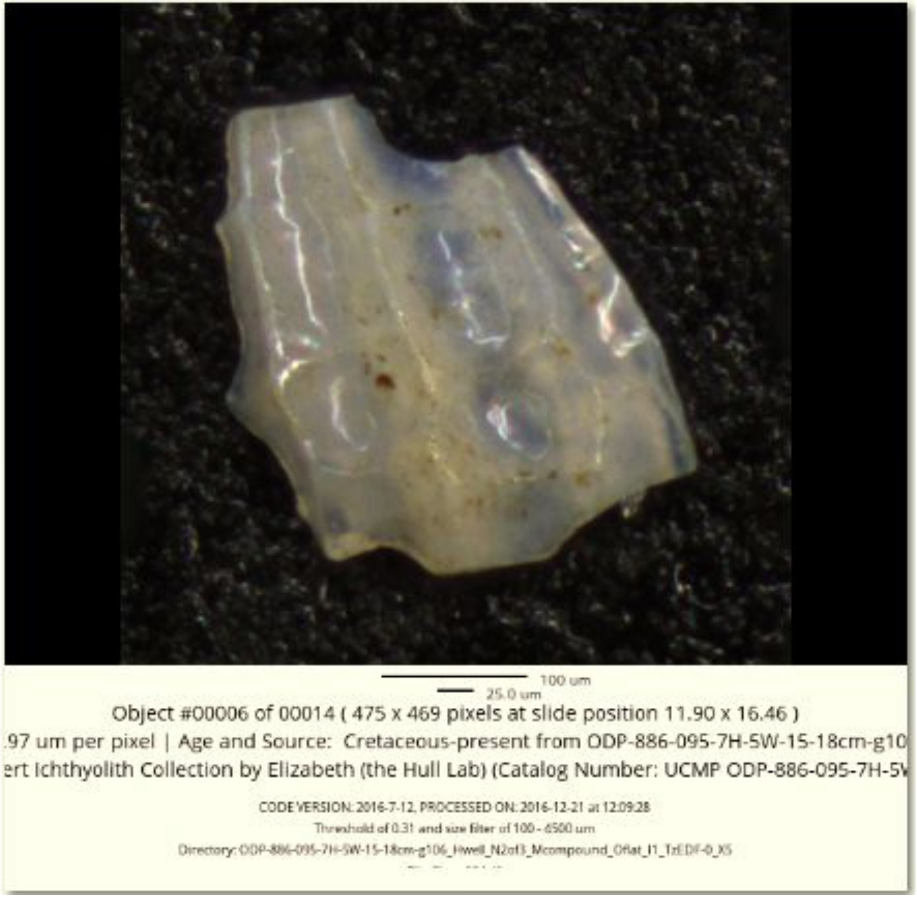

ODP-886-095-7H-5W-15-18cm-g106_obj00006_edf

The wrinkly type has a pointed spade shapel It is a geometric denticle with meandering ridges which form multiple cells. However, they are different than the many celled type due to the non uniformity of these cells. There ridges are thicker from anterior to posterior and thinner ridges compose the cells.

Fossil Only

Wrinkly Kite

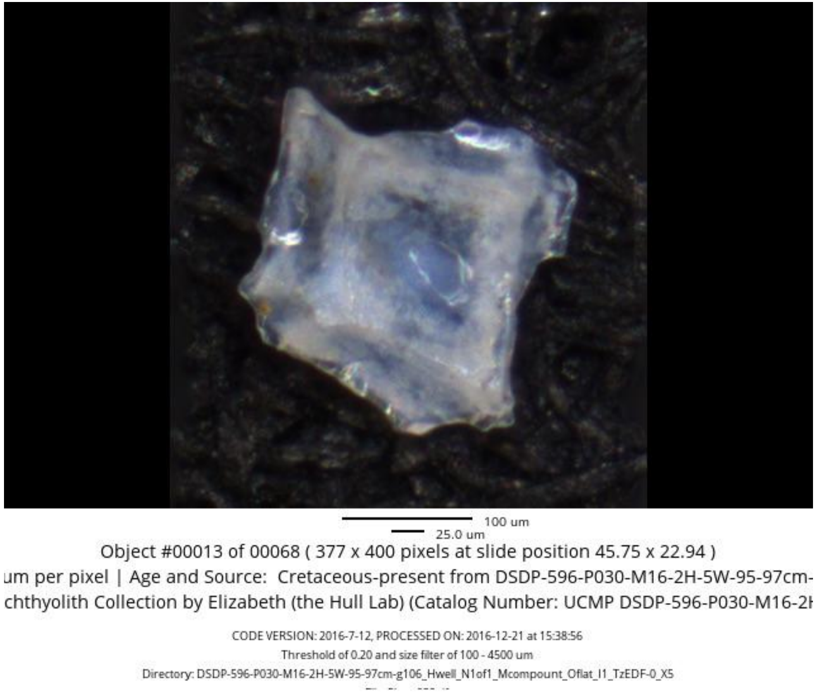

DSDP-596-P030-M16-2H-5W-95-97cm-g106_obj00013

The wrinkly kite has a cruciform shape with four vertices and four straight and/or meandering ridges which compose the central shape which outlines one oval dimple. The vertices of the central shape may have short ridges which branch out. The ridges do not define the edge shape which is outlined by a flat area of the crown.

Fossil Only

Zebra Hoof

The zebra hoof type has a pointed spade shape and four ridges which diverge from the anterior. The middle 2 ridges define the length of the crown and the side ridges are shorter and begin ~ ⅓ up the length of the central ridges and terminate at the posterior of the crown.

Taxonomic Citation
Squatina varii, Vaz, D. F., & de Carvalho, M. R. (2018), Fig.B

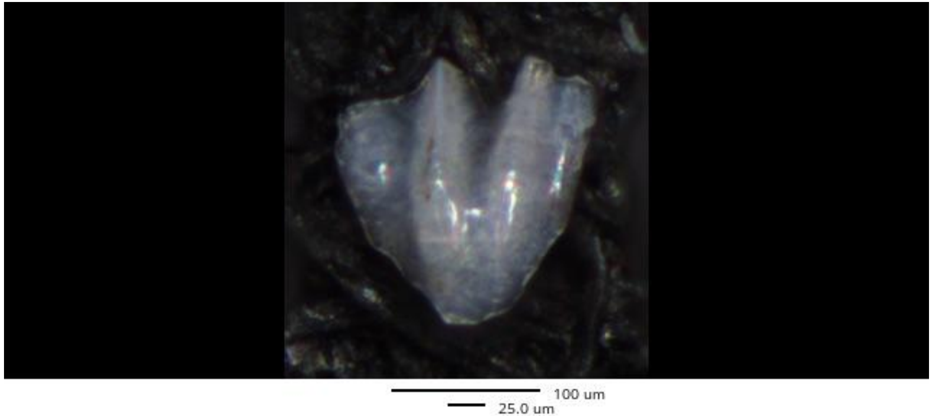

Object #00007 of 00068 (252 x 261 pixels at slide position 32.35 x 16.62)
um per pixel | Age and Source: Cretaceous-present from DSDP-596-P030-M16-2H-5W-95-97cm-
chthyolith Collection by Elizabeth (the Hull Lab) (Catalog Number: UCMP DSDP-596-P030-M16-2H

CODE VERSION: 2016-7-12, PROCESSED ON: 2016-12-21 at 15:38:56
Threshold of 0.20 and size filter of 100 - 4500 um
Directory: DSDP-596-P030-M16-2H-5W-95-97cm-g106_Hwell_N1of1_Mcompount_Oflat_I1_TzEDF-0_X5

DSDP-596-P030-M16-2H-5W-95-97cm-g106_obj00007
