# Supplementary material for: Quantifying the Denticle Multiverse: A Standardized Coding System to Capture Three Dimensional Morphological Variations for Quantitative Evolutionary and Ecological Studies of Elasmobranch Denticles
Source: Integr Org Biol. 2025 May 13;7(1):obaf021. doi: 10.1093/iob/obaf021 (PMC12576789; doi:10.1093/iob/obaf021)
Supplement: obaf021_Supplemental_Files [file obaf021_supplemental_files.zip › Appendix_05-Example Denticle R Vignette.pdf]

```
##############################################
#                                            #
#     Example Code for Denticle Morphology   #
#     analysis, including workflow           #
#     and notes for recreating figures       #
#              v0.5, 15 Aug 2024             #
#                                            #
##############################################

# Welcome to this R script for calculating morphological disparity between
# dermal denticles or fish teeth. Note that while the general workflow for
# denticles and teeth is the same, the underlying trait matrices are
completely
# independent for each microfossil type, and therefore denticles and teeth
# should be analyzed independently. You will be able to define which
# character coding scheme you are using within the R function.

# For Denticles you will use the traits and weights objects for denticles.
# The most recent versions are
# ichthyoliths::denticle_traits_v0.5 #trait matrices
# ichthyoliths::denticle_weights_v0.5 #weights for each trait

# The morphological disparity function (distances_clust) can take a long time
# and a lot of computing resources to run depending on the number of objects
you
# are comparing. This is because it is calculating a pairwise distance
between
# every single object in the dataset. The more objects, the more comparisons.
# Thus, I strongly recommend saving the calculated distances dataset as an
.RData
# object and also as standalone .csv files (example code below). This will
# allow you to come back to these analyses and pick up where you left off
rather
# than re-running the distances function. In a future version of this R
package,
# I will have a "add objects" function, but at present, if you add objects to
# your datasets, you'll have to re-run the distances function in full.

##### General Workflow #####
# Step 0: Load the libraries
# Step 1: Call in the coded morphology matrix and define the columns which
#     contain numerical character state values. At this point you may also
#     clean up the dataset spreadsheet or define additional metadata columns
```

```
# Step 2: Run the disparity calculation (distances_clust function) and check
for errors
# Step 3: Ordinate the distance matrix (NMDS) and save the ordination
coordinates to the matrix
# Step 4: Plot the dataset!

########################################
#                                      #
#     Step 0: Set up the workspace     #
#                                      #
########################################

##### Libraries #####
# run this whole section every time, even if you've used a .RData file for
disparity calculations
library(ichthyoliths) #For ichthyolith distance calculations and range chart
library(doParallel) #For calculating disparity efficiently using parallel
computing
library(vegan) #for NMDS analyses
library(viridis) #for the range chart colors
library(rgl) #for 3D plotting - not necessary for functionality of the
package, but very fun

# # To install the ichthyoliths package from Github, run the following code:
# library(devtools)
# install_github('esibert/ichthyoliths', force = TRUE) #force = TRUE
overwrites any prior installation

##############################################
#                                            #
#     Step 1: Call in the datasets           #
#             and specify the version of     #
#             ichthyolithMorph used          #
#                                            #
##############################################

##### 1a. Define trait disparity matrices and define weights vector (or use
defaults) #####
# The package has pre-loaded the traits and weights for denticle morphology
v0.4 and v0.5.
# Note that for all future releases, the manuscript will specify the version
used,
# and updated versions will be added as appropriate.
```

```
# If you want to define your own trait matrices or use this architecture to
# analyse an entirely different type of fossil, the way to import/organize
the
# trait CSVs is included at the end of this script.

# ## Use the package-defined versions of the code (here using denticles_v0.5)
# you can either call this here, or you can call them directly in the
distance function:
# traitset <- ichthyoliths::denticle_traits_v0.5
# weightset <- ichthyoliths::denticle_weights_v0.5

##### 1b. Import coded dataset #####

## Call in the dataset:
# If your dataset is directly from a file using our google drive template,
skip the first line
dentdat <- read.csv('example_code/dentmorph_V0.5_Morphotypes.csv', skip = 1,
header = TRUE, blank.lines.skip = TRUE)

## Figure out which columns hold the numerical data for the coded denticles.
This will likely be the
# last 46 columns of the spreadsheet if you are using our google drive
template.
# To confirm,
colnames(dentdat)
# This displays all the columns. We want columns 57 to 100 from this
spreadsheet

## Which columns hold numerical coding data (e.g. A1-O10)
full_morphcols.morpotypes <- c(57:102) #should be 46 columns



####################################################
#                                                  #
#     Step 2: Running the distances function       #
#                                                  #
####################################################

#### Step 2a: run the distance function #####
dent_distances.morphotypes <- distances_clust(morph = dentdat,
                                              traits = denticle_traits_v0.5,
```

```
                                              weights =
denticle_weights_v0.5,
                                              morphCols =
full_morphcols.morpotypes,
                                              IDCol = 1,
                                              coresFree=2)

### Parameter explanations:
# morph is the coded matrix
# traits is the trait matrices, defined in the R package (as above) or you
can pull in your own
# weights is the weights vector, defined in the R package (as above) or you
can make your own
# morphCols are the columns that contain the morphotype code (this is defined
above)
# IDCol is the column with the unique identifier for each object, usually a
filename or
# coresFree passes to the doParallel loop and defines computing resources
used.

# Note that the distances_clust function is also able to do sensitivity
analyses
#  e.g. discount particular characters, upweight/downweight differently, etc.
#  If you are interested in using this feature to explore your morphospace,
#  please refer to the help file for the distances_clust function.
#  The function also can handle continuous trait values, though at present
this is
#  not well-tested, so use at your own risk.

##### Step 2b: Turn the output of the distance function into a distance
matrix for ordination #####


# make distmat
dent.distmat.morphotypes <- distmat(dent_distances.morphotypes, type = "avg")

# The distance calculation calculates disparity using two different methods:
#  Average disparity across all traits considered
#  Total disparity across all traits considered
# Where there are 0 values coded, those traits are not considered for those
pairs
#  as is standard in such disparity analyses, and allows for effective
cross-comparison
```

```
#  of broken ichthyoliths.
#  Thus typically we use the "average" rather than the "sum" values for
further calculations
#  However, you may choose differently. Feel free to explore the distance
calculation
#  output to see the different metrics calculated and how you want to handle
them.


##### Step 2c (optional): Save the output so you don't have to re-run long
disparity calculations #####
###  Distances calculation output dataframe
write.csv(dent_distances.morphotypes, file =
'example_code/dent_distances.csv', row.names = FALSE)

### Distance matrix
write.csv(dent.distmat.morphotypes, file = 'example_code/dent_distmat.csv',
row.names = FALSE)
# Note that you should also set col.names = FALSE, or remember to include
skip=1
#  when calling the distmat back in. For some reason R throws an error on my
#  computer (but not others) when including col.names = FALSE in the
write.csv command.


####################################################
#                                                  #
#        Step 3: Ordination!                       #
#                                                  #
####################################################

################### DENTICLES ###################

NMDS3.morphotypes <- metaMDS(dent.distmat.morphotypes, k=3, distance =
"euclidean", try = 100, trymax = 100)

#add ordination coordinates to morphology matrix for plotting
# This allows you to plot based on any metadata column you have included in
the original data sheet
dentdat$MDS1 <- NMDS3.morphotypes$points[,1]
dentdat$MDS2 <- NMDS3.morphotypes$points[,2]
dentdat$MDS3 <- NMDS3.morphotypes$points[,3]
```

```
# Save the ordination to csv for later work
write.csv(dentdat, file = 'example_code/dentmorph_ordination.csv', row.names
= FALSE)

####################################################
#                                                  #
#        Step 4: Make Plots!                       #
#                                                  #
####################################################

##### Example Plots: Ridge system type #####

# Graphical Parameters
cols <- c('firebrick', 'goldenrod1', 'green2', 'purple', 'blue', 'gray70')
pchs <- c(21, 24, 22, 23, 8, 25)

##### Plot by coercing code data into factor #####
plot(dentdat$MDS1, dentdat$MDS2, cex = 1.2,
     col = cols[as.factor(dentdat$F1)], bg = cols[as.factor(dentdat$F1)],
     pch = pchs[as.factor(dentdat$F1)],
     xlab = 'MDS1', ylab = 'MDS2')
# Ridge system type is trait F1.
legend('topleft', legend = c('smooth', 'linear','geometric', 'meandering',
'spine', 'Branching'), pch = 16, cex = 1, col = cols)


##### 3D Plot #####
# ## 3d Plot with RGL library, its cool to look at - and for some reason
doesn't work on macs, sorry!
# # see https://r-graph-gallery.com/3d_scatter_plot.html
# plot3d(x = dentdat$MDS1, y = dentdat$MDS2, z = dentdat$MDS3,
#        col = cols[as.factor(dentdat$F1)], pch = 16, radius = 0.05, type =
's')


##### Code for plot using a loop to objects by their character state(s) #####
# Blank plot
plot(dentdat$MDS1, dentdat$MDS2, type = 'n',
     xlab = 'MDS1', ylab = 'MDS2')
for(i in 1:length(unique(dentdat$F1.1))) {
   points(subset(dentdat, dentdat$F1.1 == i, select = c('MDS1', 'MDS2')),
          col = cols[i], bg = cols[i], pch = pchs[i])
}
```

```
# legend
legend('topleft', legend = c('smooth', 'linear','geometric', 'meandering',
'spine', 'Branching'), pch = pchs, cex = 1, col = cols, pt.bg = cols)


##### Figure for Manuscript #####

##### Figure for Manuscript #####
par(mfrow = c(1,3), xpd = NA, oma = c(1, 0, 1, 0))
# mtext("Trait F1: Ridge System Type", side = 3)

## Plot 1: MDS1/MDS2 ##
plot(dentdat$MDS1, dentdat$MDS2, type = 'n',
     xlab = '', ylab = '', axes = F)
box()
mtext('MDS1', side = 1, line = 1)
mtext('MDS2', side = 2, line = 1)
mtext('MDS1 / MDS2', side = 3, line = 0.5, font = 2)
for(i in 1:length(unique(dentdat$F1.1))) {
   points(subset(dentdat, dentdat$F1.1 == i, select = c('MDS1', 'MDS2')),
          col = cols[i], bg = cols[i], pch = pchs[i])
}


## Plot 2: MDS1/MDS3 ##
plot(dentdat$MDS1, dentdat$MDS3, type = 'n',
     xlab = '', ylab = '', axes = F)
box()
mtext('MDS1', side = 1, line = 1)
mtext('MDS3', side = 2, line = 1)
mtext('MDS1 / MDS3', side = 3, line = 0.5, font = 2)
for(i in 1:length(unique(dentdat$F1.1))) {
   points(subset(dentdat, dentdat$F1.1 == i, select = c('MDS1', 'MDS3')),
          col = cols[i], bg = cols[i], pch = pchs[i])
}

## Annotations (add to middle plot) ##
# legend (add to plot #2)
legend('bottom', inset = c(0, -0.28), cex = 1.4,
       legend = c('Smooth', 'Linear','Geometric', 'Meandering', 'Spine',
'Branching'),
       pch = pchs, col = cols, pt.bg = cols,
       #bty = 'n',
```

```
       horiz = T, xjust = 0.5, yjust = 0.5)
mtext("Character F1: Ridge System Type", side = 3, font = 2, line = 3, cex =
1.1)

## Plot 3: MDS2/MDS3
plot(dentdat$MDS2, dentdat$MDS3, type = 'n',
     xlab = '', ylab = '', axes = F)
box()
mtext('MDS2', side = 1, line = 1)
mtext('MDS3', side = 2, line = 1)
mtext('MDS2 / MDS3', side = 3, line = 0.5, font = 2)
for(i in 1:length(unique(dentdat$F1.1))) {
   points(subset(dentdat, dentdat$F1.1 == i, select = c('MDS2', 'MDS3')),
          col = cols[i], bg = cols[i], pch = pchs[i])
}



###########################################
#           EXTRA CODE                    #
###########################################

##### Manually import trait CSV files and define weights #####
# # Traits:
# traitset <- import_traits_csvs(csvname = "Trait*", csvpath =
"data/v0.5/traitCSV_denticles_v0.5/", recurs = F)

# # Here we have given a specific weight to each denticle character; If no
weights are defined, the function assumes equal weight to all traits
# weightset <-
c(2,0.5,1,1,1,1,1,1,1,1,0.5,0.5,0.5,2,1,1,1,0.5,0.5,0.5,0.5,0.5,0.5,1,0.5,0.5
,0.5,0.5,0.5,1,0.5,0.5,0.5,1,0.5,0.5,1,1,1,1,1,1,1,1,1,1)
# names(weightset) <- names(traitset) #assign names to the weights vector to
confirm correct order


##### Find coding errors #####
# Troubleshooting: If you have any character state values that are out of
bounds
# (e.g. coded an "8" where there are only 6 character states), the
distances_clust
# function returns an NA value.
```

```
# Since implementing the dropdown-menu based coding this has not been an
issue, but this is how we
# used to identify coding errors
df.na <- subset(dent_distances.morphotypes,
is.na(dent_distances.morphotypes$dist.sum))

#list of objects that broke the function (if any): (I often use column 3, but
the object IDs (column 8) are also good for this);
# Correct issues and re-run the dat_distances function until this returns an
empty object.
unique(df.na[,8])
```
