# Supplementary material for: Quantifying the Denticle Multiverse: A Standardized Coding System to Capture Three Dimensional Morphological Variations for Quantitative Evolutionary and Ecological Studies of Elasmobranch Denticles
Source: Integr Org Biol. 2025 May 13;7(1):obaf021. doi: 10.1093/iob/obaf021 (PMC12576789; doi:10.1093/iob/obaf021)
Supplement: obaf021_Supplemental_Files [file obaf021_supplemental_files.zip › Appendix_06-Links for manuscript Supplemental Documents and Links DentMorph.pdf]

List of Supplemental Documents and Links for:

Quantifying the denticle multiverse: a standardized coding system to capture three dimensional morphological variation for quantitative evolutionary and ecological studies of elasmobranch denticles

Supplemental Appendices:

Appendix 1: Denticle Table: An expanded tabular version of the morphological character code, with example photographs and citations for each currently observable character state.

Appendix 2: Line Drawings: A two-page formatted summary document of all possible character states in the current (published) version of the denticle morphology code.

Appendix 3: Morphotypes: A slide show documenting all defined unique morphotypes used in this study, with keywords and documentation. The codes for each morphotype are included in Appendix 4 - Morphotype Codes.

Appendix 4: Morphotype Codes: An excel file with the full character coding scheme and all morphotypes defined in the manuscript fully coded. This is also available as a read-only google spreadsheet that can be copied for use by any interested party. This is also included in the R package 'example_code' folder. Please see note below about using the Denticle Coding Spreadsheets.

Appendix 5: Example R Script: Example code for using the *ichthyoliths* R package for denticle morphology, using the morphotypes spreadsheet as input. This is also available through GitHub:
ichhyoliths R package: https://github.com/esibert/ichthyoliths
Example Denticle R Vignette: https://tinyurl.com/ykx46wx6

Denticle coding spreadsheets
This document links readers to two google spreadsheet files which include the previously defined morphotypes and their codes, citations, and relevant taxonomic data. The second file is a blank sheet with dropdown menus linked to the "code" and "unique types" sheets (also found in this spreadsheet). To code your own denticles, simply make a copy of the blank spreadsheet and code by using the dropdown menus, adding additional columns if you need to add additional metadata such as ontogenetic stage or location on the body. Additionally, characters and trait states can be added by editing the "code" sheet and adding associated columns to the "blank" sheet.

Morphotypes - https://tinyurl.com/2hcfkrdk
Blank Spreadsheet - https://tinyurl.com/fffx7e32
