# Supplementary material for: Quantifying the Denticle Multiverse: A Standardized Coding System to Capture Three Dimensional Morphological Variations for Quantitative Evolutionary and Ecological Studies of Elasmobranch Denticles
Source: Integr Org Biol. 2025 May 13;7(1):obaf021. doi: 10.1093/iob/obaf021 (PMC12576789; doi:10.1093/iob/obaf021)
Supplement: obaf021_Supplemental_Files [file obaf021_supplemental_files.zip › Fijian Abstract.pdf]

Abstract:

Na yago ni matavuvale vaka ika me vaka na qio kei na vai e dau dikevi vakawasoma e na veiqaravi nei ira e ra dau vakadidike e na veimataqali vuli e so. Me vaka na vuli veika vaka-ivakarau makawa, na vuli ni veisau ni tuvaki vakayago, na vuli e dau dikeva na duidui ni kena vakayagataki na veitikini yago ni manumanu kei na ituvatuva e dusia na veika e dau vakayacori ena bula kei na nodra itovo na manumanu (bio-inspired design).

Na kena toso na vakadidike ni vuli me baleta na yago ni vuvale vaka ika oqo, e kunekune dredre tiko na kena vakatauvatani na veika e dikevi baleta ni sega ni tiko e dua na yavu dei me cokona vata na veimataqali vuli e sa cavuti oti. Ka mai vakavuna na qiqo ni vakadidike kei na kena vakayagataki na kena ivosa matau e so. E na vuku ni vakadidike oqo, keitou sa vakarautaka e dua na sala vou e rawa ni semata kina na i tukutuku lailai me vaka na yaca ni ika, na kena ibulibuli kei na kena yaga me kilai kina na kedra duidui. Na sala keitou vakayacora e kumuna vata na veiporokaramu e so e na mona livaliva me vaka na google sheet kei na R.

Na yaga ni porokaramu e vukea na kena soqoni ka dikevi na duidui ni tukutuku e kumuni rawa e na vakadidike oqo. Na mona ni porokaramu oqo e vukea na kena waki vata na itukutuku, na itaba, na SEMs kei na yaloyalo vakamatailalai (CT scans) ni yagodra na qio kei na kena vakadikevi lesu na batidra e kune e na boto ni sauloa ka tu mai na dua na drau na milioni na yabaki sa oti.

Na mona ni porokaramu o ya e vakarawarawataka na vakatauvatani ni vakadidike kei na tukutuku e ra kumuna na vei matailawalawa ni vakadidike tudei e liu, ka vakacurumi kina e dua na iwasewase vou ni ivakatakilakila e so.

Na mona ni porokaramu oqo e vakararamataka na kena dikevi na kena tuvaki ni kuli ni yago ni veimataqali ika mai na matavuvale ni qio kei na vai. E rawa ni vakayagataki talega e na kena vakatauvatani ni kuli ni yagodra e na balavu ni gauna e ra bula tu kina se kena bula voli e na veitaba gauna. Na mona ni porokaramu oqo e na vukea na kena kilai vakatitobu kei na kena vakatauvatani na i tuvaki ni kuli ni matavuvale ni ika oqo e na vei vuli eso.
