# Supplementary material for: Quantifying the Denticle Multiverse: A Standardized Coding System to Capture Three Dimensional Morphological Variations for Quantitative Evolutionary and Ecological Studies of Elasmobranch Denticles
Source: Integr Org Biol. 2025 May 13;7(1):obaf021. doi: 10.1093/iob/obaf021 (PMC12576789; doi:10.1093/iob/obaf021)
Supplement: obaf021_Supplemental_Files [file obaf021_supplemental_files.zip › Portuguese Abstract.docx.pdf]

Abstract

Dentículos dérmicos - escamas microscópicas semelhantes a dentes - são uma característica diagnostica importante da pele dos elasmobrânquios e são de interesse para uma ampla variedade de áreas, incluindo paleontologia, biologia evolutiva, biologia do desenvolvimento, morfologia funcional e design bioinspirado. Embora a pesquisa sobre dentículos dérmicos seja uma área em crescimento, atualmente não existe um vocabulário ou estrutura padronizada para comparar a morfologia dos dentículos entre diferentes áreas de pesquisa, o que acaba isolando e limitando os esforços de pesquisa sobre dentículos. Aqui, apresentamos uma estrutura morfológica que inclui um código de caracteres que captura de maneira abrangente a morfologia dos dentículos a partir de uma grande diversidade de tipos de amostras de dentículos e métodos de imagem, e que é respaldada por uma ferramenta de codificação fácil de usar baseada no Google Sheets e por um pacote R para replicar análises de disparidade. O código é baseado em uma ampla revisão da literatura de imagens publicadas de dentículos, MEVs e tomografias computadorizadas (CT) de dentículos de tubarões viventes, além de uma revisão de dezenas de milhares de dentículos fósseis de sedimentos oceânicos pelágicos com mais de 100 milhões de anos. A flexibilidade e replicabilidade do código facilitam a comparação entre estudos e equipes de pesquisa independentes, além da adição de novas categorias de caracteres. Os morfotipos de dentículos são definidos como dentículos com combinações únicas de características. Este sistema de codificação facilita análises de disparidade com base morfológica sobre a diversidade morfológica dos dentículos, seja ao longo do tempo geológico, ao longo do corpo de um tubarão, ou ao longo de uma série temporal de desenvolvimento, oferecendo uma ferramenta mais detalhada, quantitativa e universal para analisar a morfologia dos dentículos em diferentes estudos.
