# Supplementary material for: Quantifying the Denticle Multiverse: A Standardized Coding System to Capture Three Dimensional Morphological Variations for Quantitative Evolutionary and Ecological Studies of Elasmobranch Denticles
Source: Integr Org Biol. 2025 May 13;7(1):obaf021. doi: 10.1093/iob/obaf021 (PMC12576789; doi:10.1093/iob/obaf021)
Supplement: obaf021_Supplemental_Files [file obaf021_supplemental_files.zip › Spanish Abstract.pdf]

Abstract translation to Spanish

Cuantificación del multiverso de dentículos: Un sistema de codificación estandarizado para capturar la variación morfológica tridimensional para estudios cuantitativos evolutivos y ecológicos de dentículos dérmicos de elasmobranquios.

I. Resumen

Los dentículos dérmicos (escamas microscópicas similares a dientes) son una característica definitoria importante de la piel de los elasmobranquios y son de interés para una amplia gama de campos, incluyendo la paleontología, la biología evolutiva, la biología del desarrollo, la morfología funcional y la ingeniería biomimética. Mientras la investigación de los dentículos dérmicos es un campo en expansión, actualmente no existe un vocabulario ni un marco estandarizado para comparar la morfología de los dentículos en diferentes campos de estudio, lo que aísla y limita los esfuerzos de investigación. Aquí se presenta un marco morfológico que incluye un código de caracteres que captura exhaustivamente la morfología de los dentículos dérmicos a partir de una amplia diversidad de tipos de muestreo y métodos de imagen. Este marco está respaldado por una herramienta de codificación fácil de usar basada en hojas de cálculo de Google y un paquete R para replicar análisis de disparidad. El código se basa en una extensa revisión bibliográfica de imágenes publicadas, microscopía electrónica de barrido (MEB) y tomografías computarizadas de dentículos de tiburón existentes, y en una revisión de decenas de miles de dentículos fósiles de sedimentos oceánicos pelágicos que datan de más de 100 millones de años. La flexibilidad y replicabilidad del código facilitan la comparación entre estudios y equipos de investigación independientes, así como la incorporación de nuevas categorías de caracteres. Los morfotipos de dentículos se definen como dentículos que presentan combinaciones únicas de rasgos de carácter. Este sistema de codificación facilita el análisis de disparidad morfológica de la diversidad de los dentículos, ya sea a lo largo de grandes períodos de tiempo, a lo largo del cuerpo de un tiburón o a lo largo de una serie temporal de desarrollo, proporcionando una herramienta más detallada, cuantitativa y universal para el análisis de la morfología de los dentículos dérmicos en diferentes estudios.
